# Supplementary figures and images for: Aorta smooth muscle-on-a-chip reveals impaired mitochondrial dynamics as a therapeutic target for aortic aneurysm in bicuspid aortic valve disease (part 1 of 2)
Source: eLife. 2021 Sep 6;10:e69310. doi: 10.7554/eLife.69310 (PMC8451027; doi:10.7554/eLife.69310)

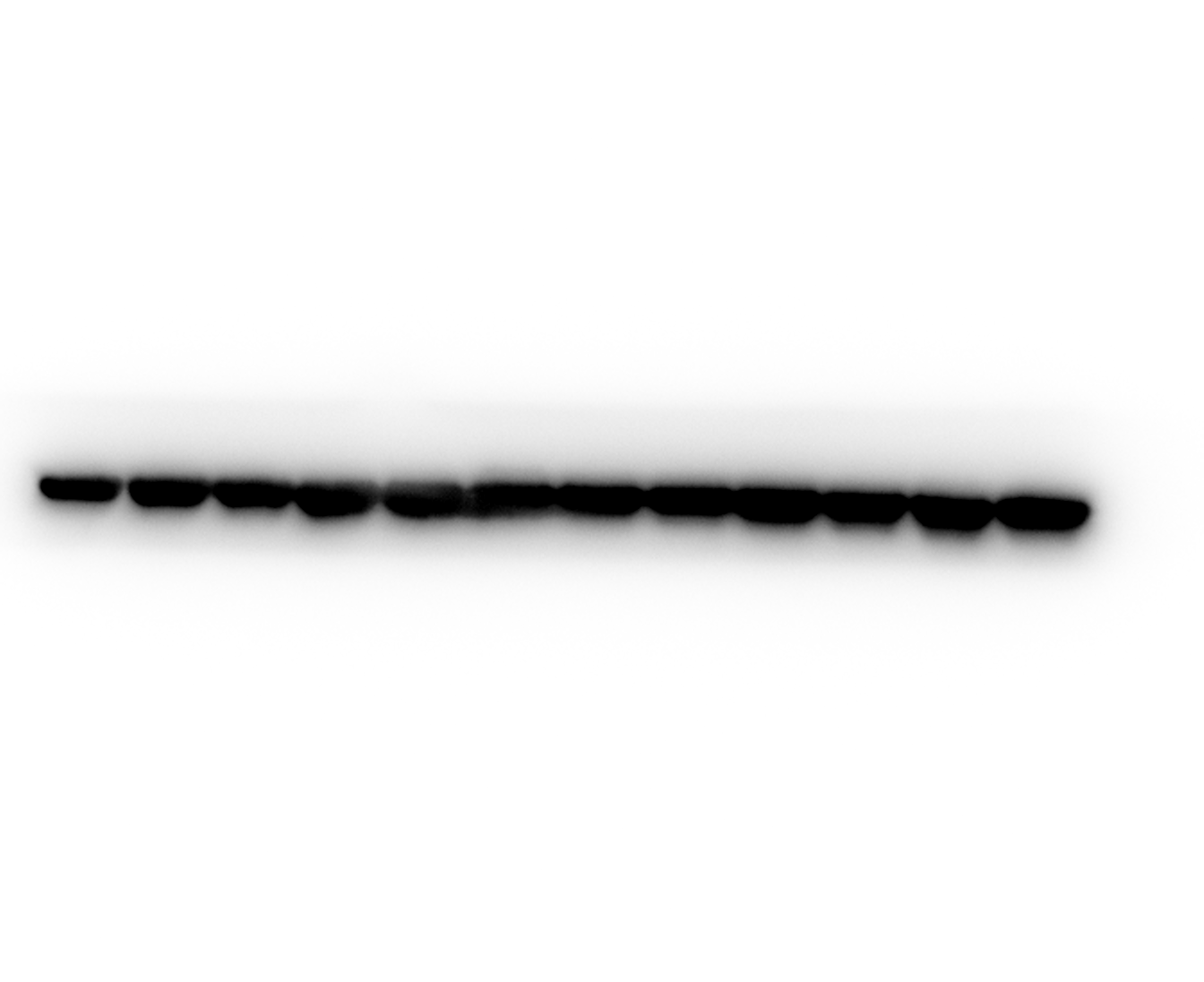

Supplement: Figure 1—source data 1. [file elife-69310-fig1-data1.zip › Figure 1-source data 1/B-ACTIN-001.Tif]

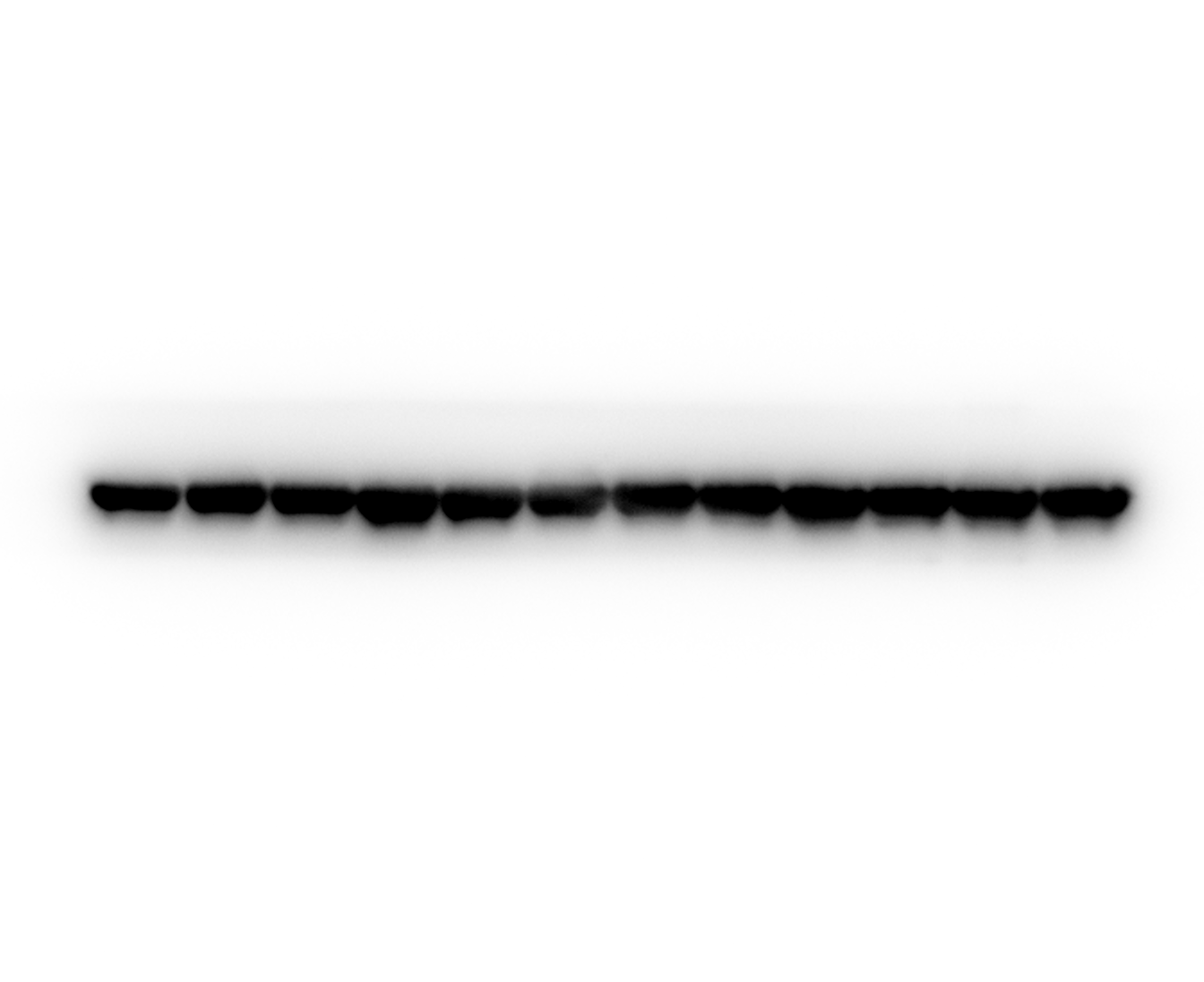

Supplement: Figure 1—source data 1. [file elife-69310-fig1-data1.zip › Figure 1-source data 1/B-ACTIN-002.Tif]

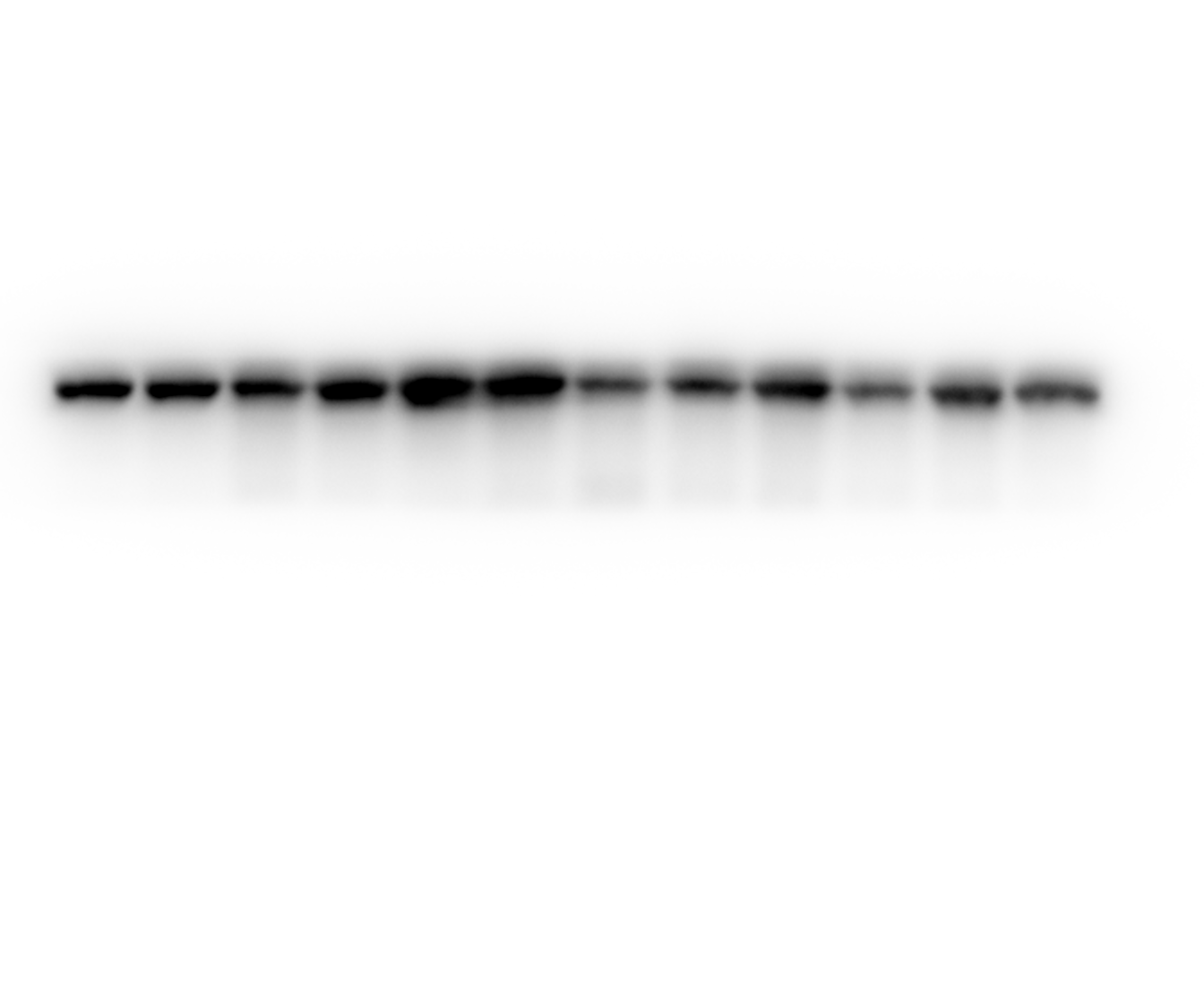

Supplement: Figure 1—source data 1. [file elife-69310-fig1-data1.zip › Figure 1-source data 1/CNN1.Tif]

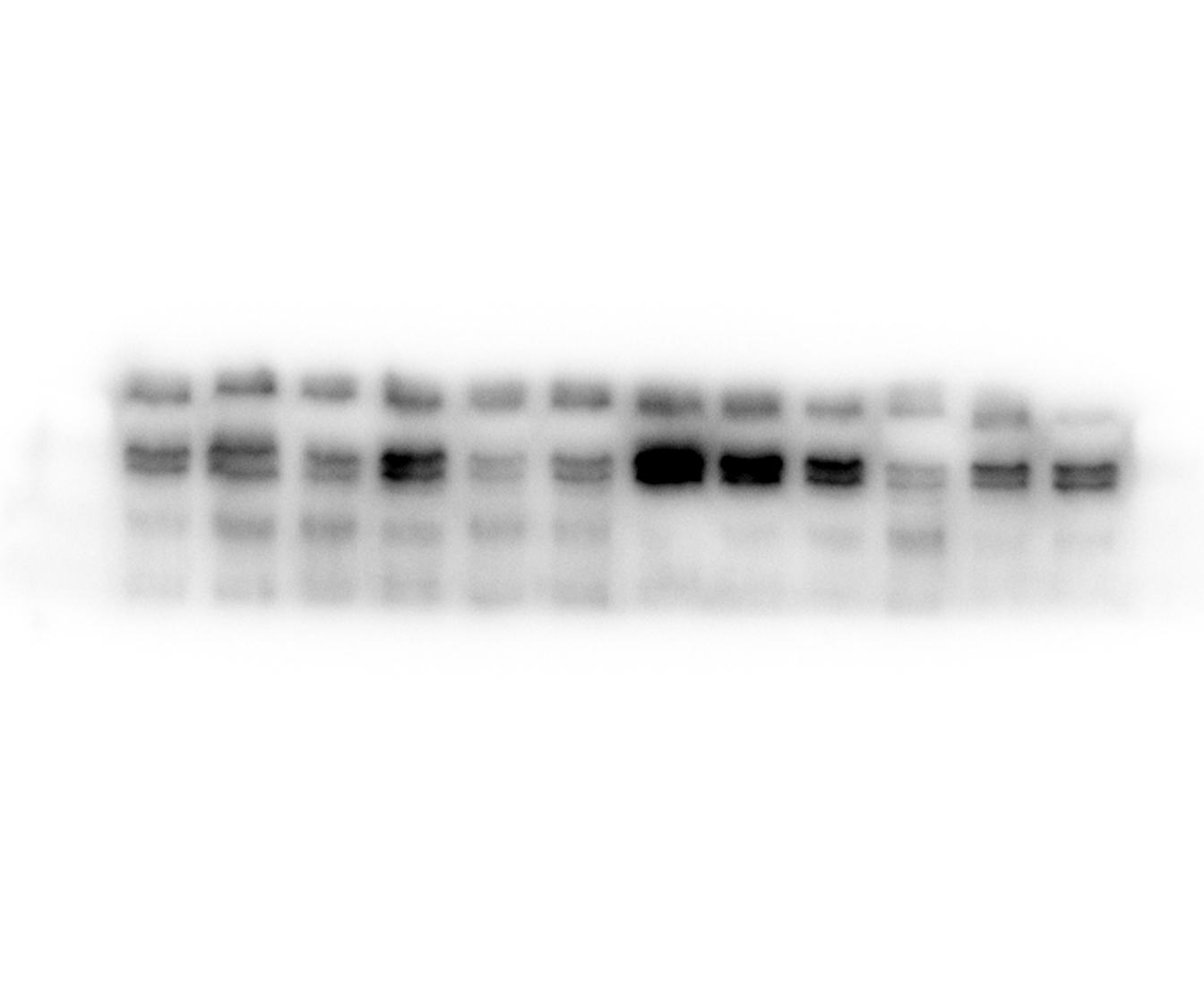

Supplement: Figure 1—source data 1. [file elife-69310-fig1-data1.zip › Figure 1-source data 1/DRP1.Tif]

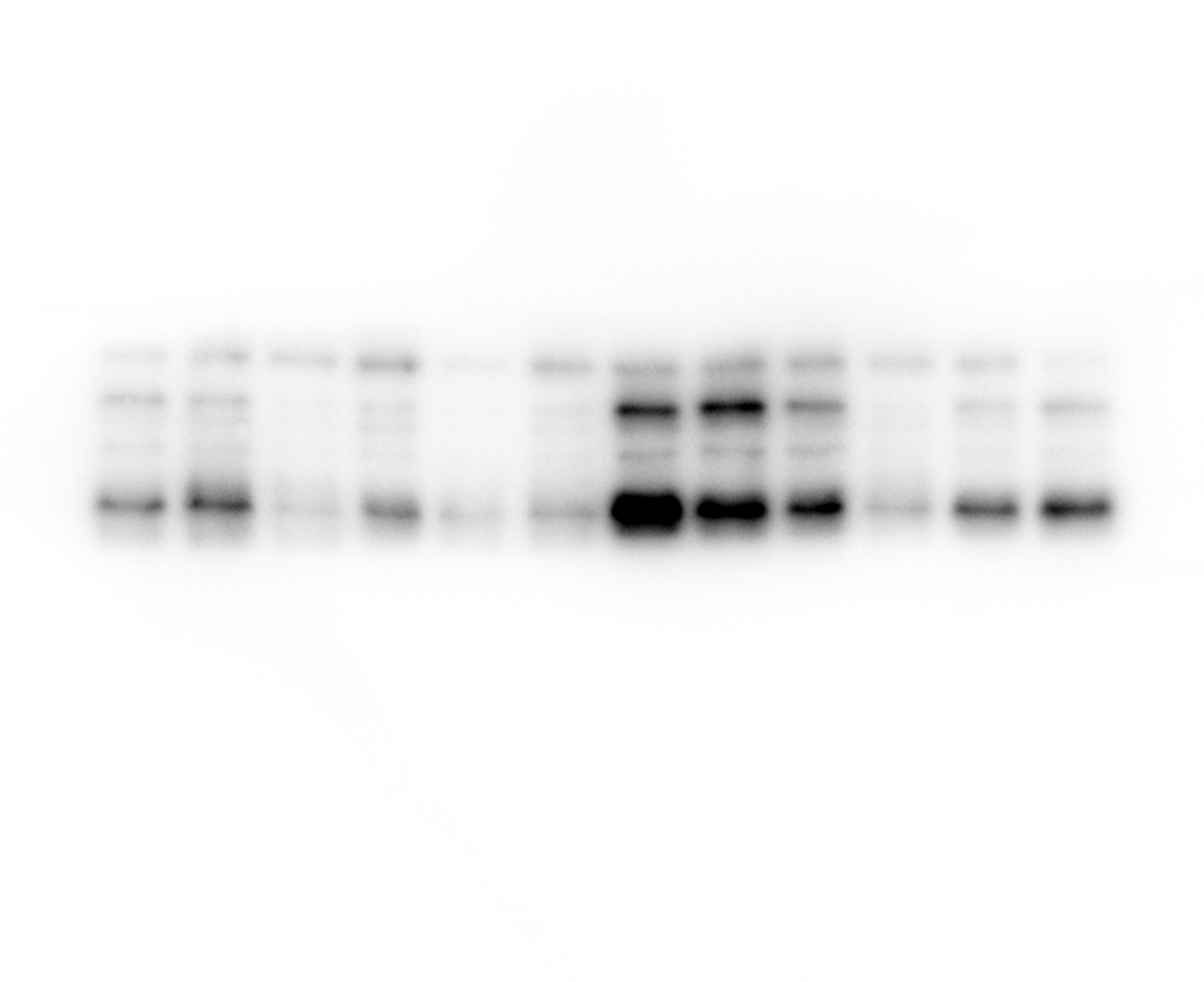

Supplement: Figure 1—source data 1. [file elife-69310-fig1-data1.zip › Figure 1-source data 1/MFF.Tif]

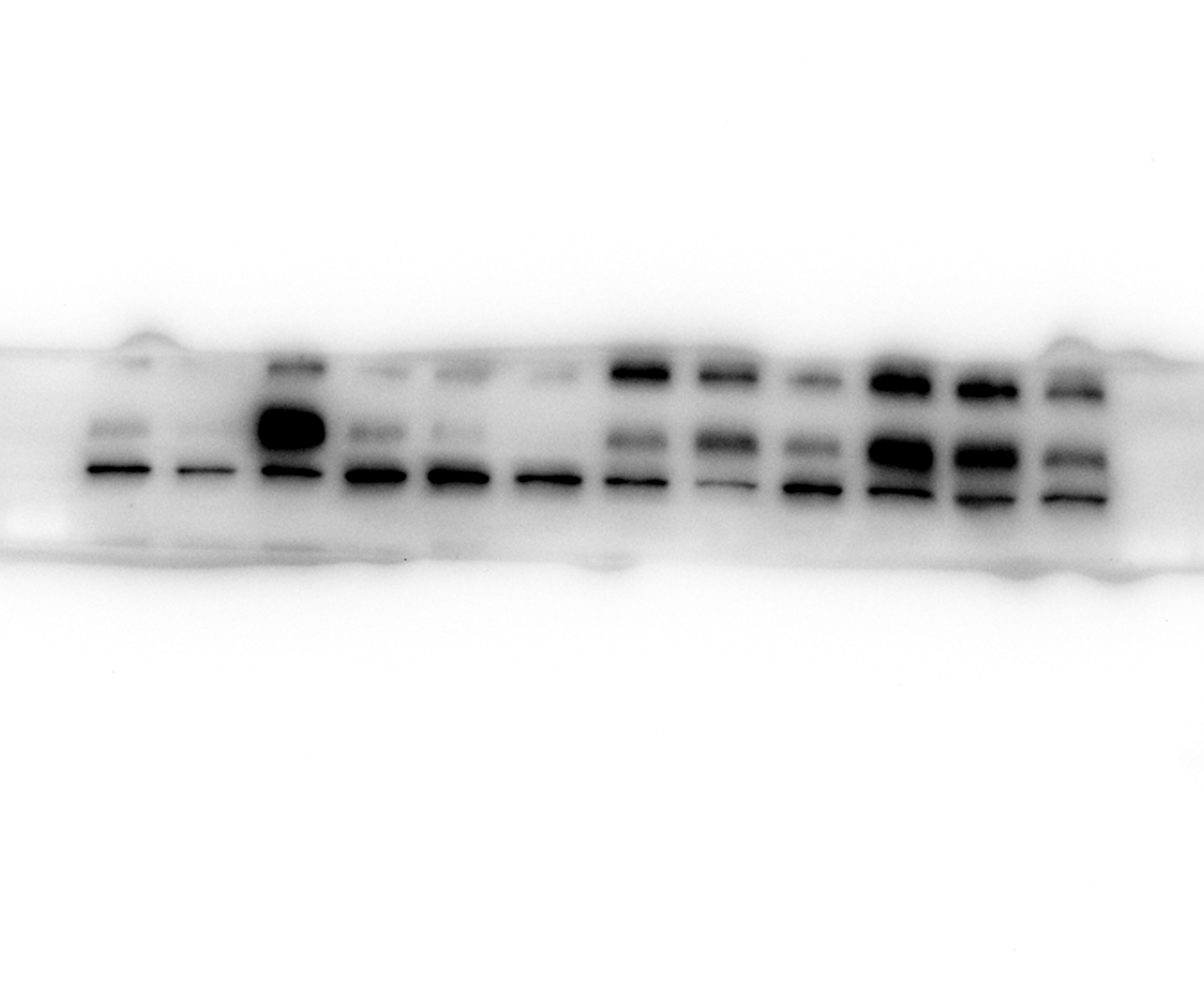

Supplement: Figure 1—source data 1. [file elife-69310-fig1-data1.zip › Figure 1-source data 1/MFN1.Tif]

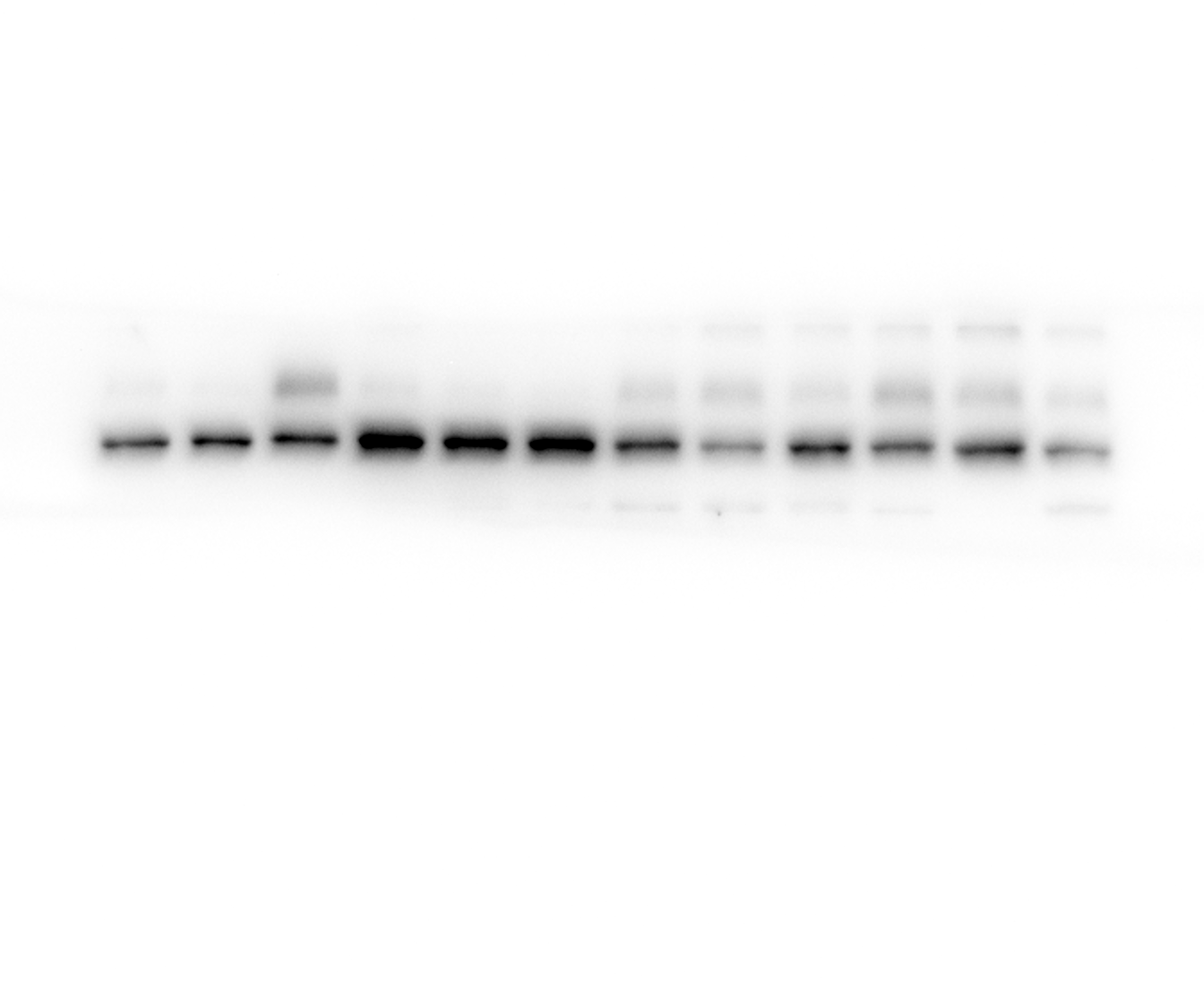

Supplement: Figure 1—source data 1. [file elife-69310-fig1-data1.zip › Figure 1-source data 1/MFN2.Tif]

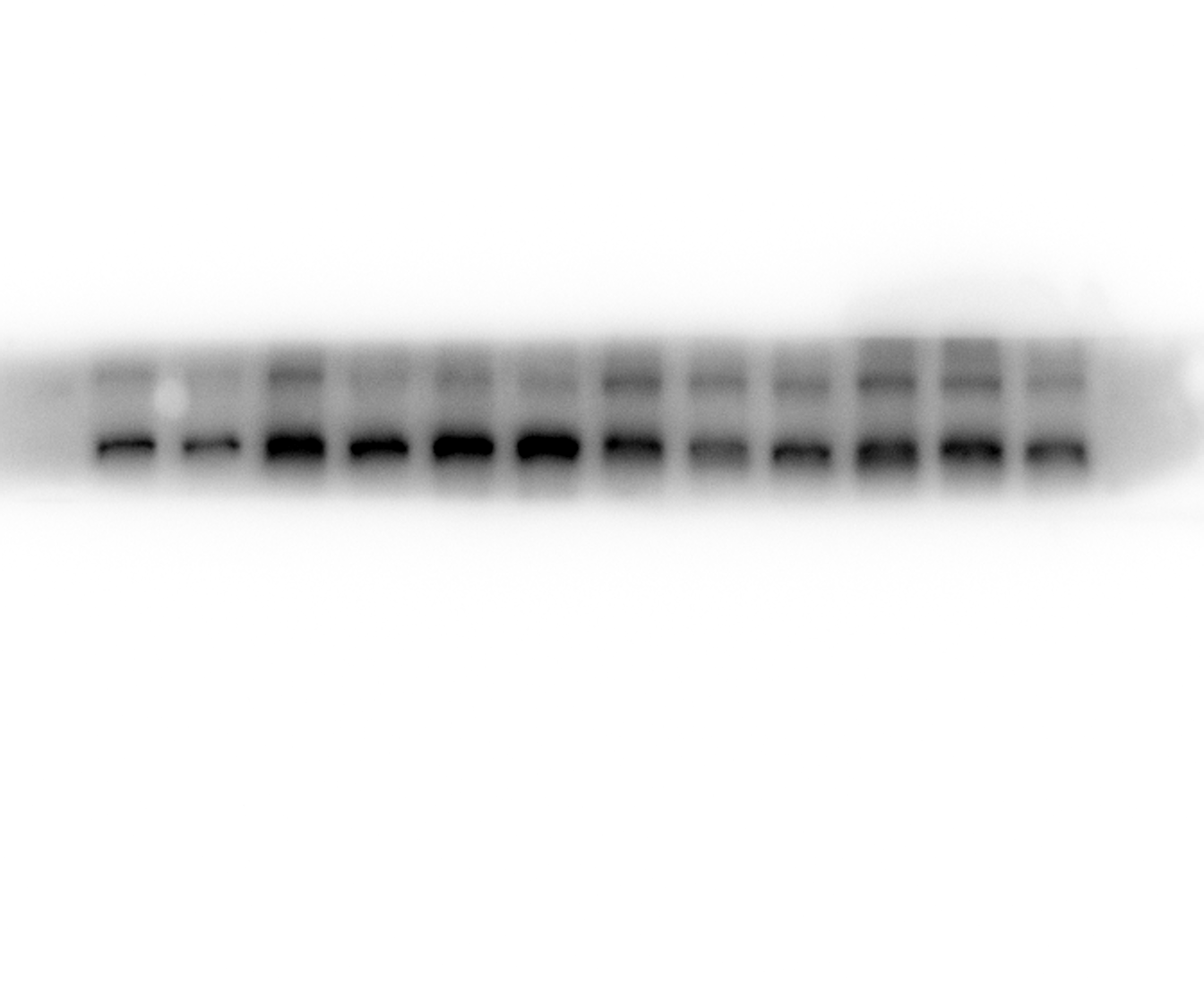

Supplement: Figure 1—source data 1. [file elife-69310-fig1-data1.zip › Figure 1-source data 1/NOTCH1.Tif]

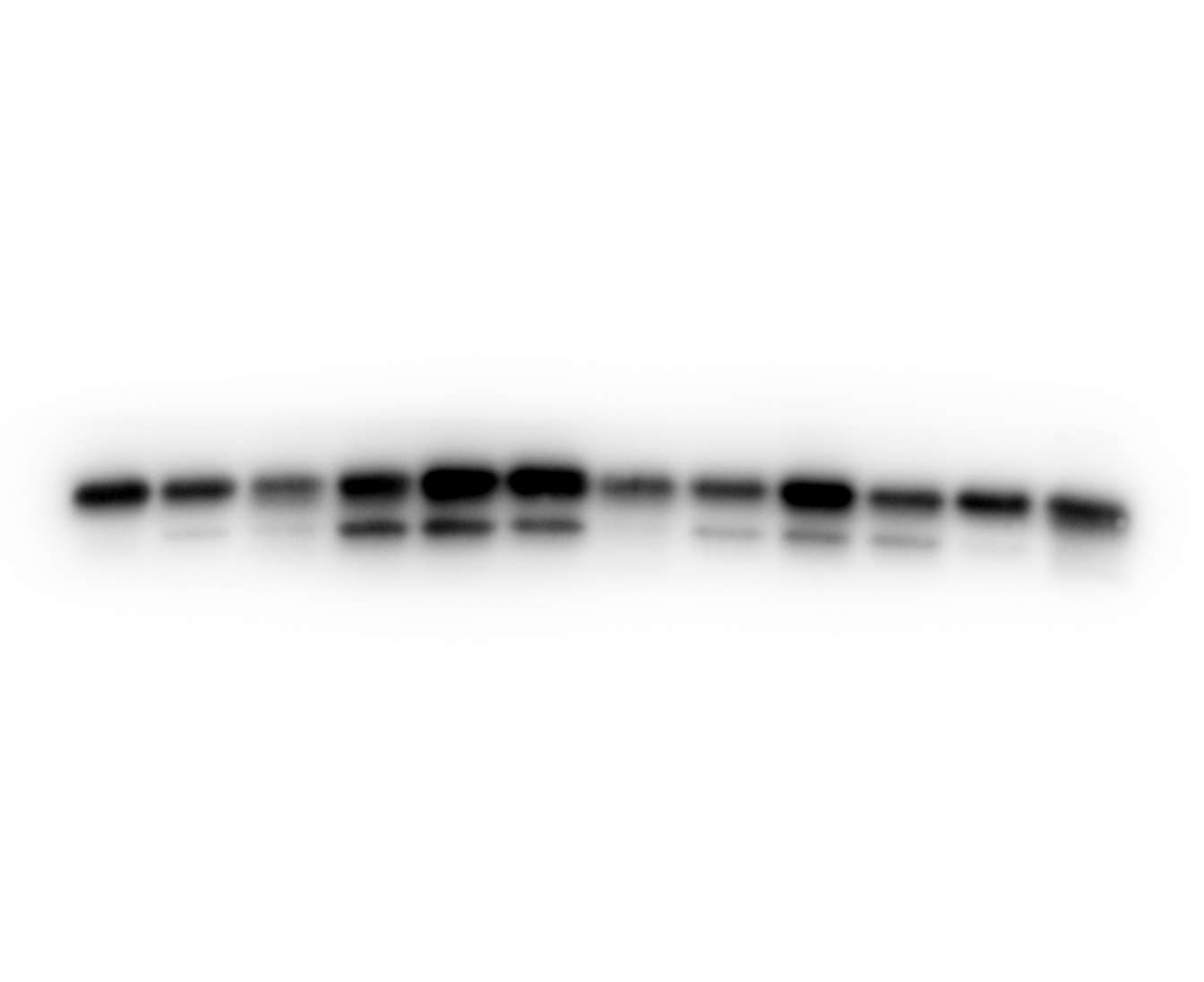

Supplement: Figure 1—source data 1. [file elife-69310-fig1-data1.zip › Figure 1-source data 1/SM22.Tif]

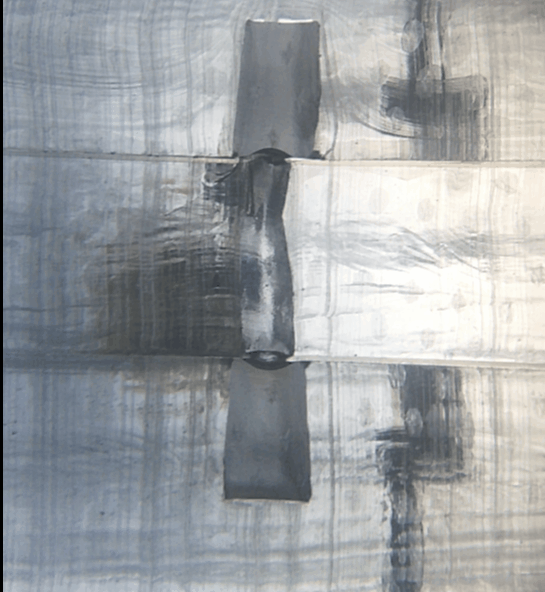

Supplement: Supplementary file 7 [file elife-69310-fig2-video1.gif]

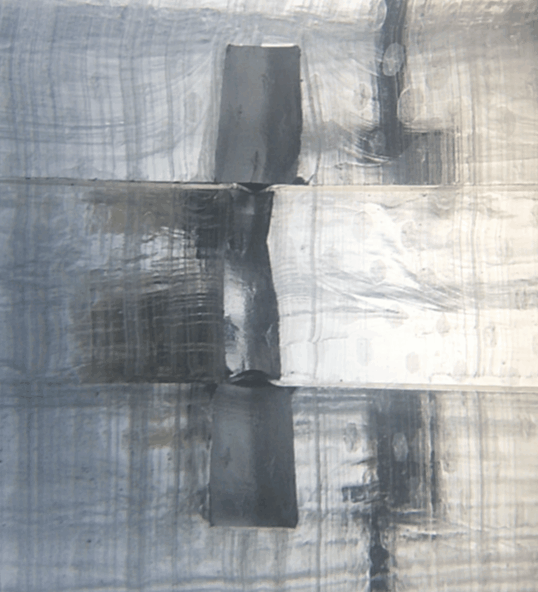

Supplement: Supplementary file 8 [file elife-69310-fig2-video2.gif]

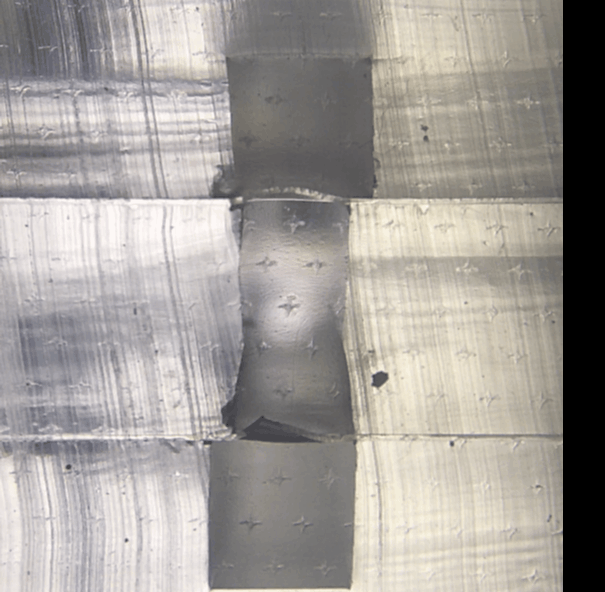

Supplement: Supplementary file 9 [file elife-69310-fig2-video3.gif]

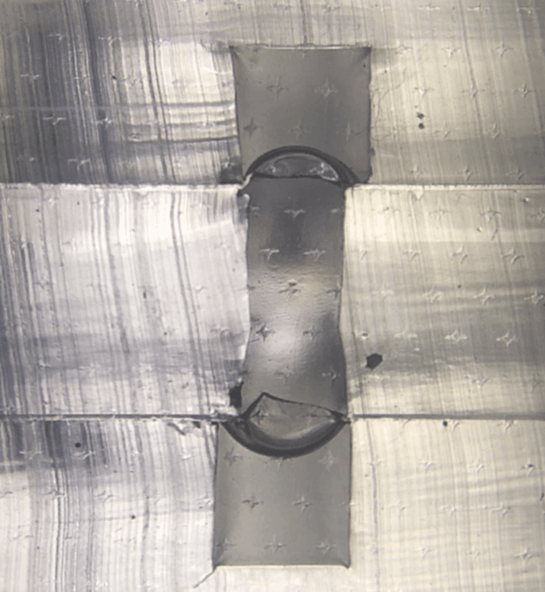

Supplement: Supplementary file 10 [file elife-69310-fig2-video4.gif]

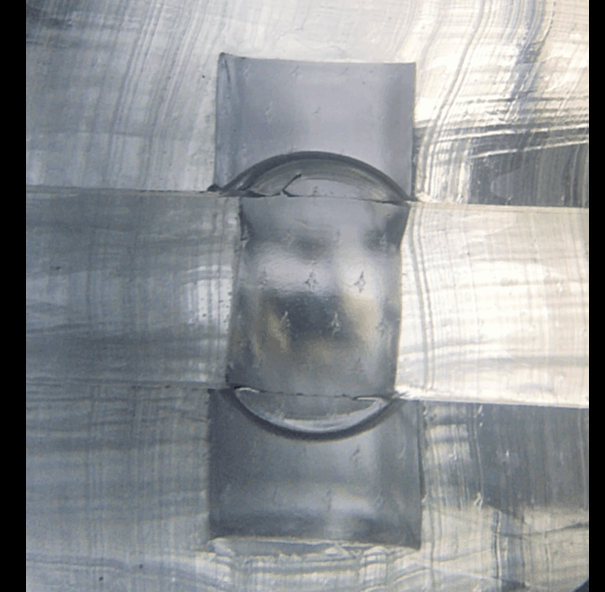

Supplement: Supplementary file 11 [file elife-69310-fig2-video5.gif]

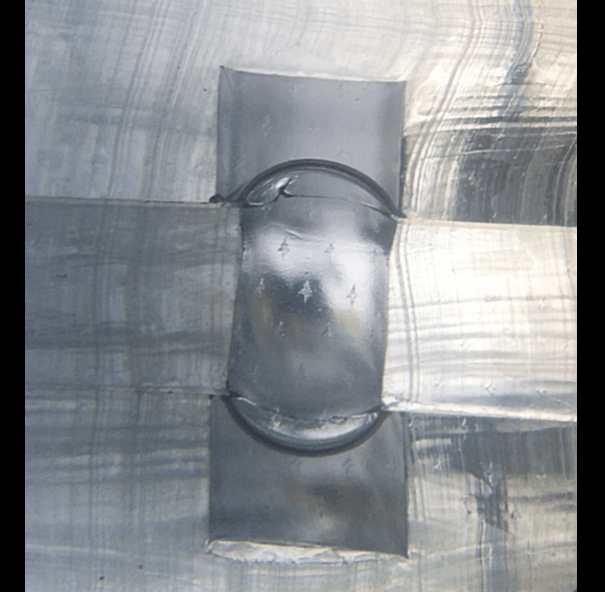

Supplement: Supplementary file 12 [file elife-69310-fig2-video6.gif]

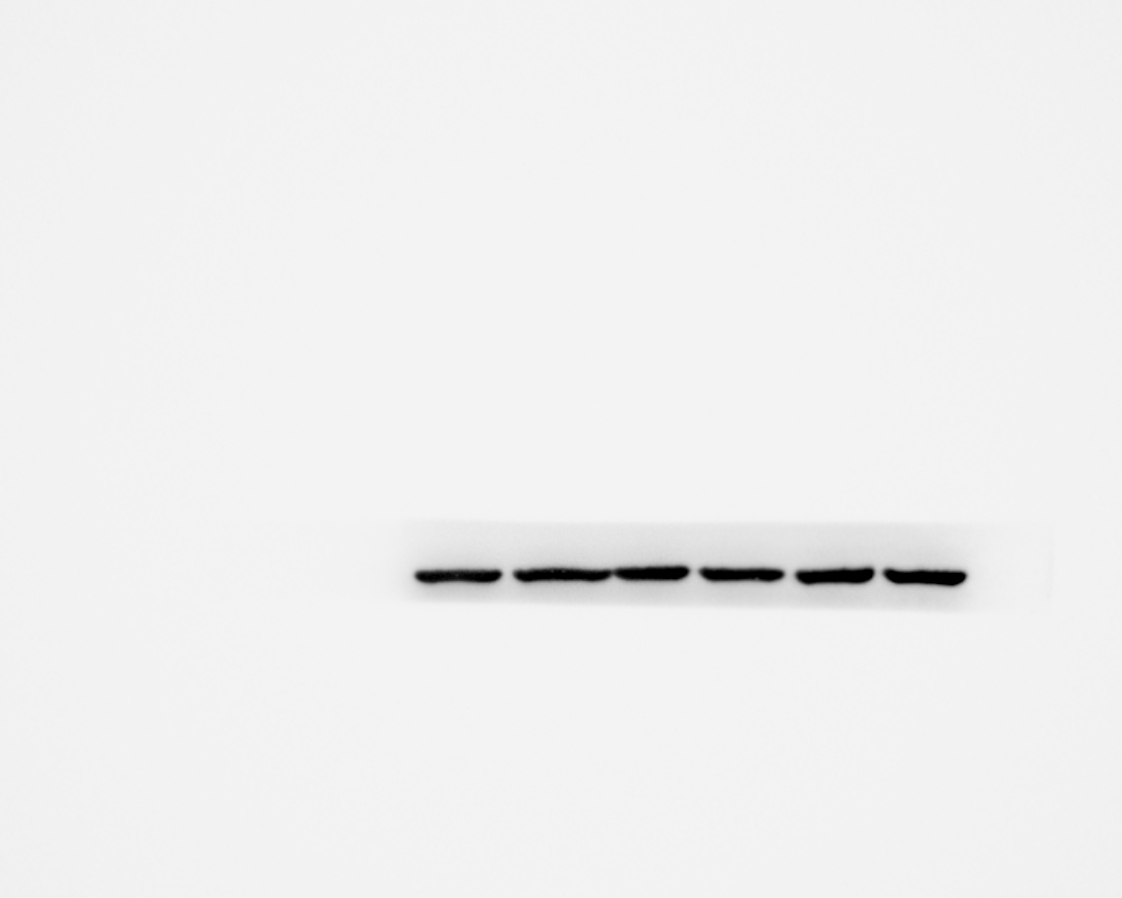

Supplement: Figure 3—source data 3. [file elife-69310-fig3-data3.zip › Figure 3-source data 3/b actin_1.jpg]

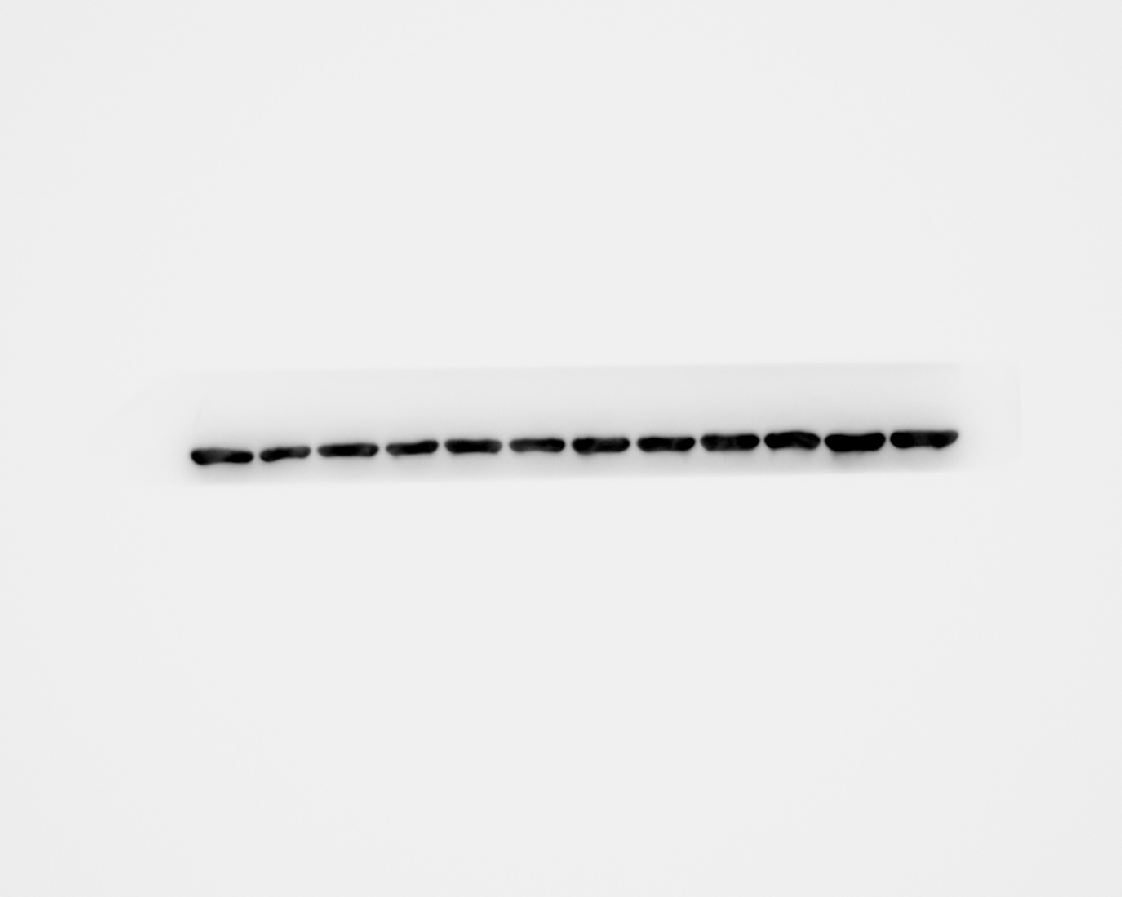

Supplement: Figure 3—source data 3. [file elife-69310-fig3-data3.zip › Figure 3-source data 3/b.actin-1.tif]

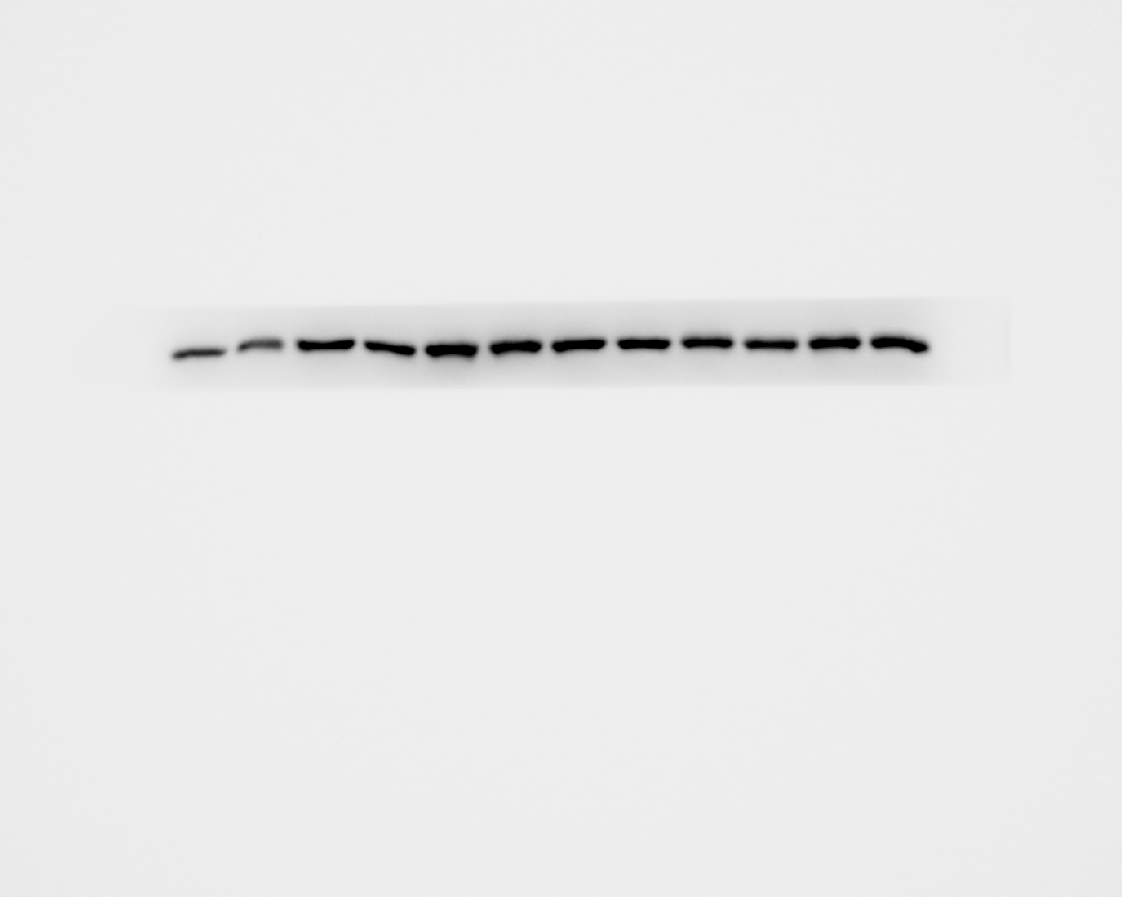

Supplement: Figure 3—source data 3. [file elife-69310-fig3-data3.zip › Figure 3-source data 3/cnn1-2.tif]

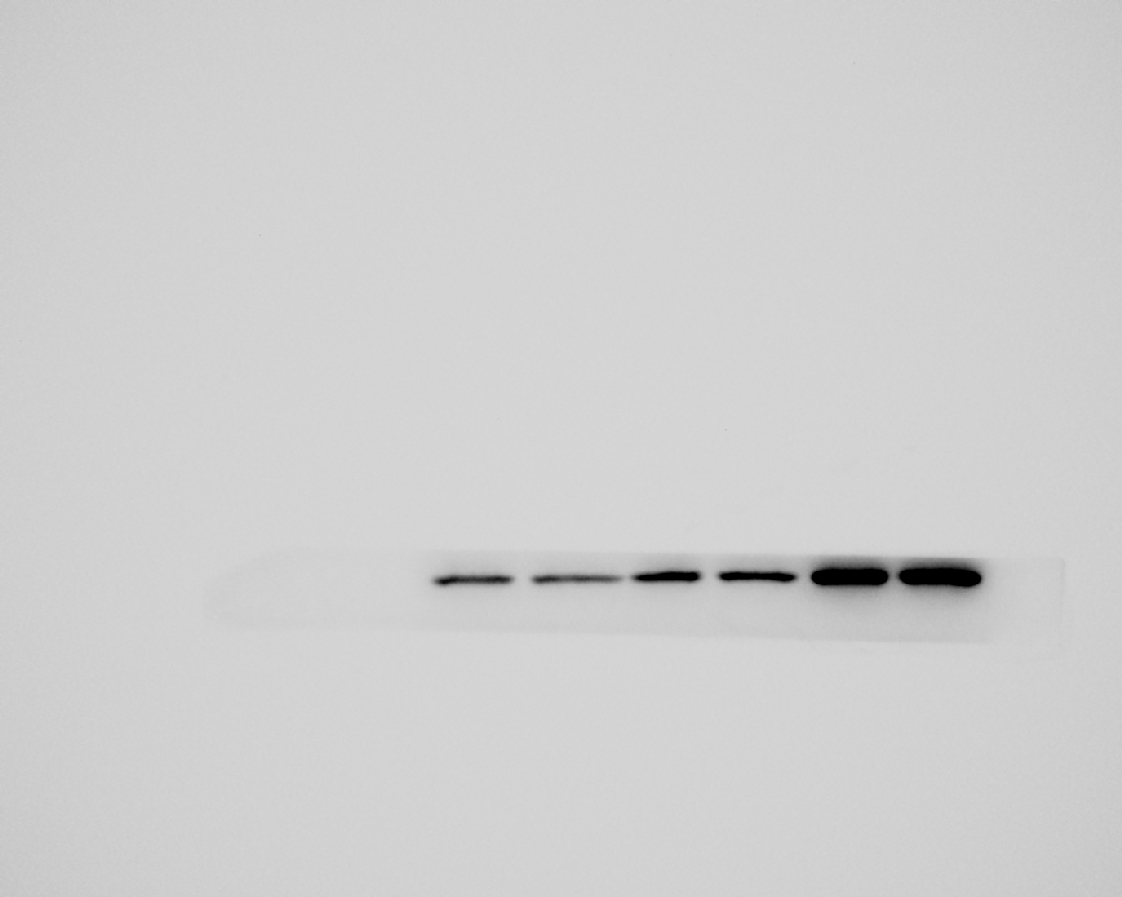

Supplement: Figure 3—source data 3. [file elife-69310-fig3-data3.zip › Figure 3-source data 3/cnn1.jpg]

CNN1

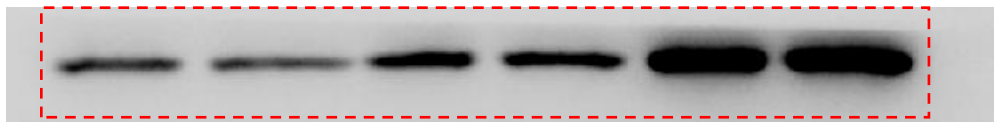

OPN

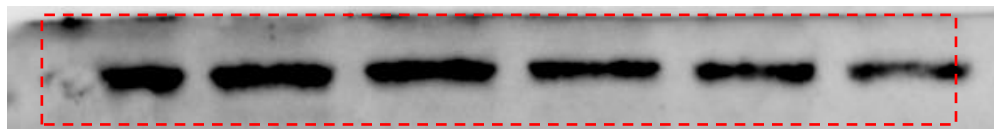

SM22

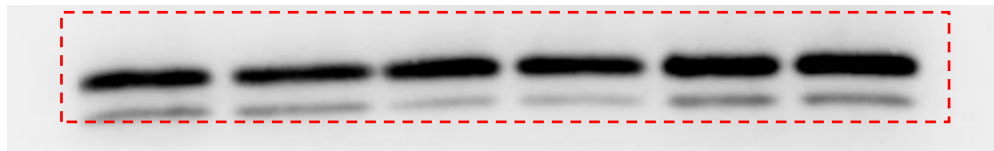

SM22

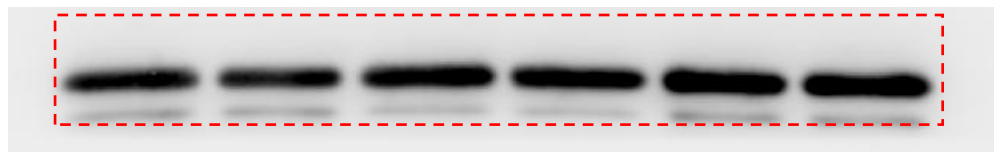

$\beta$ -actin

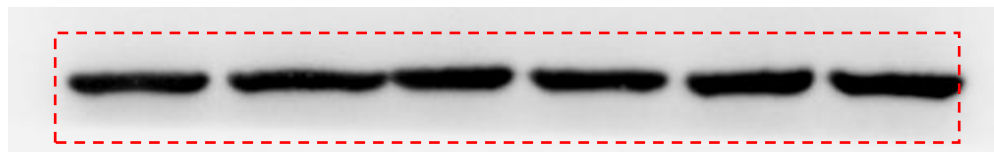

CNN1

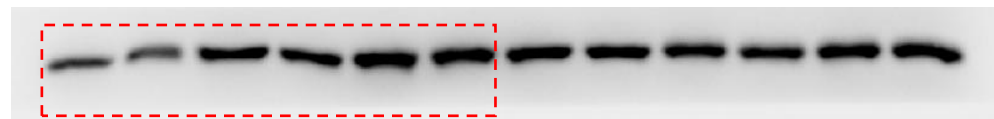

OPN

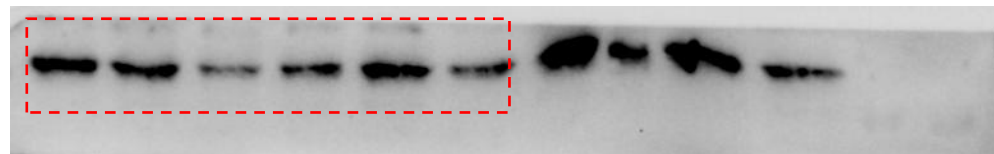

$\beta$ -actin

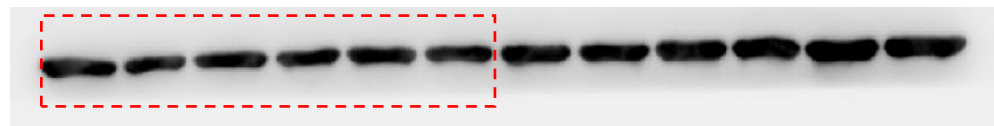

Supplement: Figure 3—source data 3. [file elife-69310-fig3-data3.zip › Figure 3-source data 3/labeled WB .pdf]

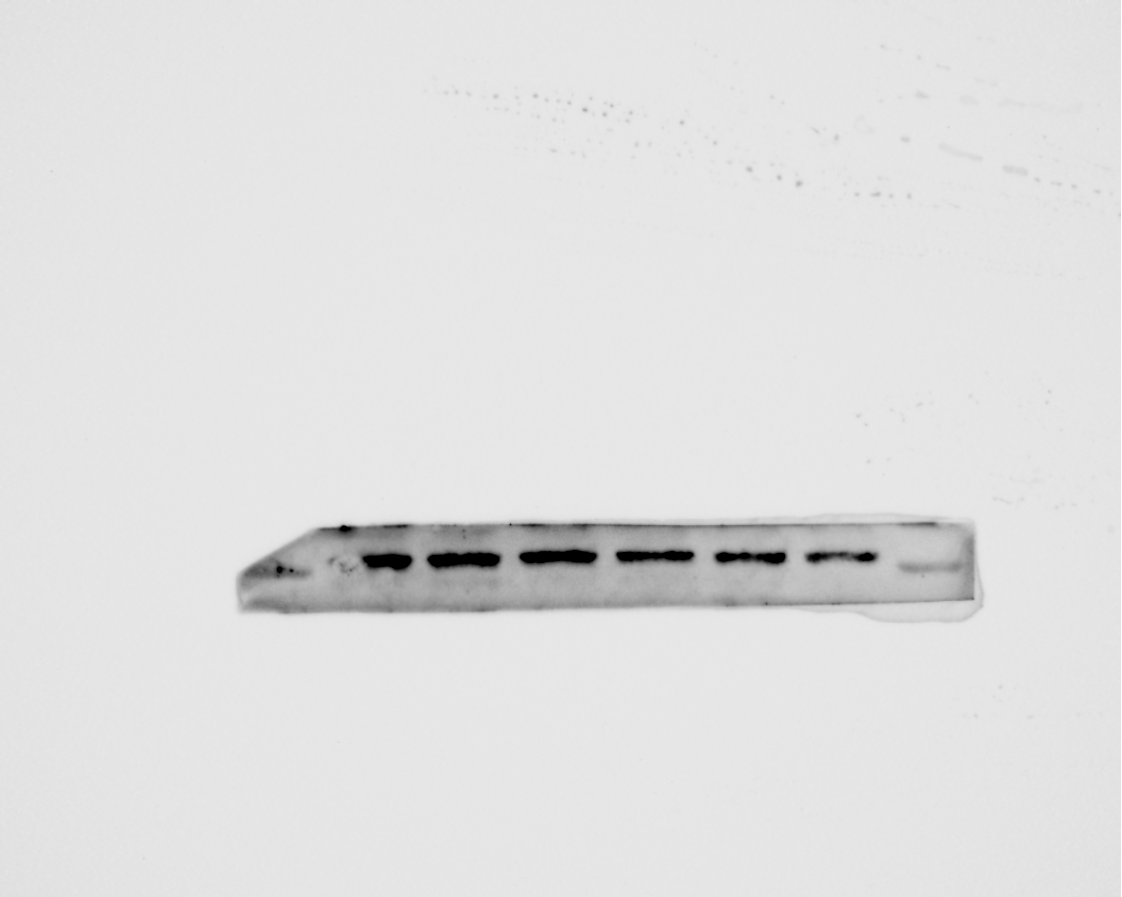

Supplement: Figure 3—source data 3. [file elife-69310-fig3-data3.zip › Figure 3-source data 3/OPN-1.tif]

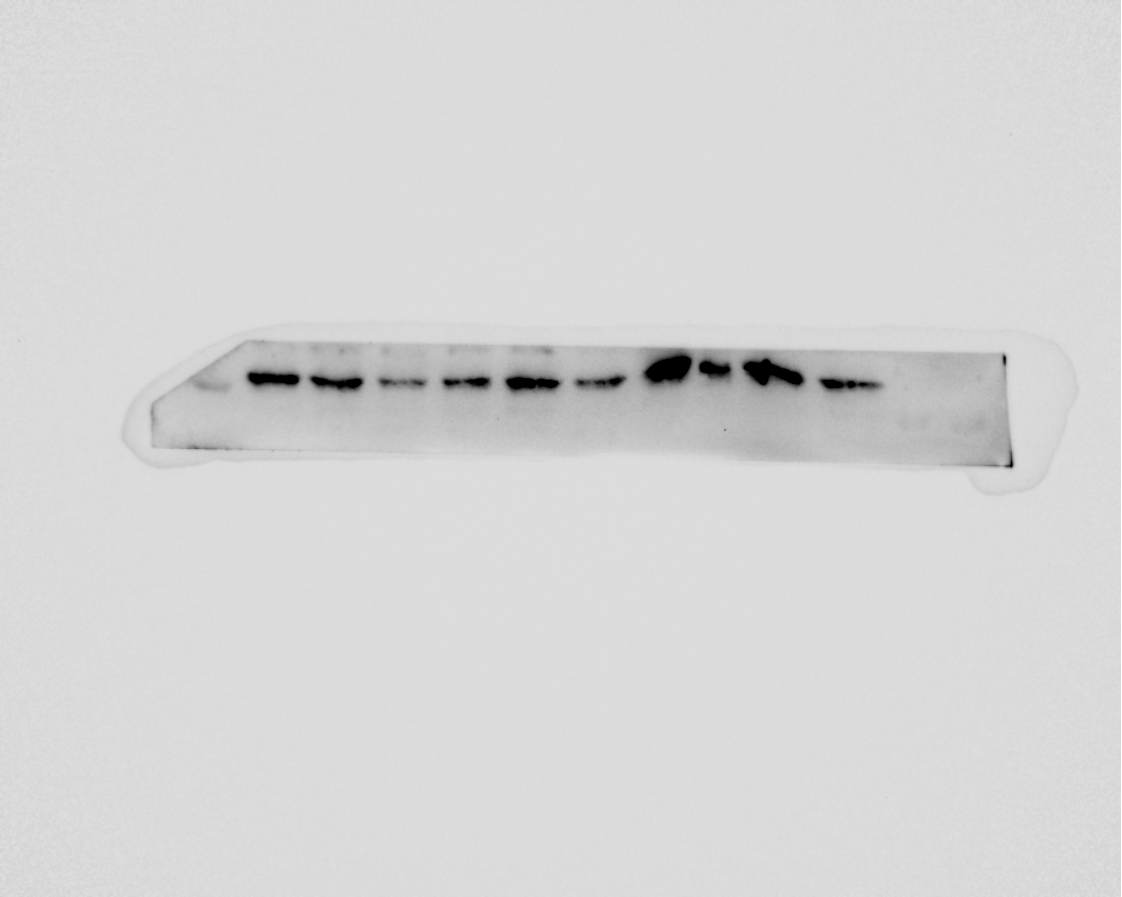

Supplement: Figure 3—source data 3. [file elife-69310-fig3-data3.zip › Figure 3-source data 3/OPN-2.tif]

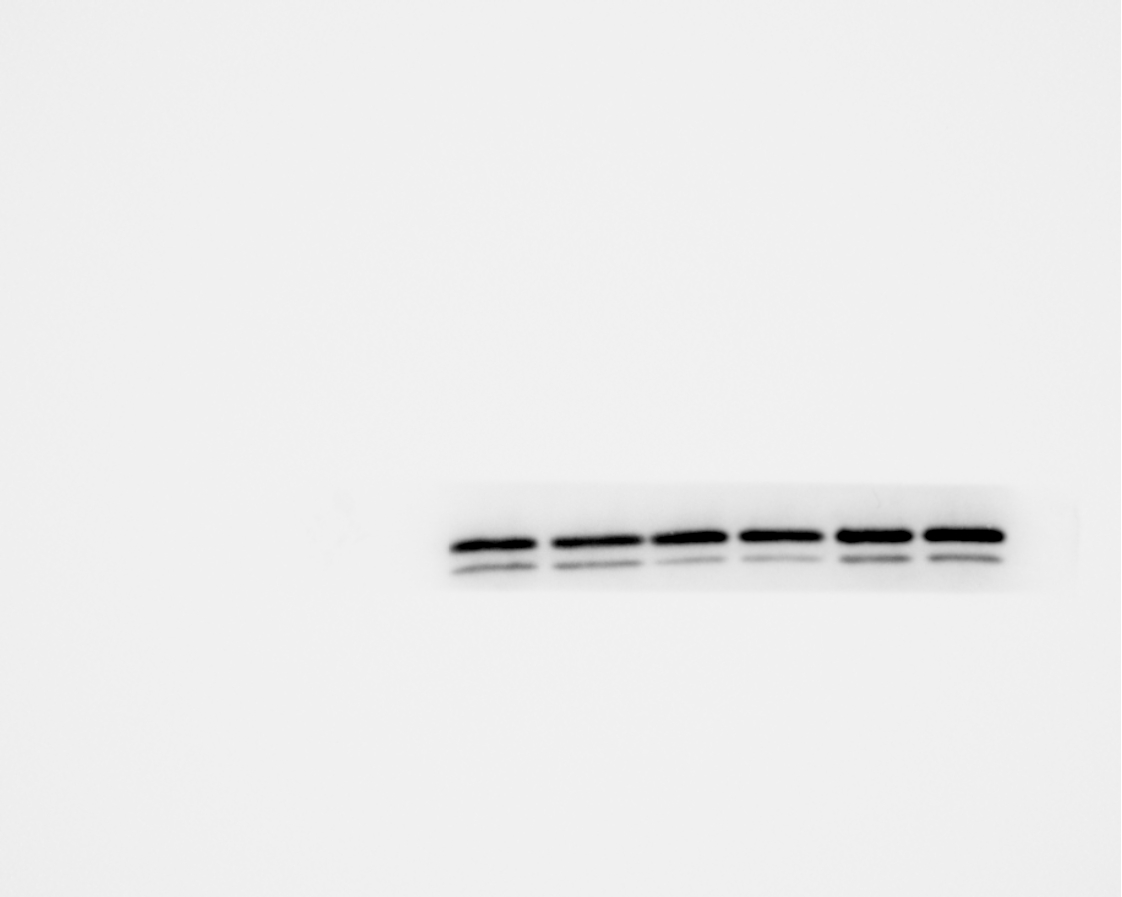

Supplement: Figure 3—source data 3. [file elife-69310-fig3-data3.zip › Figure 3-source data 3/sm22-2.jpg]

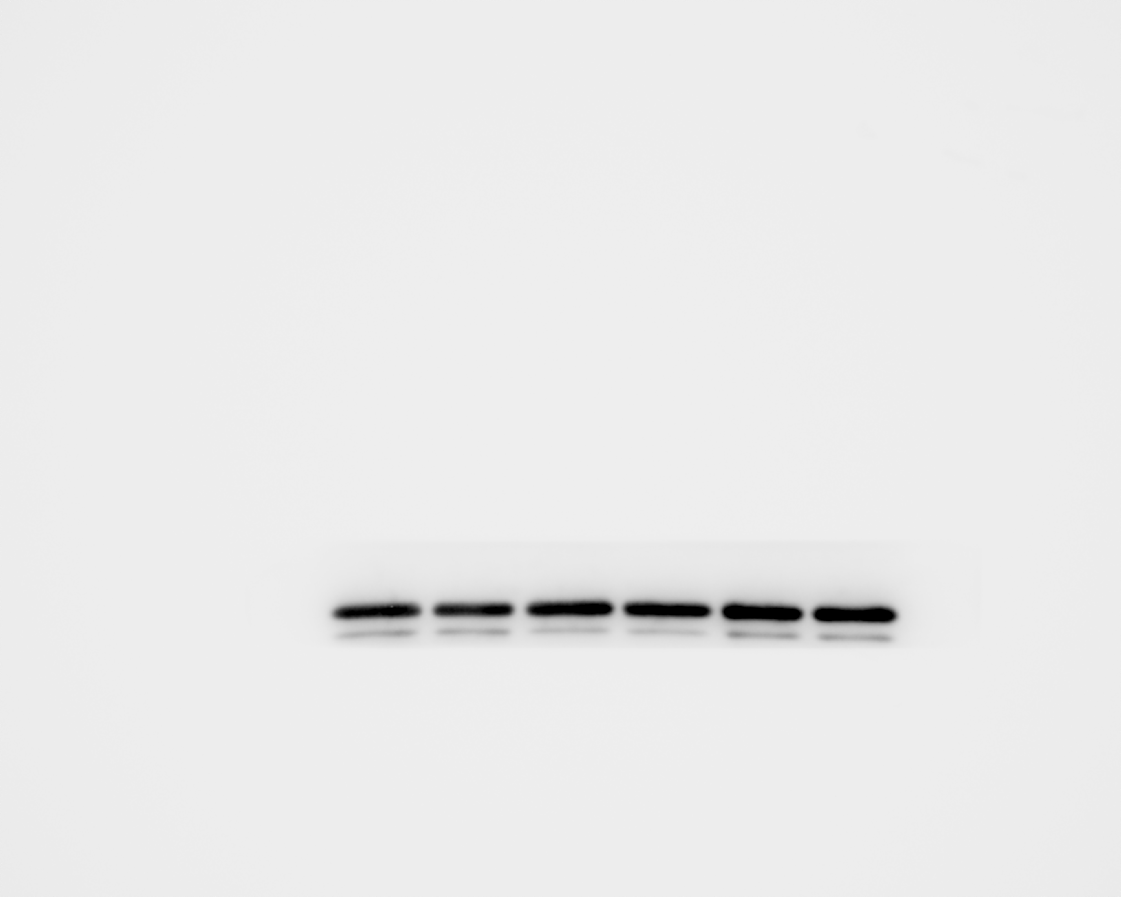

Supplement: Figure 3—source data 3. [file elife-69310-fig3-data3.zip › Figure 3-source data 3/sm22~2_2(Chemiluminescence).tif]

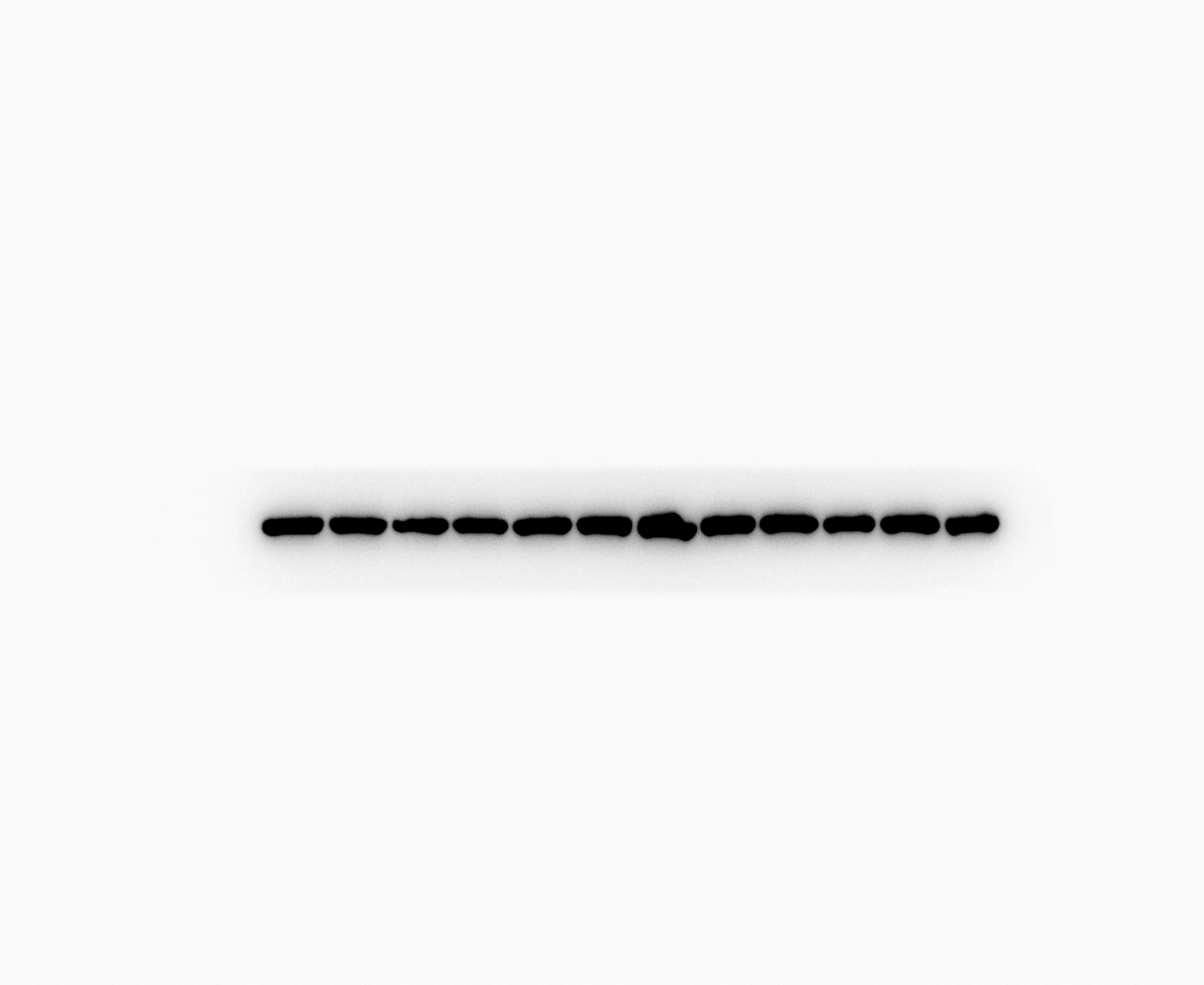

Supplement: Figure 4—source data 2. [file elife-69310-fig4-data2.zip › Figure 4-source data 2/b-actin-2.tif]

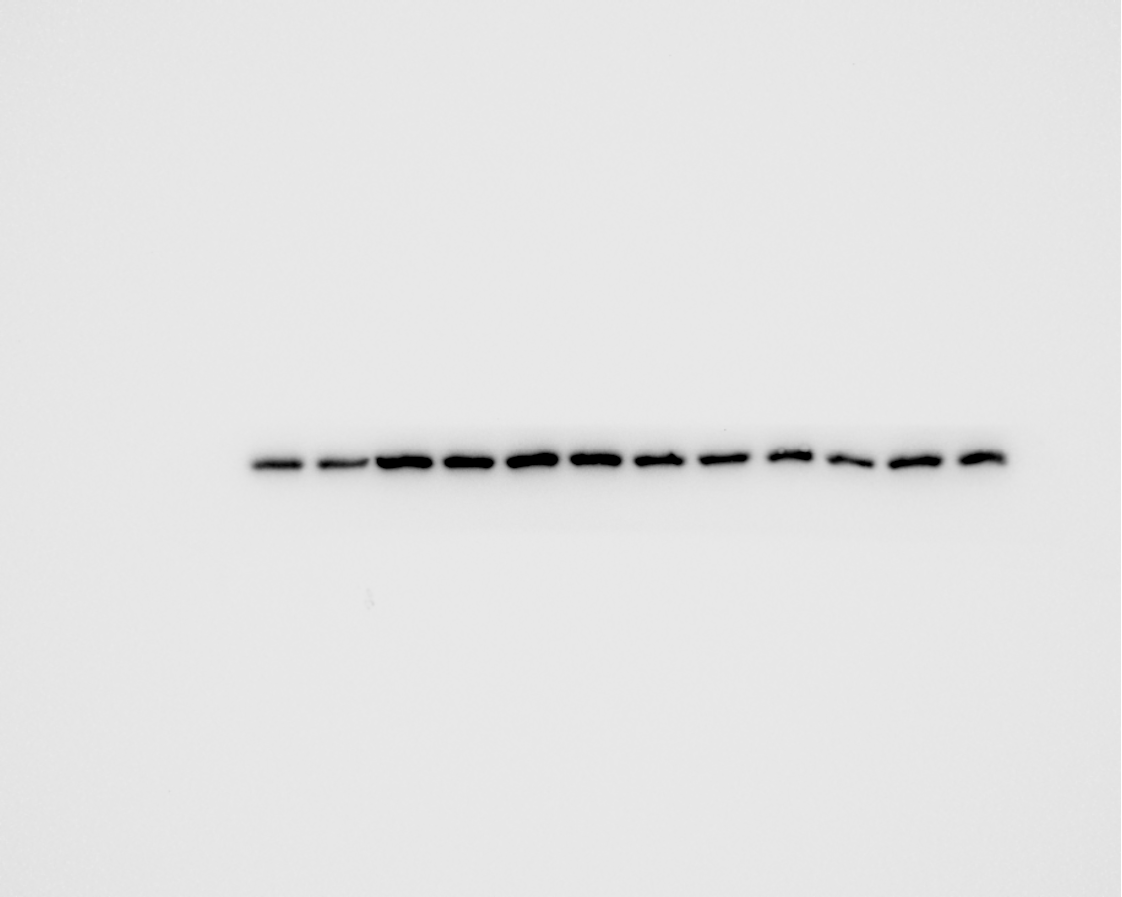

Supplement: Figure 4—source data 2. [file elife-69310-fig4-data2.zip › Figure 4-source data 2/cnn1-_1(Chemiluminescence).tif]

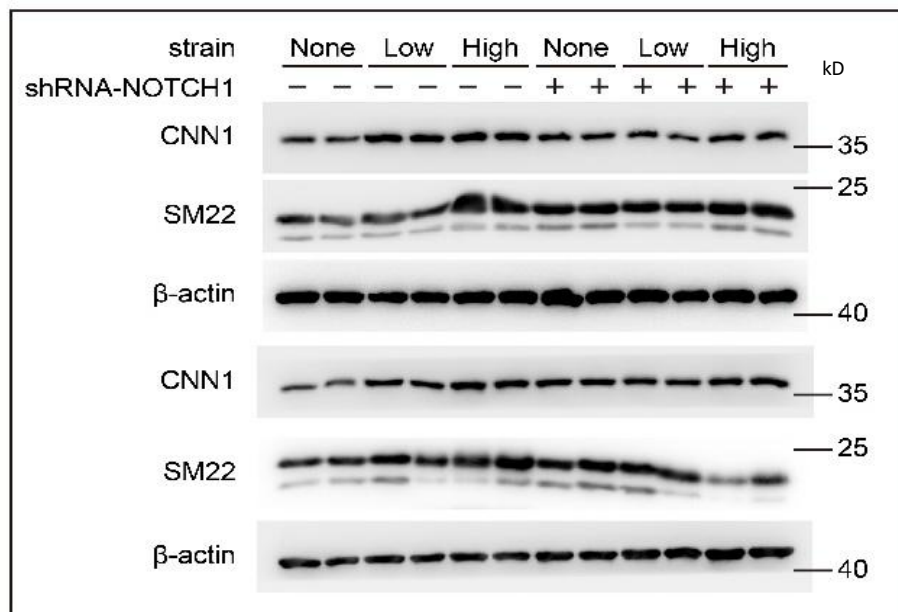

Supplement: Figure 4—source data 2. [file elife-69310-fig4-data2.zip › Figure 4-source data 2/labeled uncropped WB .pdf]

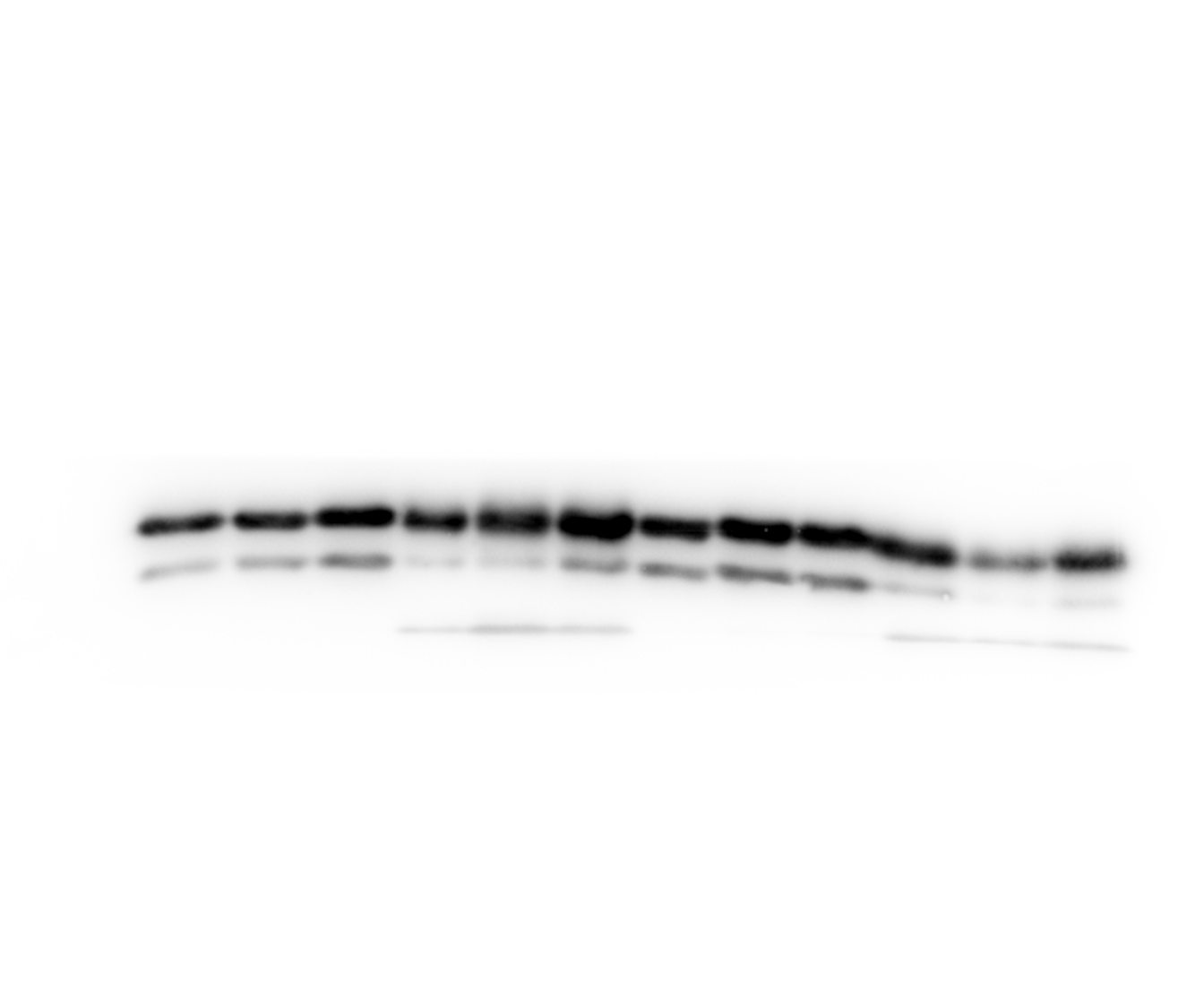

Supplement: Figure 4—source data 2. [file elife-69310-fig4-data2.zip › Figure 4-source data 2/SM22-001-3.Tif]

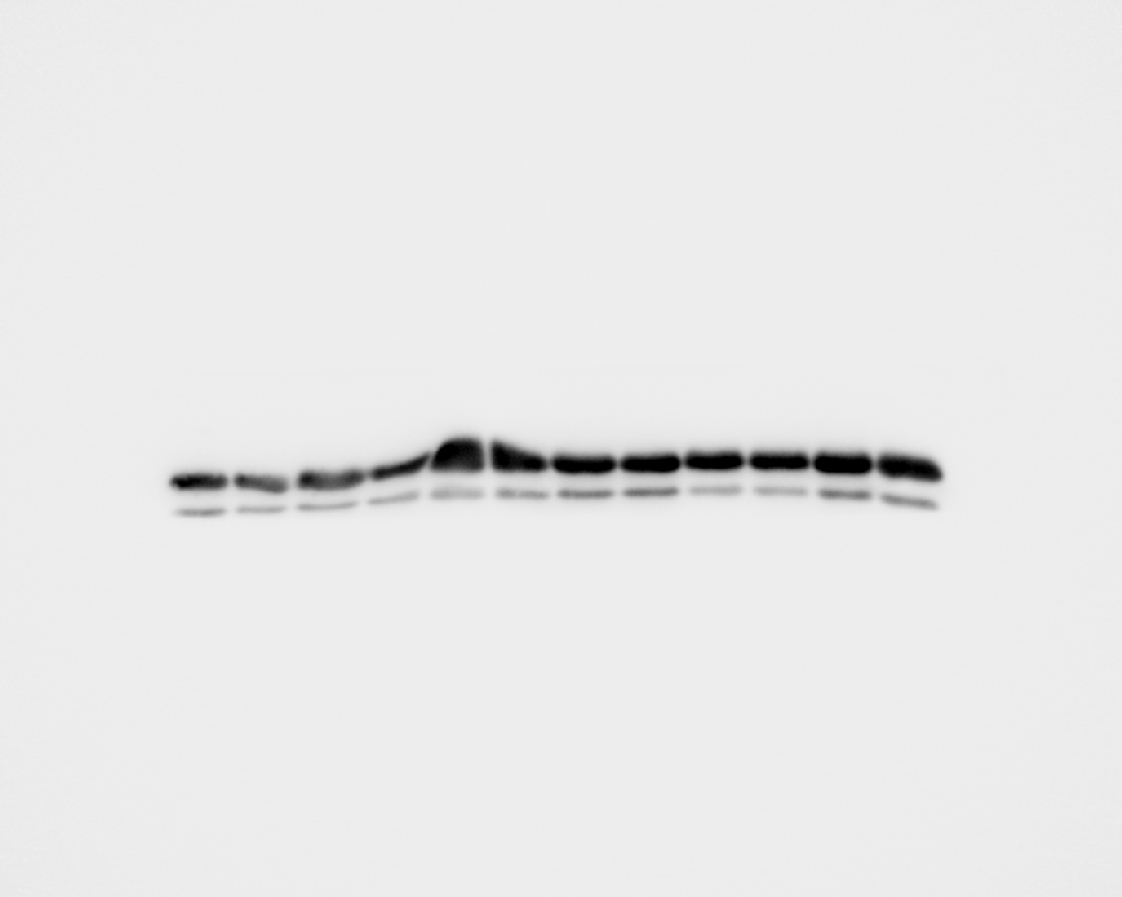

Supplement: Figure 4—source data 2. [file elife-69310-fig4-data2.zip › Figure 4-source data 2/sm22-1_2(Chemiluminescence).jpg]

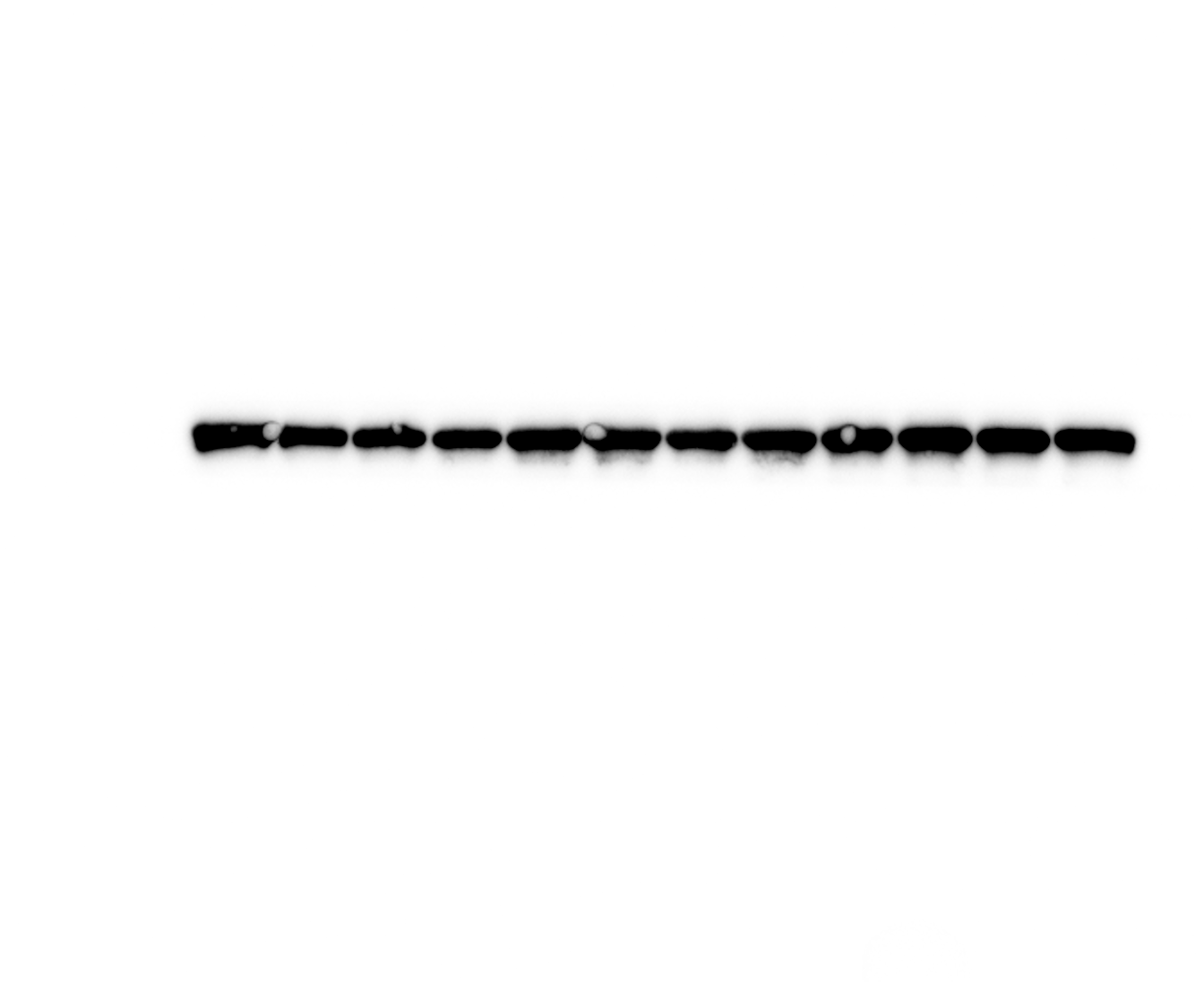

Supplement: Figure 4—figure supplement 1—source data 1. [file elife-69310-fig4-figsupp1-data1.zip › Figure 4-figure supplement 1-source data 1/B-ACTIN-001-3.Tif]

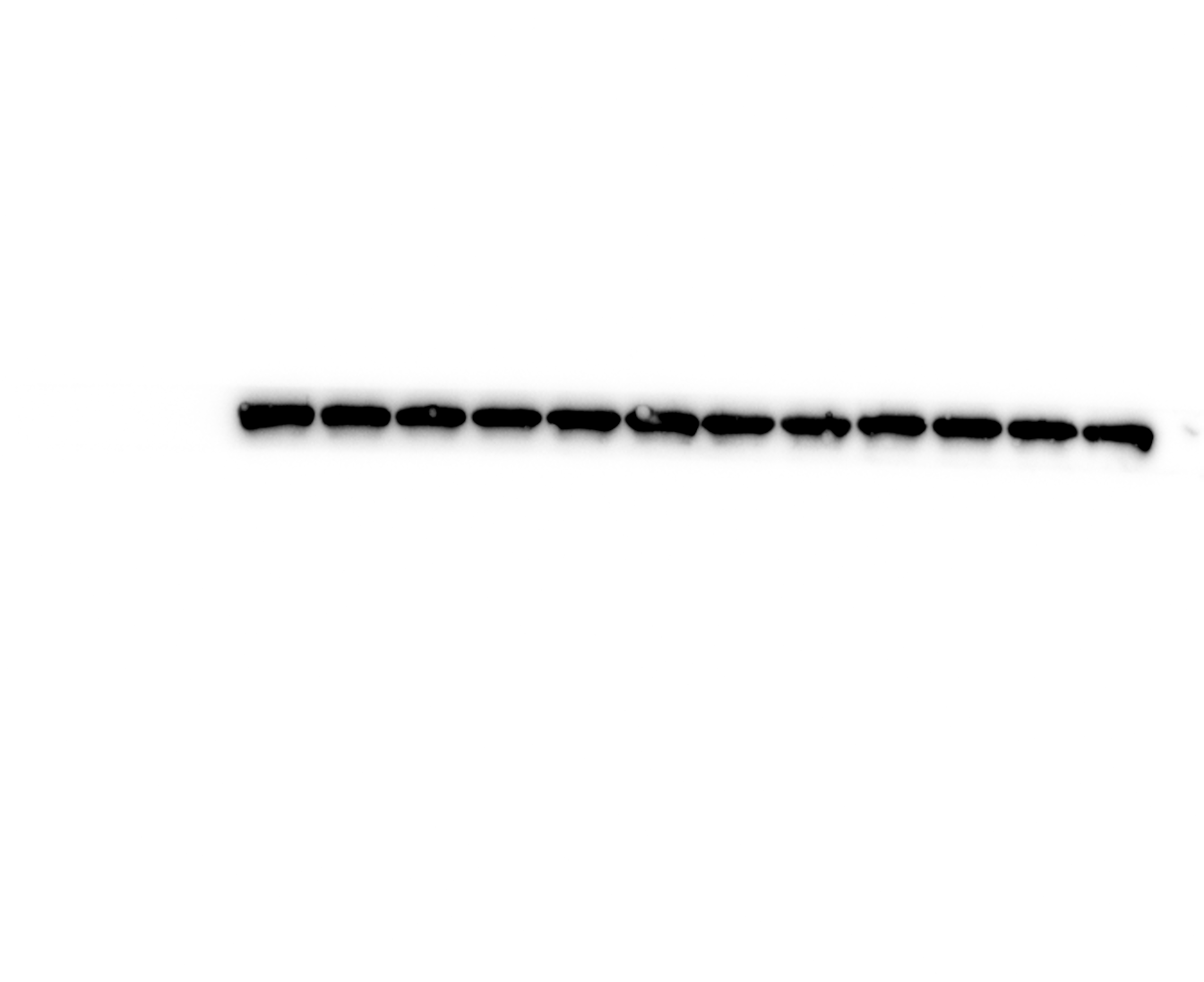

Supplement: Figure 4—figure supplement 1—source data 1. [file elife-69310-fig4-figsupp1-data1.zip › Figure 4-figure supplement 1-source data 1/B-ACTIN-002-3.Tif]

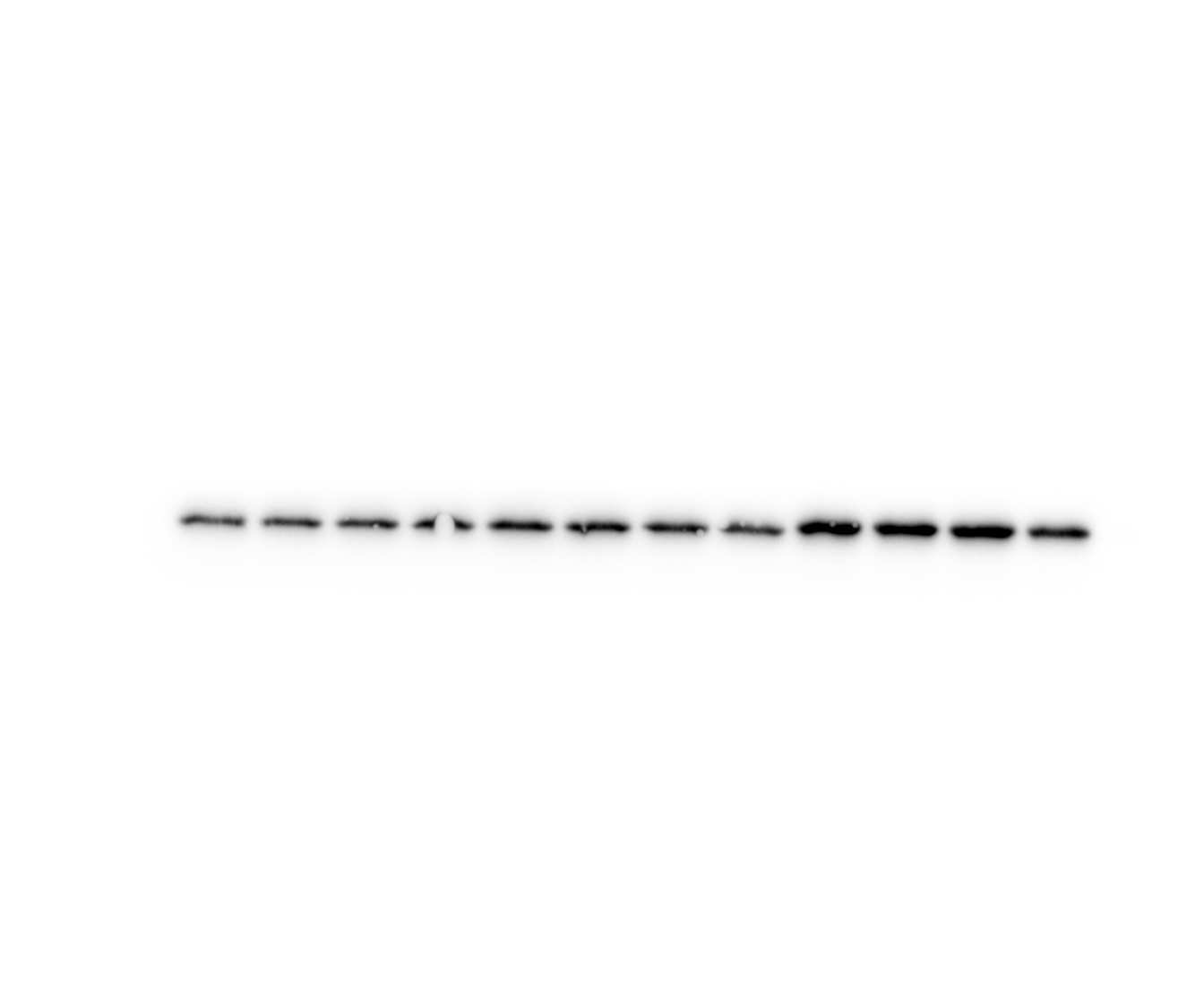

Supplement: Figure 4—figure supplement 1—source data 1. [file elife-69310-fig4-figsupp1-data1.zip › Figure 4-figure supplement 1-source data 1/CNN1-002-3.Tif]

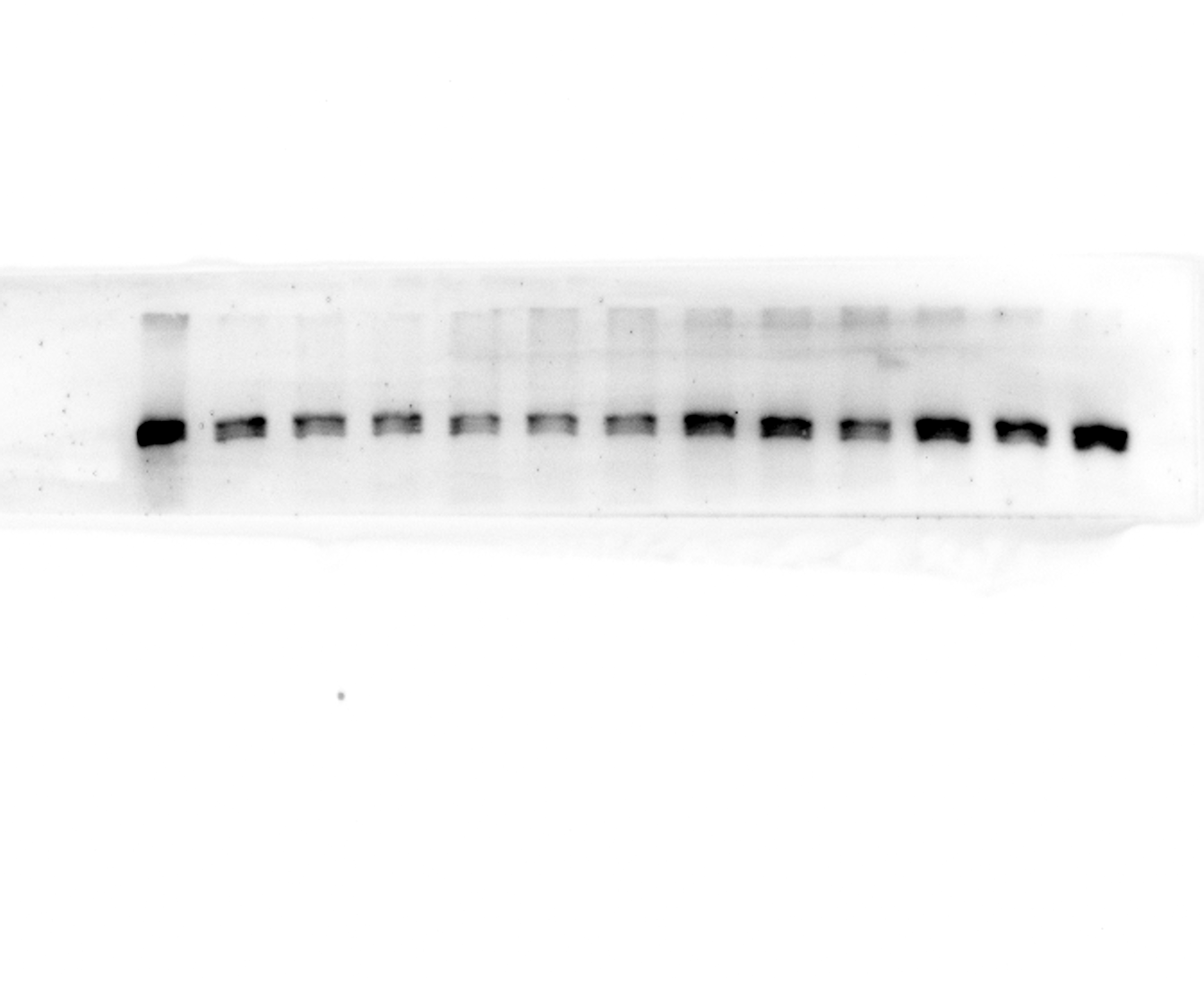

Supplement: Figure 4—figure supplement 1—source data 1. [file elife-69310-fig4-figsupp1-data1.zip › Figure 4-figure supplement 1-source data 1/DRP-1-002-1.Tif]

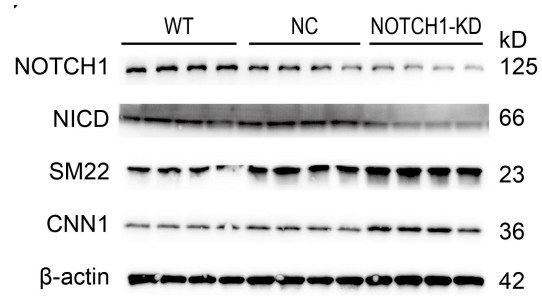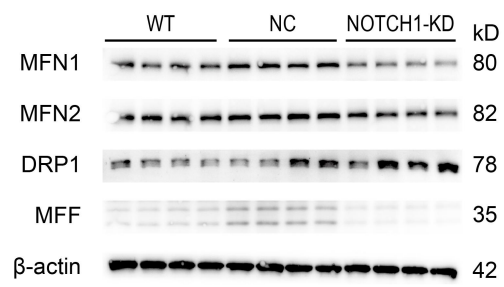

Supplement: Figure 4—figure supplement 1—source data 1. [file elife-69310-fig4-figsupp1-data1.zip › Figure 4-figure supplement 1-source data 1/labeled uncropped WB .pdf]

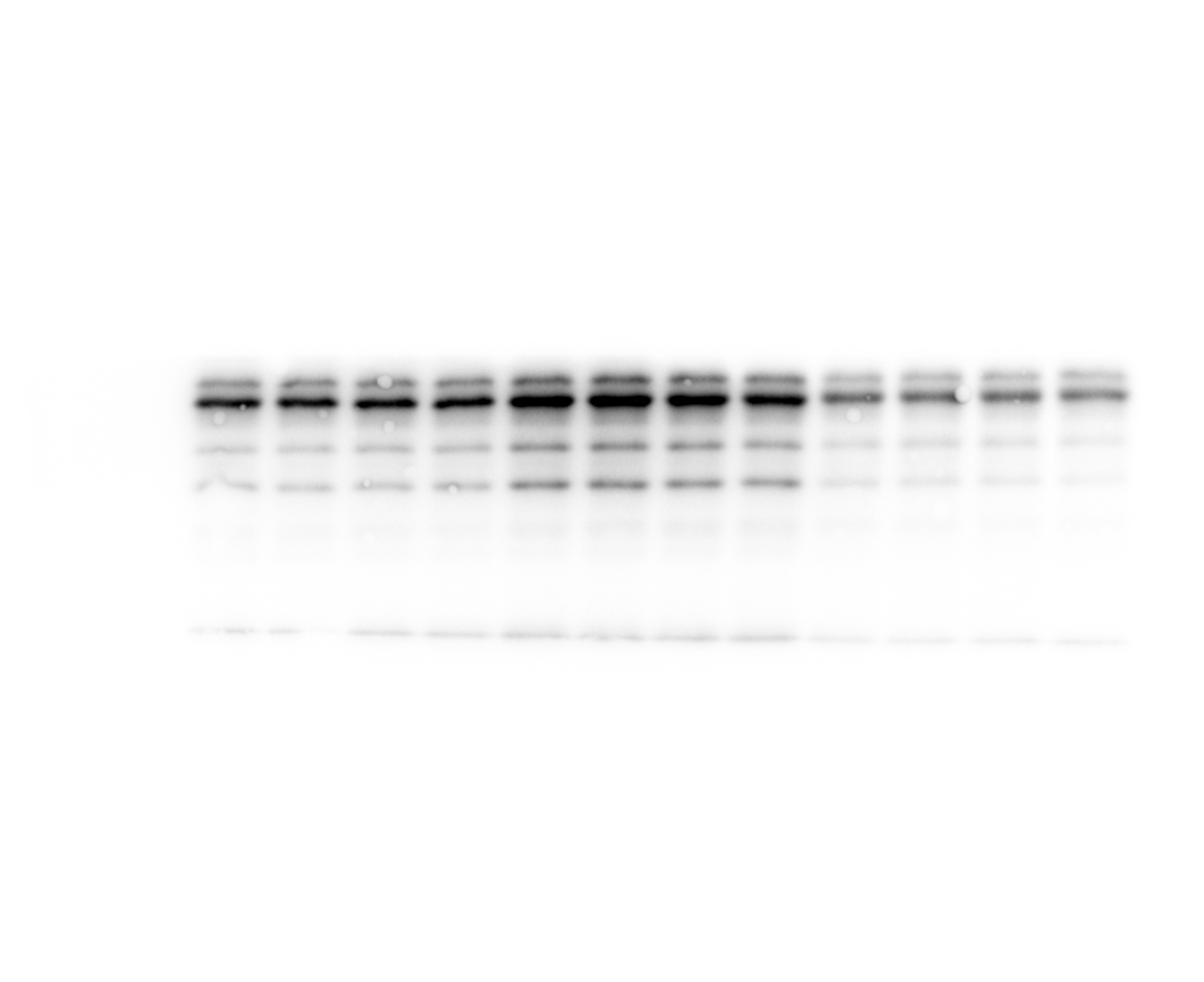

Supplement: Figure 4—figure supplement 1—source data 1. [file elife-69310-fig4-figsupp1-data1.zip › Figure 4-figure supplement 1-source data 1/MFF-001-3.Tif]

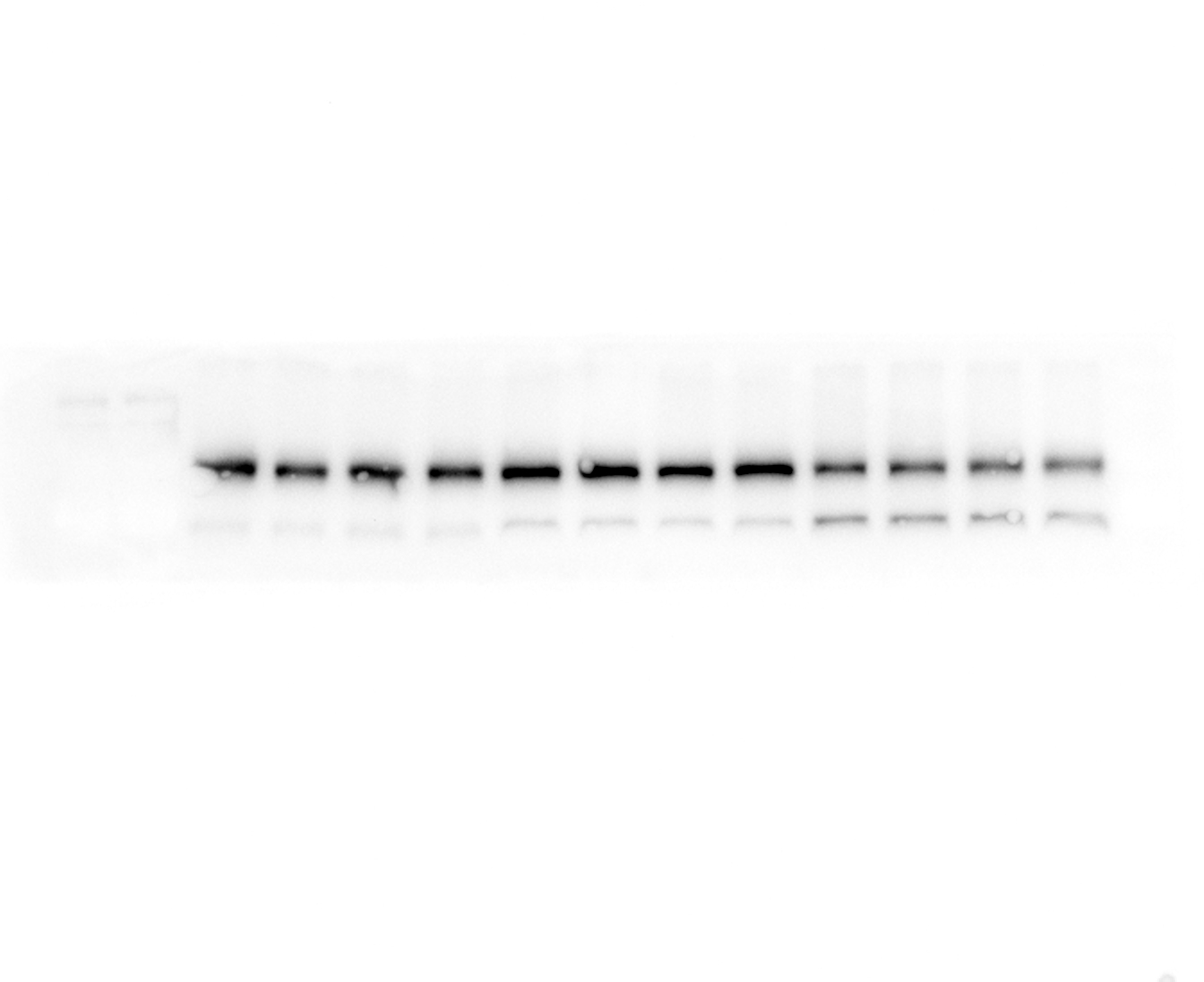

Supplement: Figure 4—figure supplement 1—source data 1. [file elife-69310-fig4-figsupp1-data1.zip › Figure 4-figure supplement 1-source data 1/MFN-1-001-3.Tif]

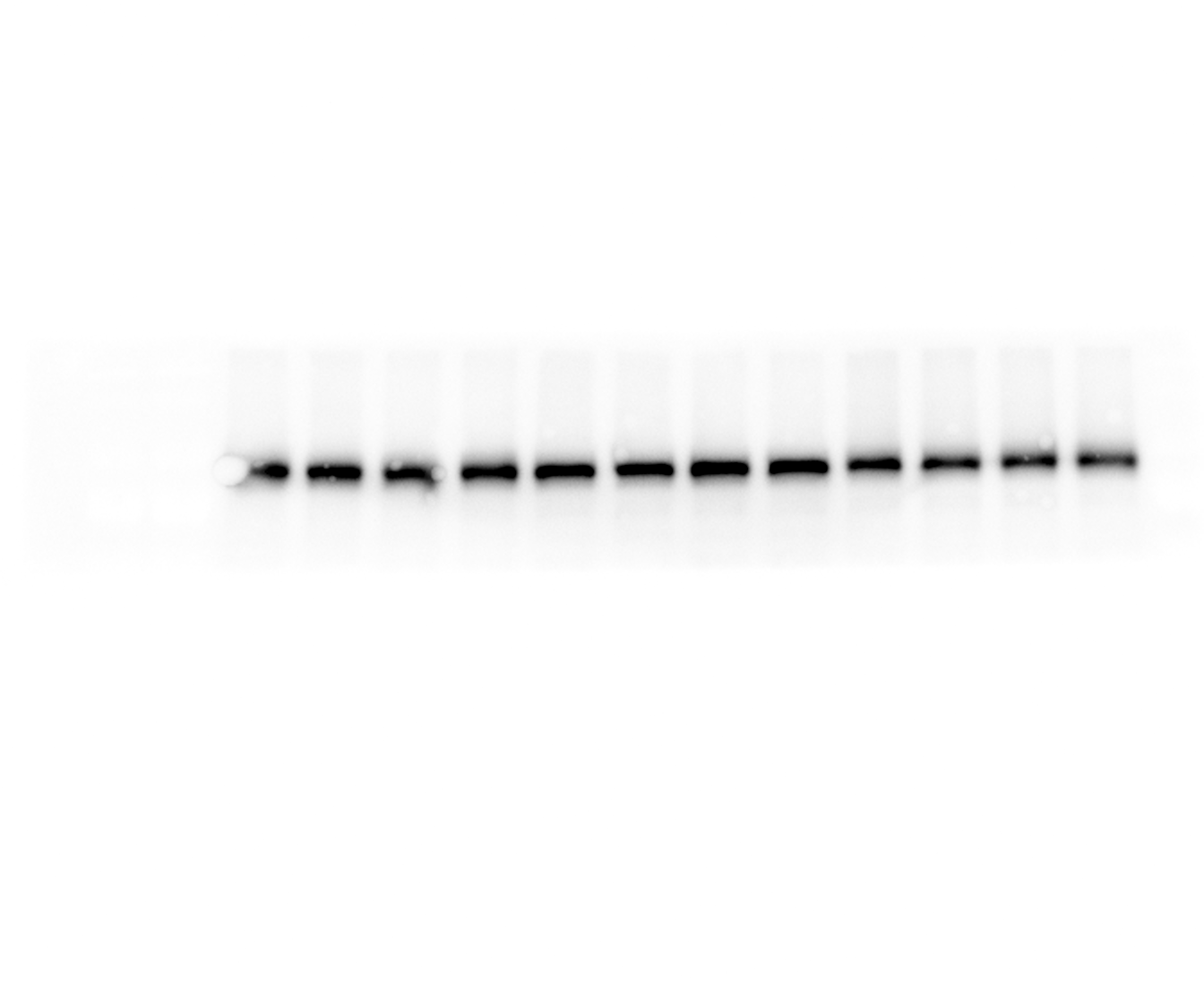

Supplement: Figure 4—figure supplement 1—source data 1. [file elife-69310-fig4-figsupp1-data1.zip › Figure 4-figure supplement 1-source data 1/MFN-2-001-3.Tif]

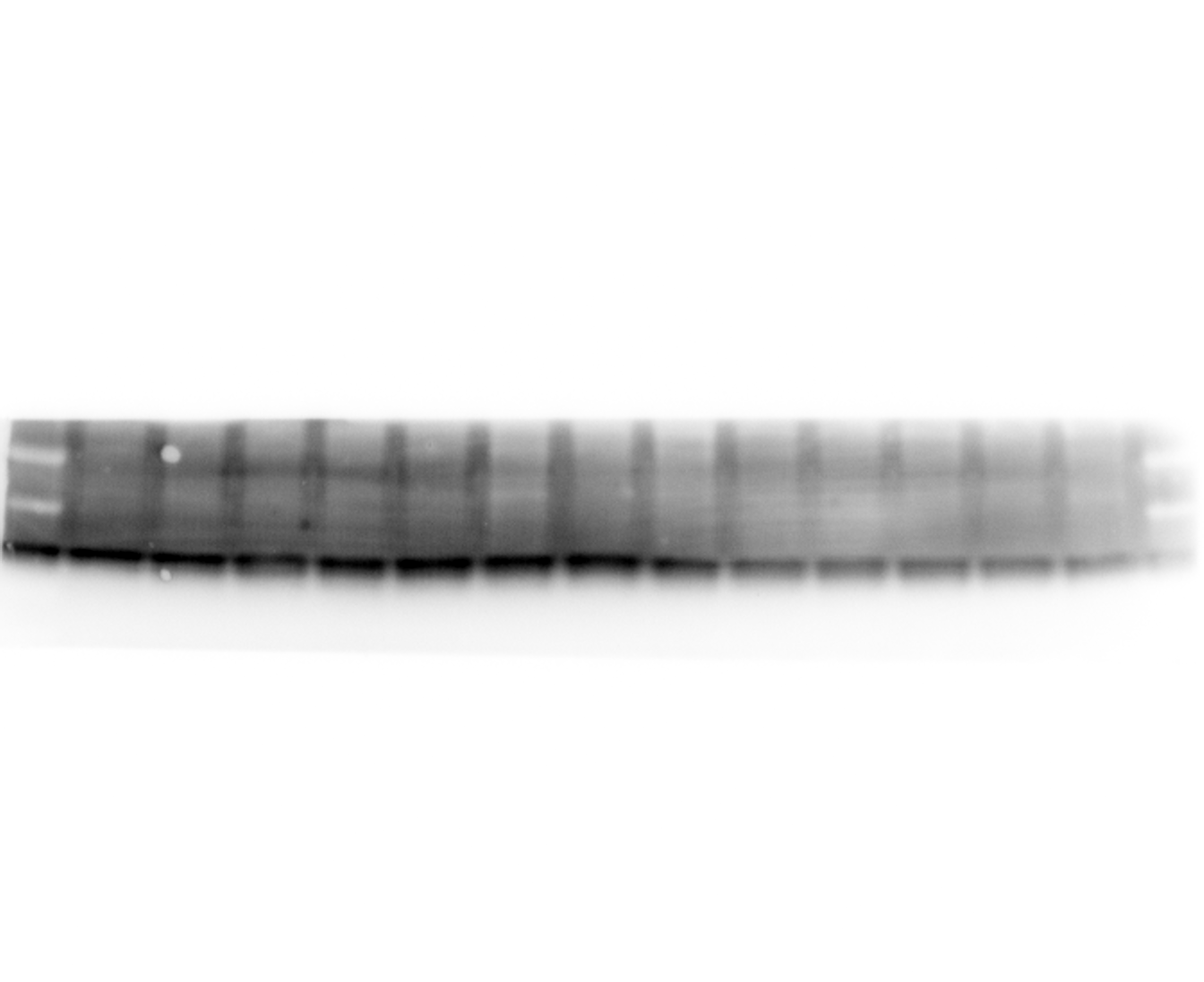

Supplement: Figure 4—figure supplement 1—source data 1. [file elife-69310-fig4-figsupp1-data1.zip › Figure 4-figure supplement 1-source data 1/NICD-001.Tif]

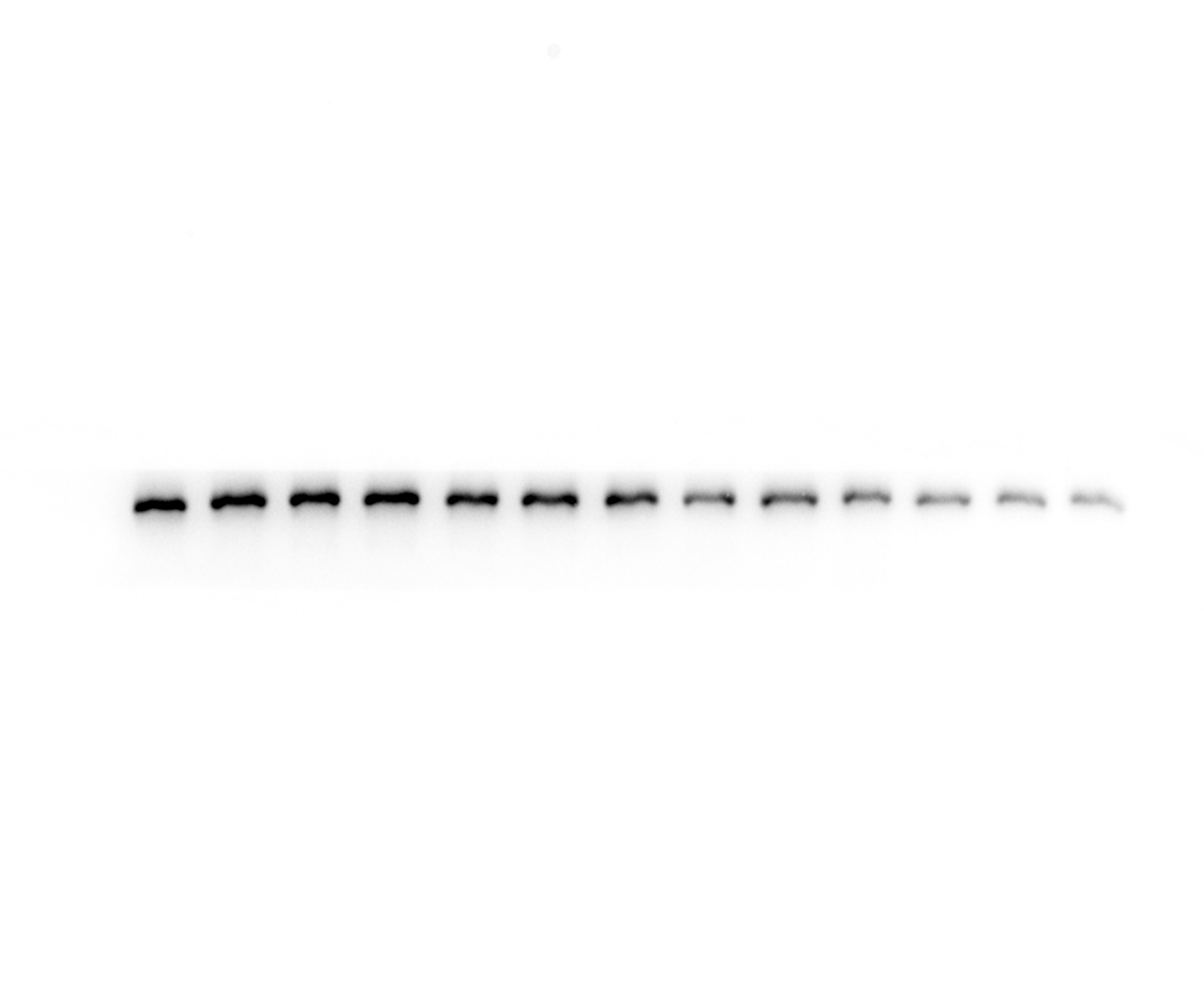

Supplement: Figure 4—figure supplement 1—source data 1. [file elife-69310-fig4-figsupp1-data1.zip › Figure 4-figure supplement 1-source data 1/NOTCH1-001.Tif]

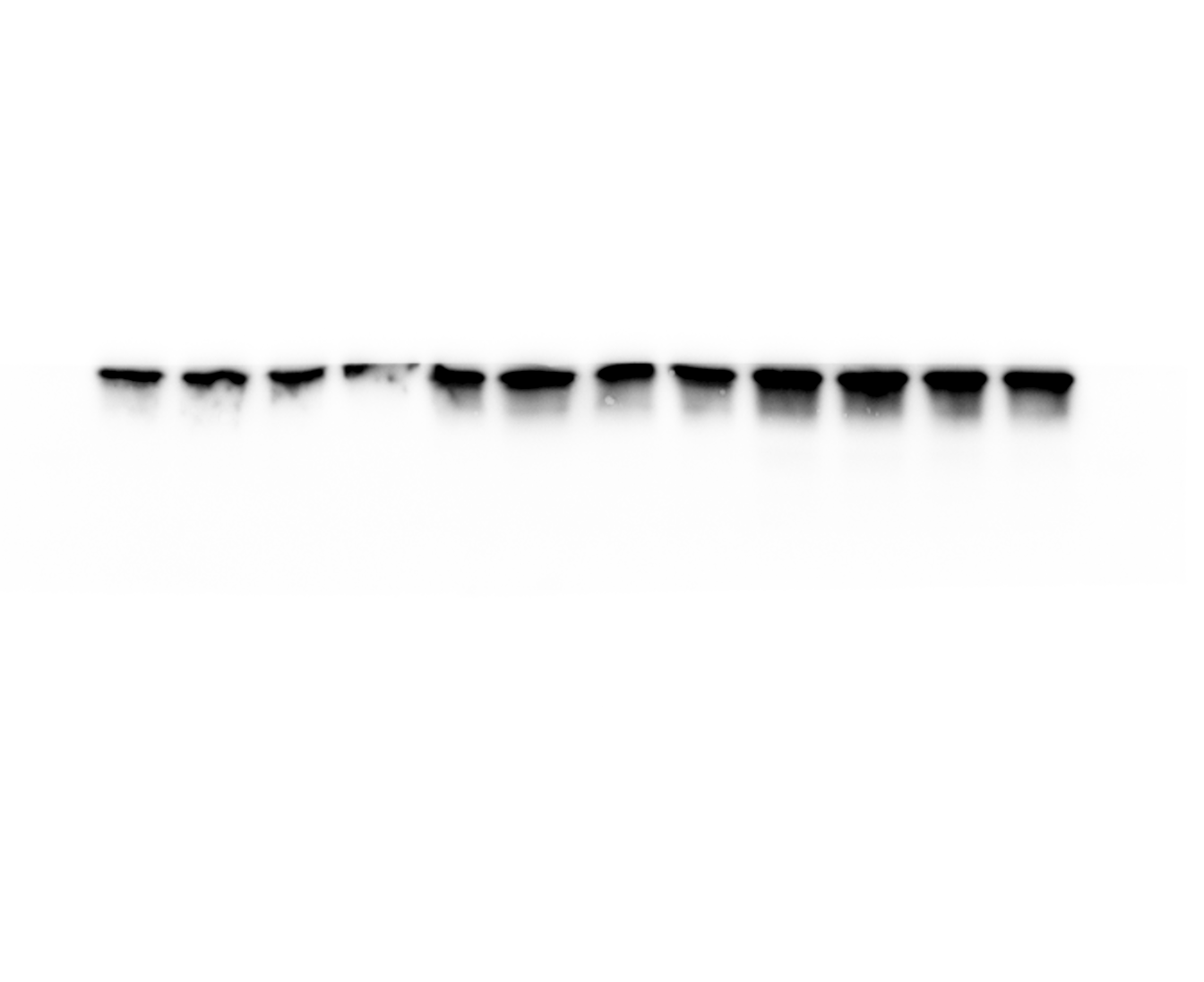

Supplement: Figure 4—figure supplement 1—source data 1. [file elife-69310-fig4-figsupp1-data1.zip › Figure 4-figure supplement 1-source data 1/SM22-001-1.Tif]

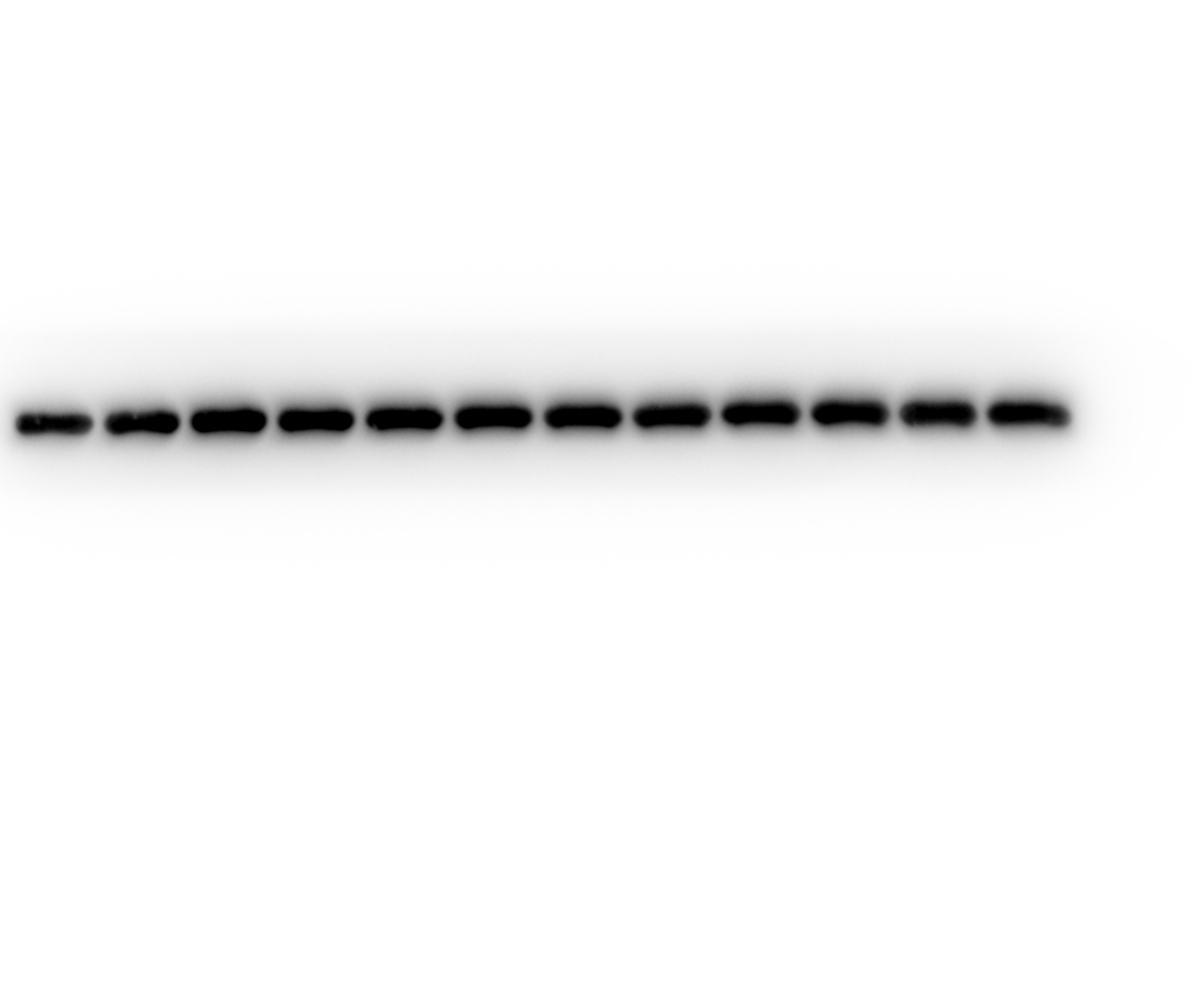

Supplement: Figure 5—source data 5. [file elife-69310-fig5-data5.zip › Figure 5-source data 5/b-actin-003-1.Tif]

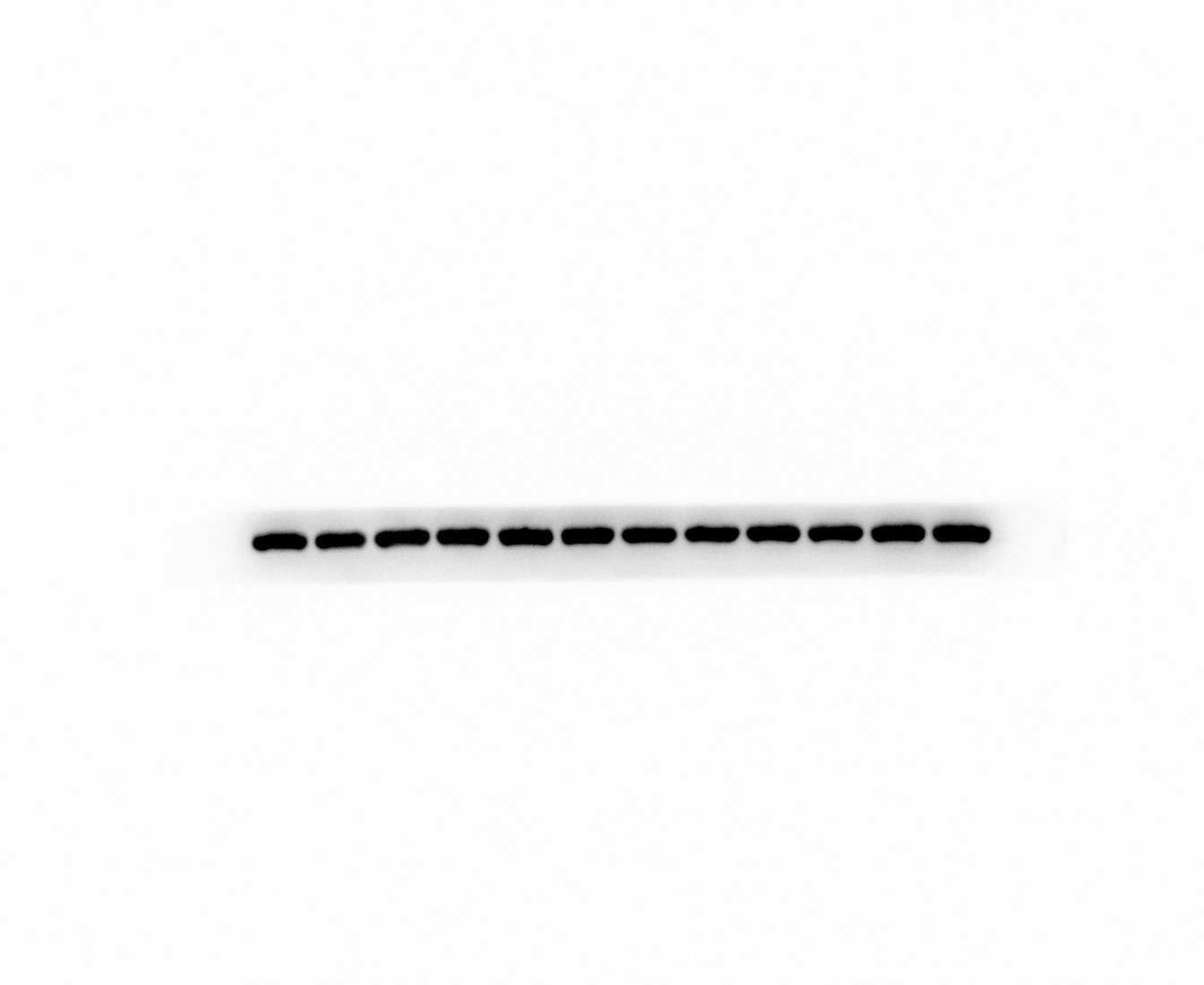

Supplement: Figure 5—source data 5. [file elife-69310-fig5-data5.zip › Figure 5-source data 5/b-actin-1_191217_151358_00.01.000_1_11986.tif]

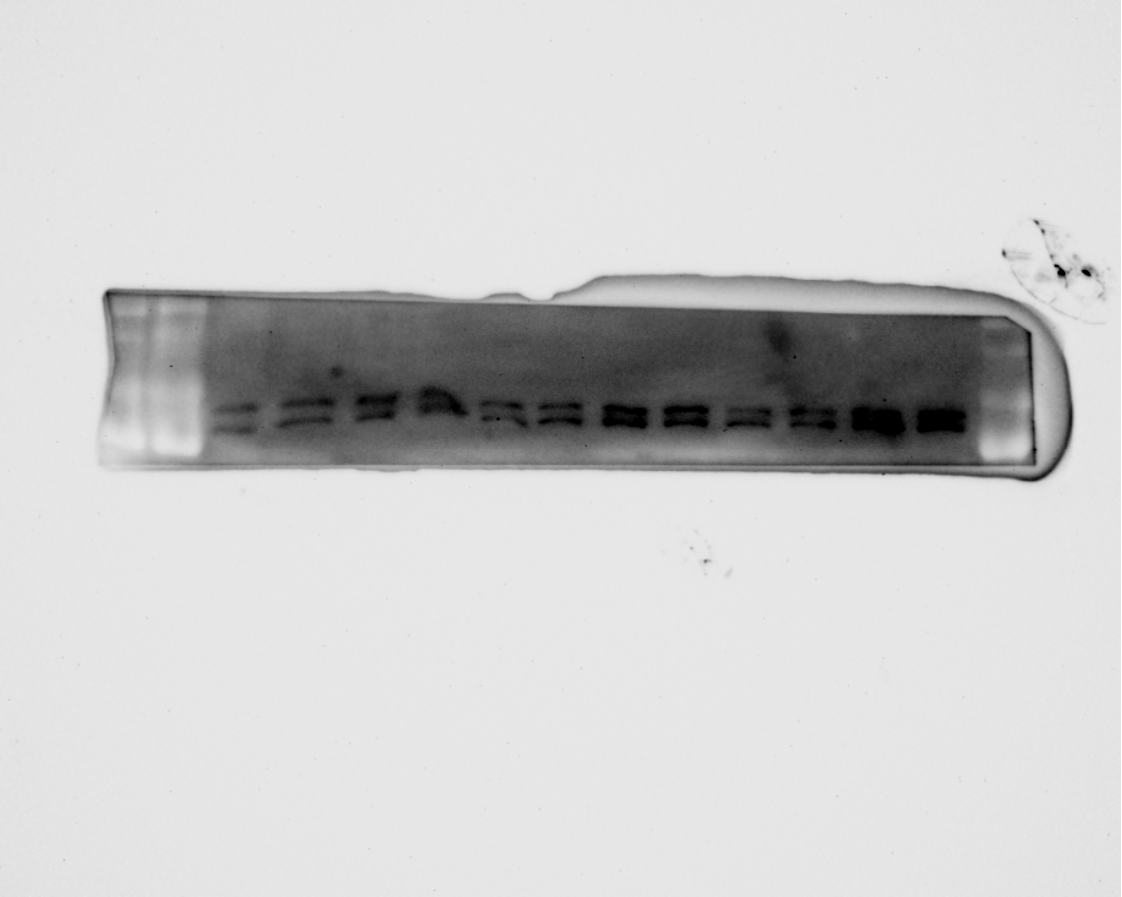

Supplement: Figure 5—source data 5. [file elife-69310-fig5-data5.zip › Figure 5-source data 5/drp-1(Chemiluminescence).tif]

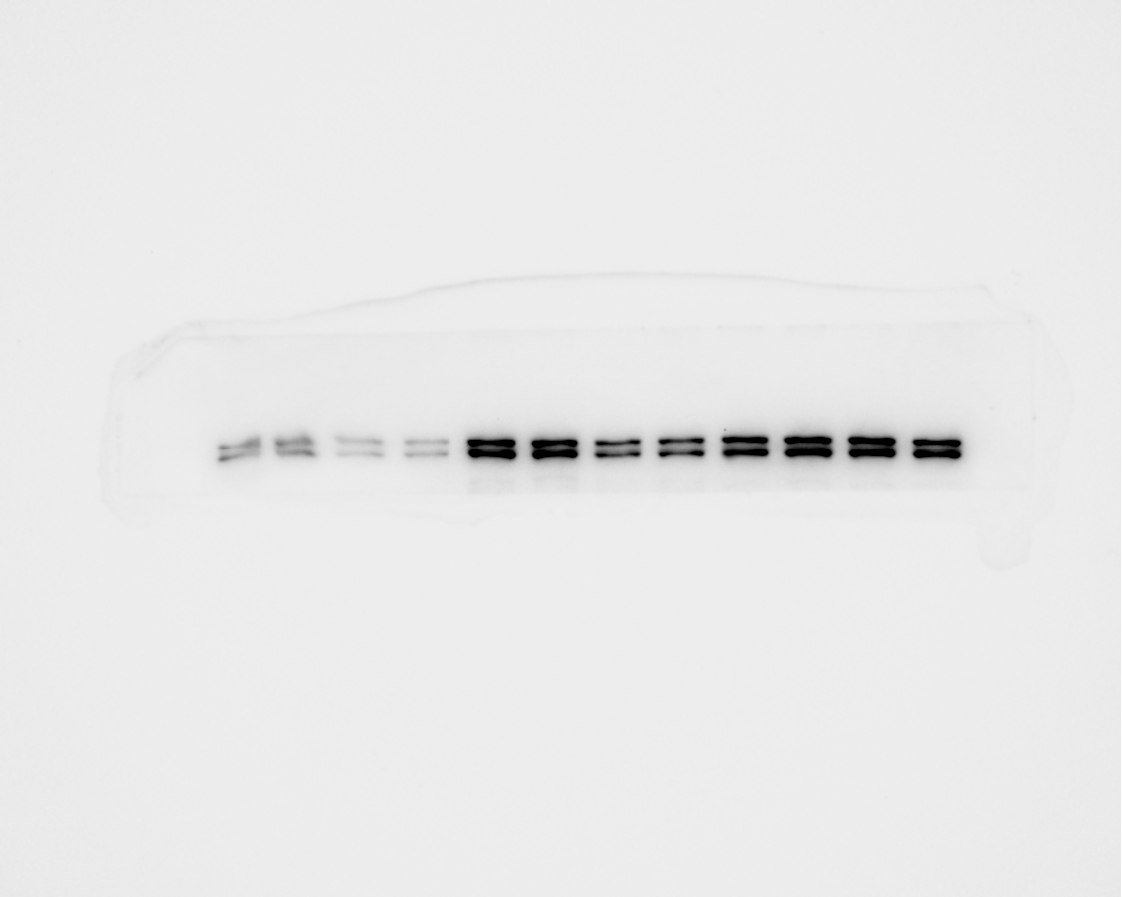

Supplement: Figure 5—source data 5. [file elife-69310-fig5-data5.zip › Figure 5-source data 5/drp1-1_1(Chemiluminescence).jpg]

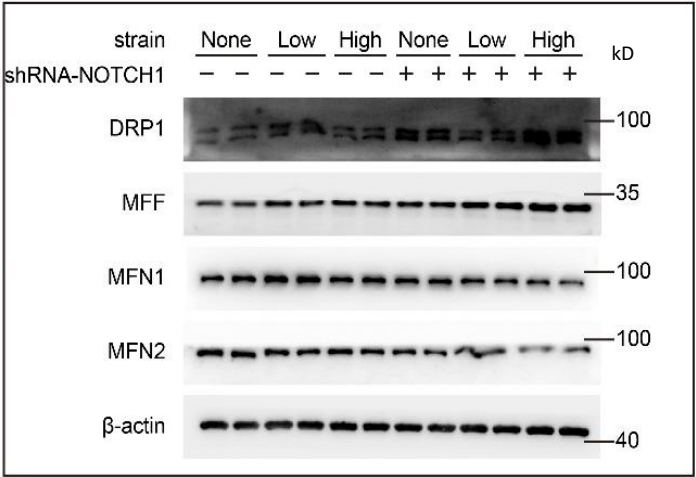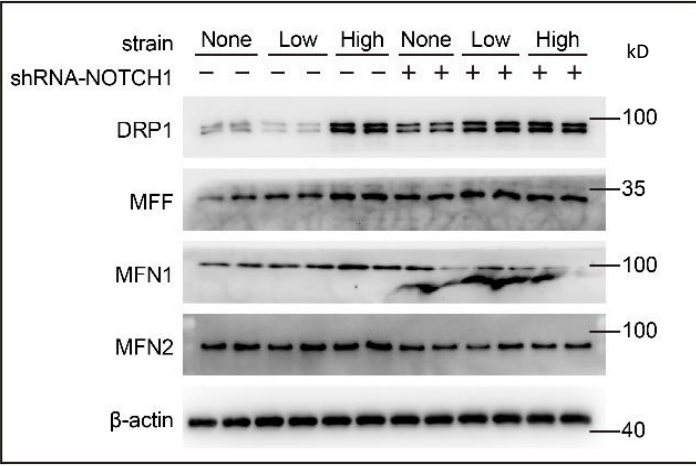

Supplement: Figure 5—source data 5. [file elife-69310-fig5-data5.zip › Figure 5-source data 5/labeled uncropped WB .pdf]

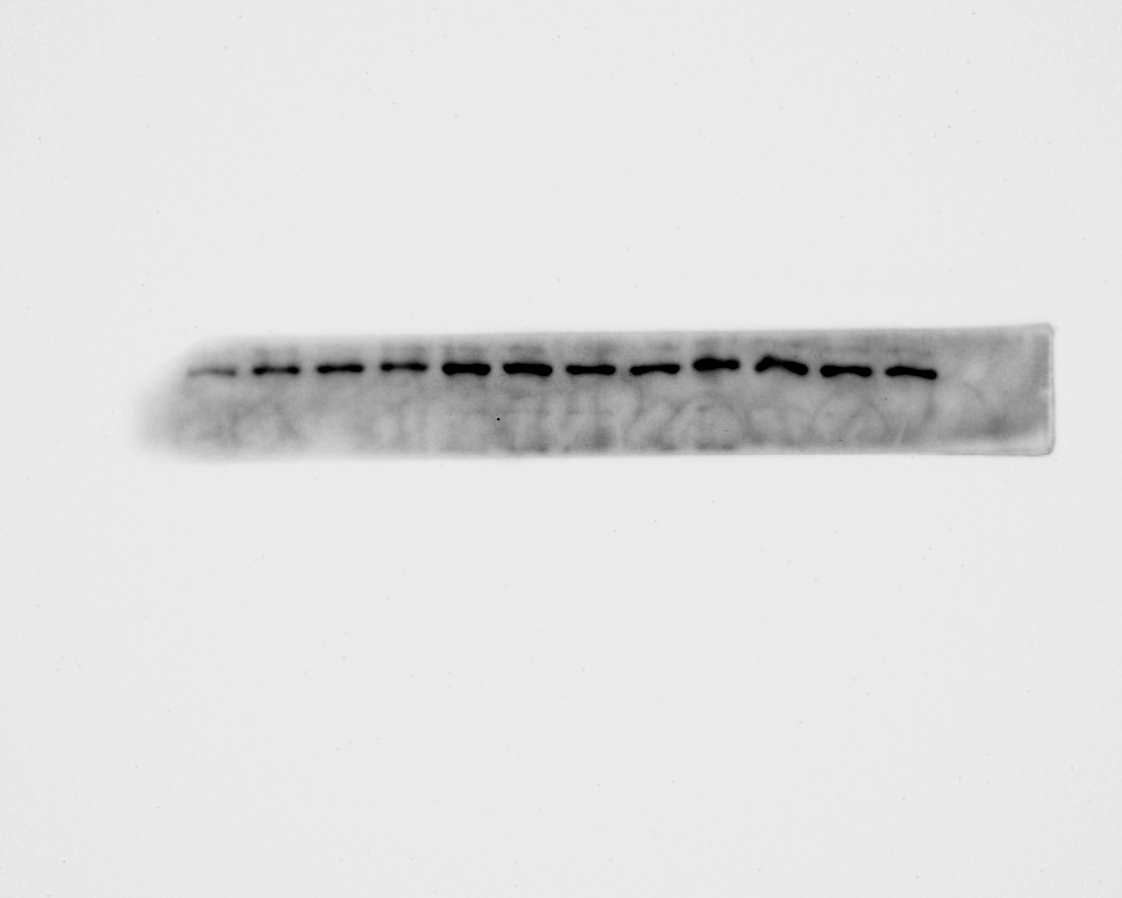

Supplement: Figure 5—source data 5. [file elife-69310-fig5-data5.zip › Figure 5-source data 5/mff-002_2(Chemiluminescence).jpg]

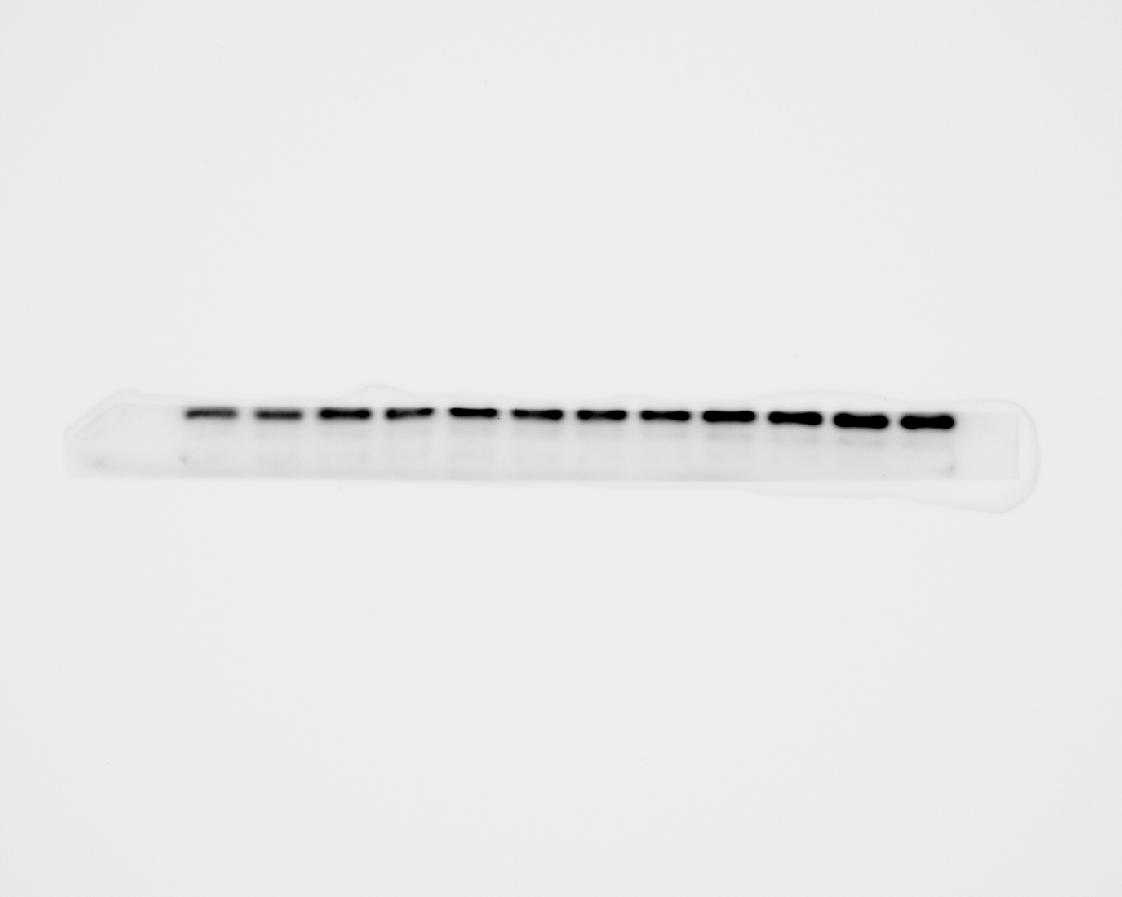

Supplement: Figure 5—source data 5. [file elife-69310-fig5-data5.zip › Figure 5-source data 5/mff_1(Chemiluminescence).tif]

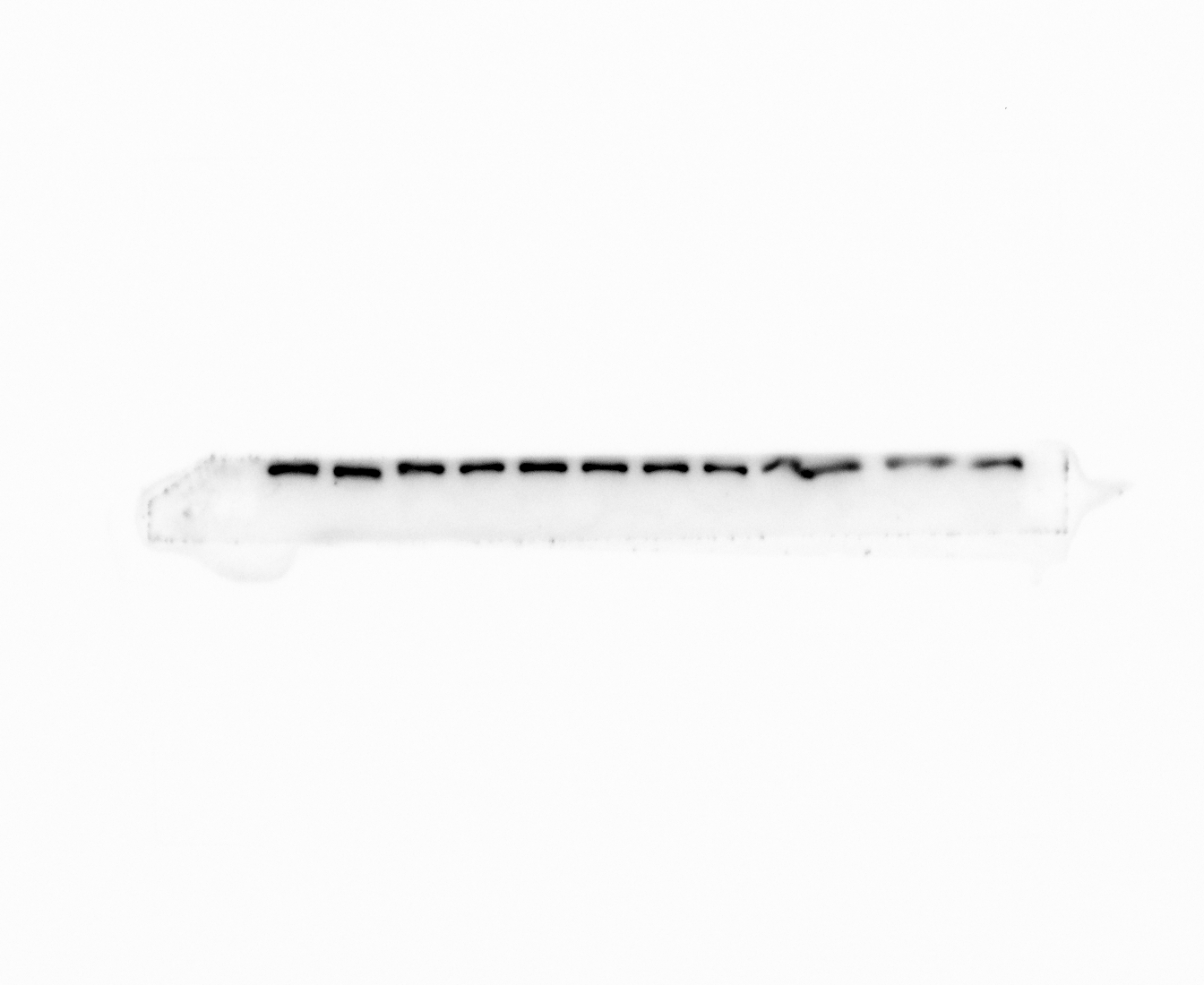

Supplement: Figure 5—source data 5. [file elife-69310-fig5-data5.zip › Figure 5-source data 5/mfn-2_191217_144056_01.18.000_1_7421.tif]

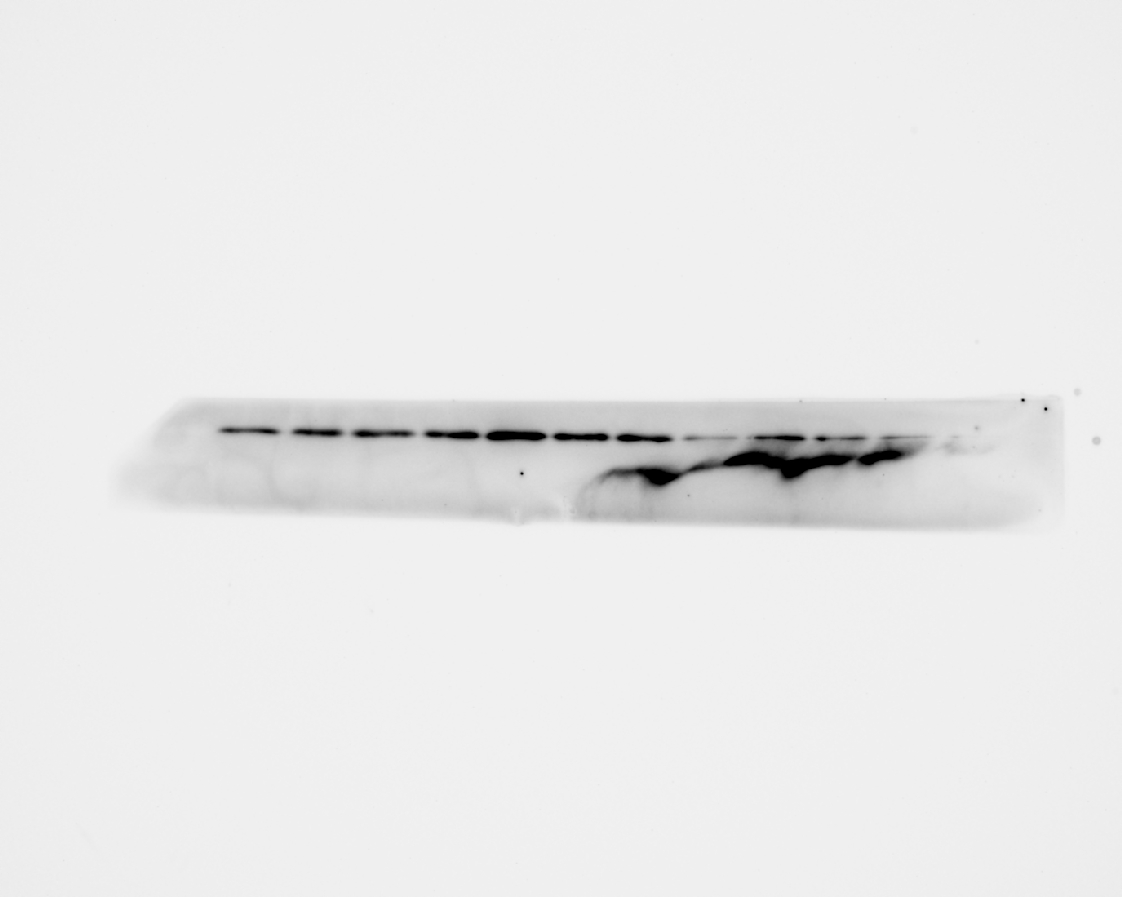

Supplement: Figure 5—source data 5. [file elife-69310-fig5-data5.zip › Figure 5-source data 5/mfn1(Chemiluminescence).jpg]

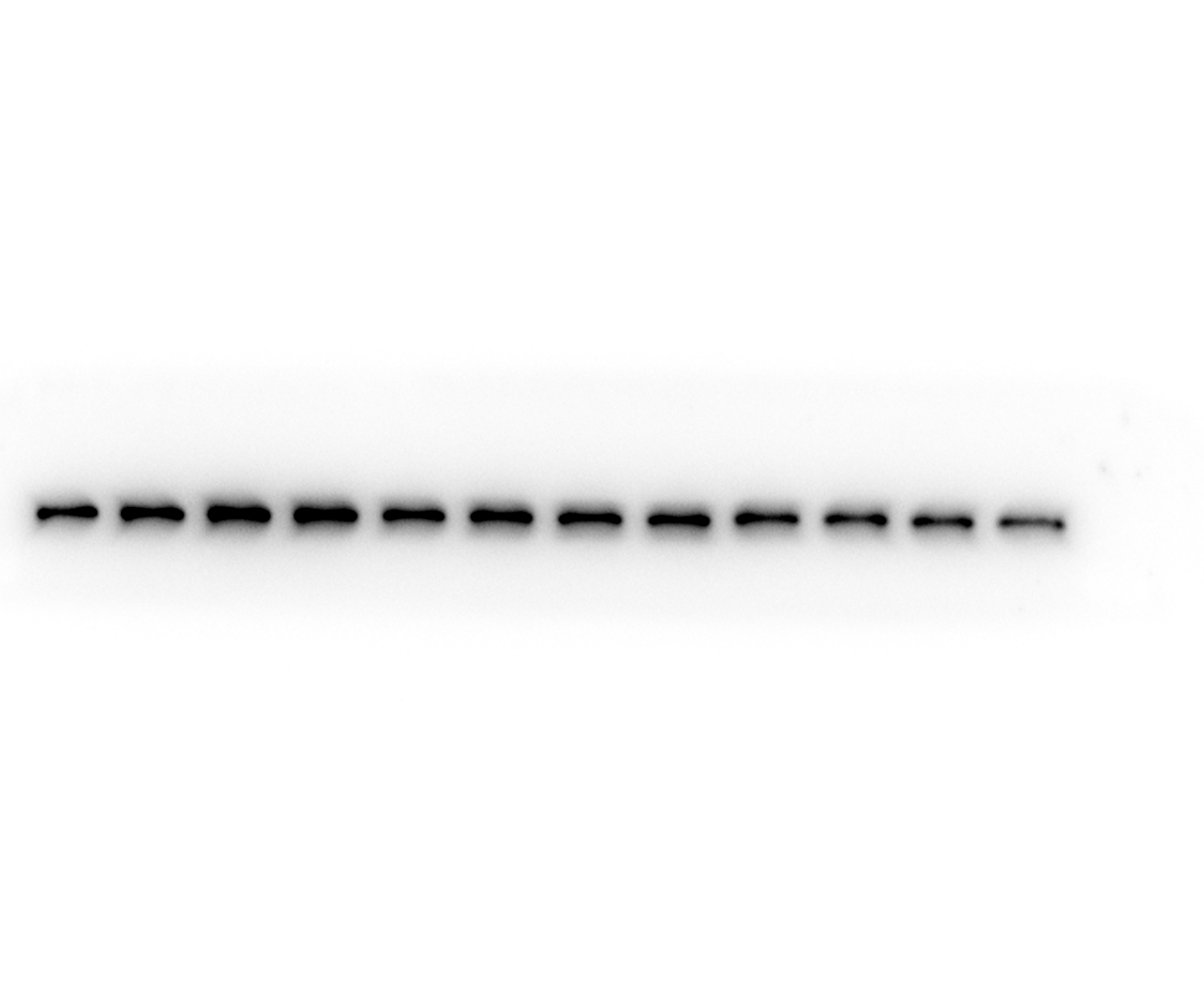

Supplement: Figure 5—source data 5. [file elife-69310-fig5-data5.zip › Figure 5-source data 5/MFN1-003-1.Tif]

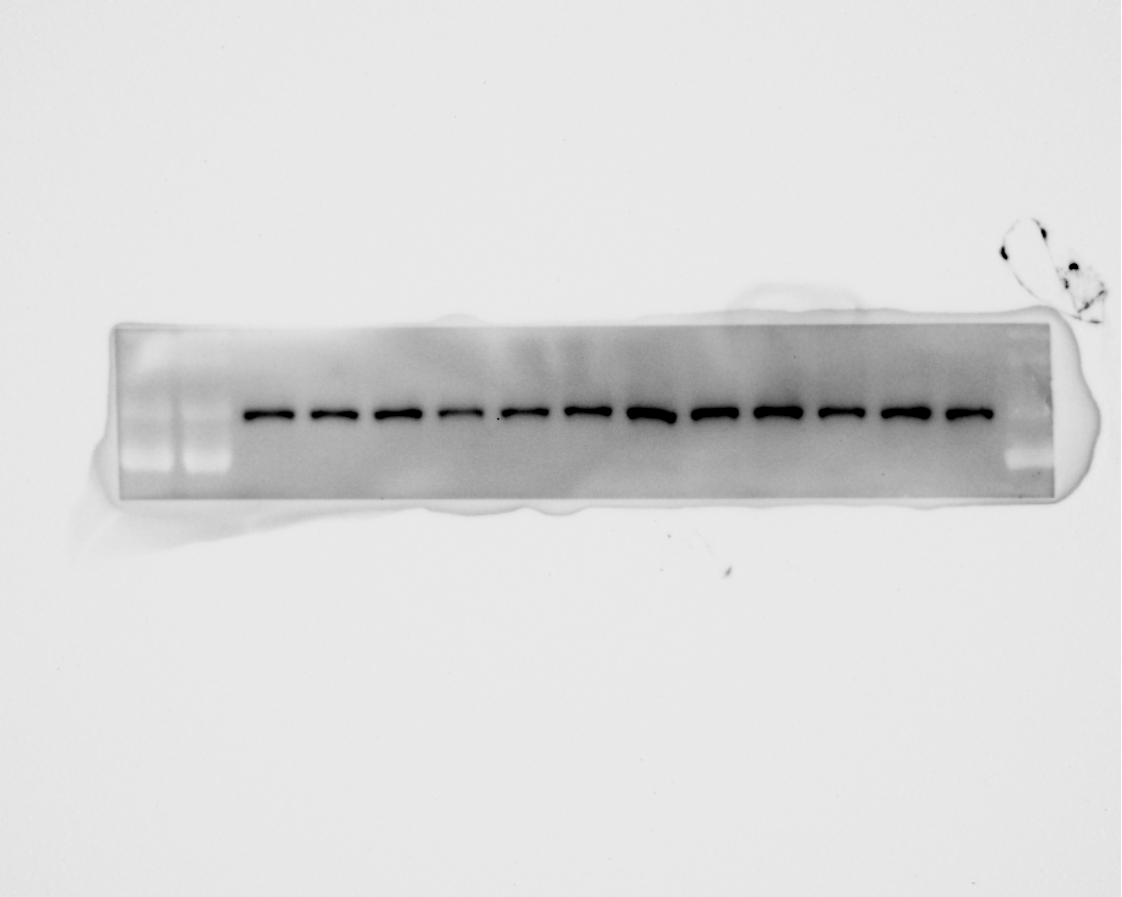

Supplement: Figure 5—source data 5. [file elife-69310-fig5-data5.zip › Figure 5-source data 5/mfn_2(Chemiluminescence).jpg]

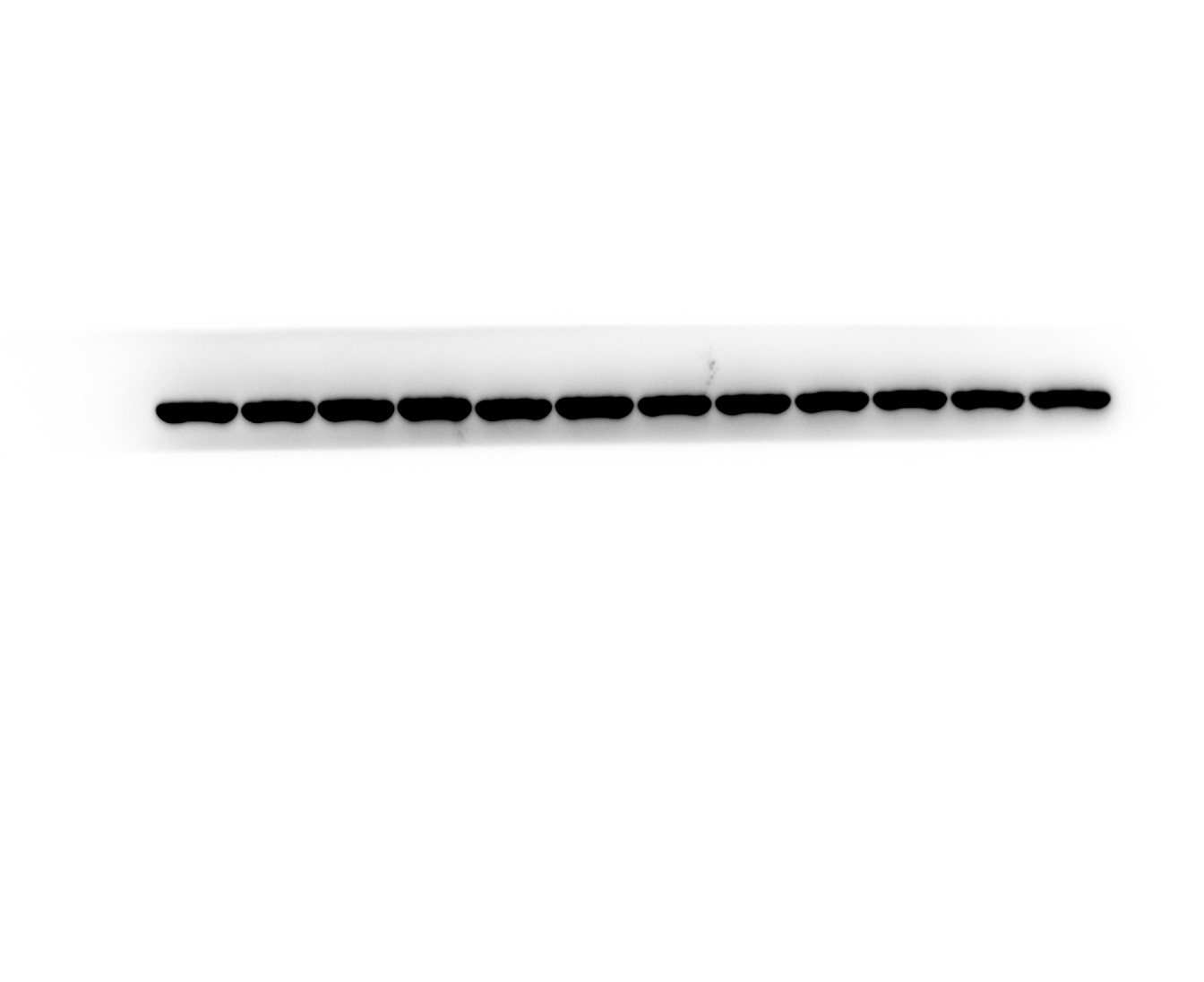

Supplement: Figure 6—source data 1. [file elife-69310-fig6-data1.zip › Figure 6-source data 1/BAV-TAA patient #1(figure 6e)/B-ACTIN-001-3.Tif]

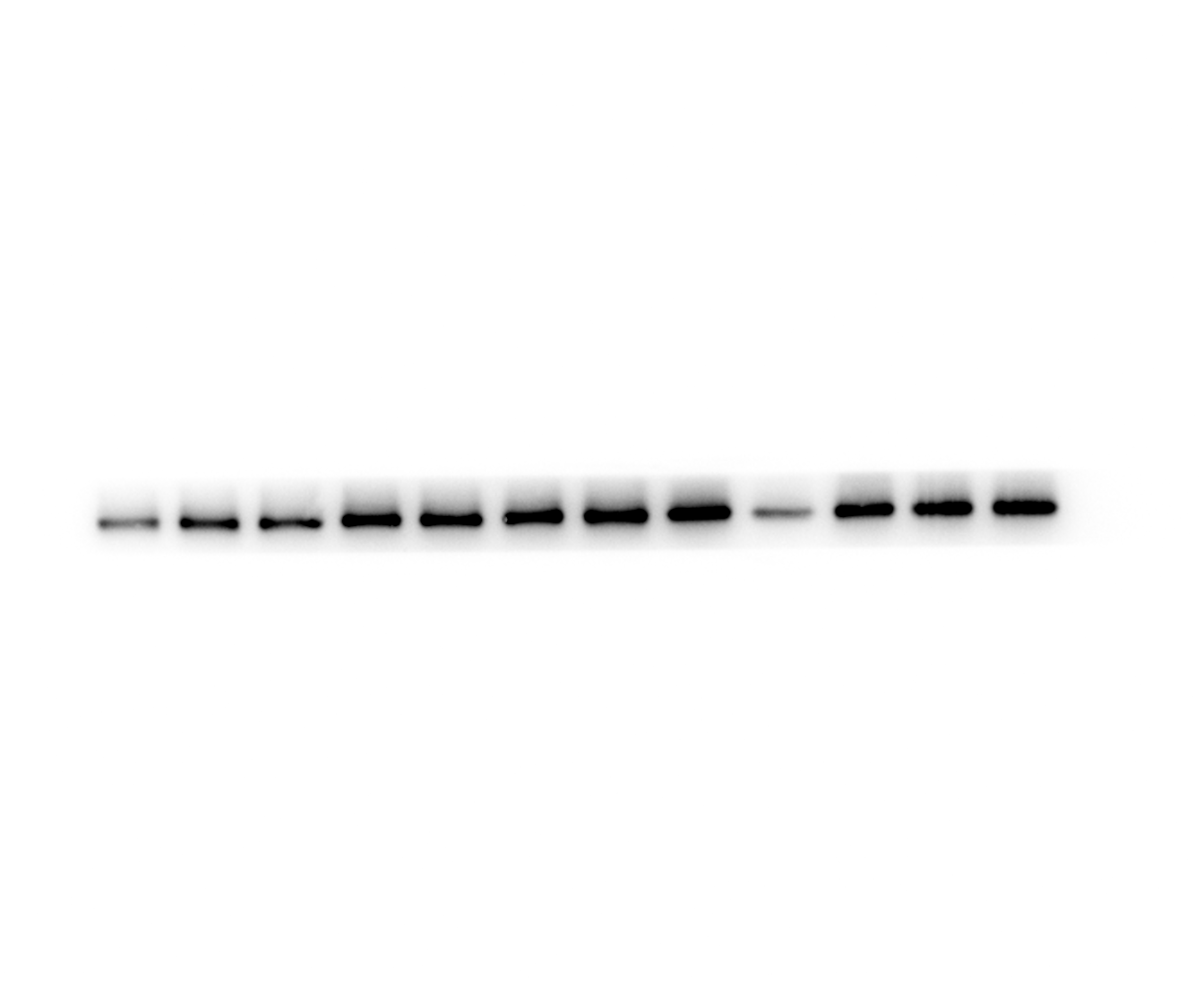

Supplement: Figure 6—source data 1. [file elife-69310-fig6-data1.zip › Figure 6-source data 1/BAV-TAA patient #1(figure 6e)/CNN1-003-3.Tif]

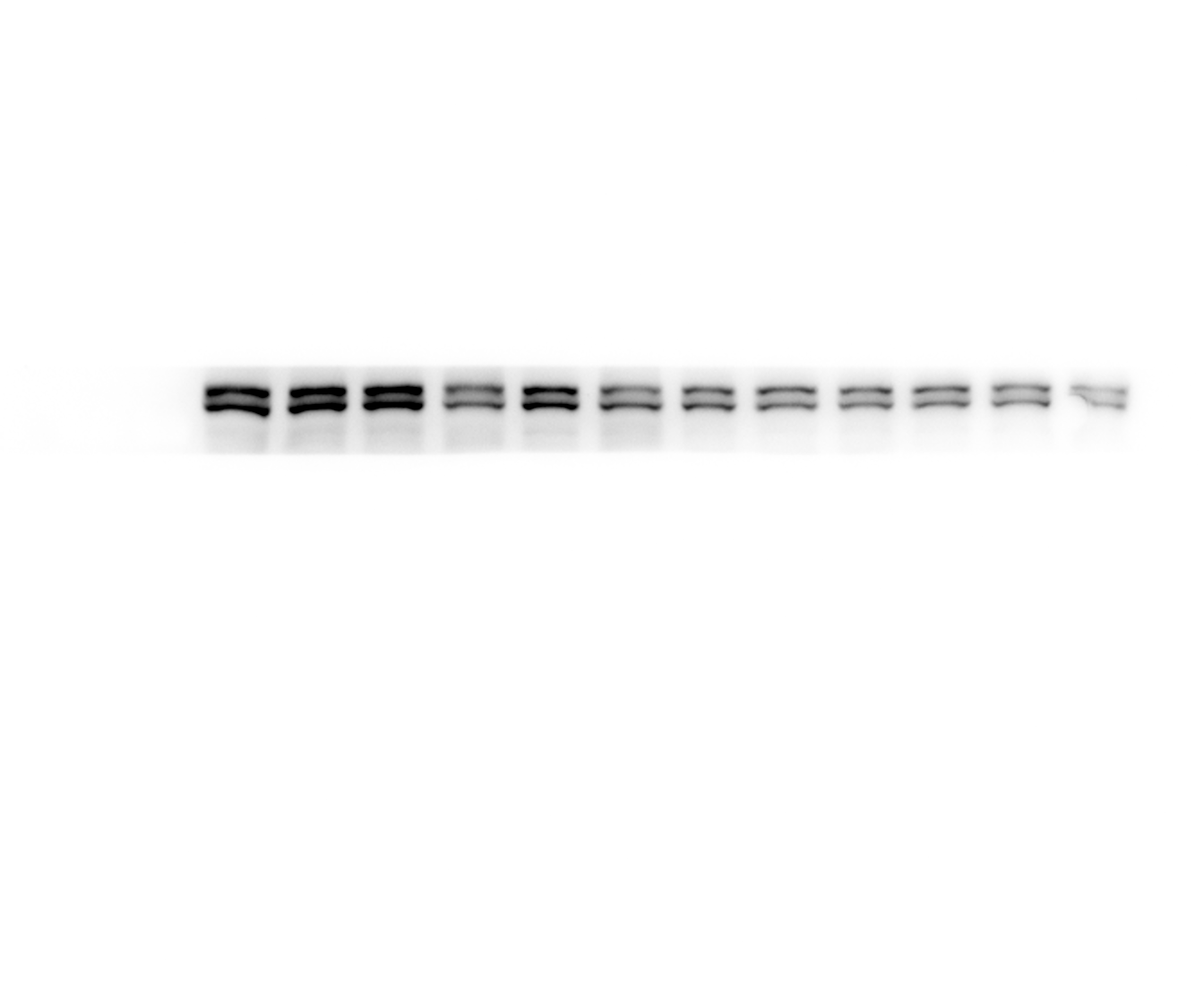

Supplement: Figure 6—source data 1. [file elife-69310-fig6-data1.zip › Figure 6-source data 1/BAV-TAA patient #1(figure 6e)/DRP-1-002-3.Tif]

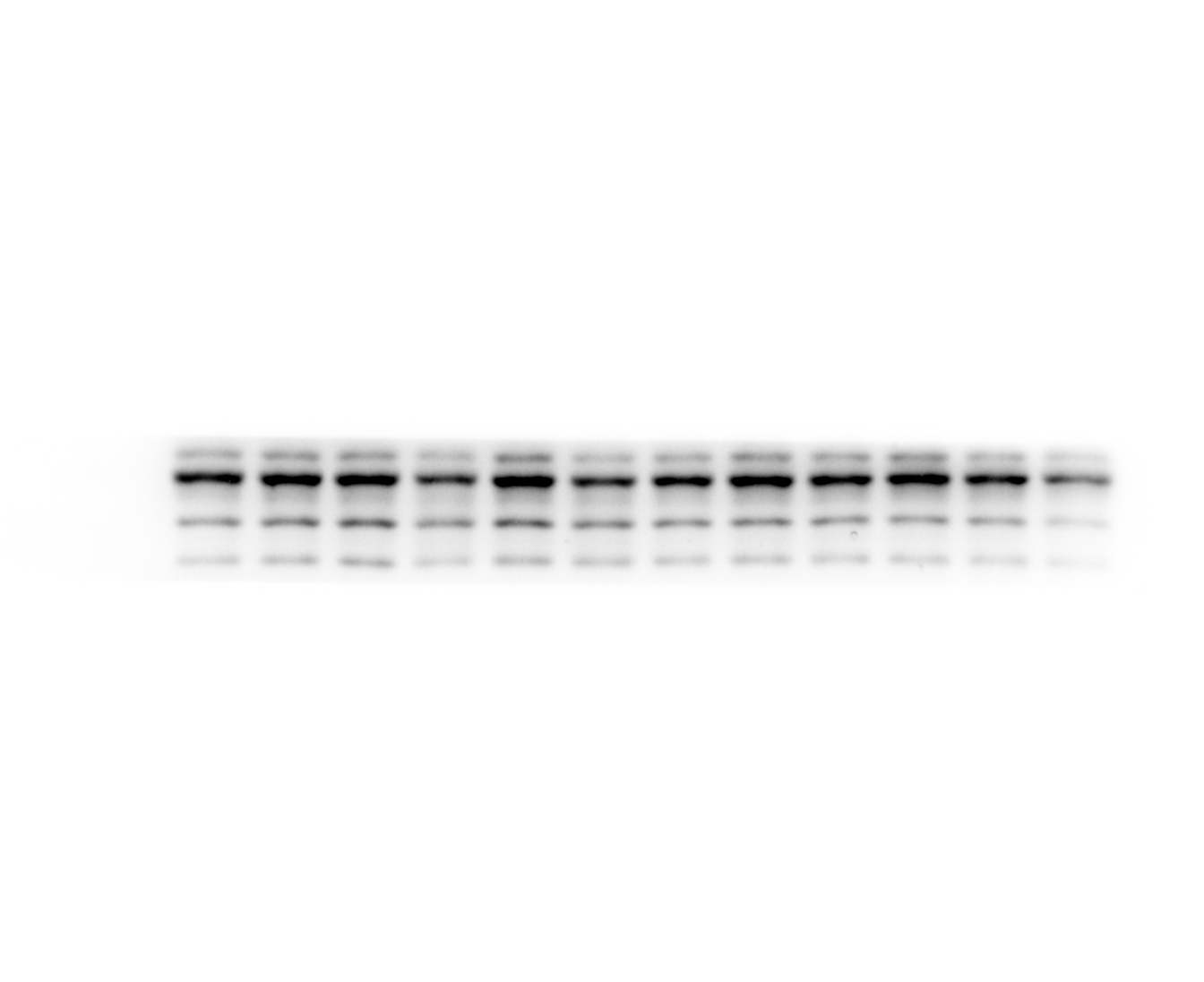

Supplement: Figure 6—source data 1. [file elife-69310-fig6-data1.zip › Figure 6-source data 1/BAV-TAA patient #1(figure 6e)/MFF-002-3.Tif]

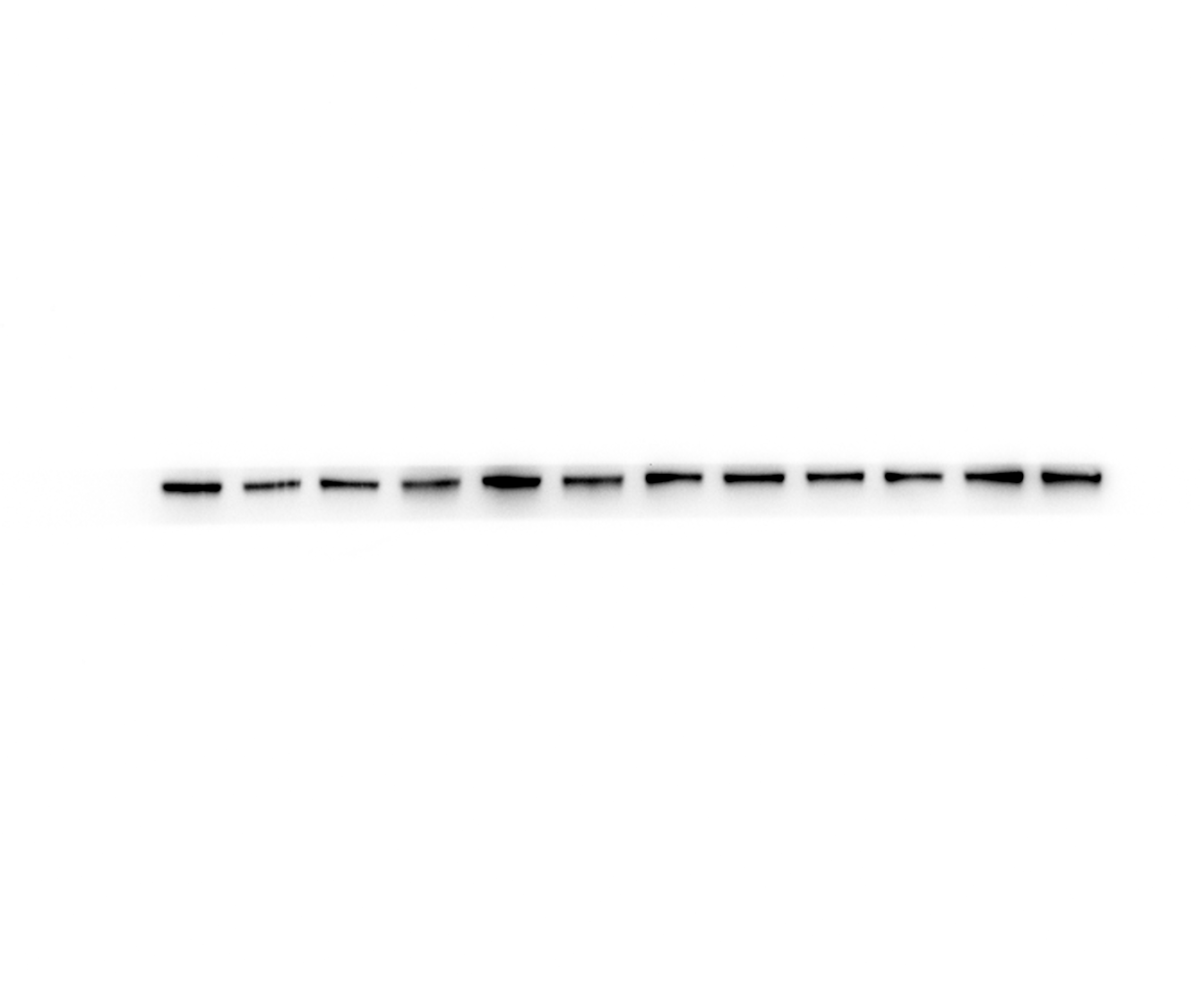

Supplement: Figure 6—source data 1. [file elife-69310-fig6-data1.zip › Figure 6-source data 1/BAV-TAA patient #1(figure 6e)/MFN-1-003-1.Tif]

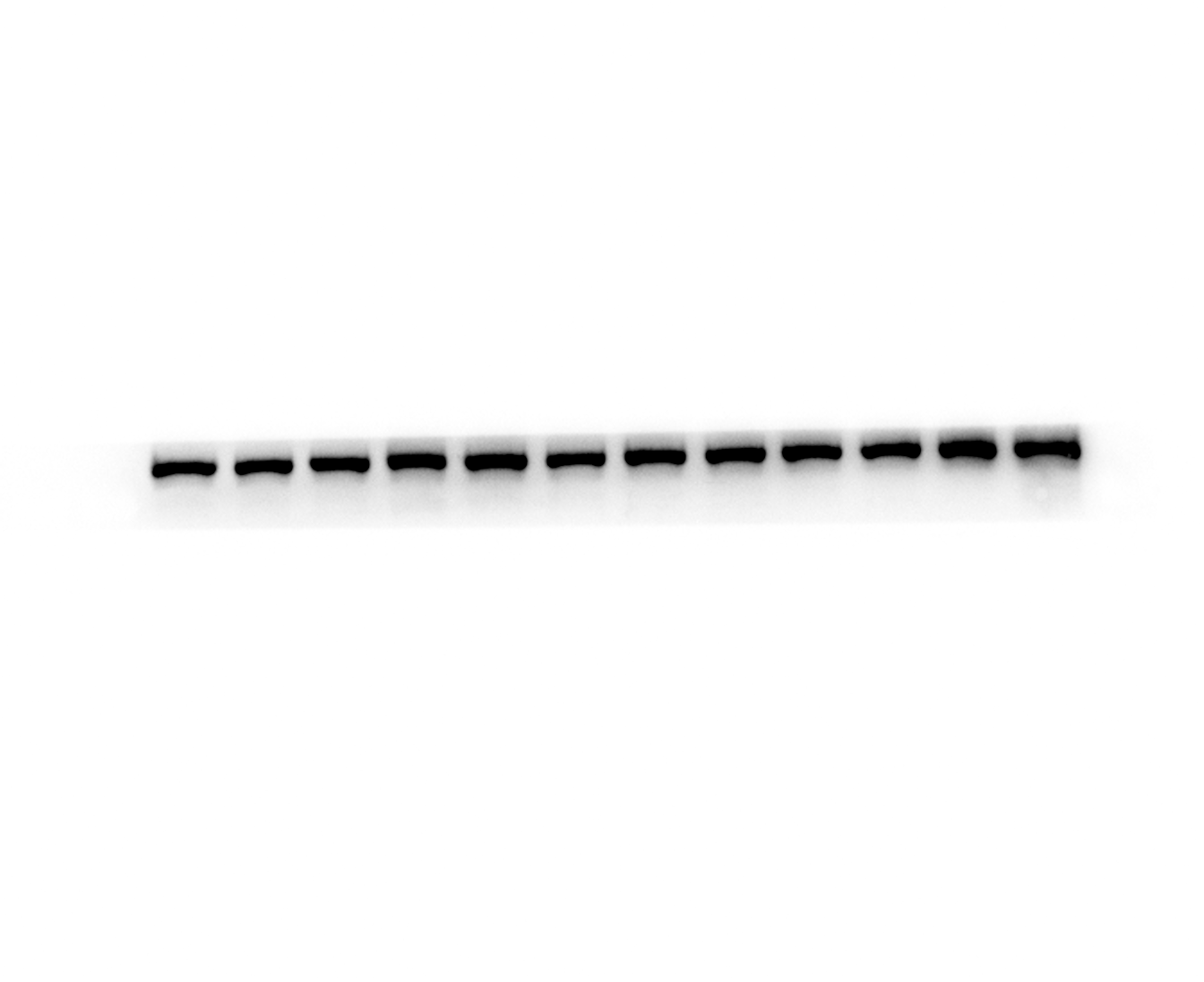

Supplement: Figure 6—source data 1. [file elife-69310-fig6-data1.zip › Figure 6-source data 1/BAV-TAA patient #1(figure 6e)/MFN-2-001-1.Tif]

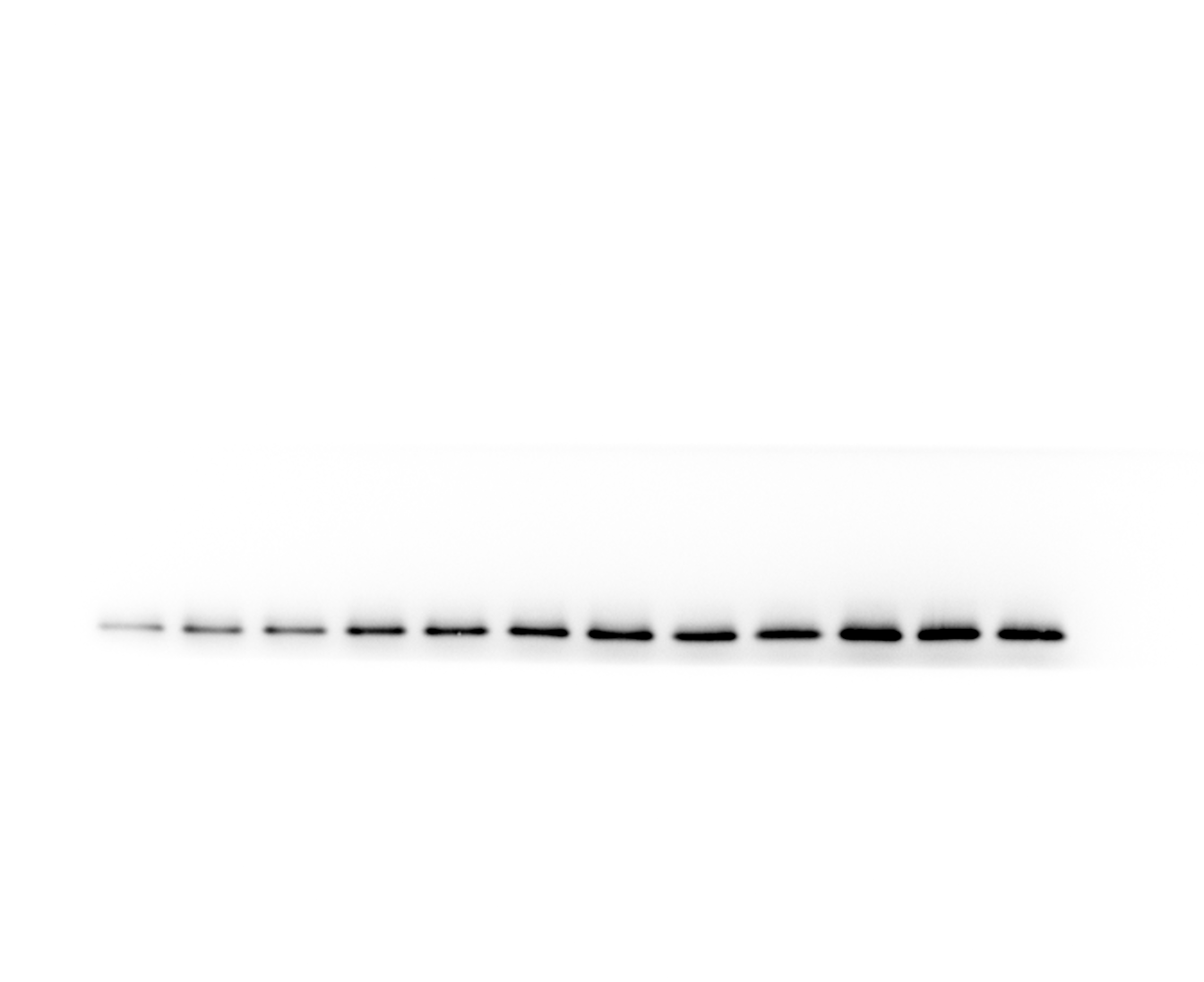

Supplement: Figure 6—source data 1. [file elife-69310-fig6-data1.zip › Figure 6-source data 1/BAV-TAA patient #1(figure 6e)/SM22-003-3.Tif]

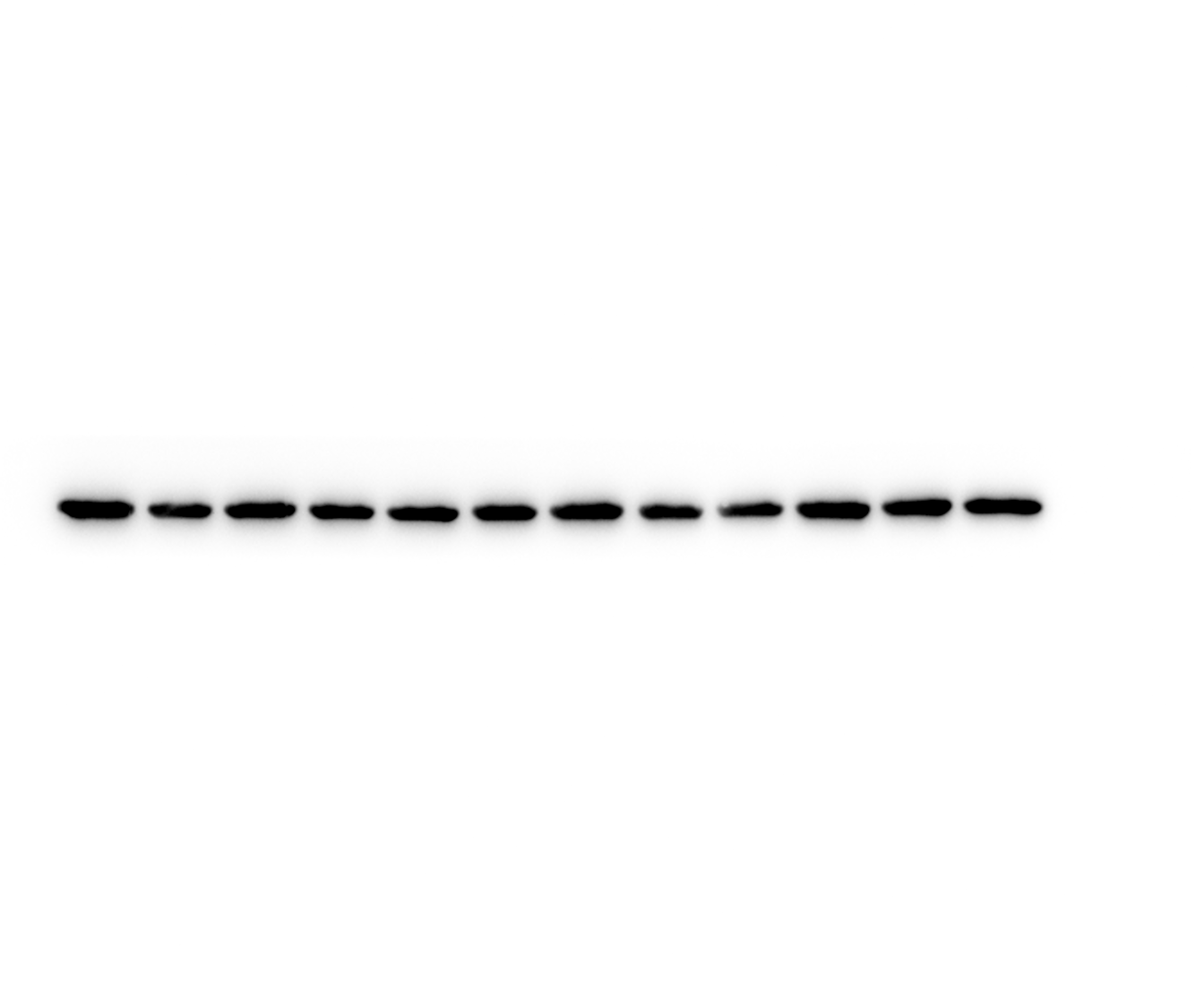

Supplement: Figure 6—source data 1. [file elife-69310-fig6-data1.zip › Figure 6-source data 1/BAV-TAA patient #2(figure 6f)/B-ACITN-002-3.Tif]

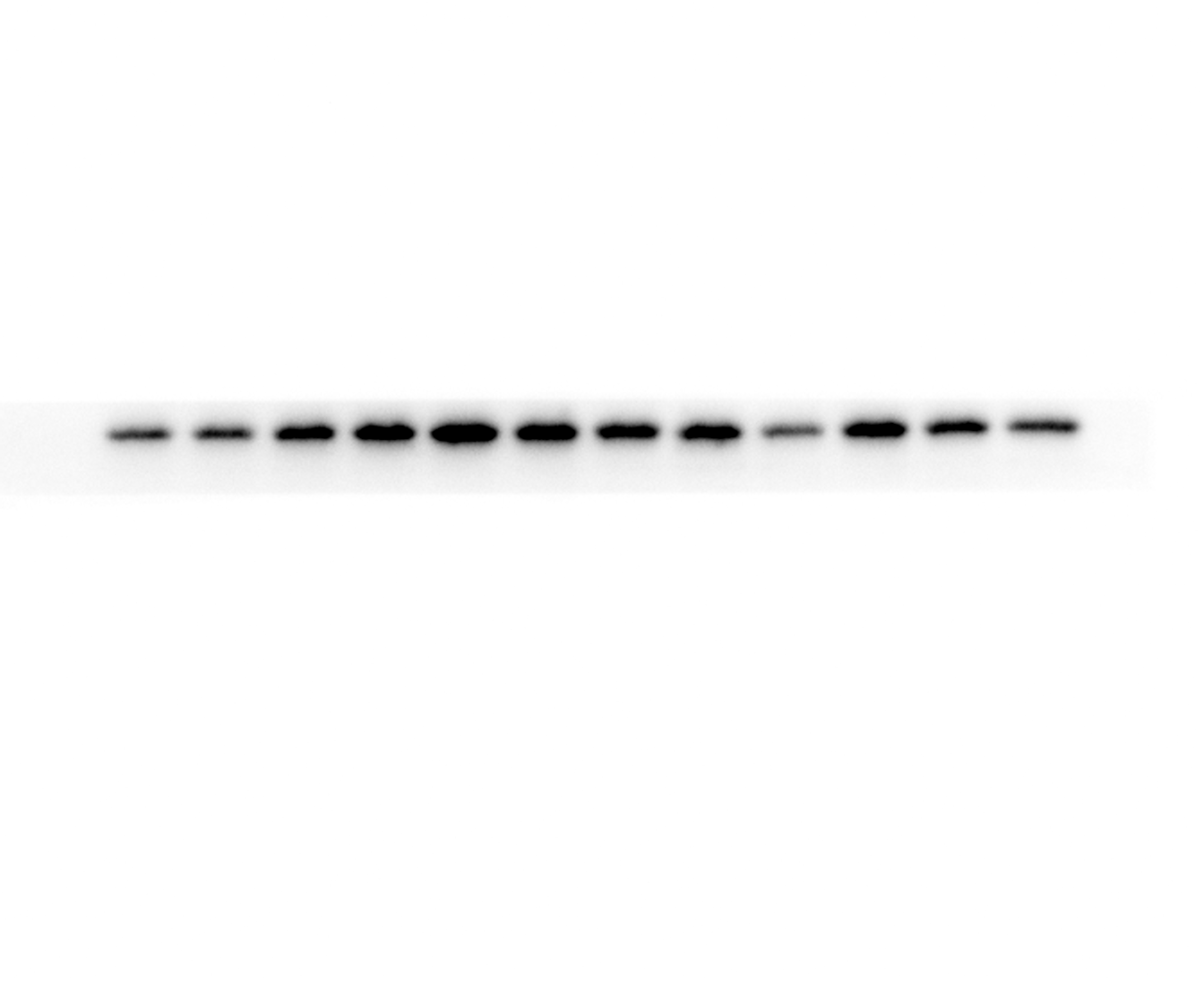

Supplement: Figure 6—source data 1. [file elife-69310-fig6-data1.zip › Figure 6-source data 1/BAV-TAA patient #2(figure 6f)/CNN1-001-3.Tif]

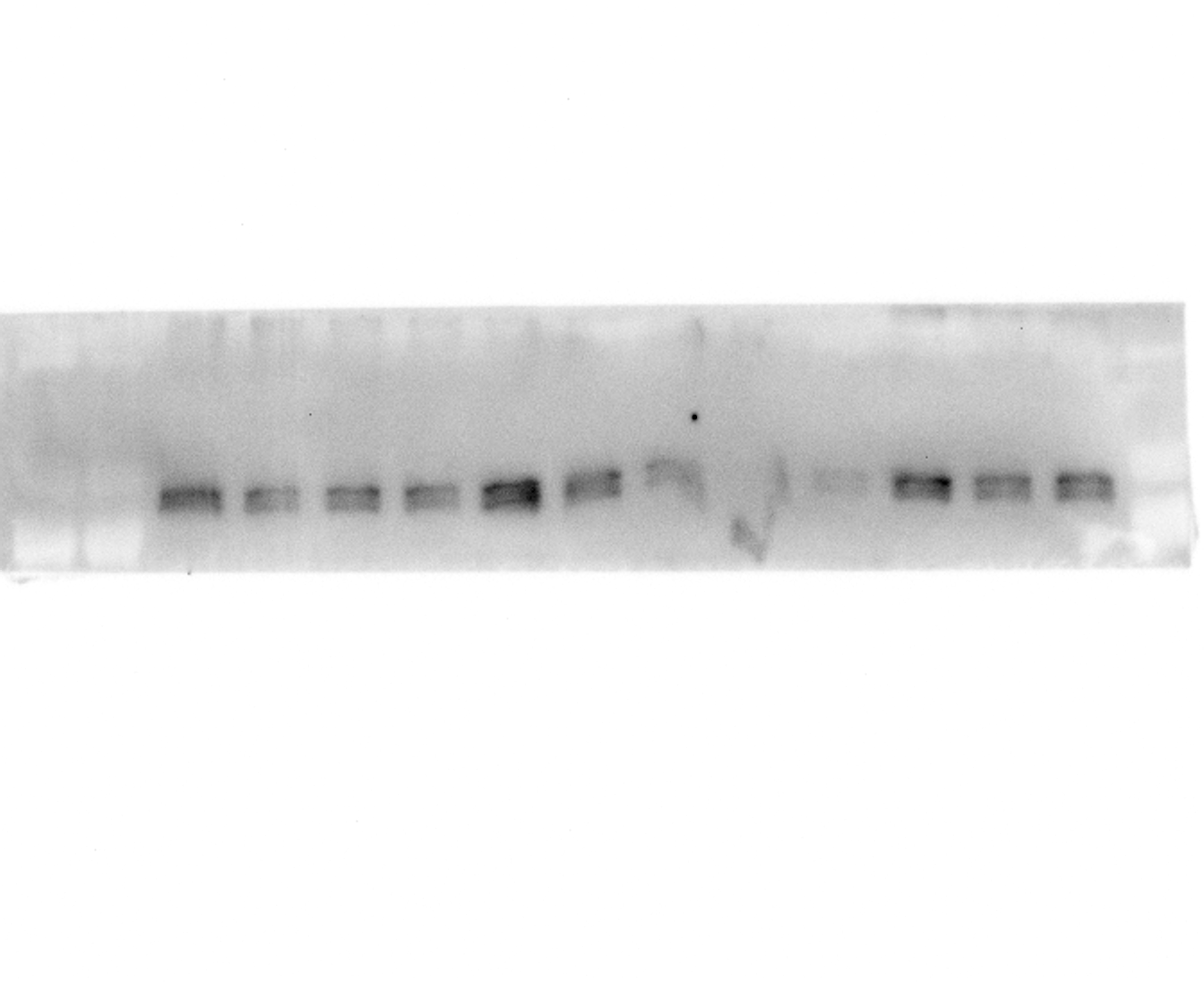

Supplement: Figure 6—source data 1. [file elife-69310-fig6-data1.zip › Figure 6-source data 1/BAV-TAA patient #2(figure 6f)/DRP-1-003-1.Tif]

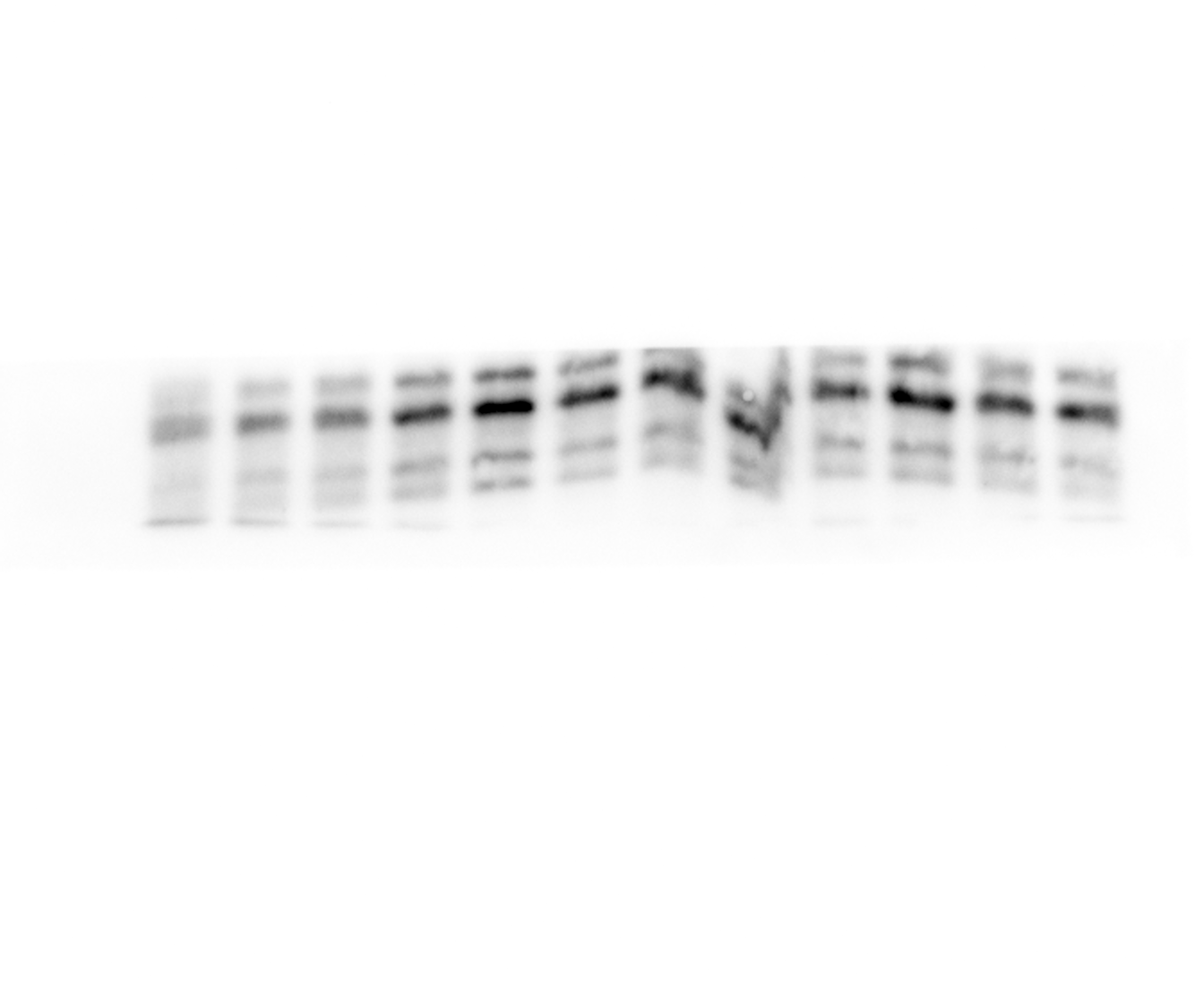

Supplement: Figure 6—source data 1. [file elife-69310-fig6-data1.zip › Figure 6-source data 1/BAV-TAA patient #2(figure 6f)/MFF-003-1.Tif]

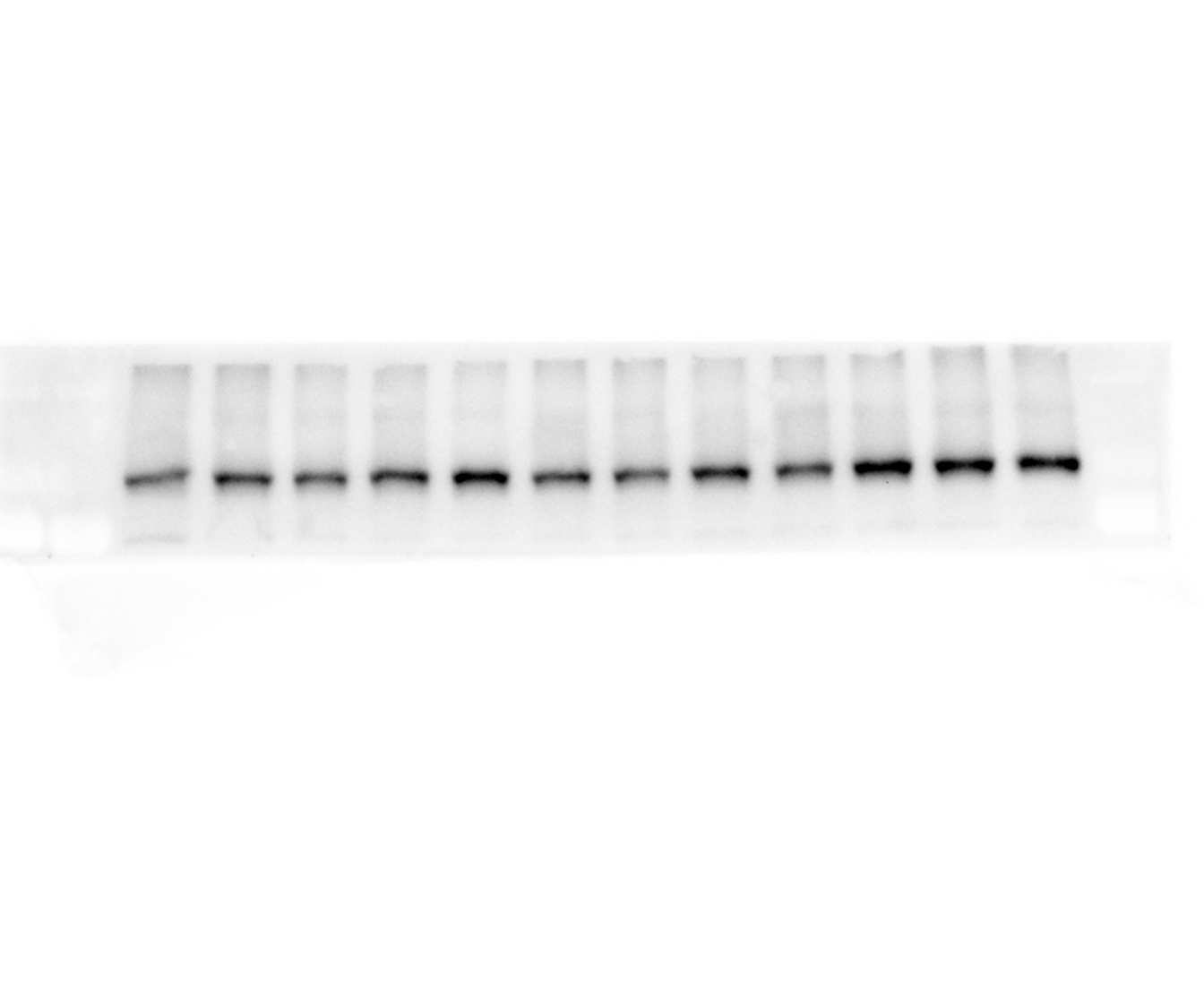

Supplement: Figure 6—source data 1. [file elife-69310-fig6-data1.zip › Figure 6-source data 1/BAV-TAA patient #2(figure 6f)/MFN1-001-1.Tif]

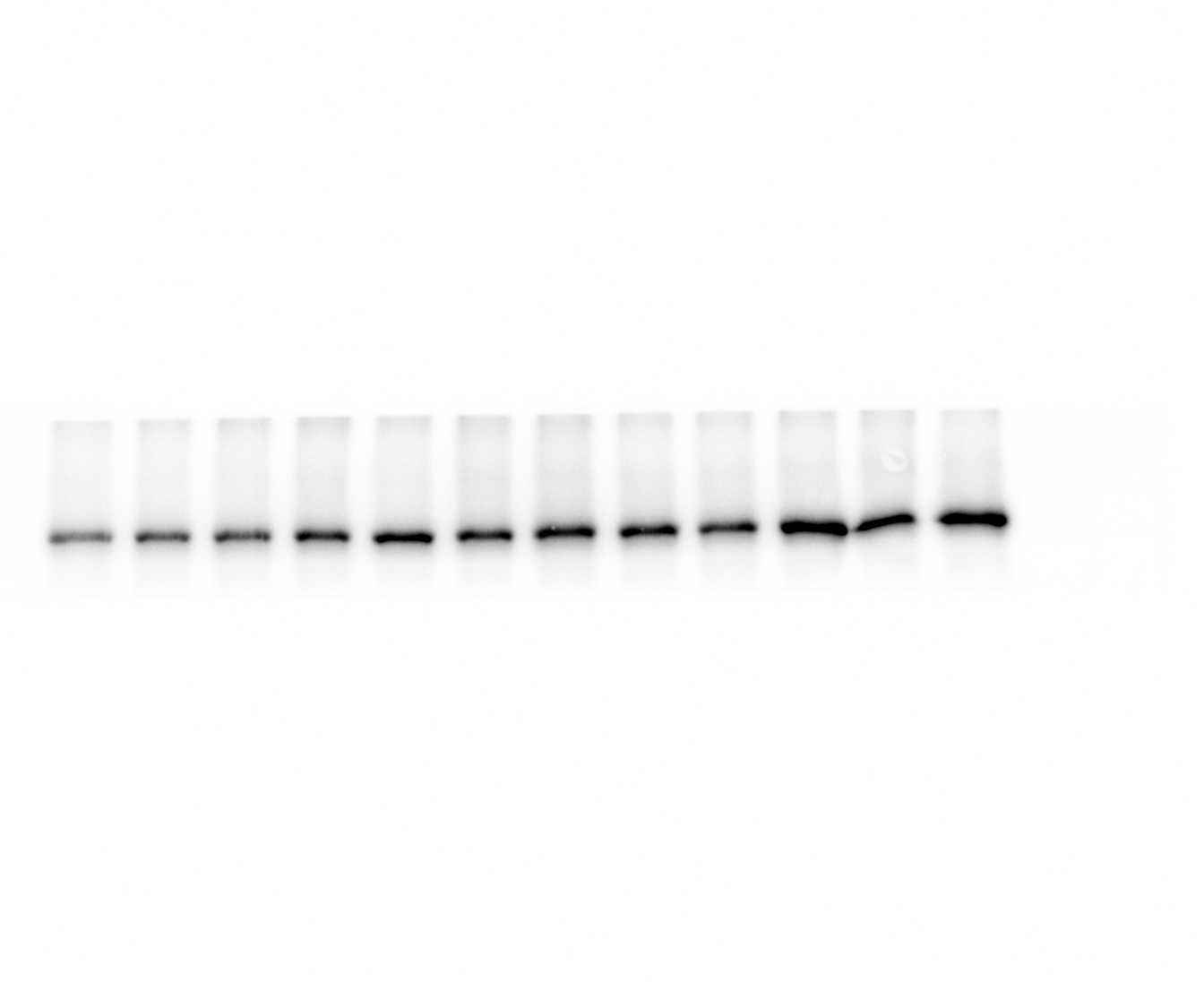

Supplement: Figure 6—source data 1. [file elife-69310-fig6-data1.zip › Figure 6-source data 1/BAV-TAA patient #2(figure 6f)/MFN2-002-3.Tif]

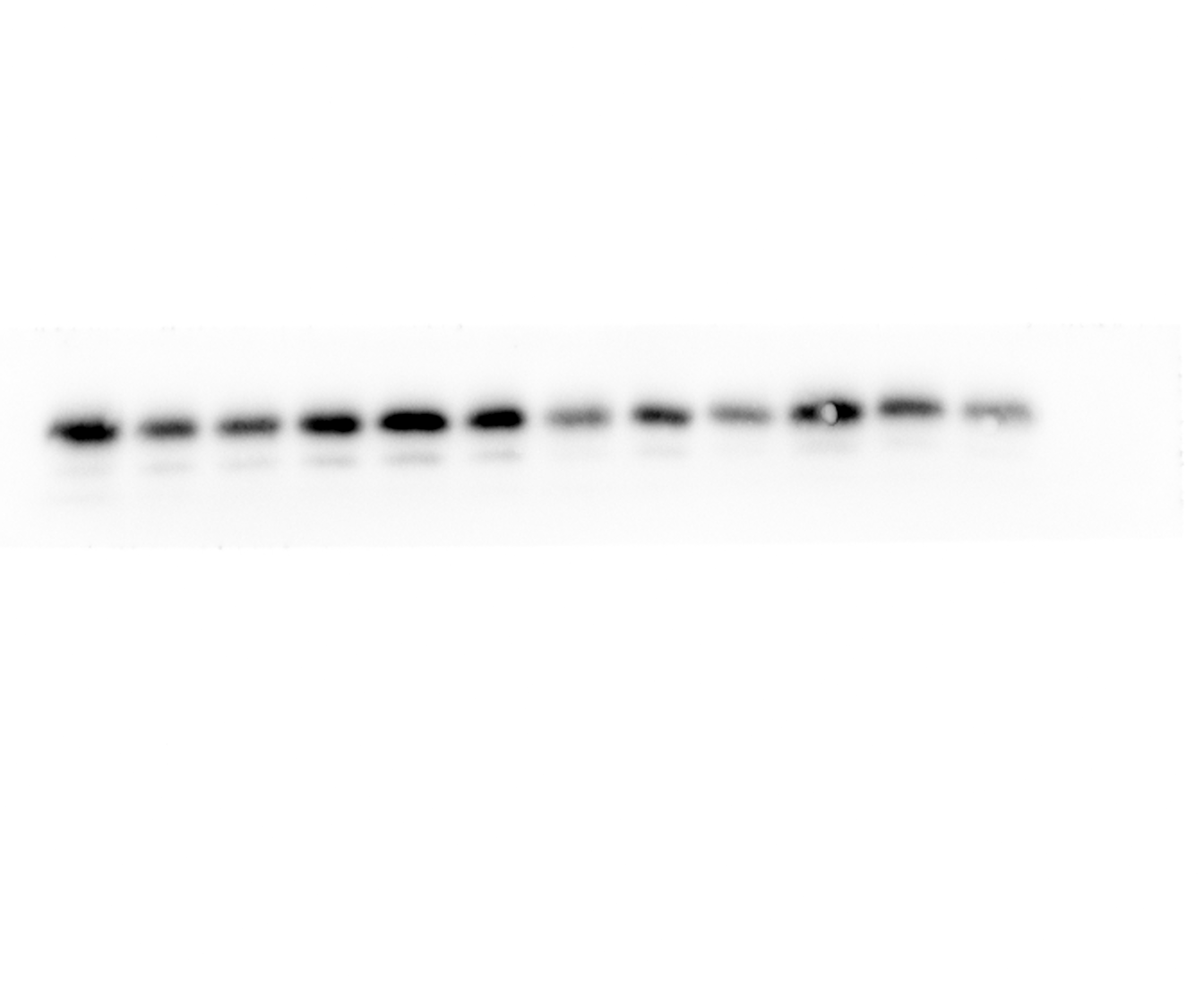

Supplement: Figure 6—source data 1. [file elife-69310-fig6-data1.zip › Figure 6-source data 1/BAV-TAA patient #2(figure 6f)/SM22-002-1.Tif]

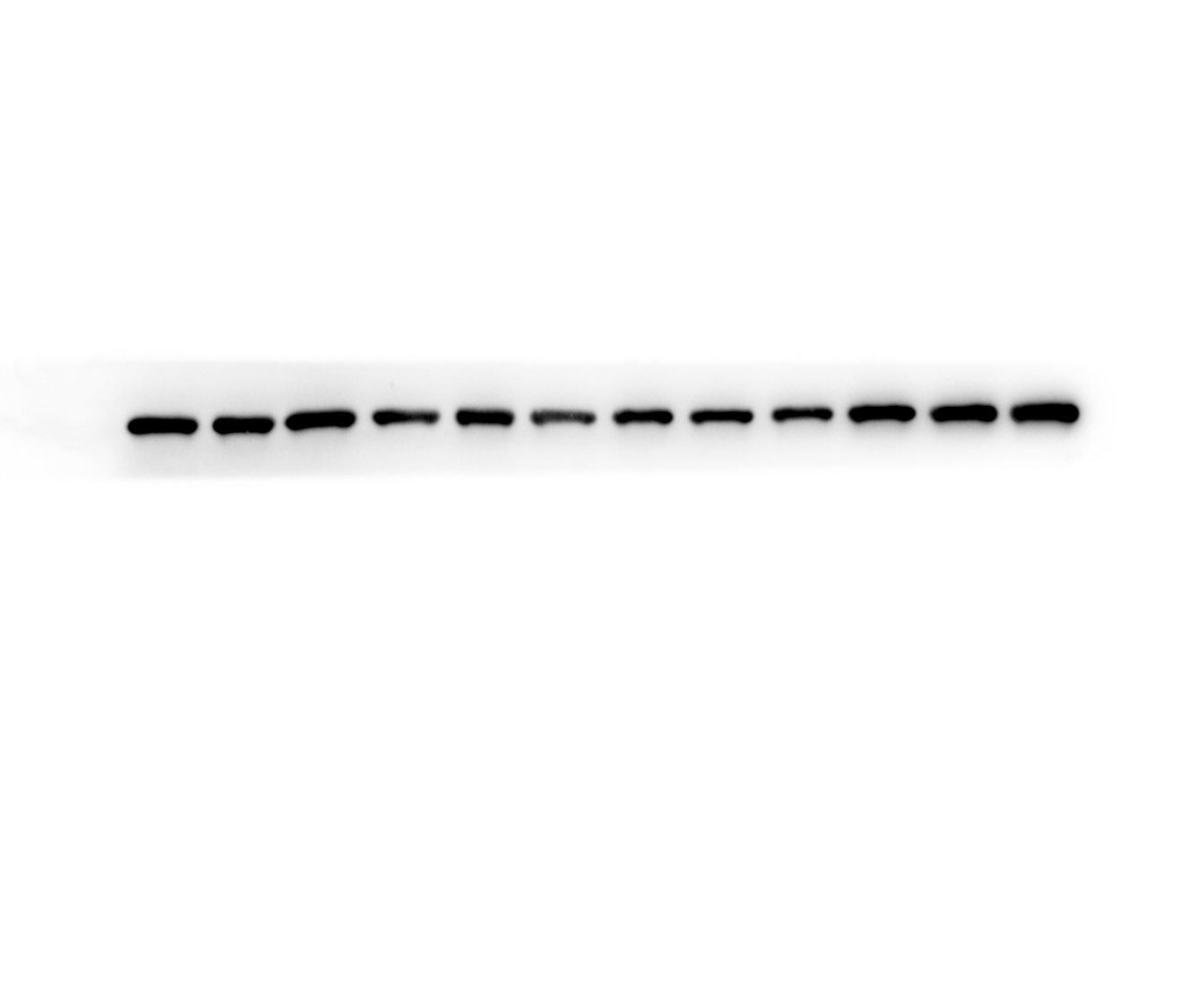

Supplement: Figure 6—source data 1. [file elife-69310-fig6-data1.zip › Figure 6-source data 1/BAV-TAA patient #3(figure 6g)/B-ACTIN-002-3.Tif]

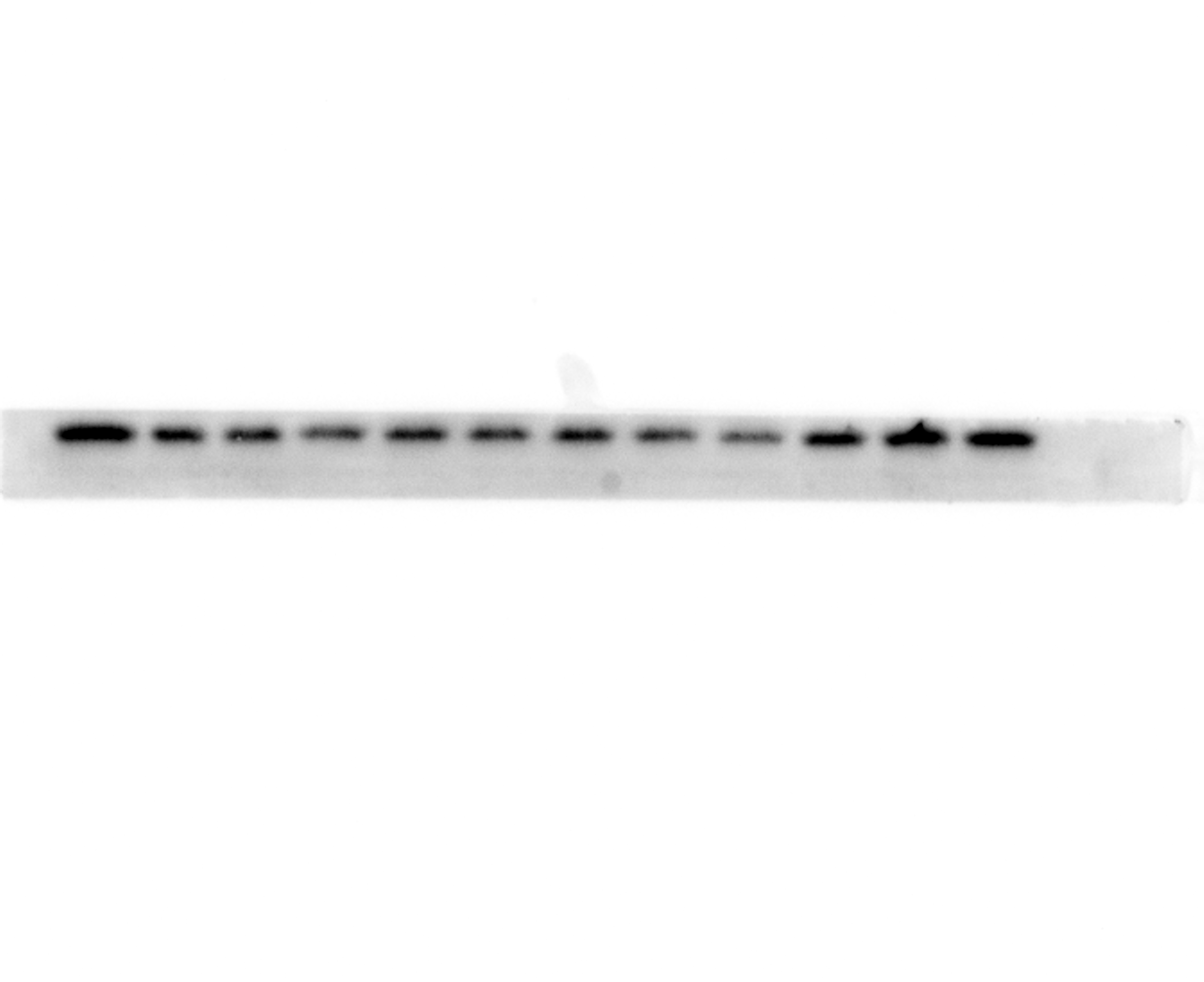

Supplement: Figure 6—source data 1. [file elife-69310-fig6-data1.zip › Figure 6-source data 1/BAV-TAA patient #3(figure 6g)/CNN1-001-1.Tif]

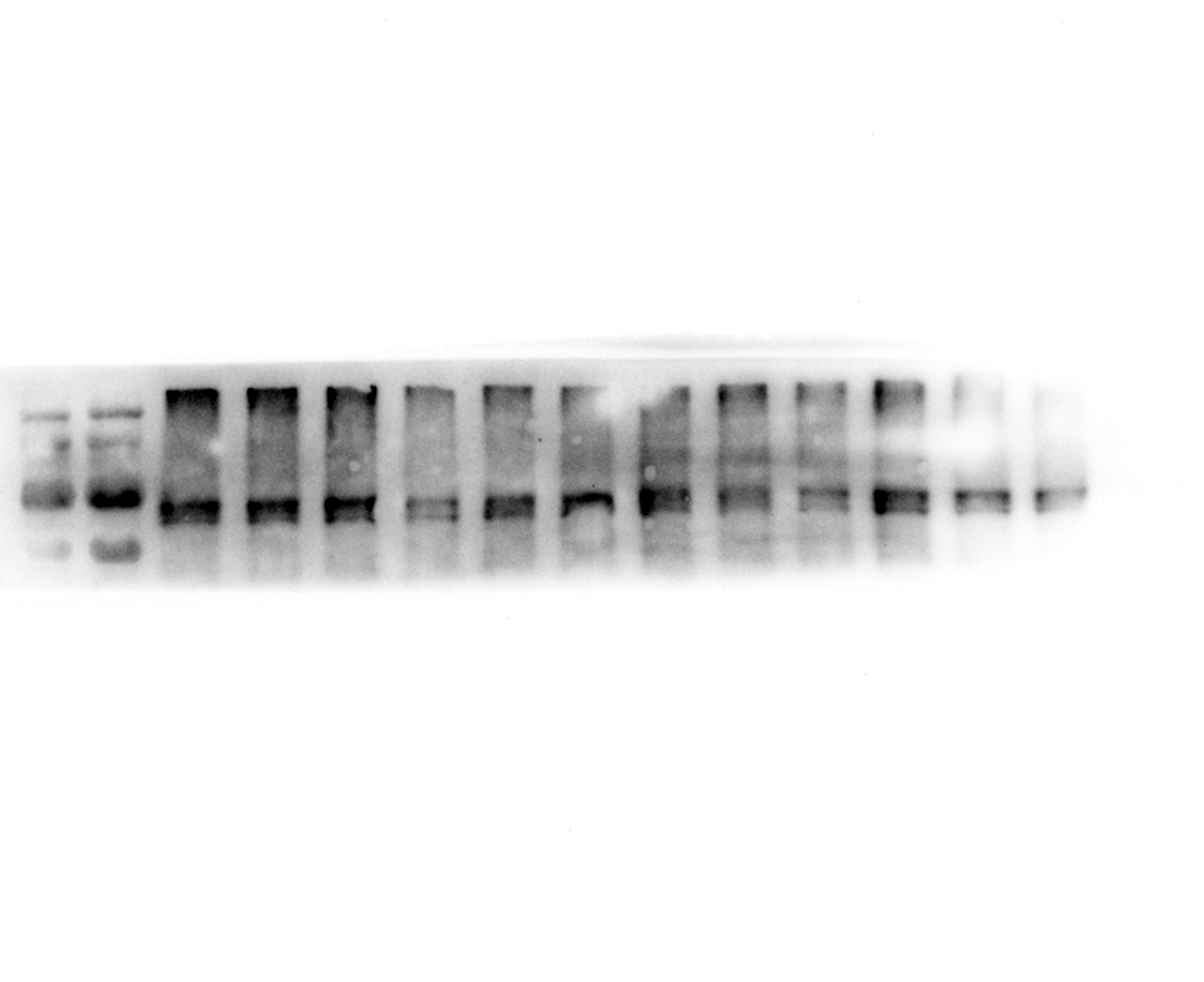

Supplement: Figure 6—source data 1. [file elife-69310-fig6-data1.zip › Figure 6-source data 1/BAV-TAA patient #3(figure 6g)/DRP-1-002-1.Tif]

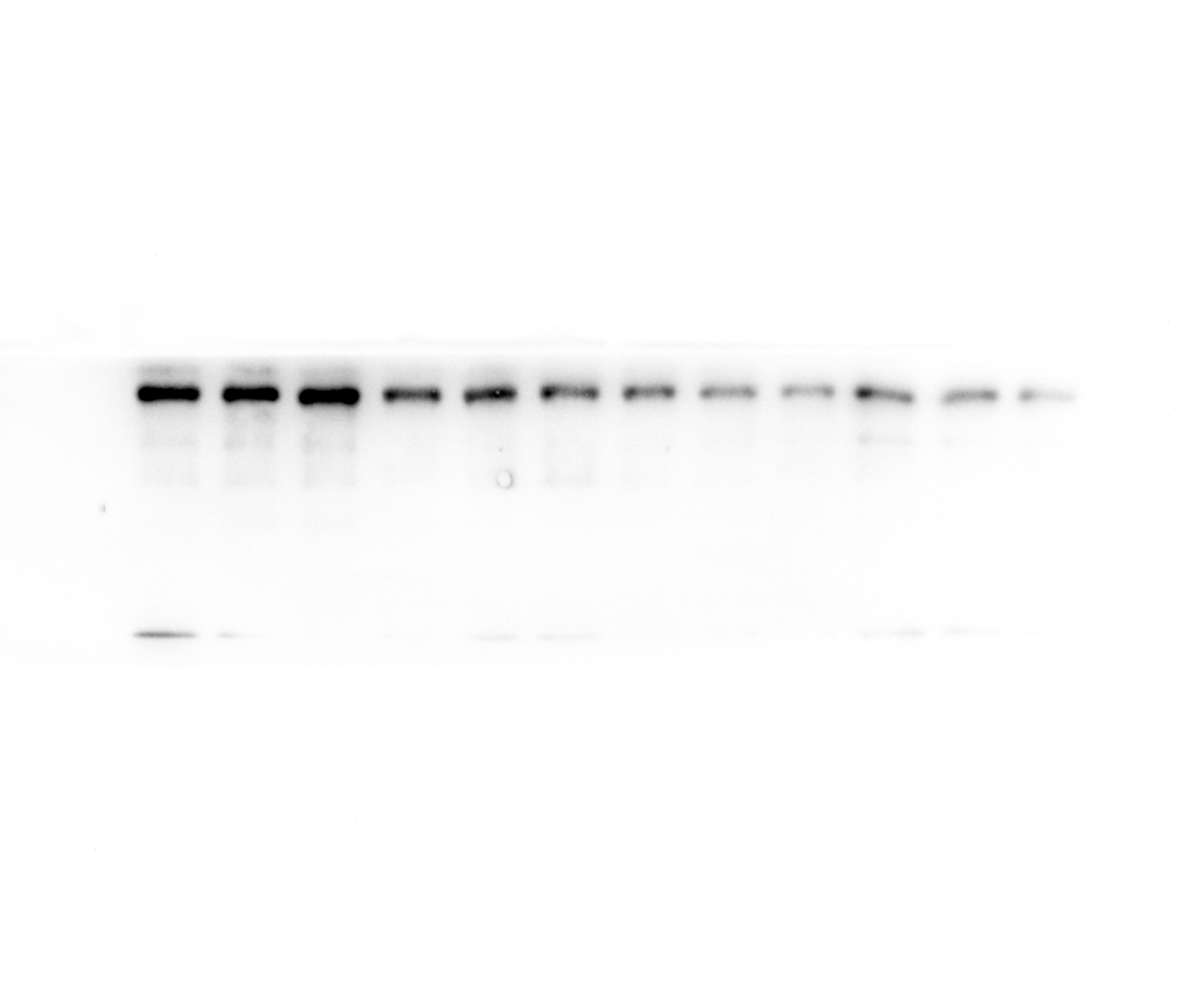

Supplement: Figure 6—source data 1. [file elife-69310-fig6-data1.zip › Figure 6-source data 1/BAV-TAA patient #3(figure 6g)/MFF-002-1.Tif]

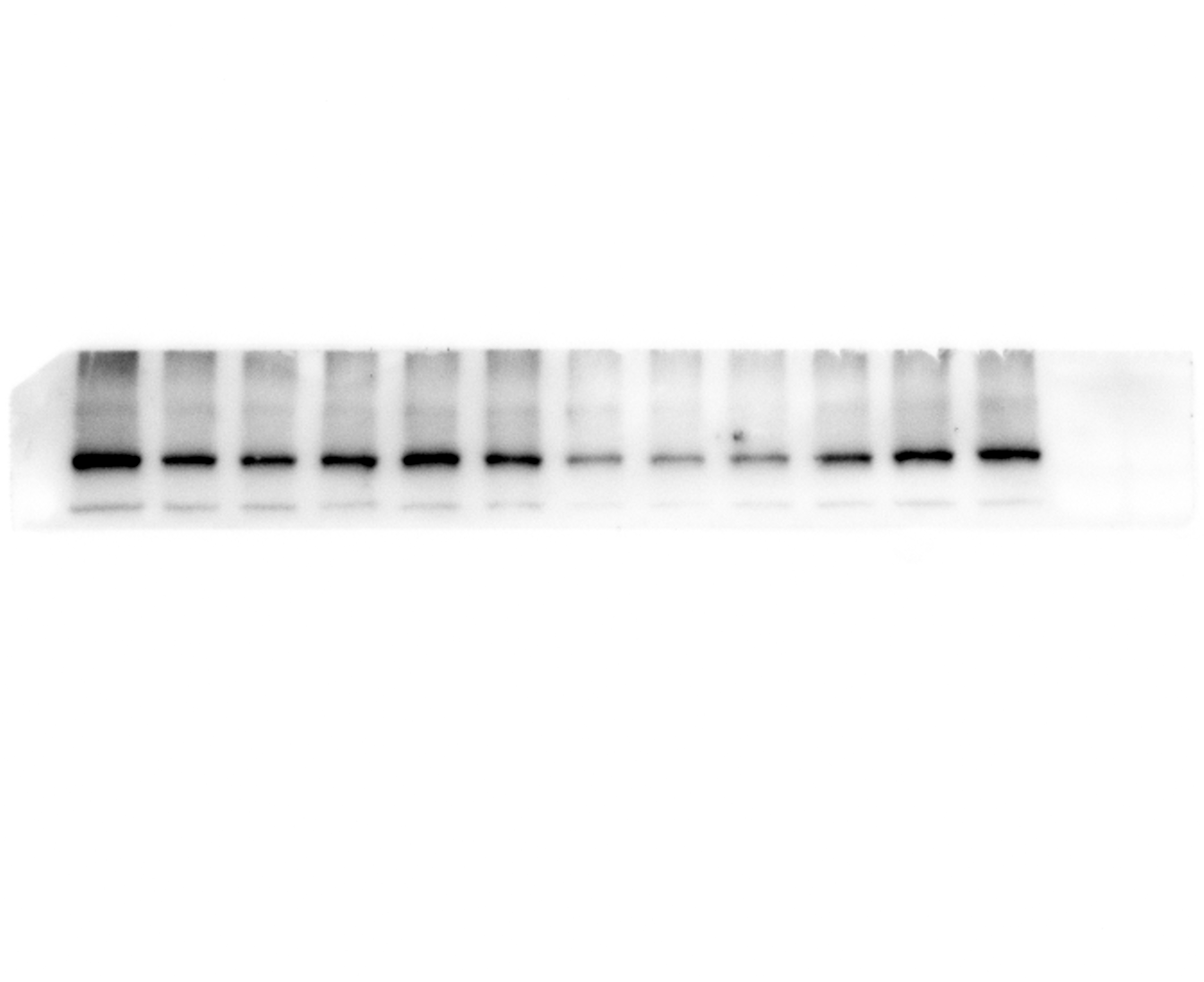

Supplement: Figure 6—source data 1. [file elife-69310-fig6-data1.zip › Figure 6-source data 1/BAV-TAA patient #3(figure 6g)/MFN1-001-1.Tif]

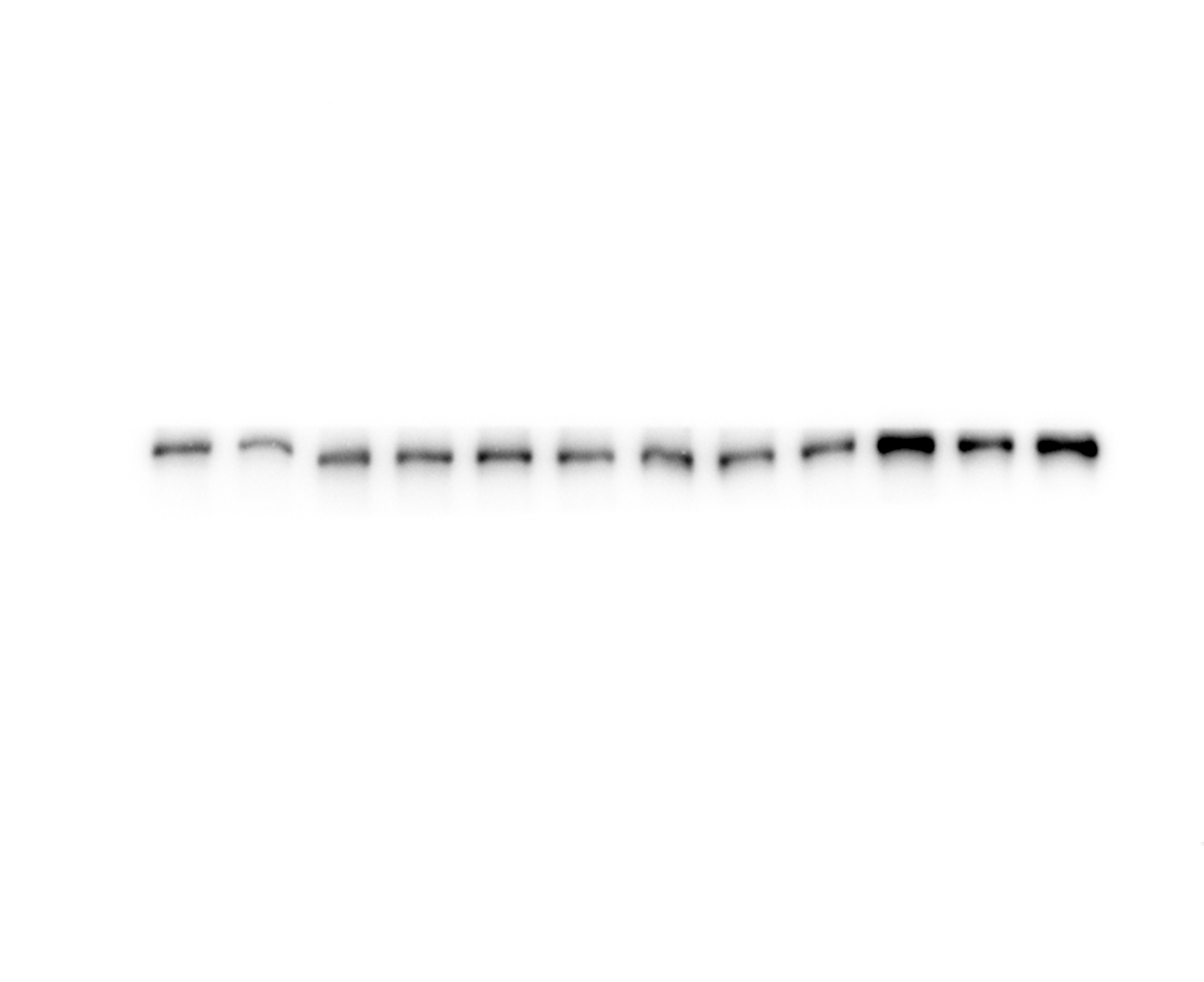

Supplement: Figure 6—source data 1. [file elife-69310-fig6-data1.zip › Figure 6-source data 1/BAV-TAA patient #3(figure 6g)/MFN2-001-1.Tif]

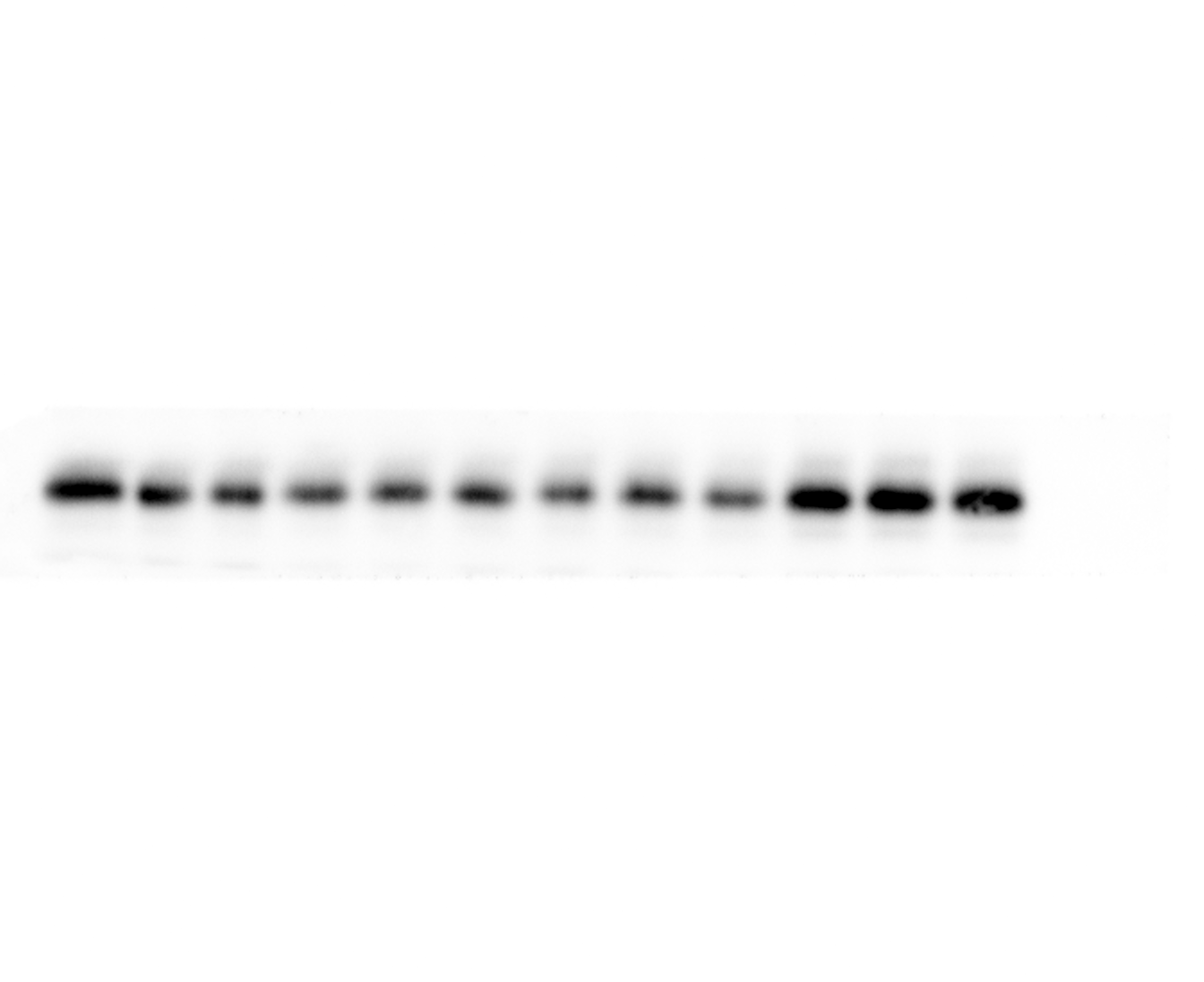

Supplement: Figure 6—source data 1. [file elife-69310-fig6-data1.zip › Figure 6-source data 1/BAV-TAA patient #3(figure 6g)/SM22-001-1.Tif]

**Fig. 6b (CRL1999 cell line)**

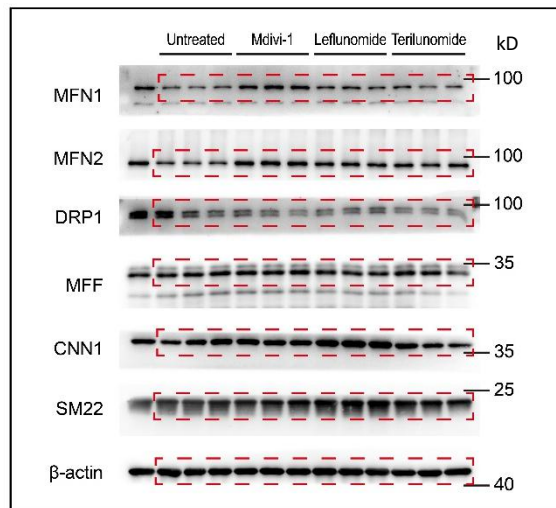

**Fig. 6c (pHAoSMC)**

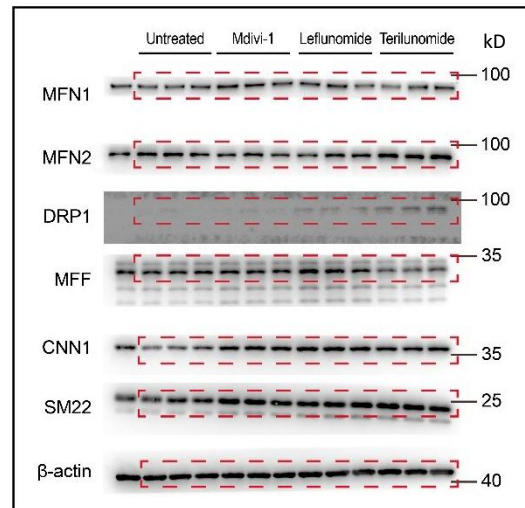

**Fig. 6d (ATCC-pHAoSMC)**

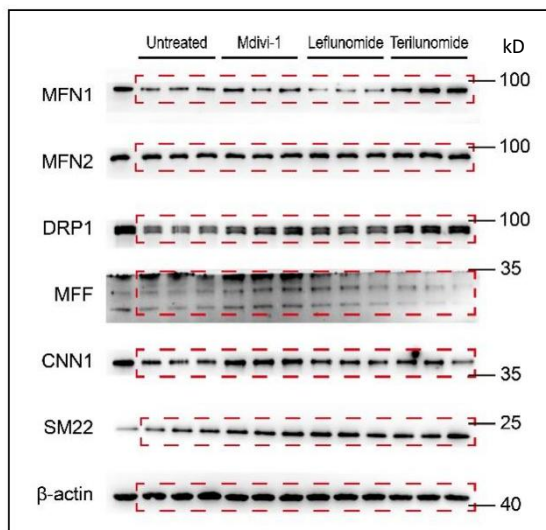

**Fig. 6e (B1-pHAoSMC)**

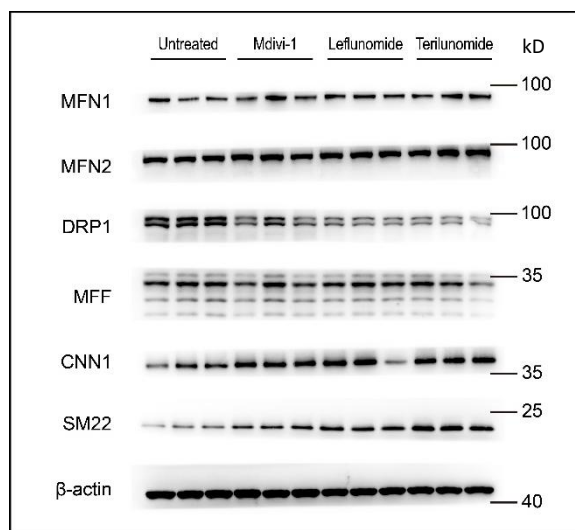

**Fig. 6f (B2-pHAoSMC)**

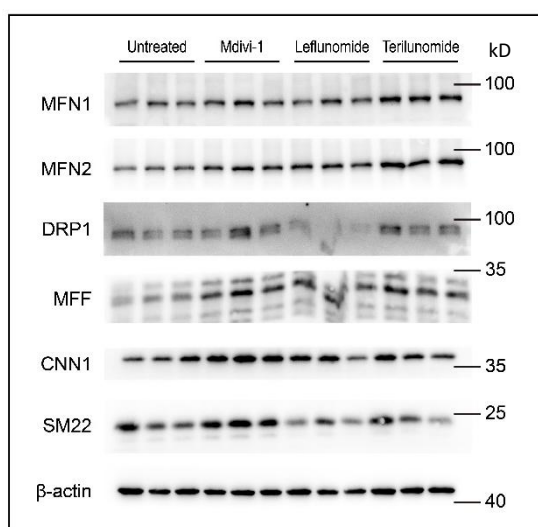

**Fig. 6g (B3-pHAoSMC)**

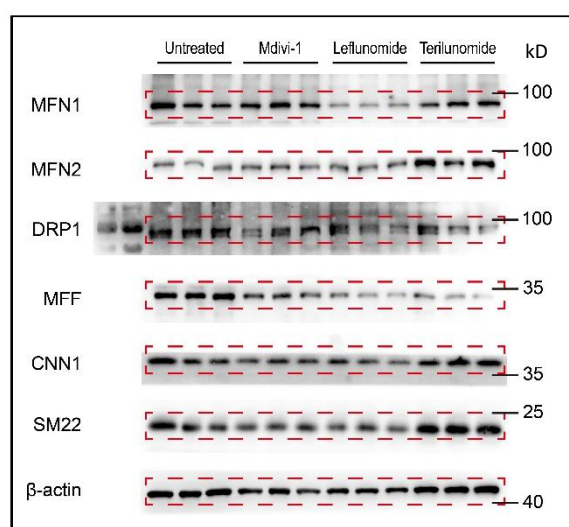

Supplement: Figure 6—source data 1. [file elife-69310-fig6-data1.zip › Figure 6-source data 1/labeled uncropped WB .pdf]

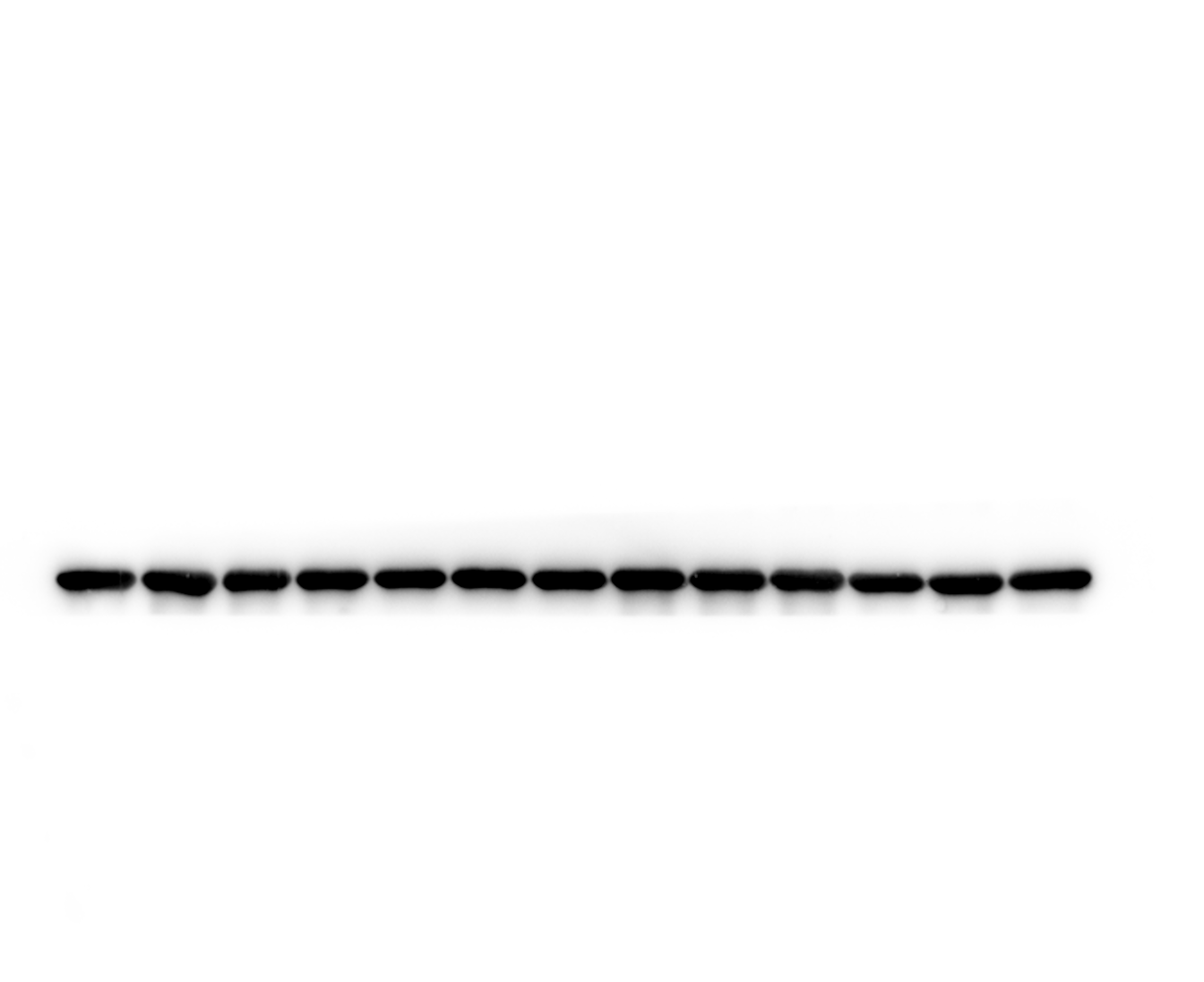

Supplement: Figure 6—source data 1. [file elife-69310-fig6-data1.zip › Figure 6-source data 1/nonaneurysmal patient #1(figure 6b)/B-ACTIN-003-1.Tif]

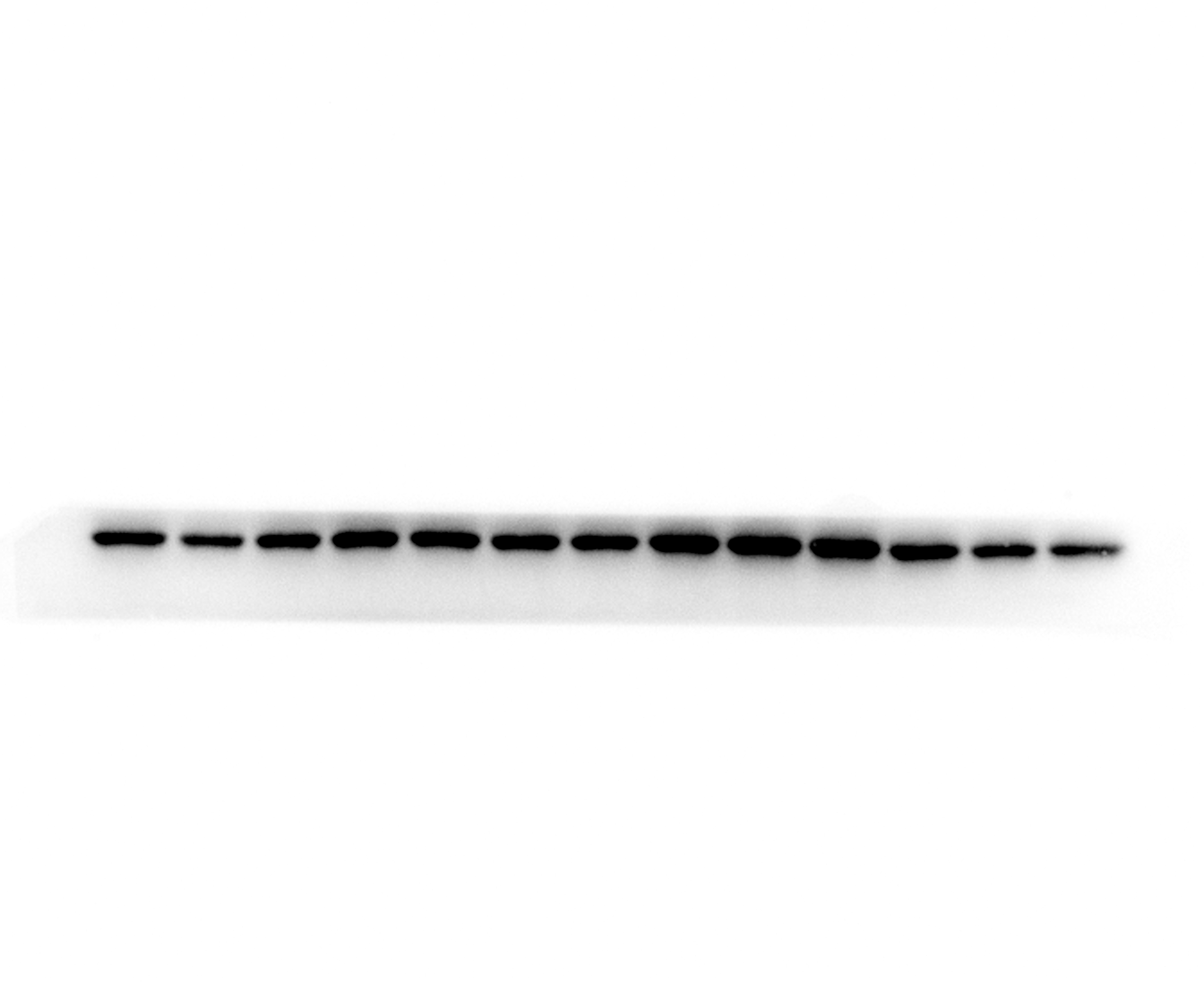

Supplement: Figure 6—source data 1. [file elife-69310-fig6-data1.zip › Figure 6-source data 1/nonaneurysmal patient #1(figure 6b)/CNN1-003-5.Tif]

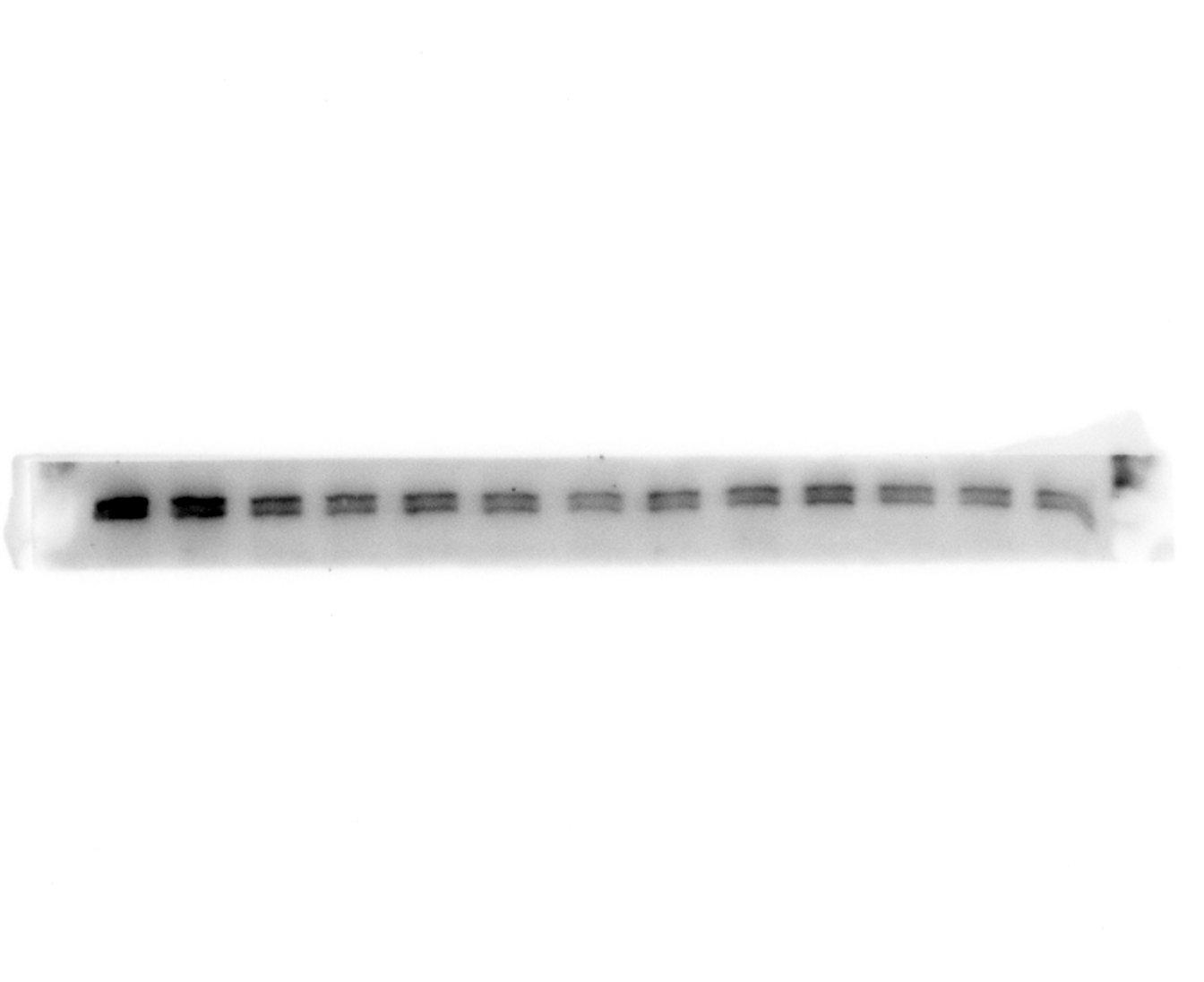

Supplement: Figure 6—source data 1. [file elife-69310-fig6-data1.zip › Figure 6-source data 1/nonaneurysmal patient #1(figure 6b)/DRP-1-001-1.Tif]

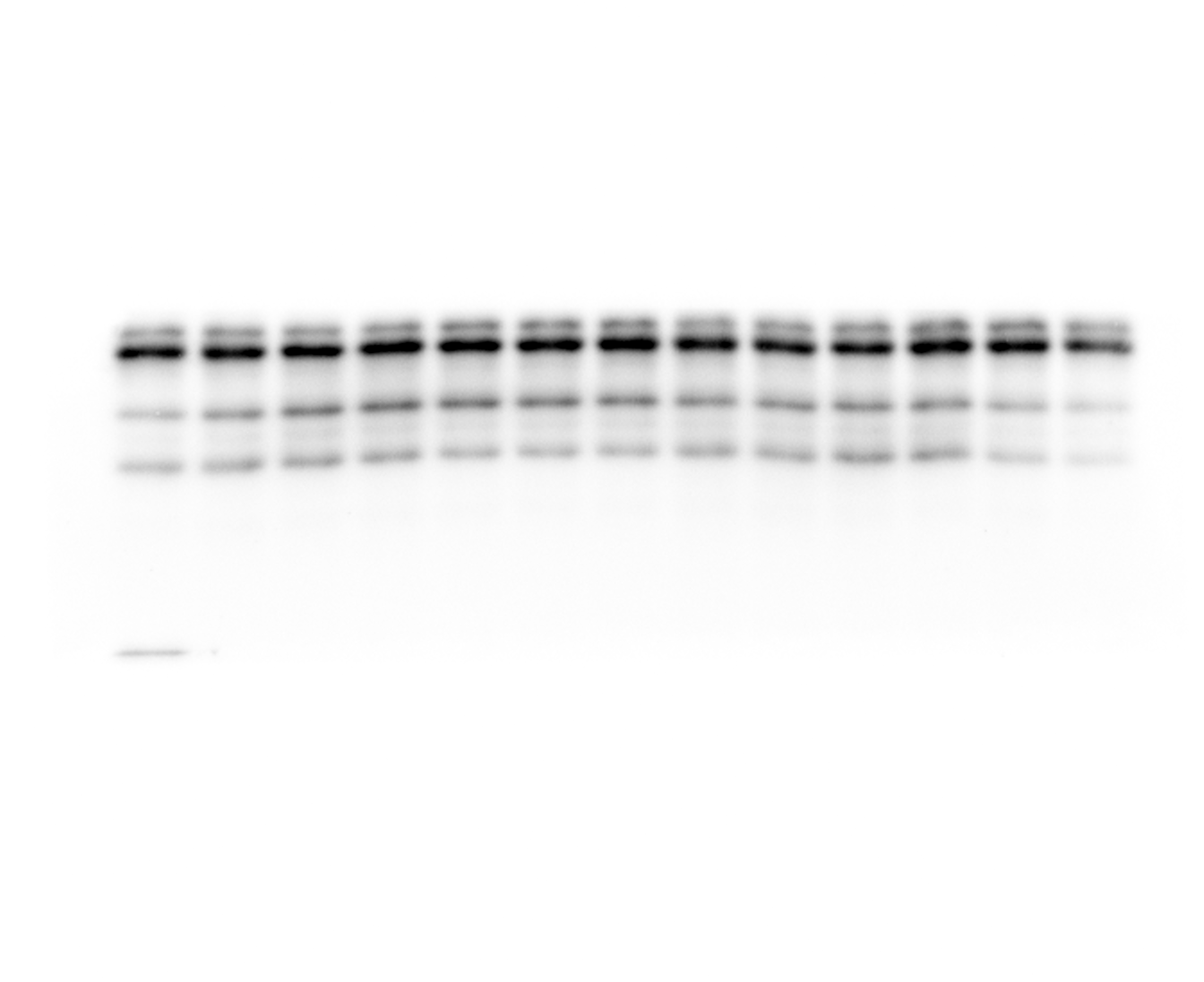

Supplement: Figure 6—source data 1. [file elife-69310-fig6-data1.zip › Figure 6-source data 1/nonaneurysmal patient #1(figure 6b)/MFF-004-1.Tif]

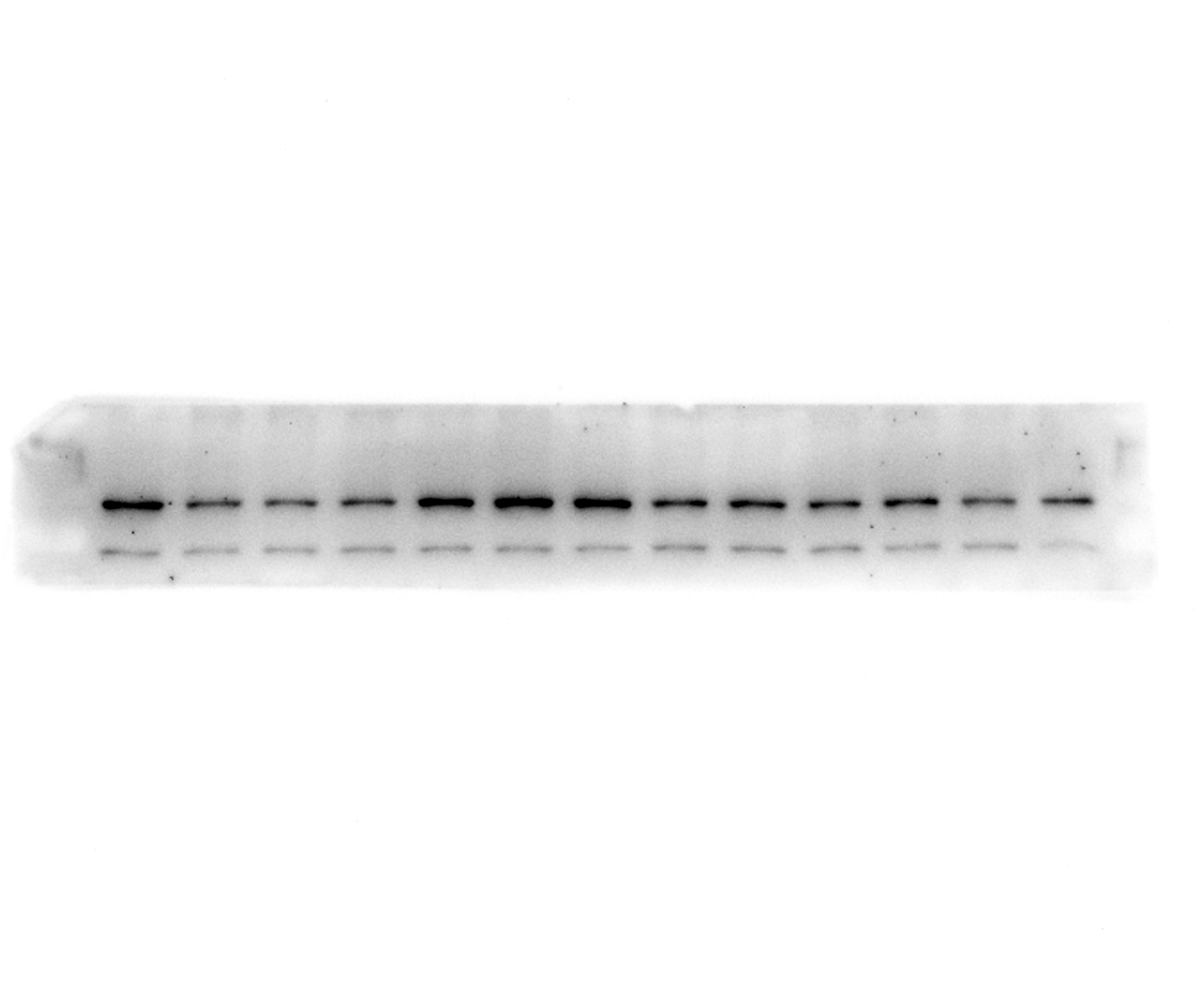

Supplement: Figure 6—source data 1. [file elife-69310-fig6-data1.zip › Figure 6-source data 1/nonaneurysmal patient #1(figure 6b)/MFN-1-004-1.Tif]

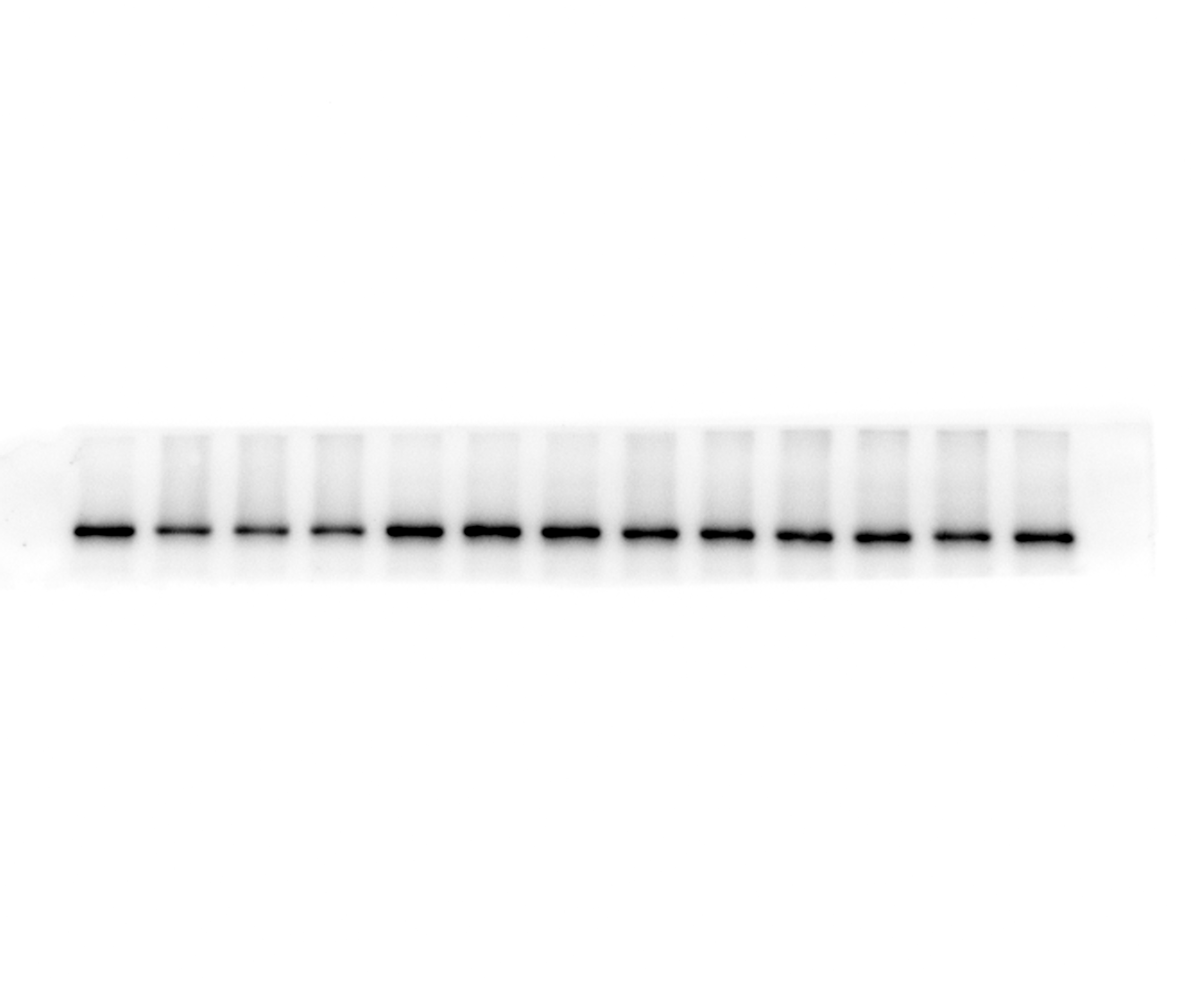

Supplement: Figure 6—source data 1. [file elife-69310-fig6-data1.zip › Figure 6-source data 1/nonaneurysmal patient #1(figure 6b)/MFN-2-003-1.Tif]

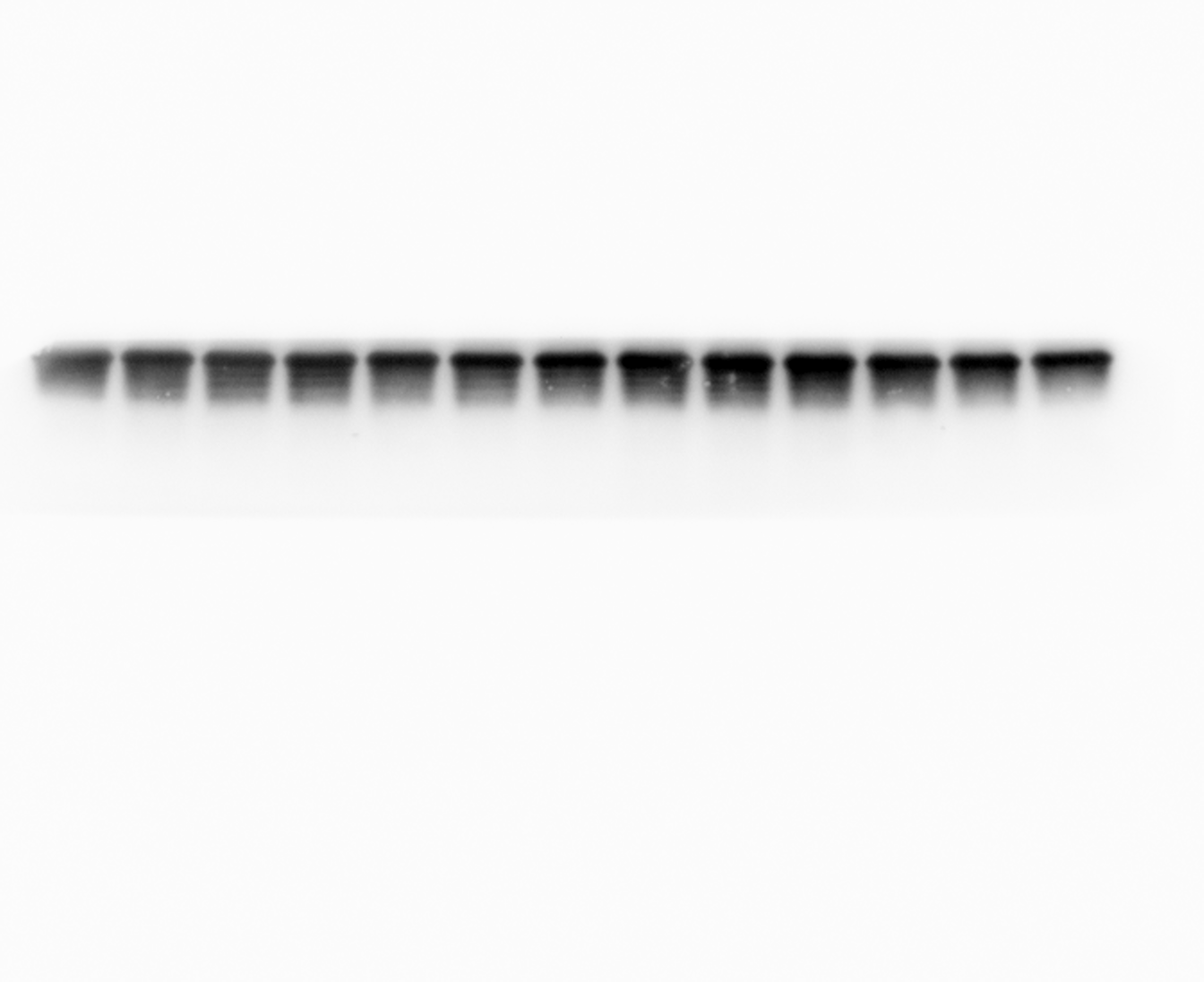

Supplement: Figure 6—source data 1. [file elife-69310-fig6-data1.zip › Figure 6-source data 1/nonaneurysmal patient #1(figure 6b)/SM22-001-7.Tif]

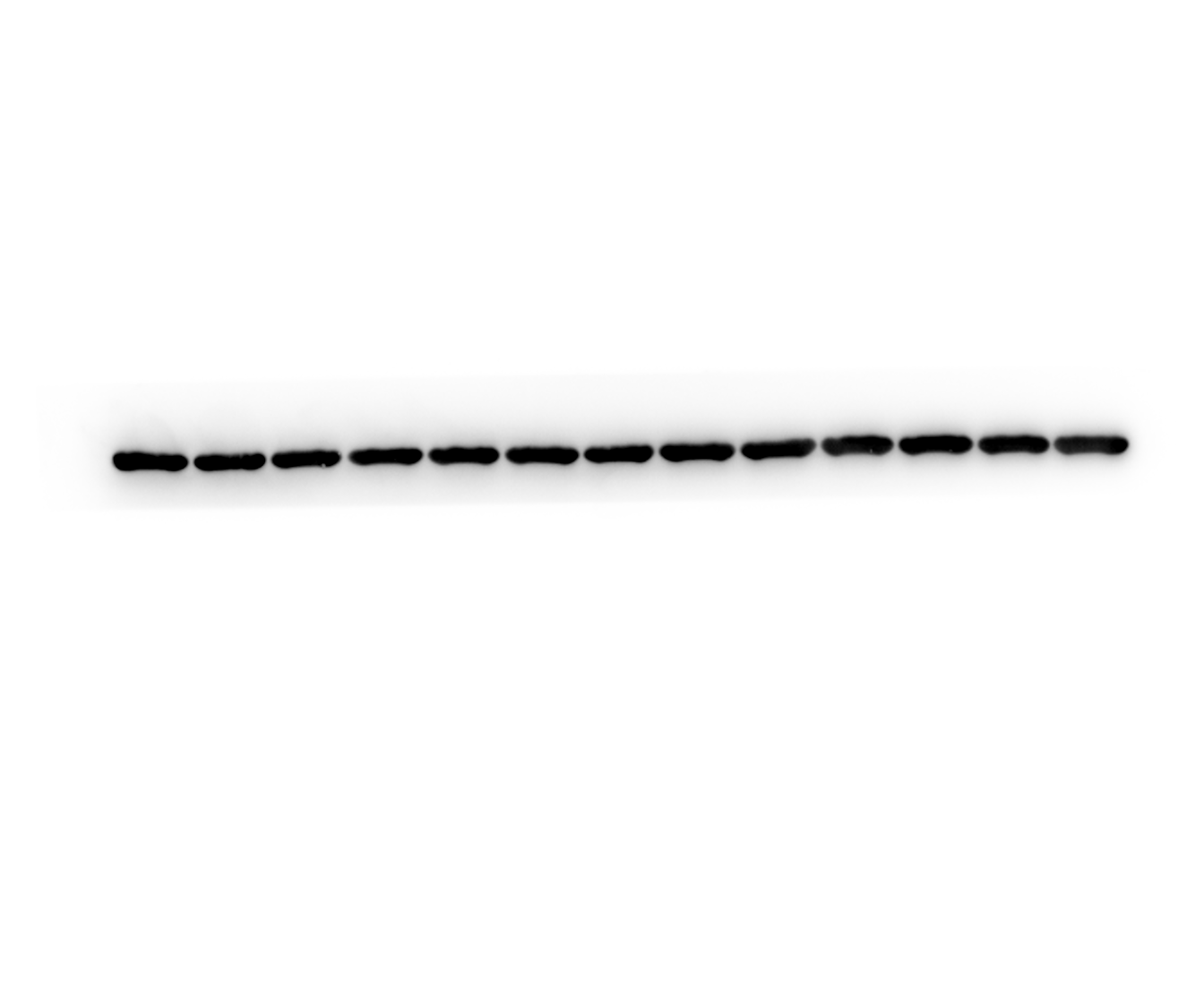

Supplement: Figure 6—source data 1. [file elife-69310-fig6-data1.zip › Figure 6-source data 1/nonaneurysmal patient #2(figure 6c)/B-ACTIN-001-3.Tif]

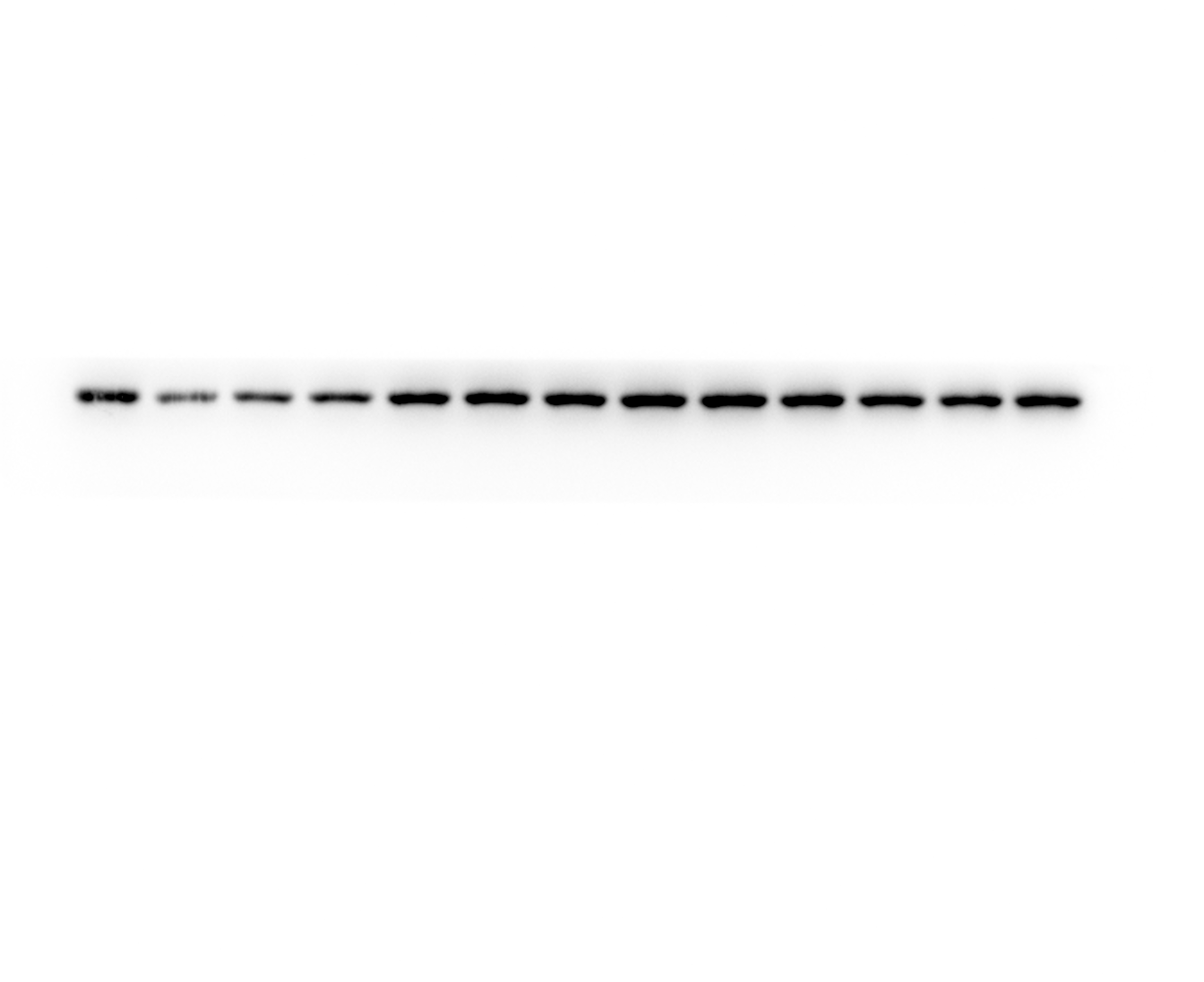

Supplement: Figure 6—source data 1. [file elife-69310-fig6-data1.zip › Figure 6-source data 1/nonaneurysmal patient #2(figure 6c)/CNN1-001-3.Tif]

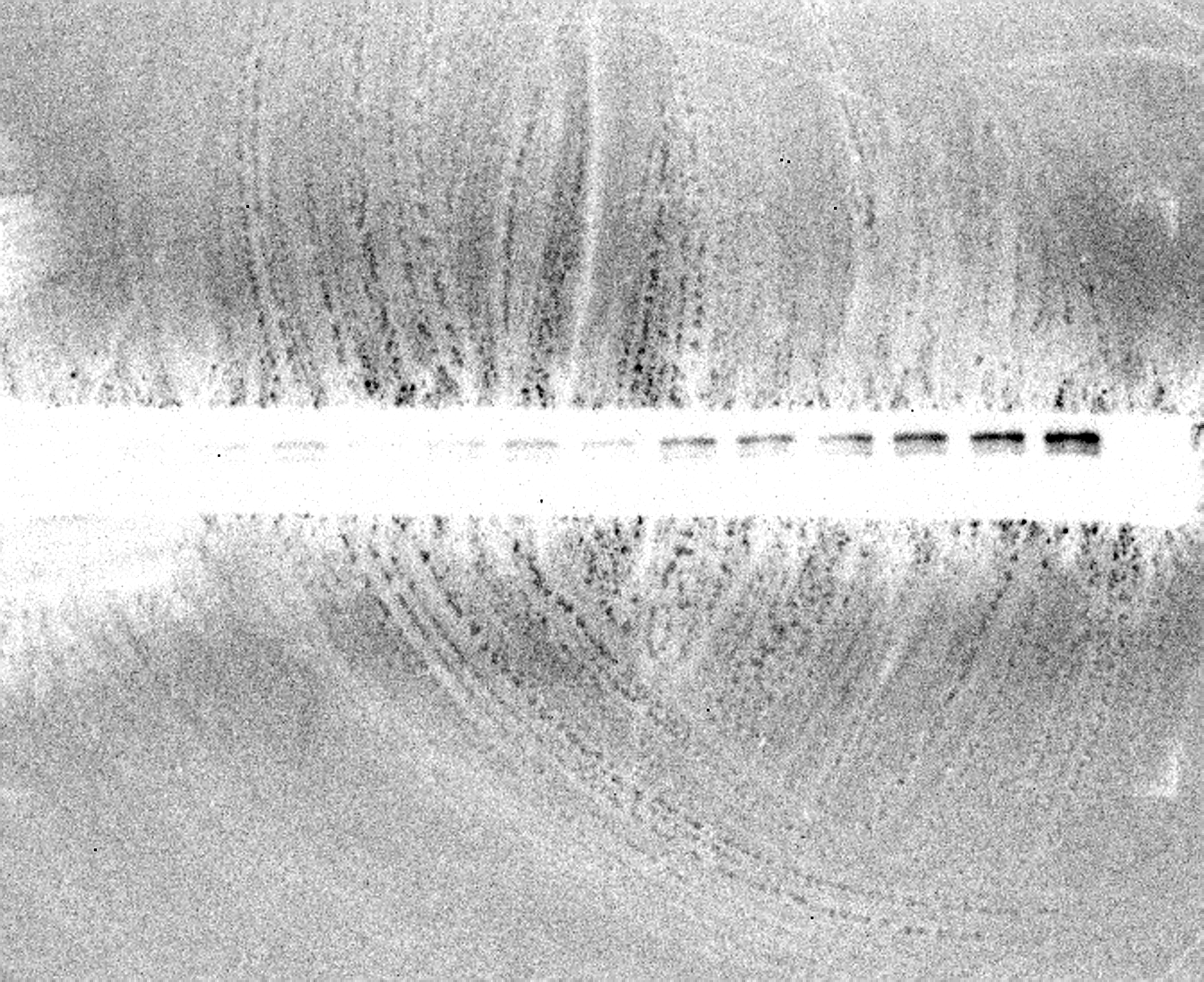

Supplement: Figure 6—source data 1. [file elife-69310-fig6-data1.zip › Figure 6-source data 1/nonaneurysmal patient #2(figure 6c)/DRP-1-002-1.Tif]

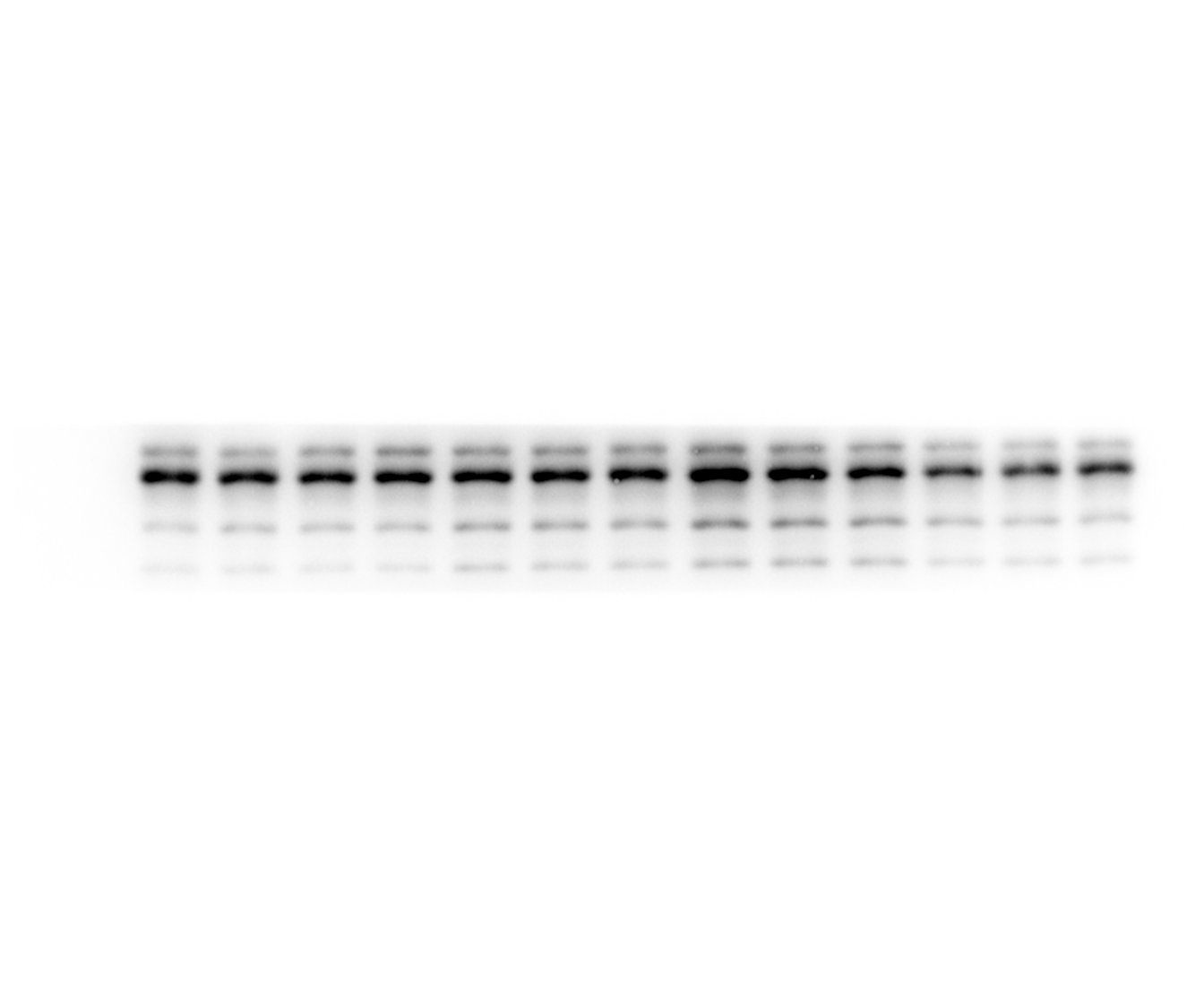

Supplement: Figure 6—source data 1. [file elife-69310-fig6-data1.zip › Figure 6-source data 1/nonaneurysmal patient #2(figure 6c)/MFF-002-3.Tif]

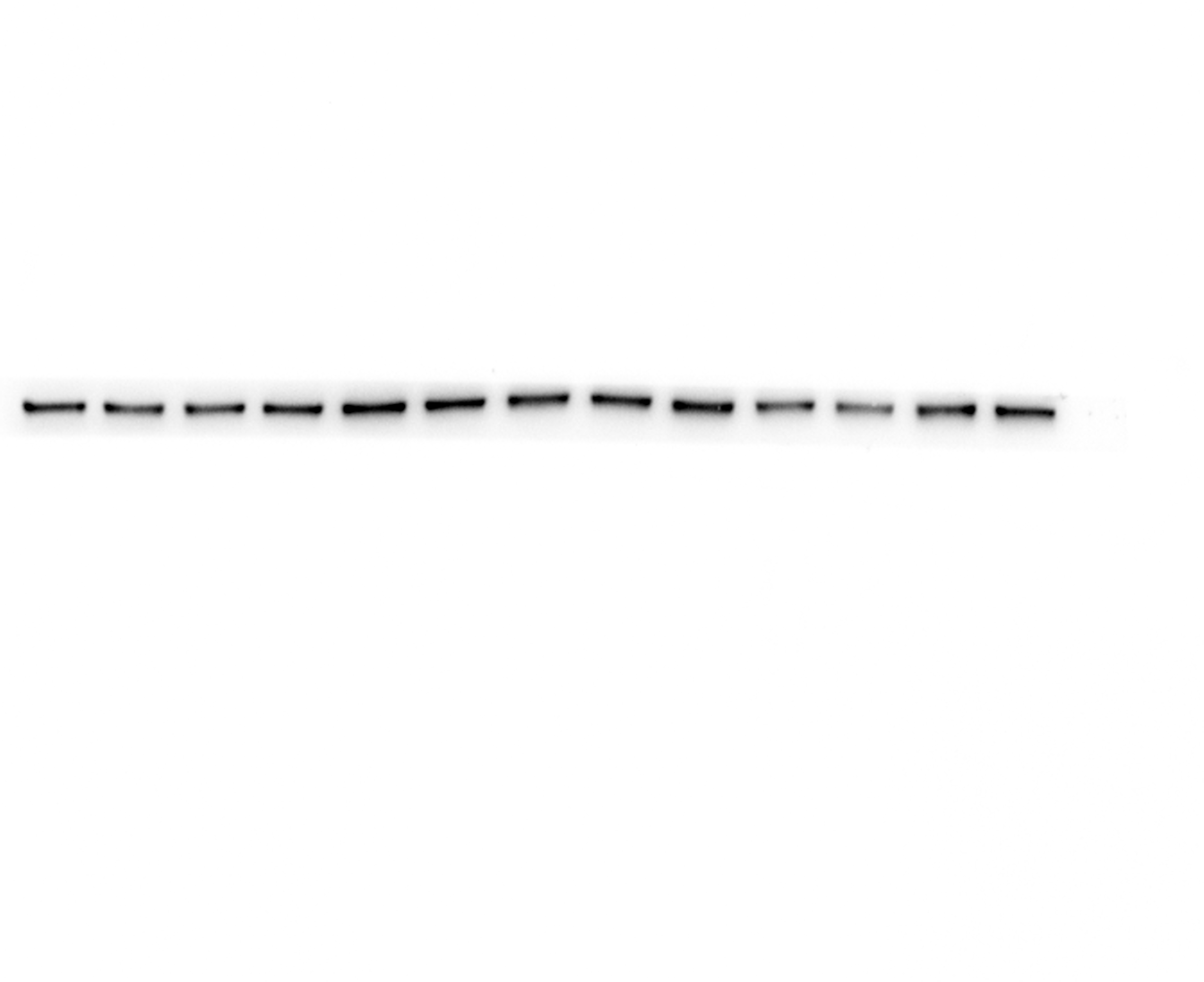

Supplement: Figure 6—source data 1. [file elife-69310-fig6-data1.zip › Figure 6-source data 1/nonaneurysmal patient #2(figure 6c)/MFN-1-004-1.Tif]

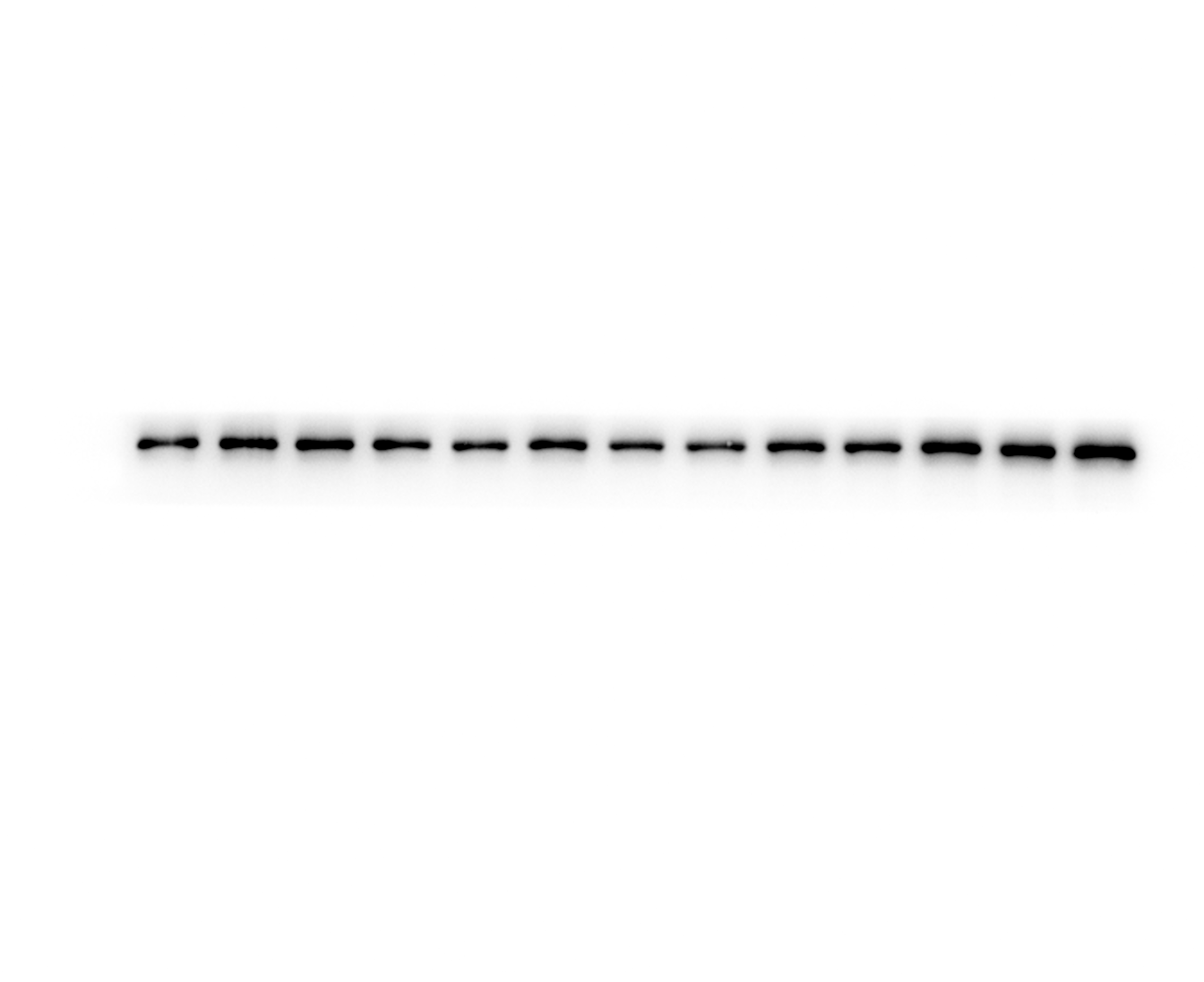

Supplement: Figure 6—source data 1. [file elife-69310-fig6-data1.zip › Figure 6-source data 1/nonaneurysmal patient #2(figure 6c)/MFN-2-001-1.Tif]

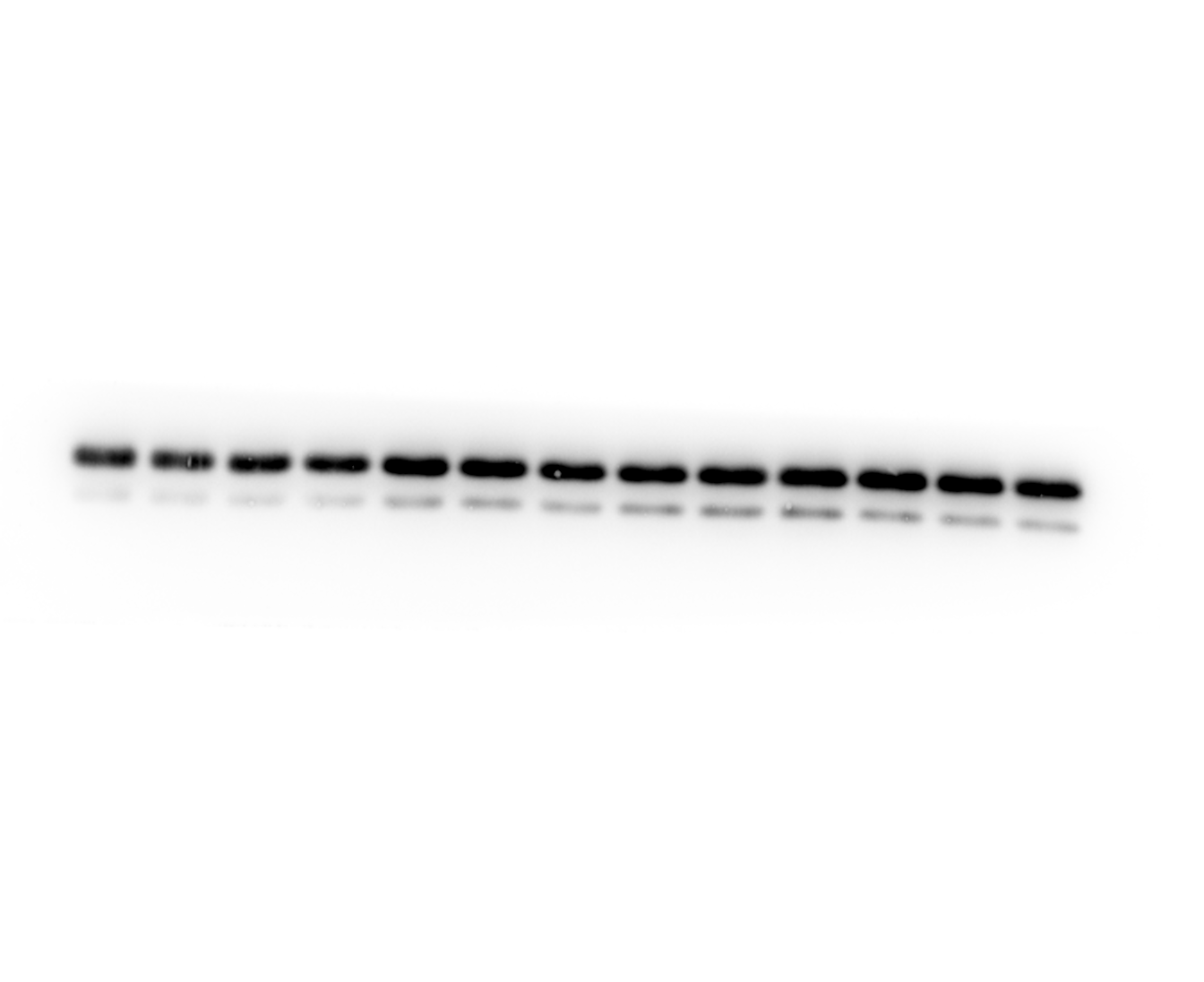

Supplement: Figure 6—source data 1. [file elife-69310-fig6-data1.zip › Figure 6-source data 1/nonaneurysmal patient #2(figure 6c)/SM22-001-1.Tif]

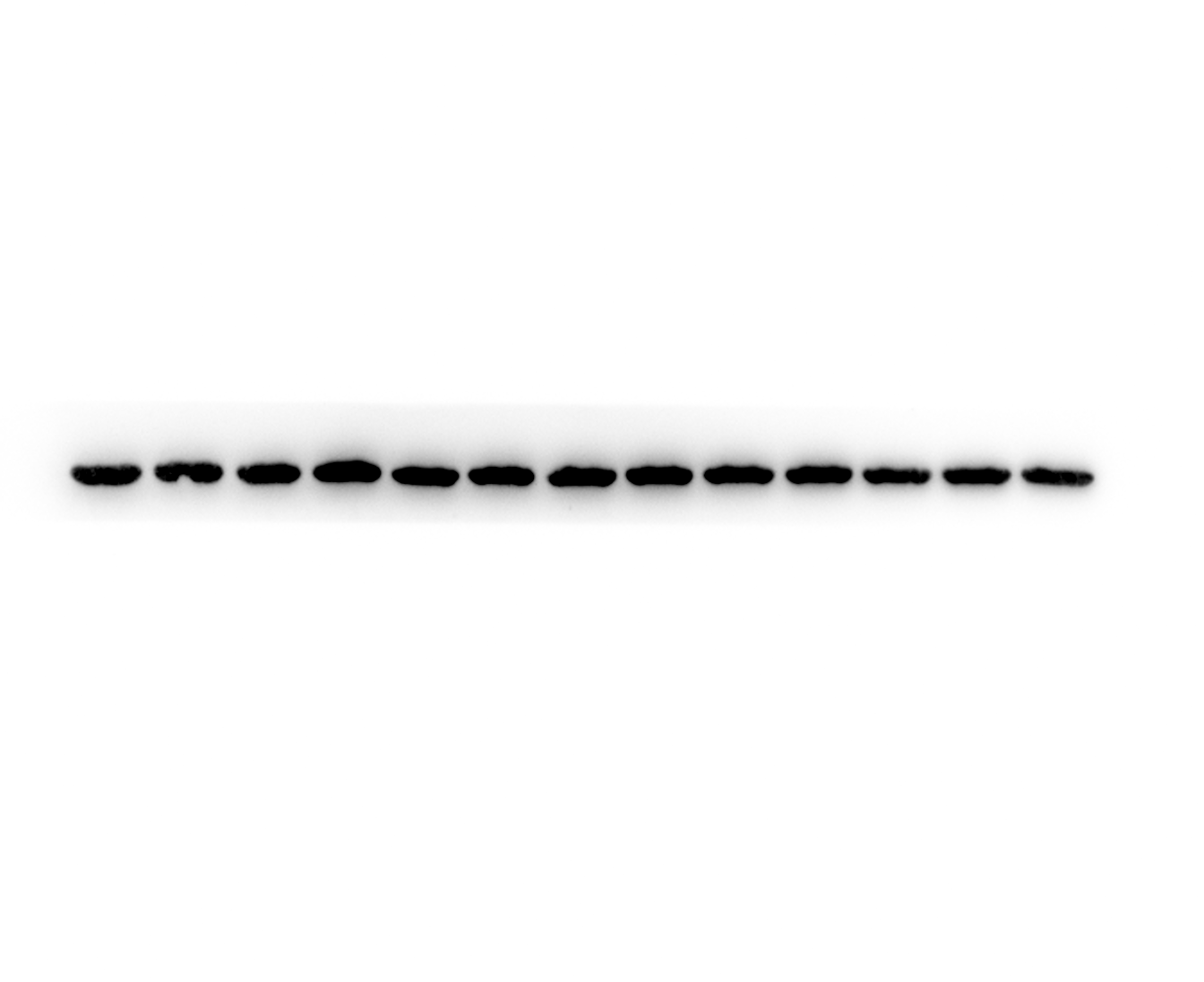

Supplement: Figure 6—source data 1. [file elife-69310-fig6-data1.zip › Figure 6-source data 1/nonaneurysmal patient #3(figure 6d)/B-ACTIN-001-1.Tif]

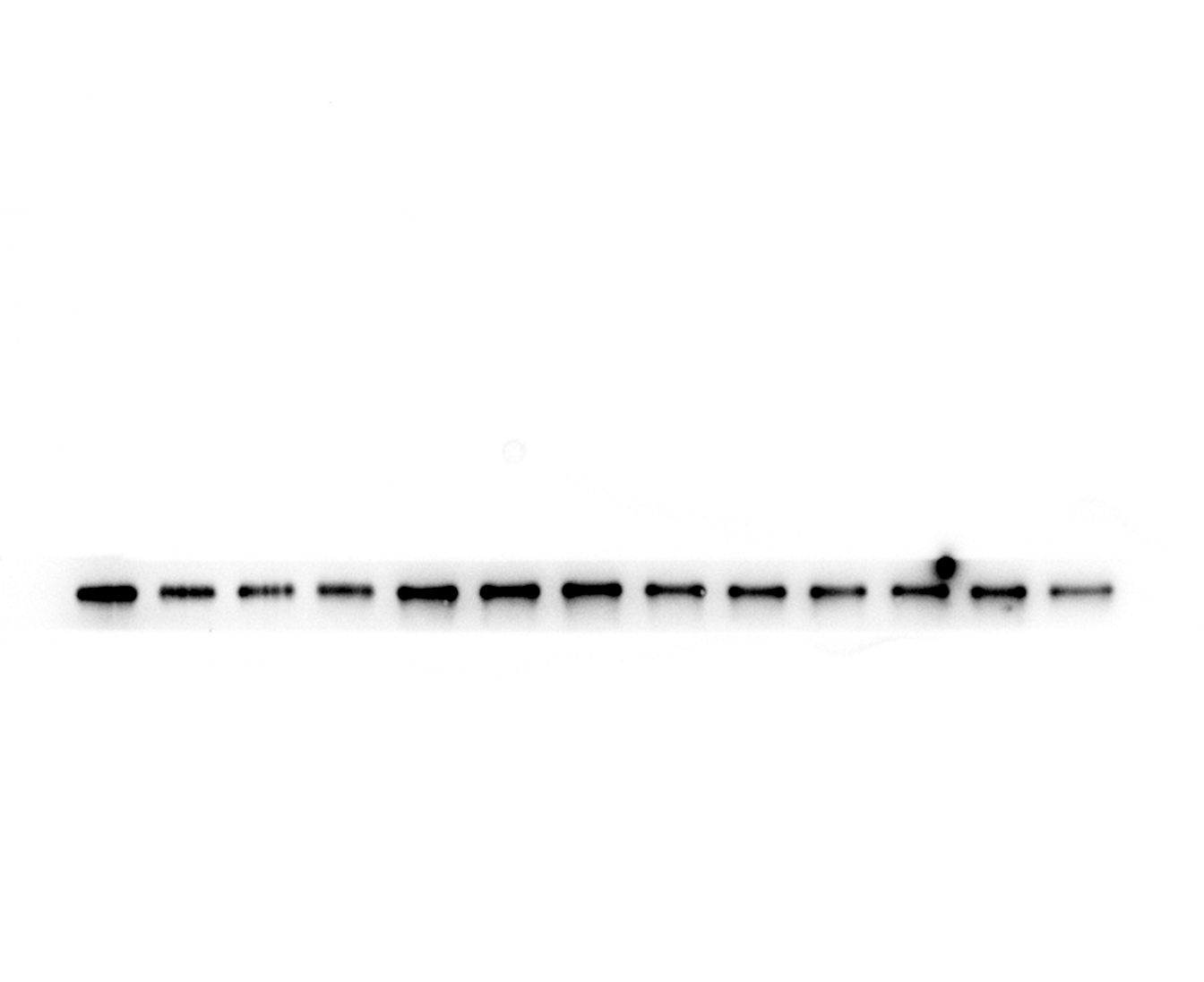

Supplement: Figure 6—source data 1. [file elife-69310-fig6-data1.zip › Figure 6-source data 1/nonaneurysmal patient #3(figure 6d)/CNN1-003-3.Tif]

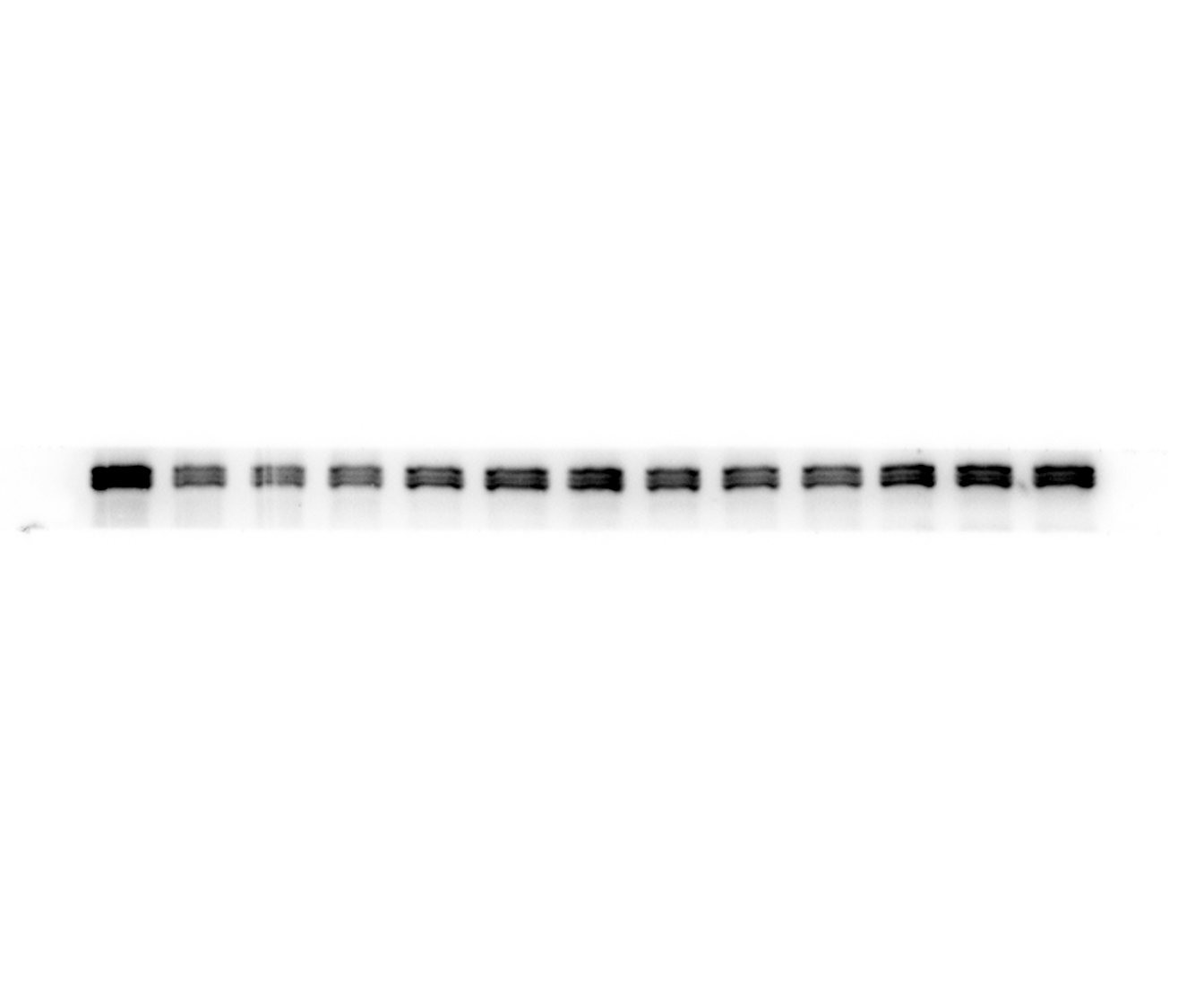

Supplement: Figure 6—source data 1. [file elife-69310-fig6-data1.zip › Figure 6-source data 1/nonaneurysmal patient #3(figure 6d)/DRP-1-002-1.Tif]

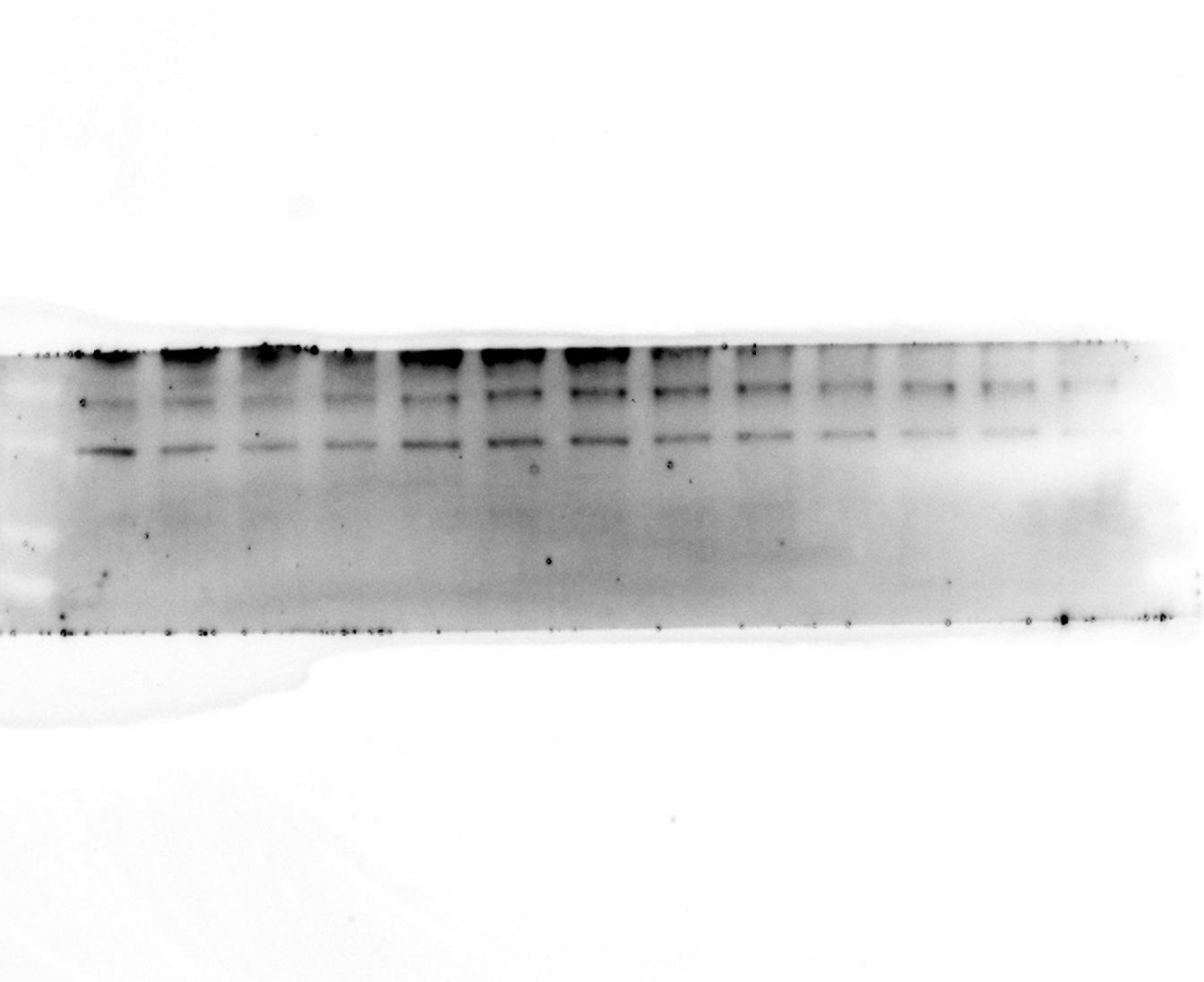

Supplement: Figure 6—source data 1. [file elife-69310-fig6-data1.zip › Figure 6-source data 1/nonaneurysmal patient #3(figure 6d)/MFF-004-1.Tif]

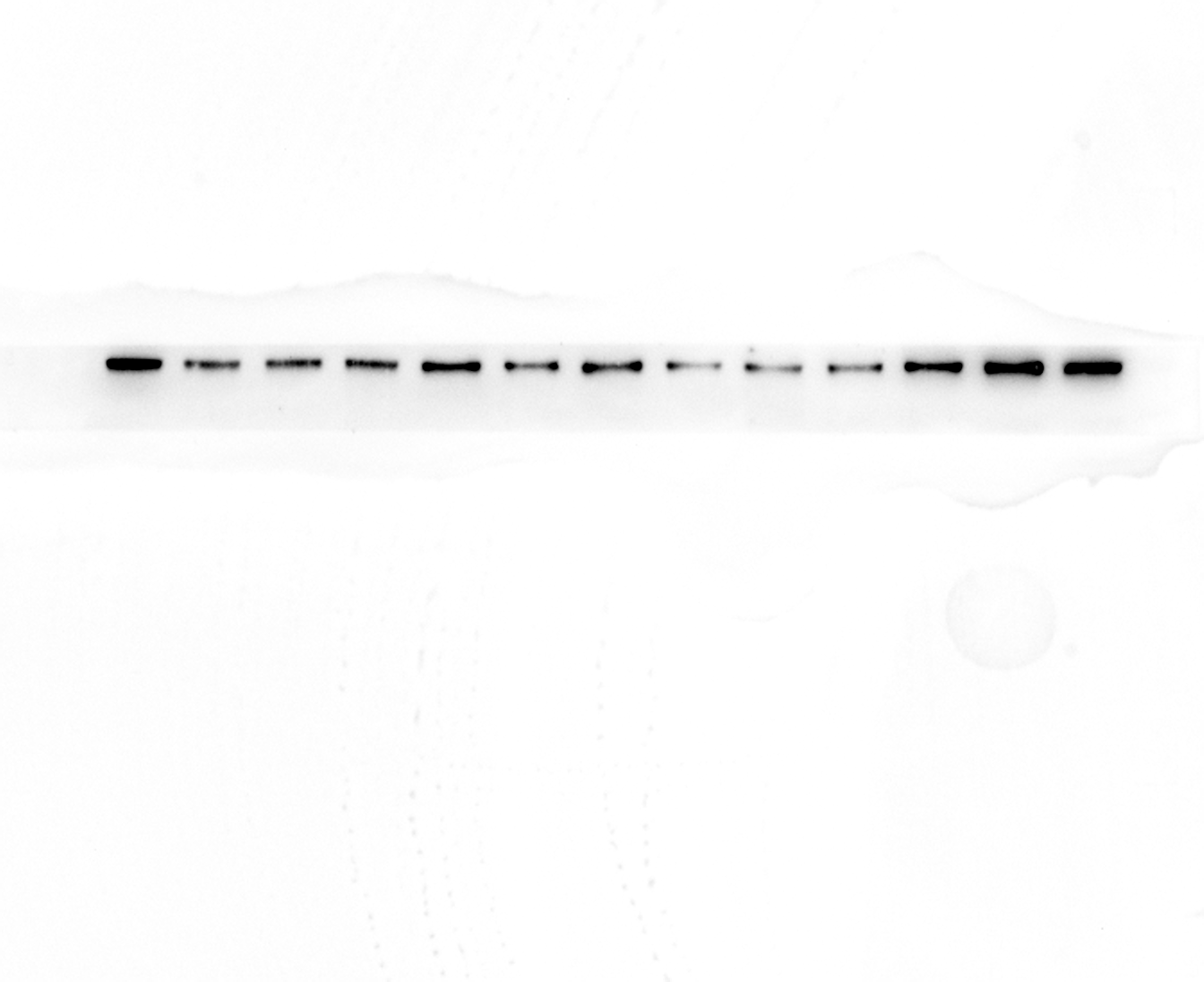

Supplement: Figure 6—source data 1. [file elife-69310-fig6-data1.zip › Figure 6-source data 1/nonaneurysmal patient #3(figure 6d)/MFN-1-003-1.Tif]

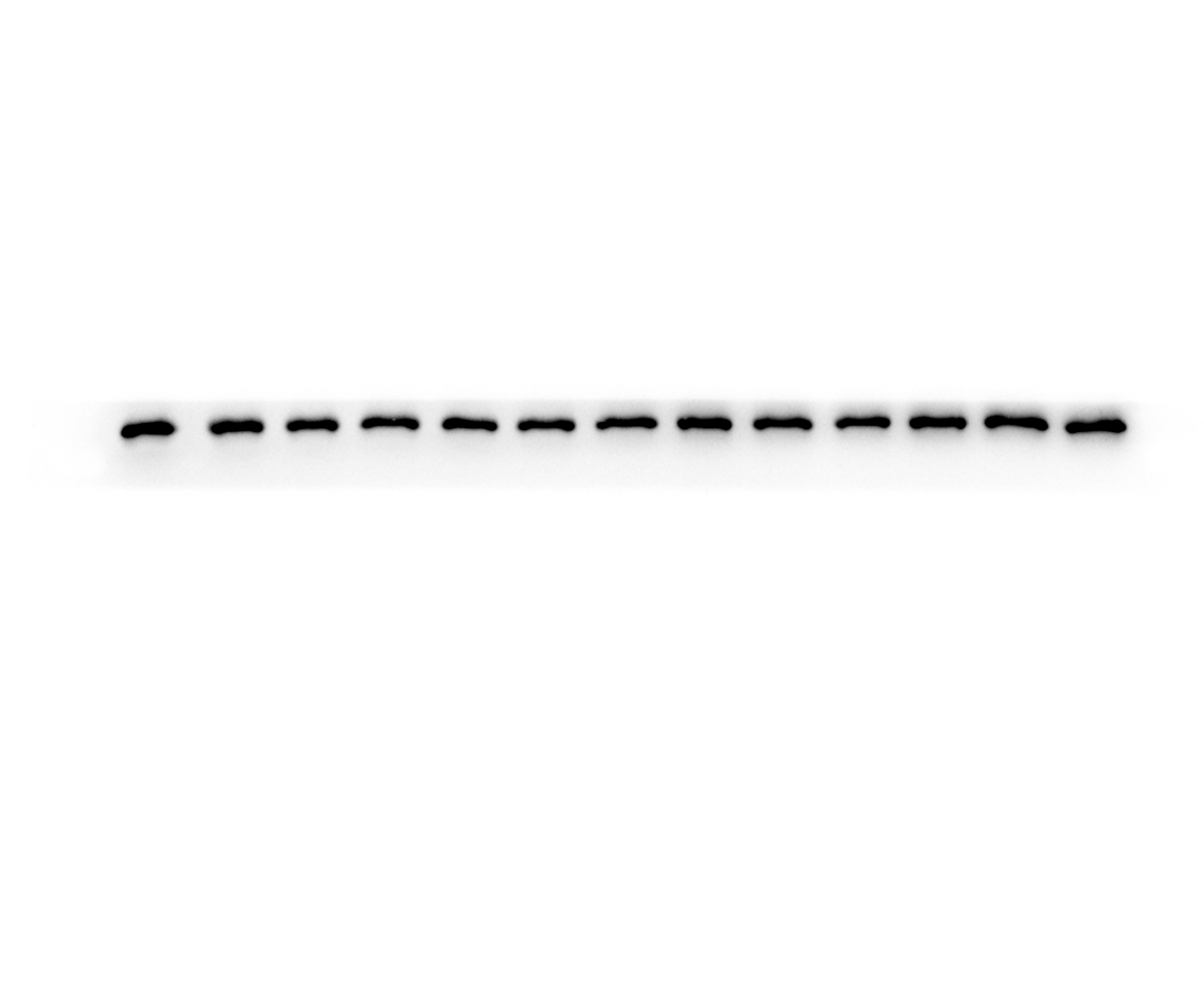

Supplement: Figure 6—source data 1. [file elife-69310-fig6-data1.zip › Figure 6-source data 1/nonaneurysmal patient #3(figure 6d)/MFN-2-001-1.Tif]

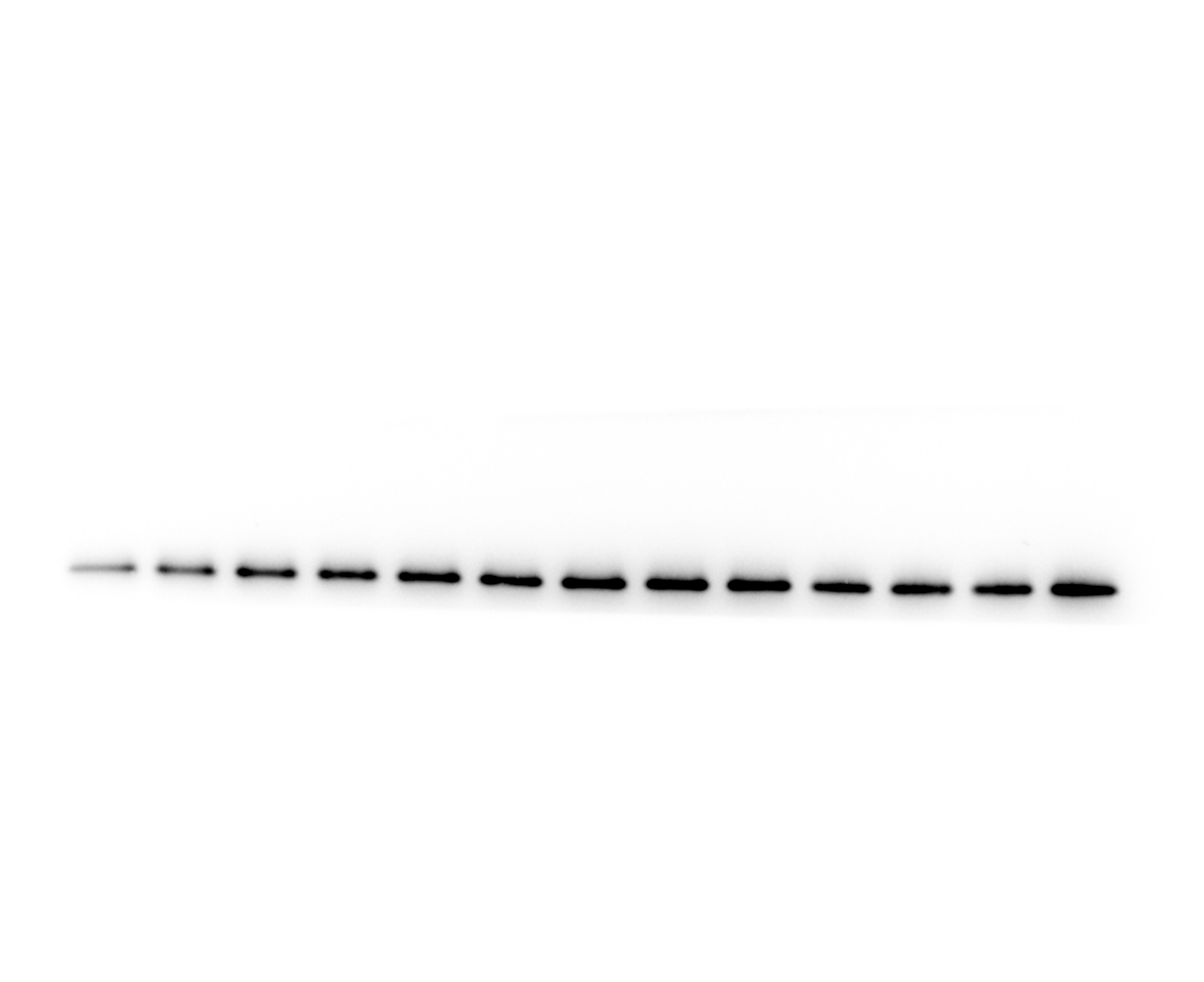

Supplement: Figure 6—source data 1. [file elife-69310-fig6-data1.zip › Figure 6-source data 1/nonaneurysmal patient #3(figure 6d)/SM22-003-3.Tif]

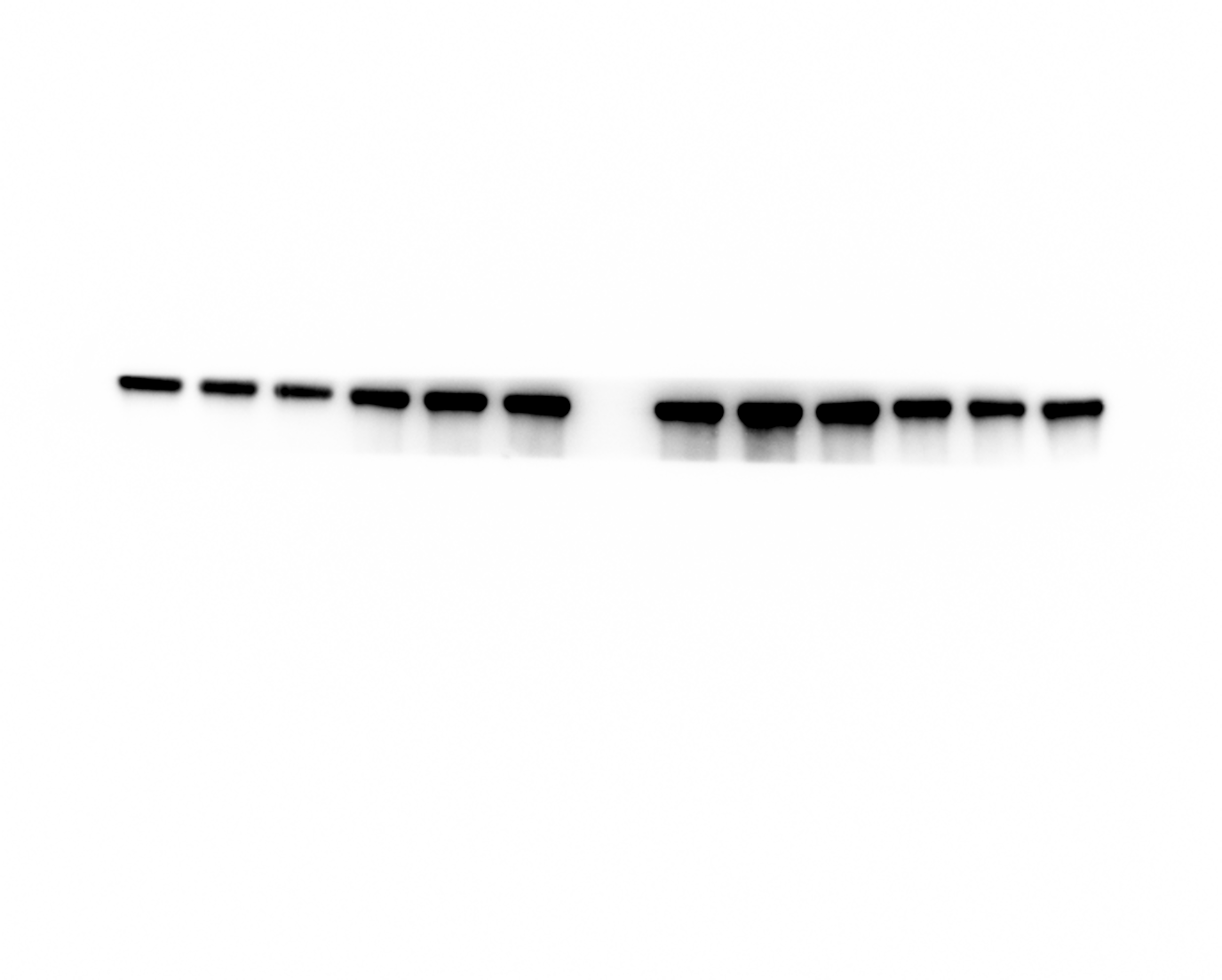

Supplement: Figure 6—figure supplement 1—source data 1. [file elife-69310-fig6-figsupp1-data1.zip › Figure 6-figure supplement 1-source data 1/BAV-TAA patient #1/3-4-B-ACTIN-001-1.Tif]

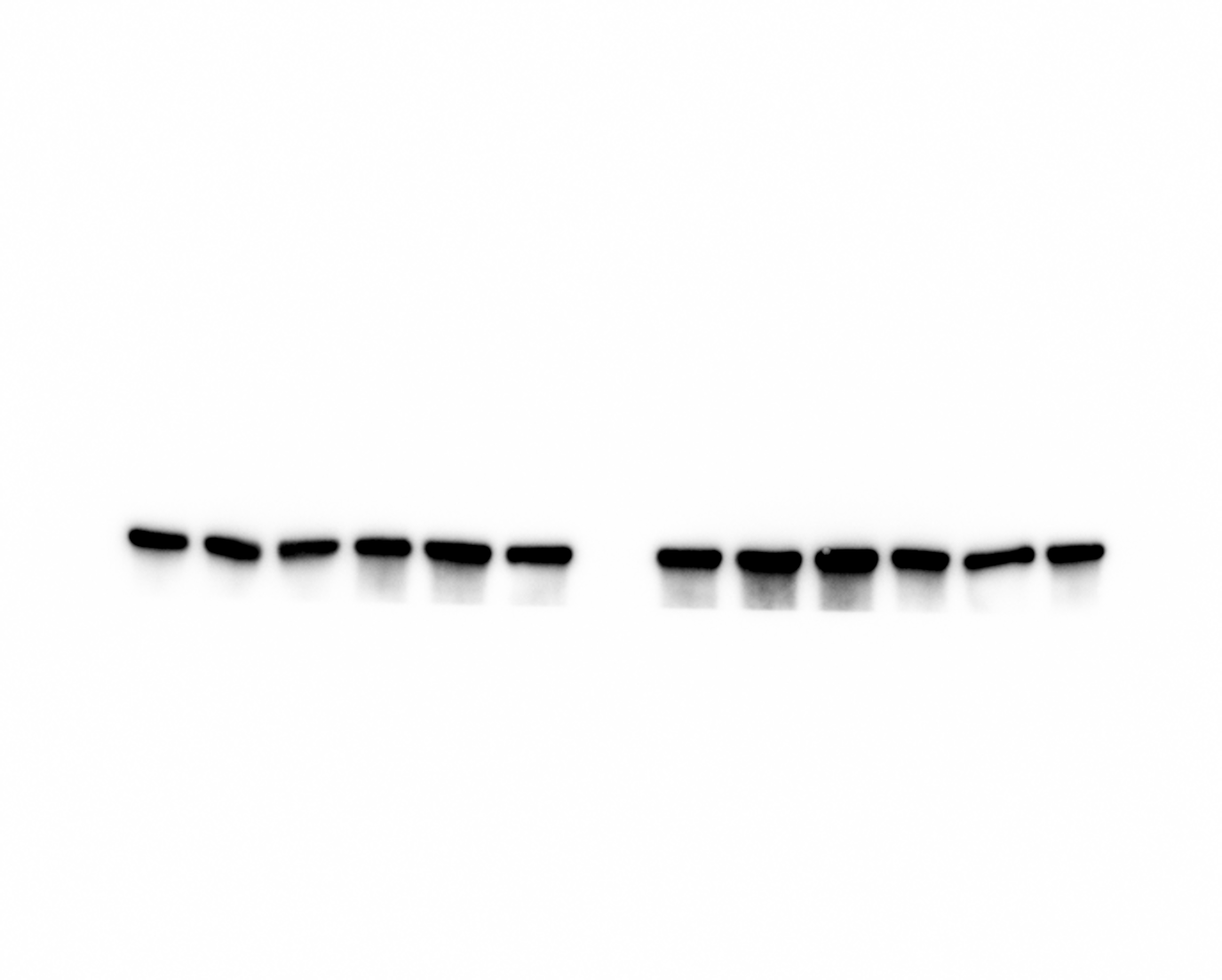

Supplement: Figure 6—figure supplement 1—source data 1. [file elife-69310-fig6-figsupp1-data1.zip › Figure 6-figure supplement 1-source data 1/BAV-TAA patient #1/3-4-B-ACTIN-002-3.Tif]

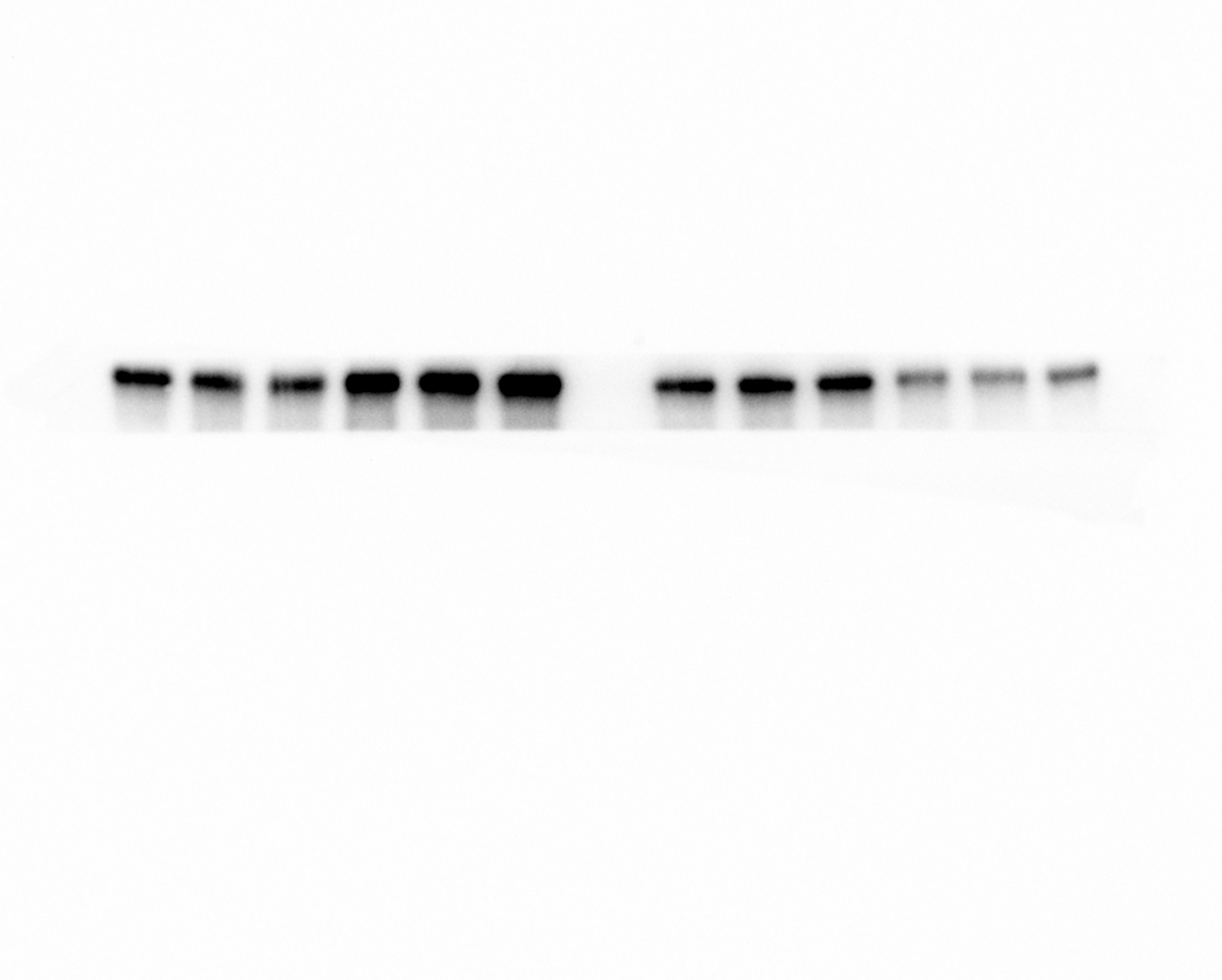

Supplement: Figure 6—figure supplement 1—source data 1. [file elife-69310-fig6-figsupp1-data1.zip › Figure 6-figure supplement 1-source data 1/BAV-TAA patient #1/3-4-CNN1-001-1.Tif]

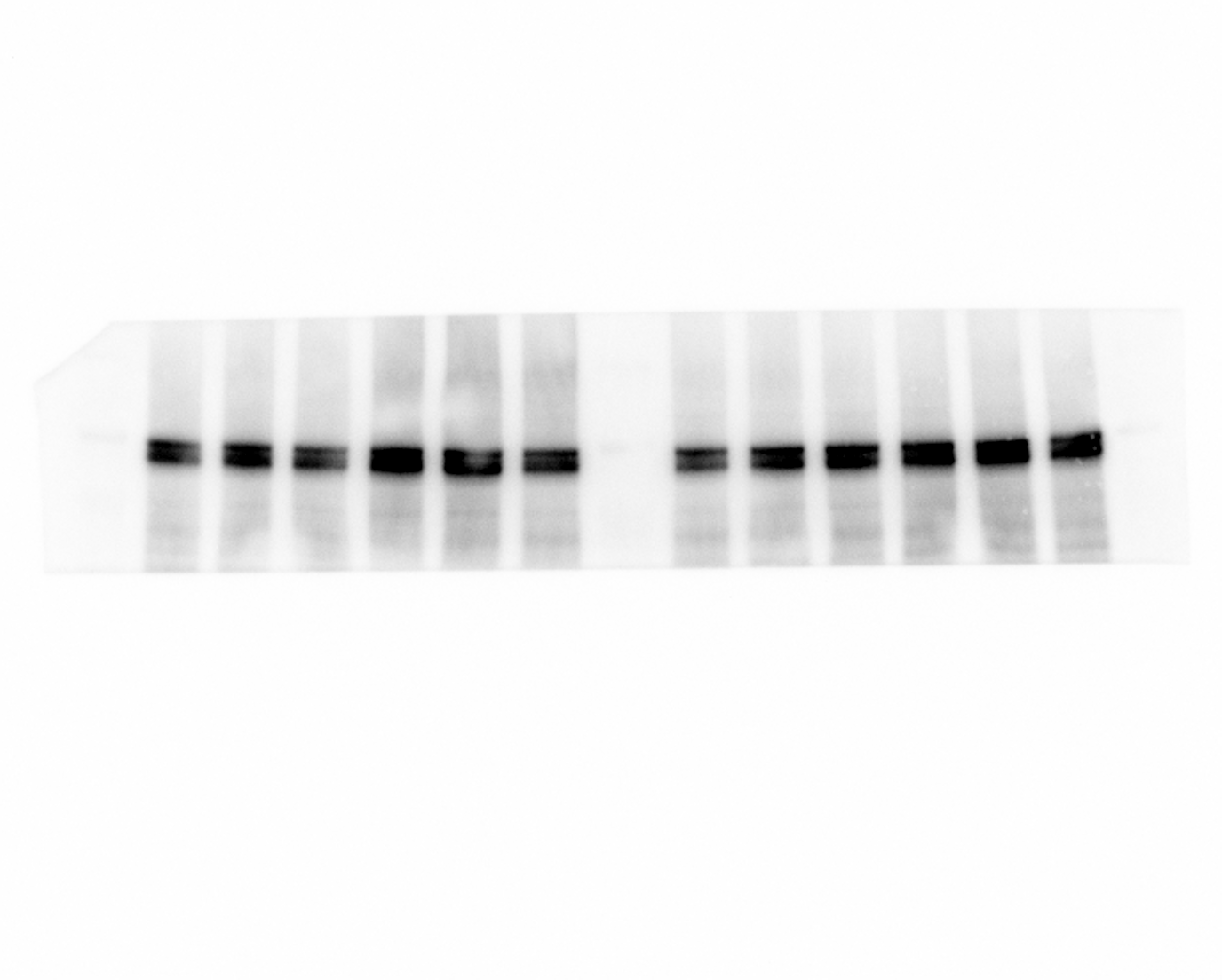

Supplement: Figure 6—figure supplement 1—source data 1. [file elife-69310-fig6-figsupp1-data1.zip › Figure 6-figure supplement 1-source data 1/BAV-TAA patient #1/3-4-DRP1-002-3.Tif]

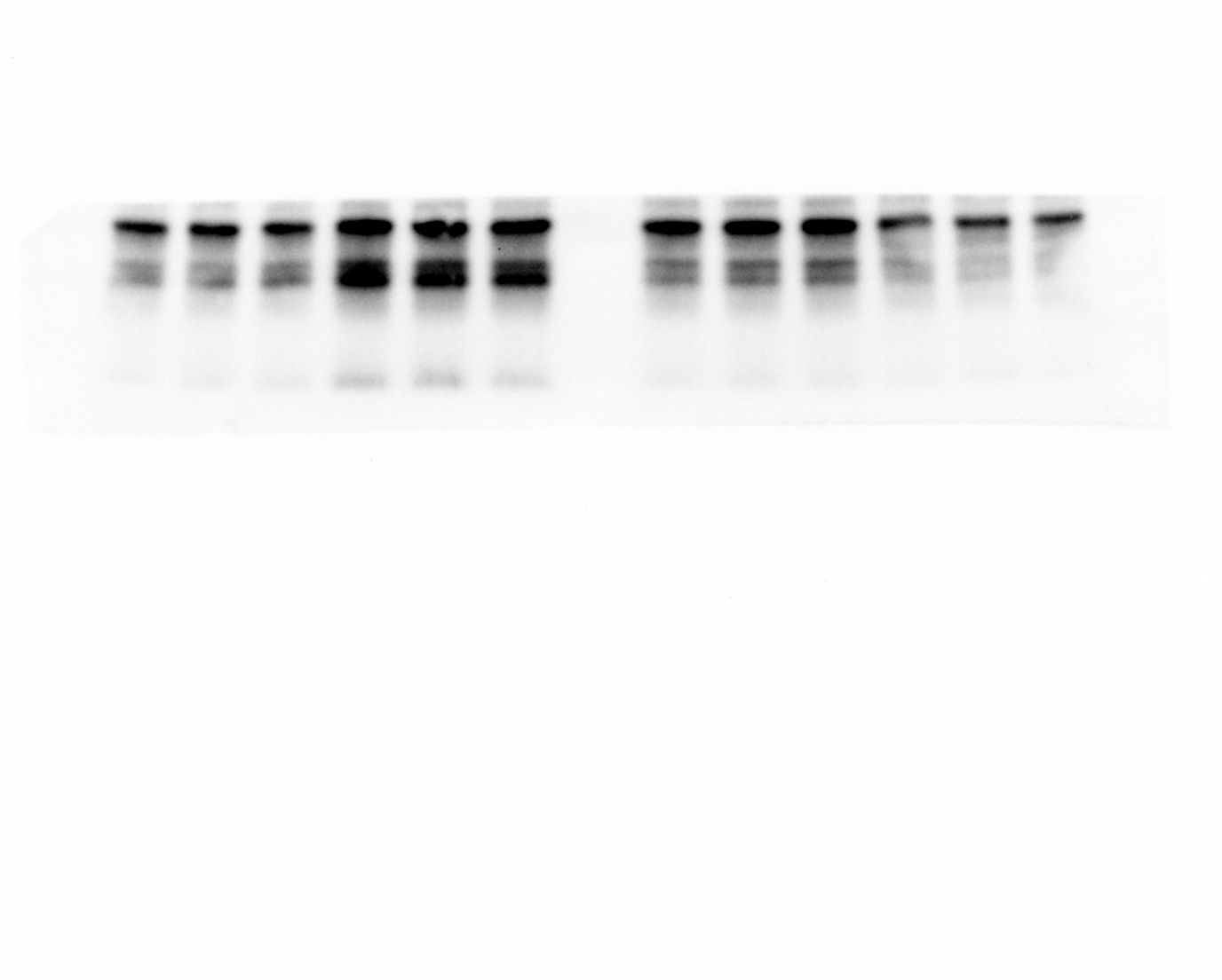

Supplement: Figure 6—figure supplement 1—source data 1. [file elife-69310-fig6-figsupp1-data1.zip › Figure 6-figure supplement 1-source data 1/BAV-TAA patient #1/3-4-MFF-002-3.Tif]

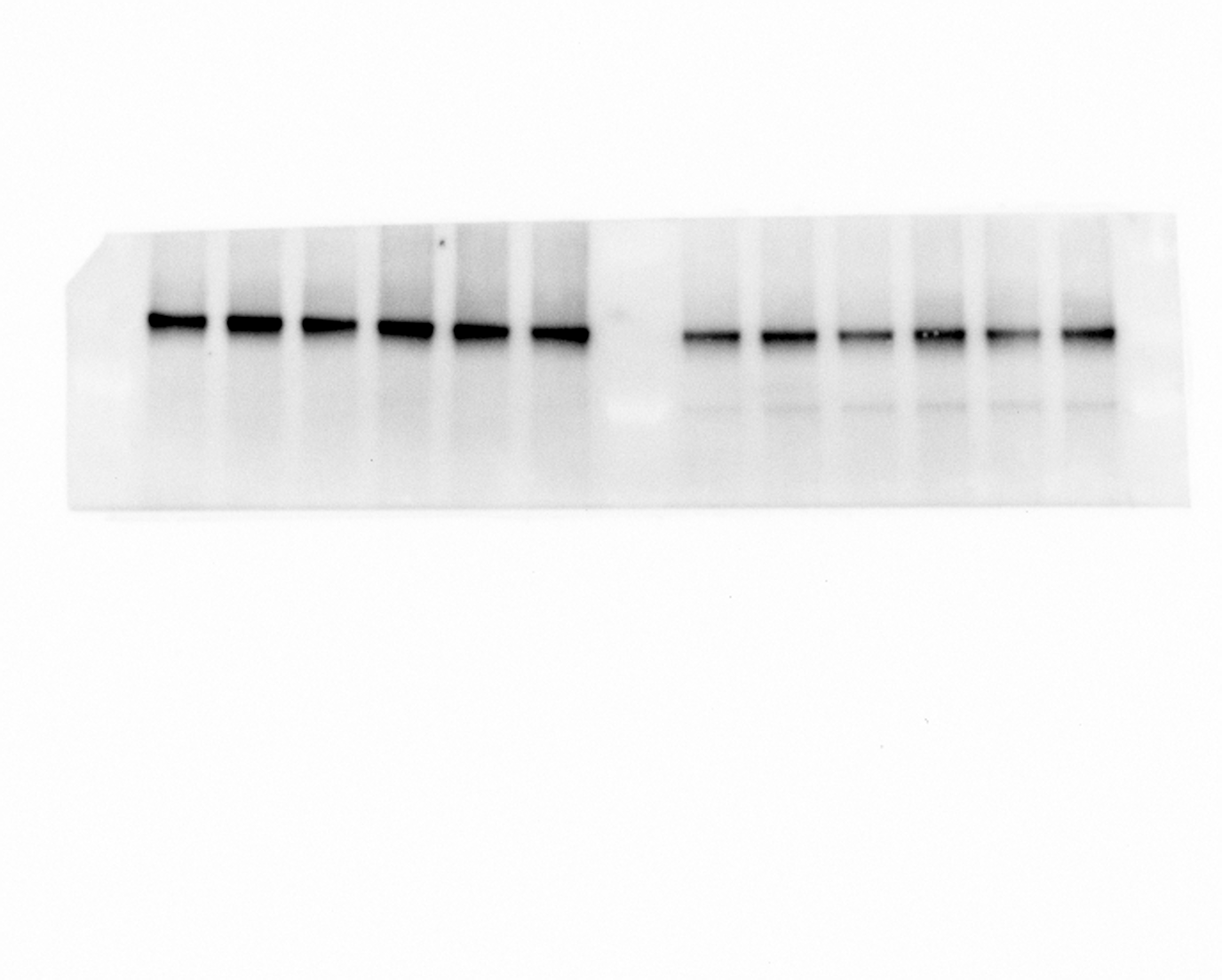

Supplement: Figure 6—figure supplement 1—source data 1. [file elife-69310-fig6-figsupp1-data1.zip › Figure 6-figure supplement 1-source data 1/BAV-TAA patient #1/3-4-MFN1-001-3.Tif]
