# Supplementary material for: Aorta smooth muscle-on-a-chip reveals impaired mitochondrial dynamics as a therapeutic target for aortic aneurysm in bicuspid aortic valve disease
Source: eLife. 2021 Sep 6;10:e69310. doi: 10.7554/eLife.69310 (PMC8451027; doi:10.7554/eLife.69310)

non-diseased patient #1

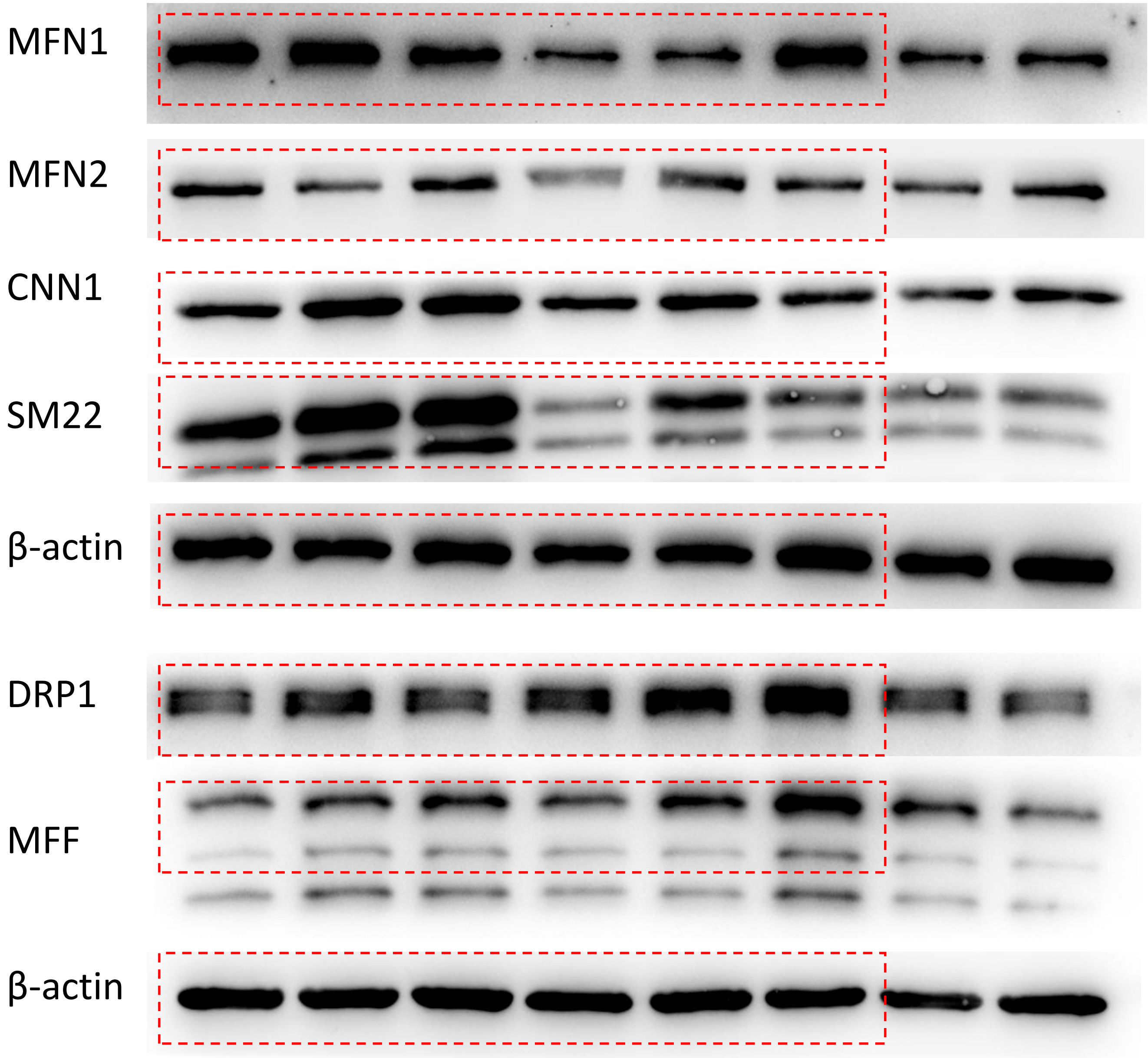

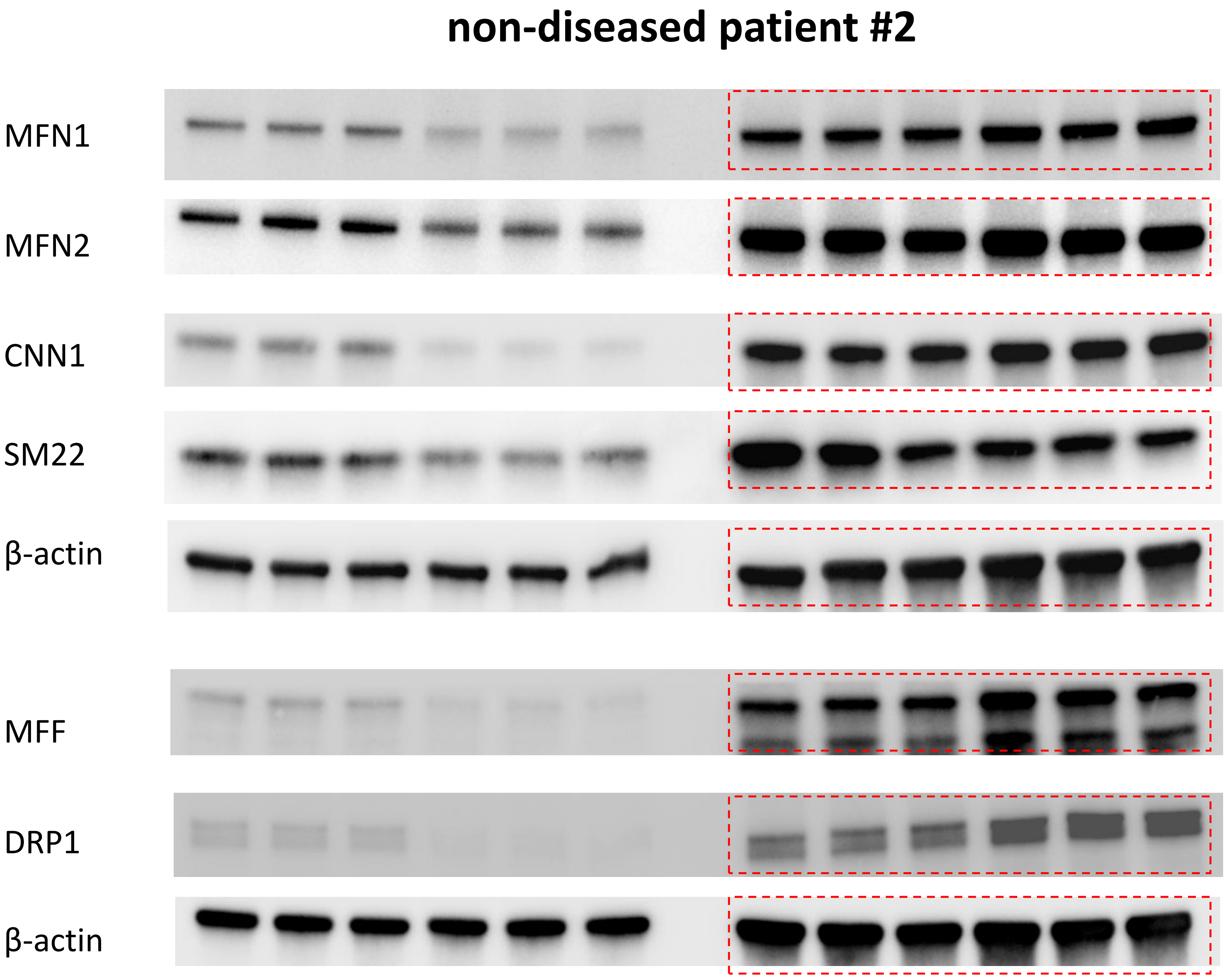
non-diseased patient #2
MFN1
MFN2
CNN1
SM22
β-actin
MFF
DRP1
β-actin

non-diseased patient #3

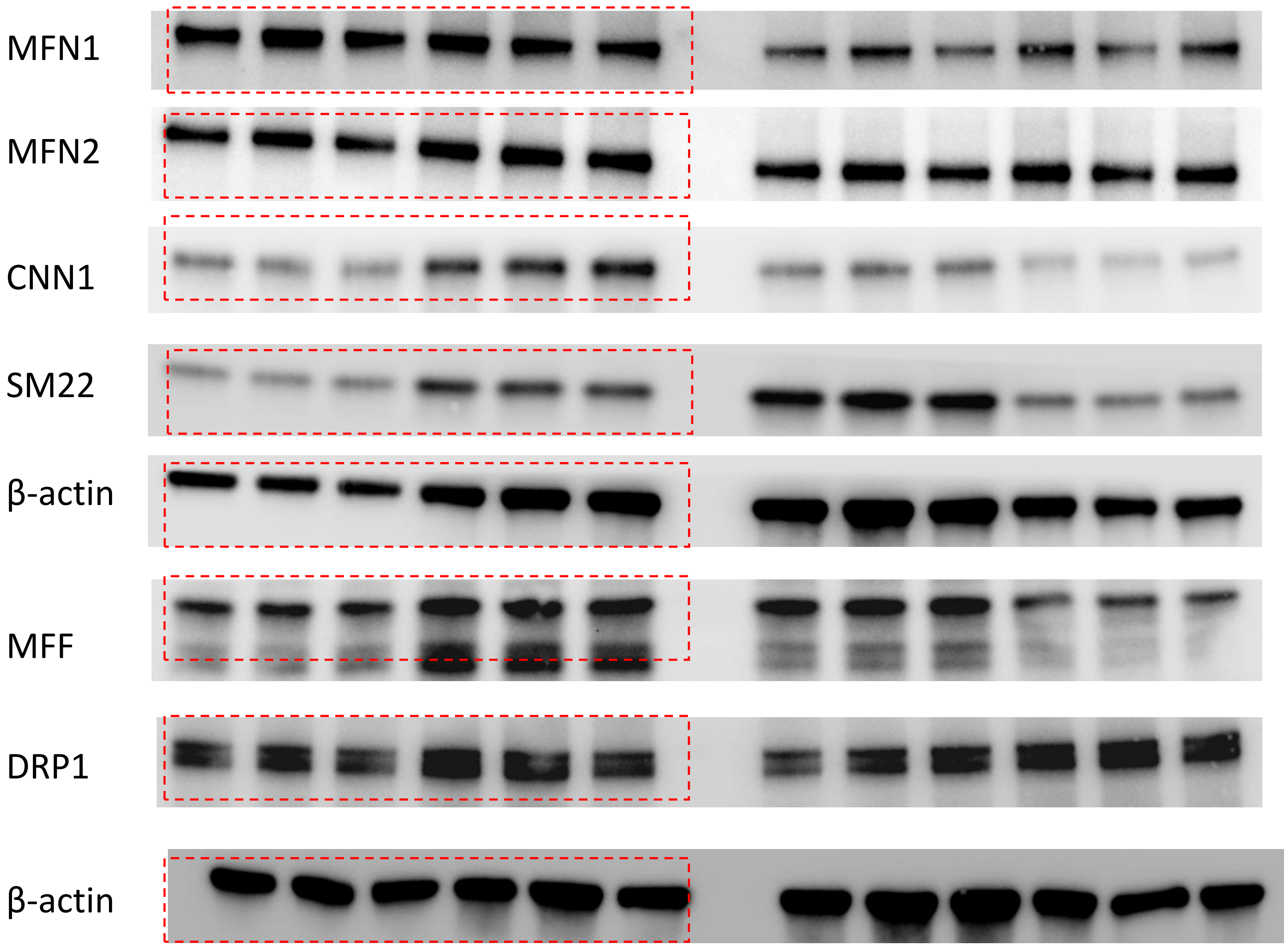

BAV-TAA patient #1

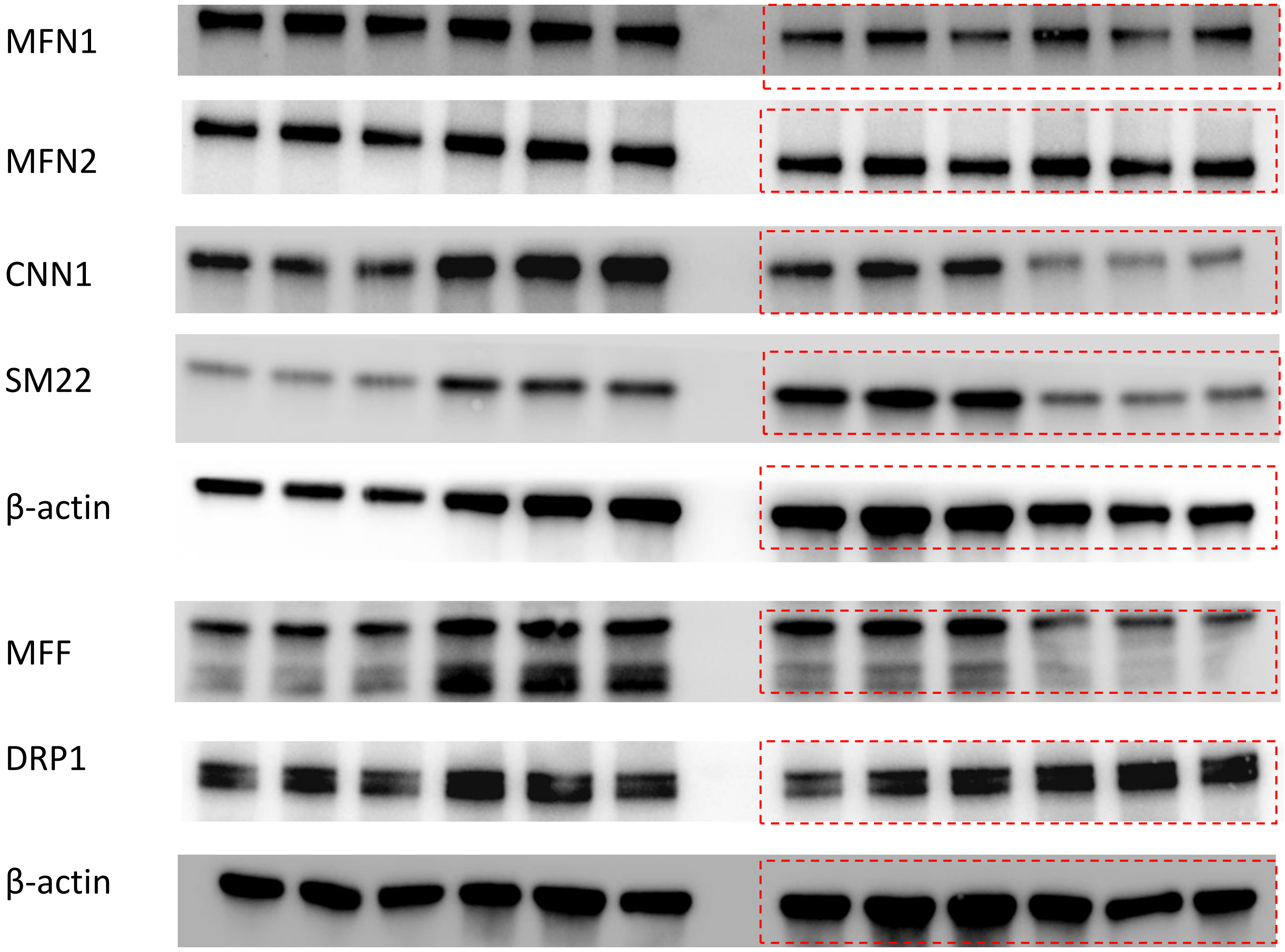

BAV-TAA patient # 2

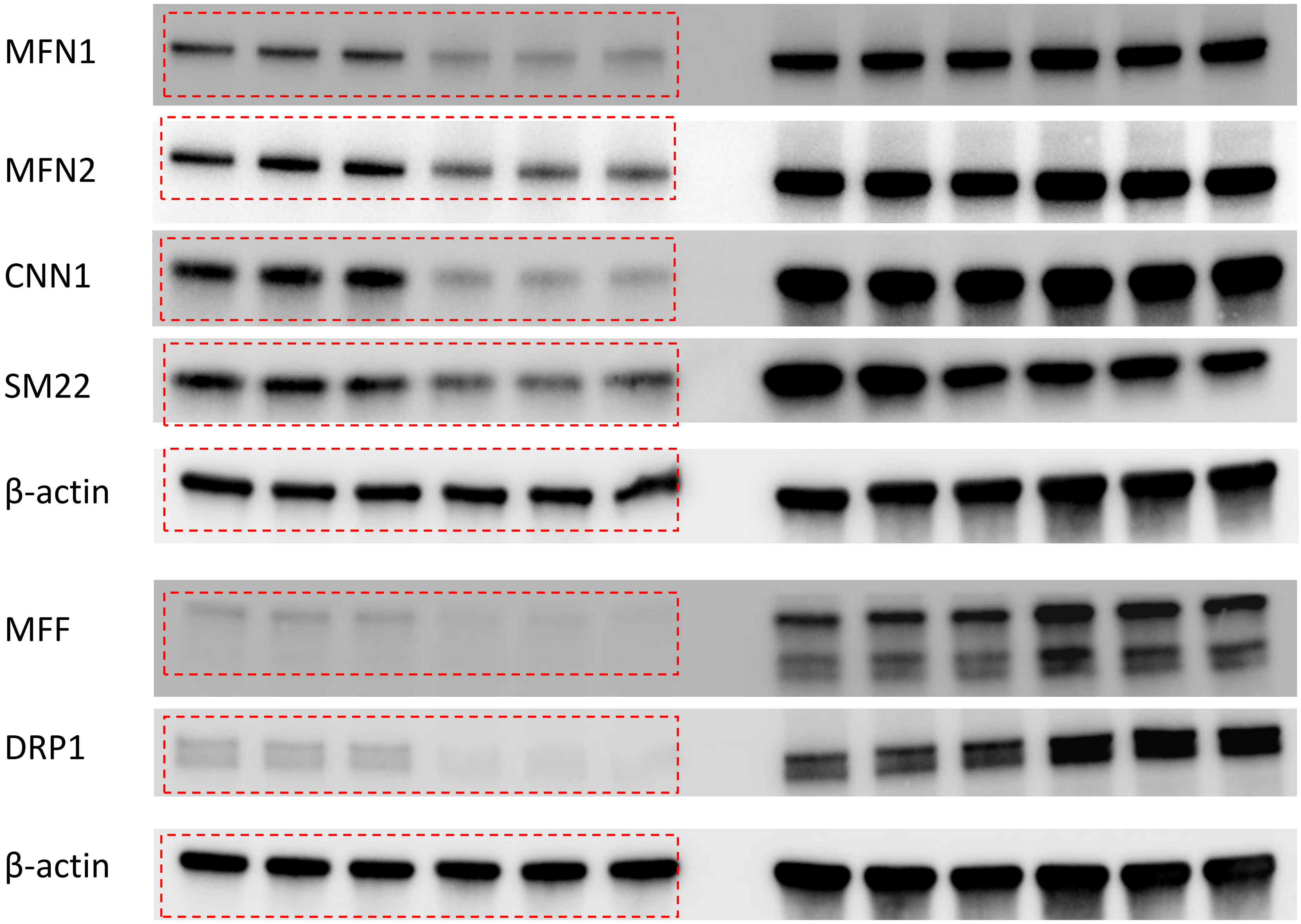

BAV-TAA patient # 3

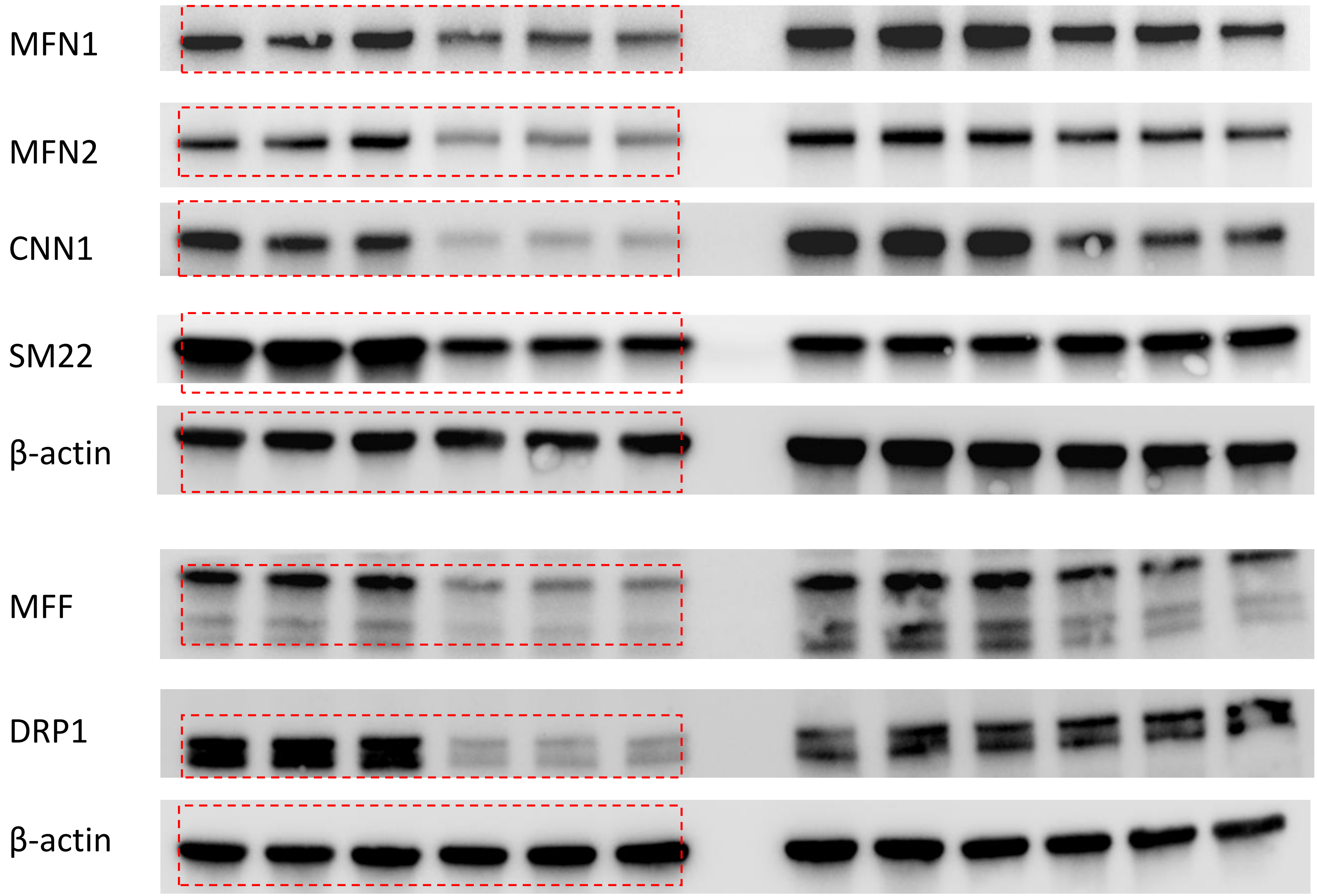

Supplement: Figure 6—figure supplement 1—source data 1. [file elife-69310-fig6-figsupp1-data1.zip › Figure 6-figure supplement 1-source data 1/labeled uncropped WB .pdf]
