# Supplementary figures and images for: Aorta smooth muscle-on-a-chip reveals impaired mitochondrial dynamics as a therapeutic target for aortic aneurysm in bicuspid aortic valve disease (part 2 of 2)
Source: eLife. 2021 Sep 6;10:e69310. doi: 10.7554/eLife.69310 (PMC8451027; doi:10.7554/eLife.69310)

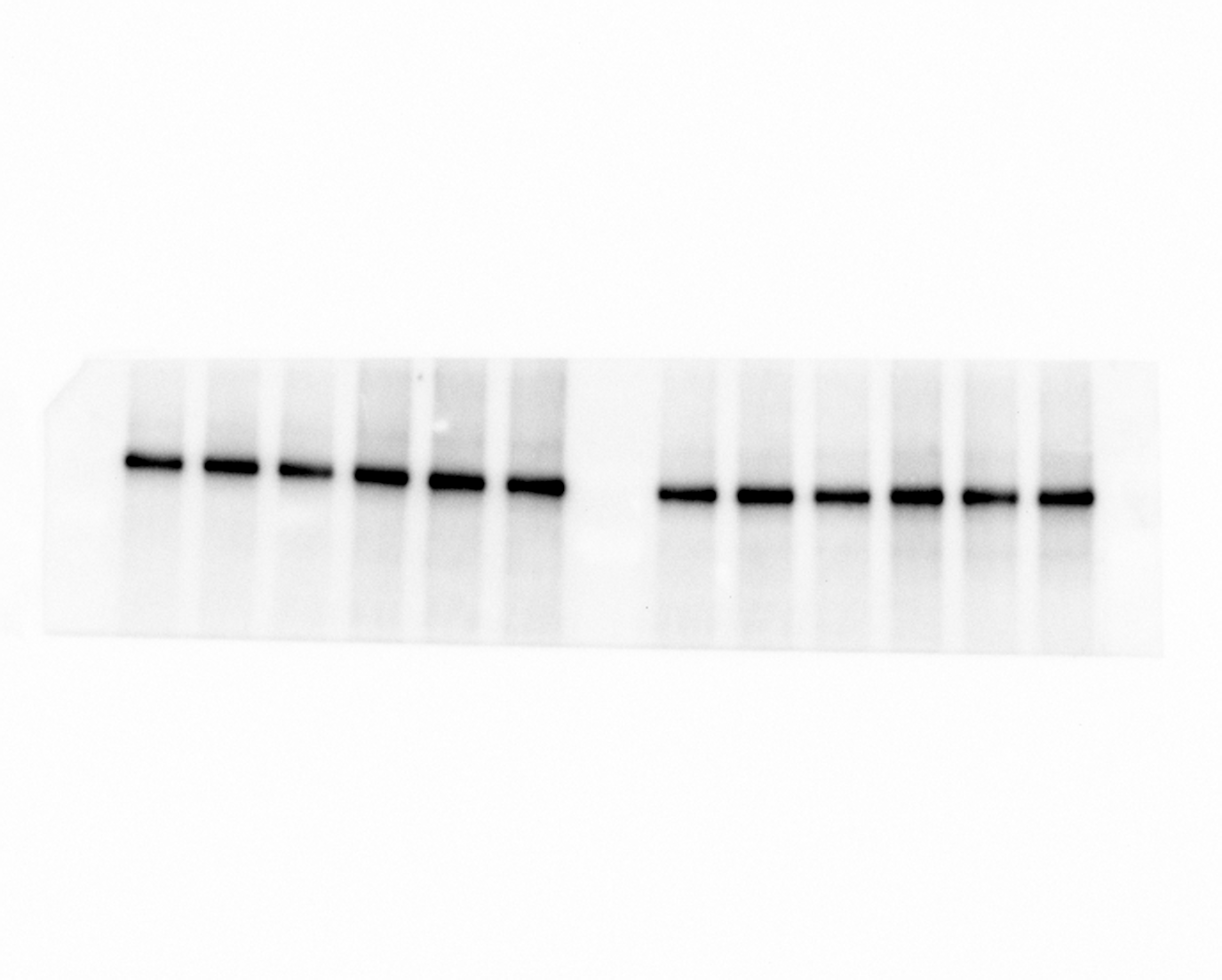

Supplement: Figure 6—figure supplement 1—source data 1. [file elife-69310-fig6-figsupp1-data1.zip › Figure 6-figure supplement 1-source data 1/BAV-TAA patient #1/3-4-MFN2-003.Tif]

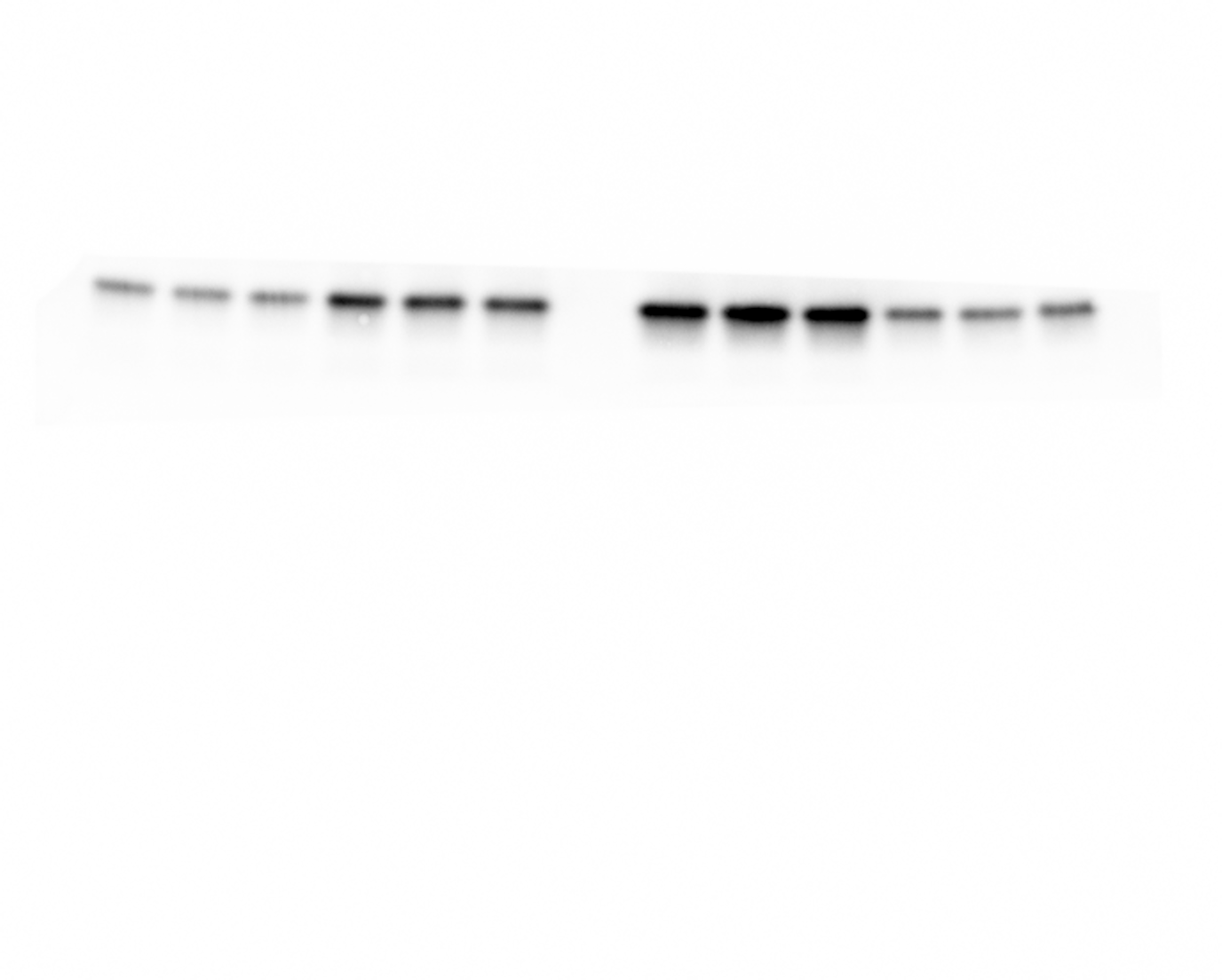

Supplement: Figure 6—figure supplement 1—source data 1. [file elife-69310-fig6-figsupp1-data1.zip › Figure 6-figure supplement 1-source data 1/BAV-TAA patient #1/3-4-SM22-003-1.Tif]

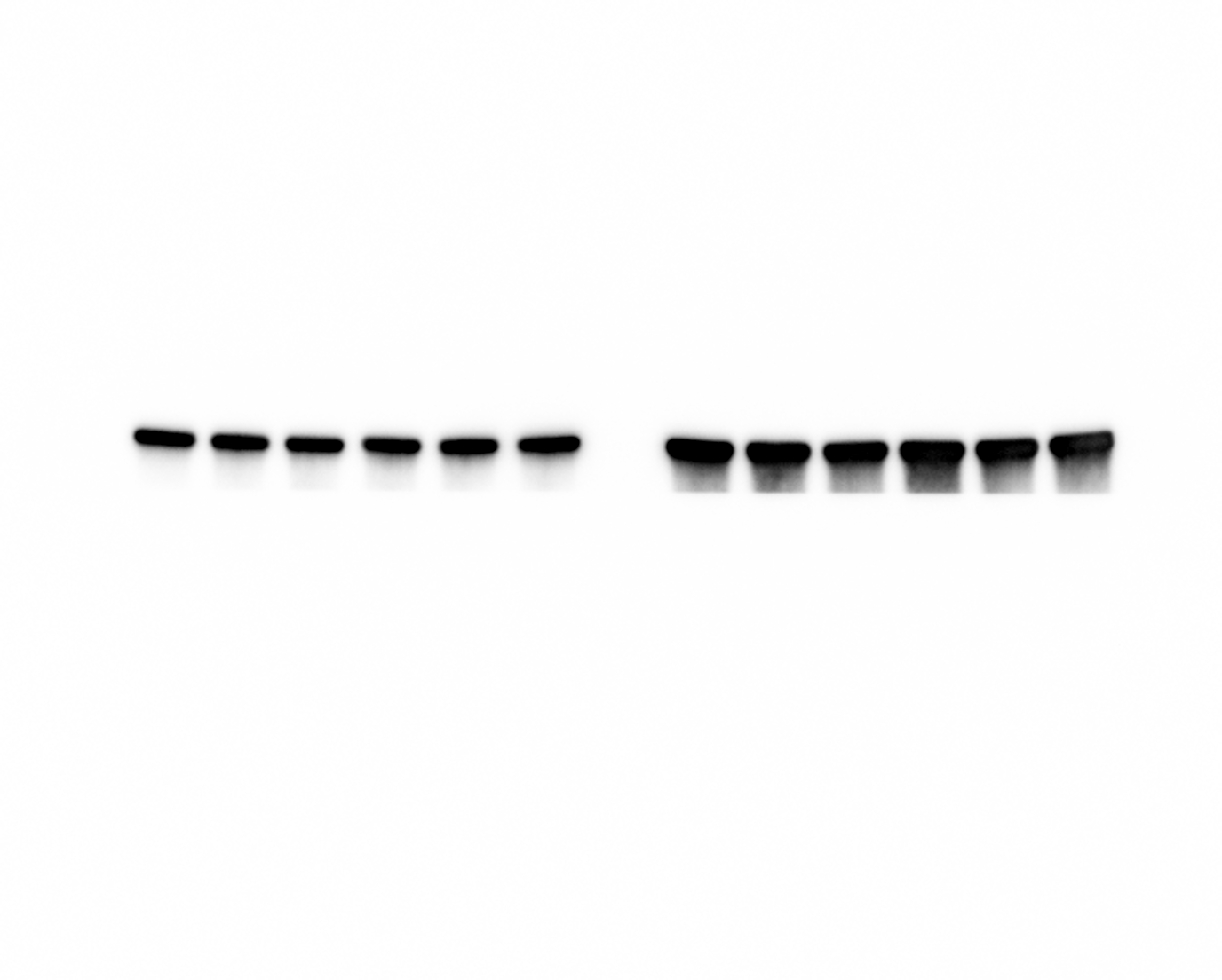

Supplement: Figure 6—figure supplement 1—source data 1. [file elife-69310-fig6-figsupp1-data1.zip › Figure 6-figure supplement 1-source data 1/BAV-TAA patient #2/1-2-B-ACTIN-001-3.Tif]

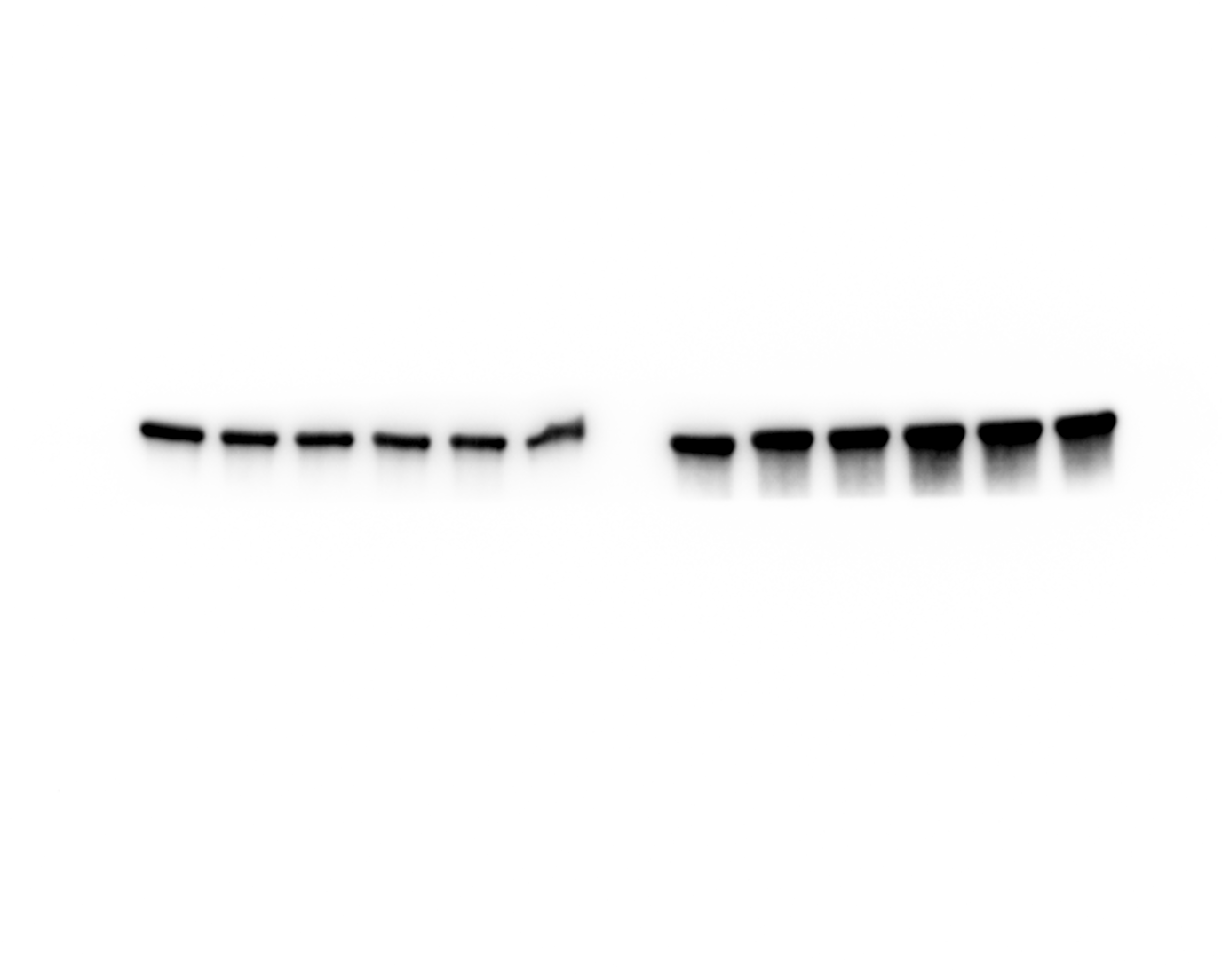

Supplement: Figure 6—figure supplement 1—source data 1. [file elife-69310-fig6-figsupp1-data1.zip › Figure 6-figure supplement 1-source data 1/BAV-TAA patient #2/1-2-B-ACTIN-002-1.Tif]

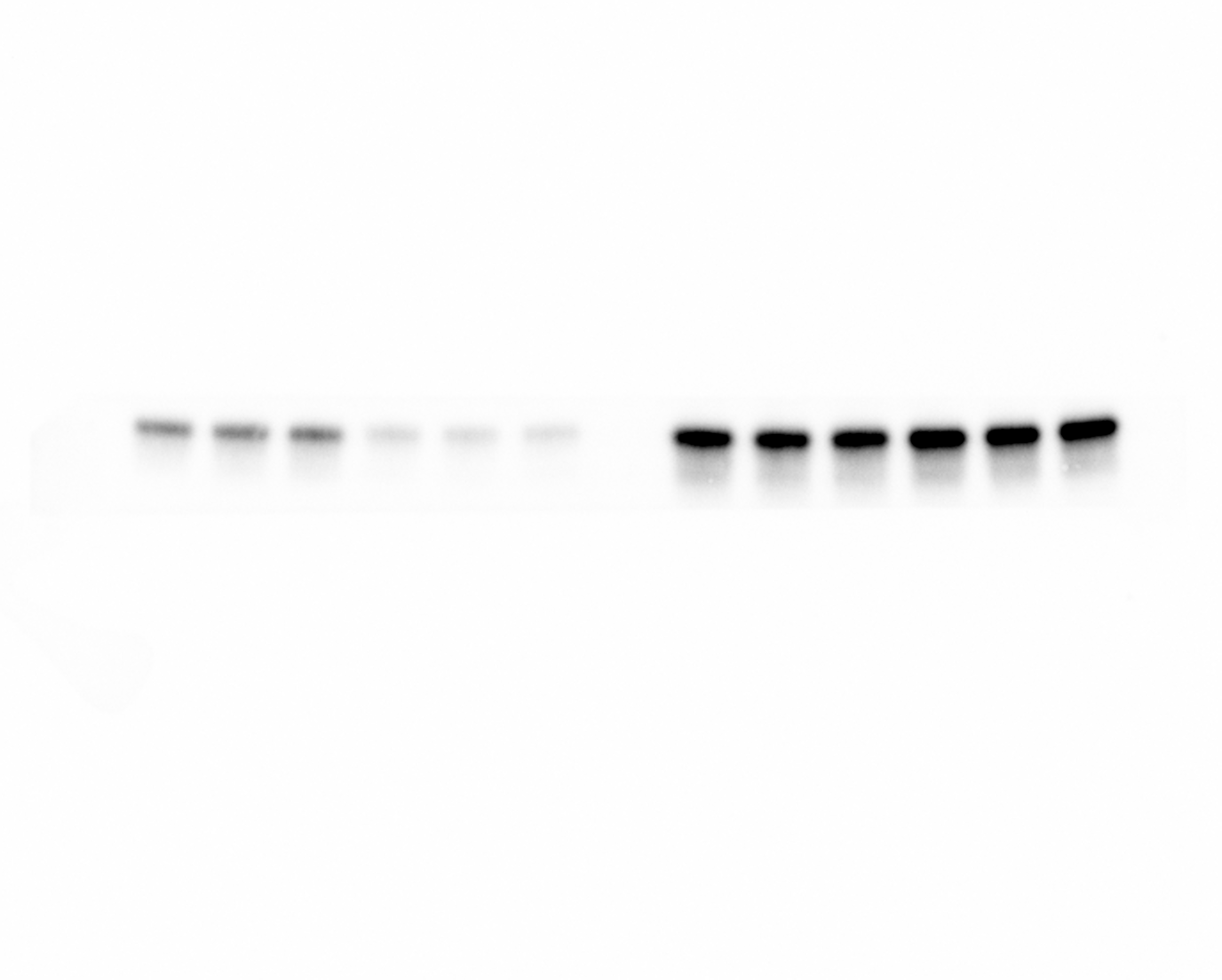

Supplement: Figure 6—figure supplement 1—source data 1. [file elife-69310-fig6-figsupp1-data1.zip › Figure 6-figure supplement 1-source data 1/BAV-TAA patient #2/1-2-CNN1-001-1.Tif]

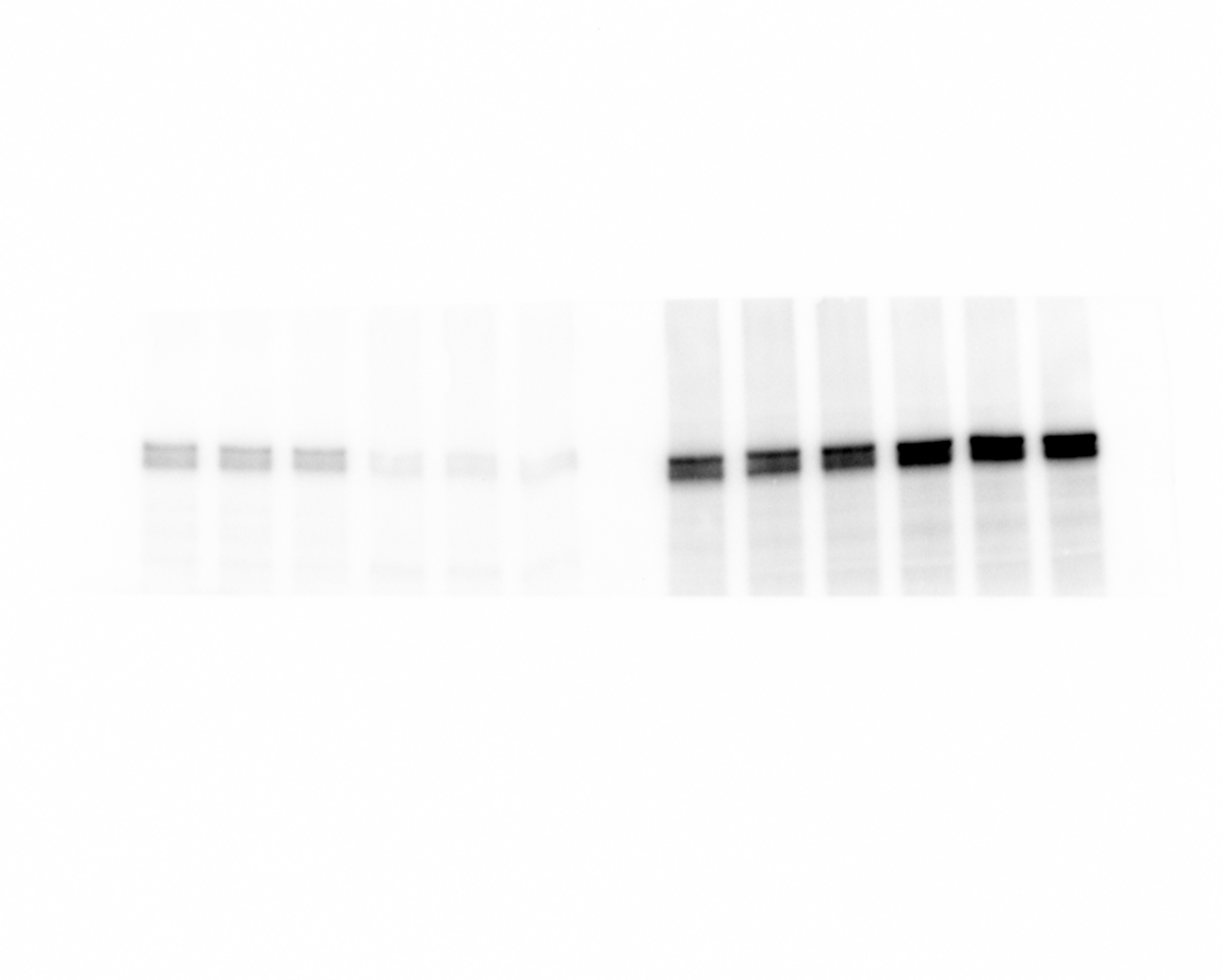

Supplement: Figure 6—figure supplement 1—source data 1. [file elife-69310-fig6-figsupp1-data1.zip › Figure 6-figure supplement 1-source data 1/BAV-TAA patient #2/1-2-DRP1-002-1.Tif]

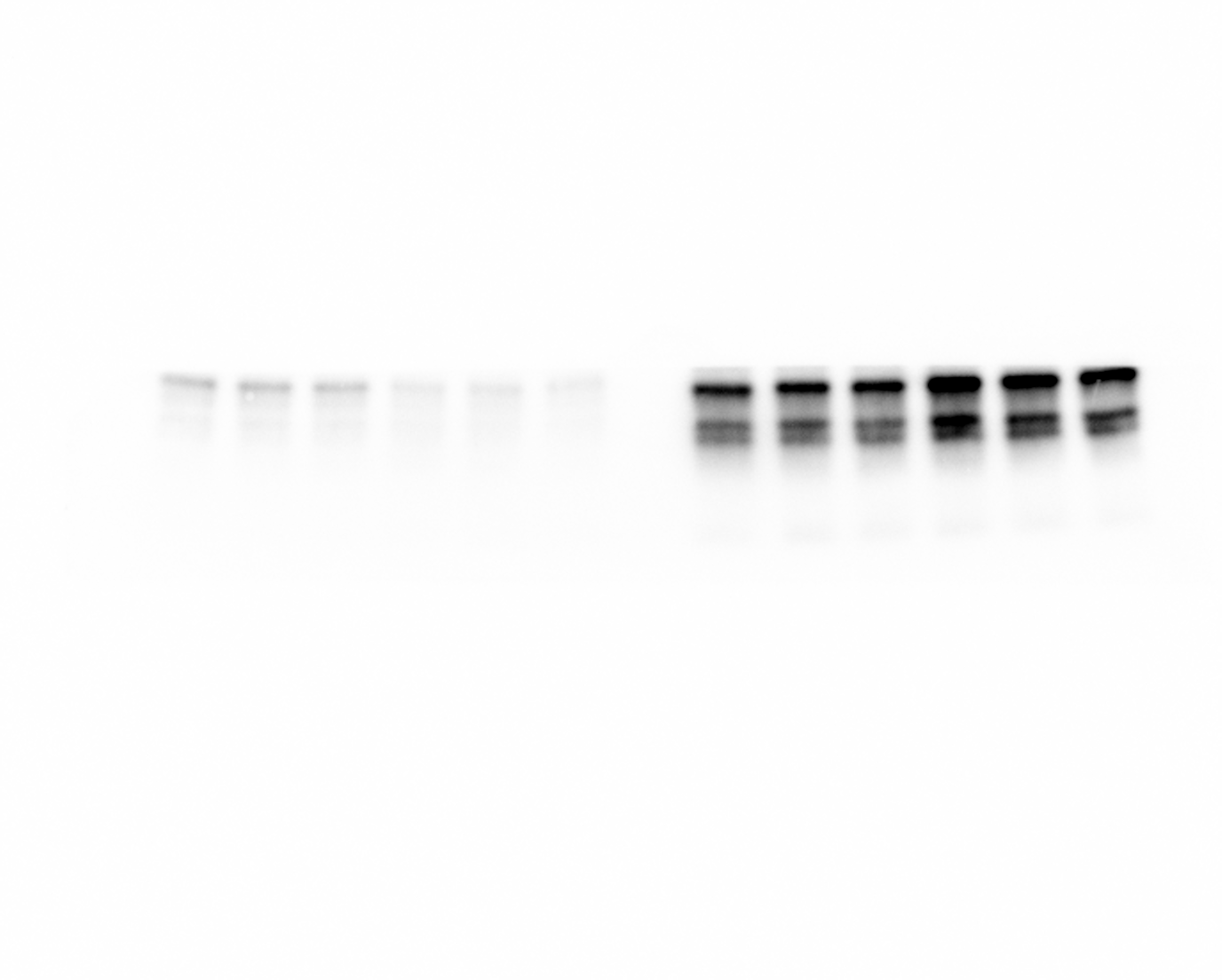

Supplement: Figure 6—figure supplement 1—source data 1. [file elife-69310-fig6-figsupp1-data1.zip › Figure 6-figure supplement 1-source data 1/BAV-TAA patient #2/1-2-MFF-002-1.Tif]

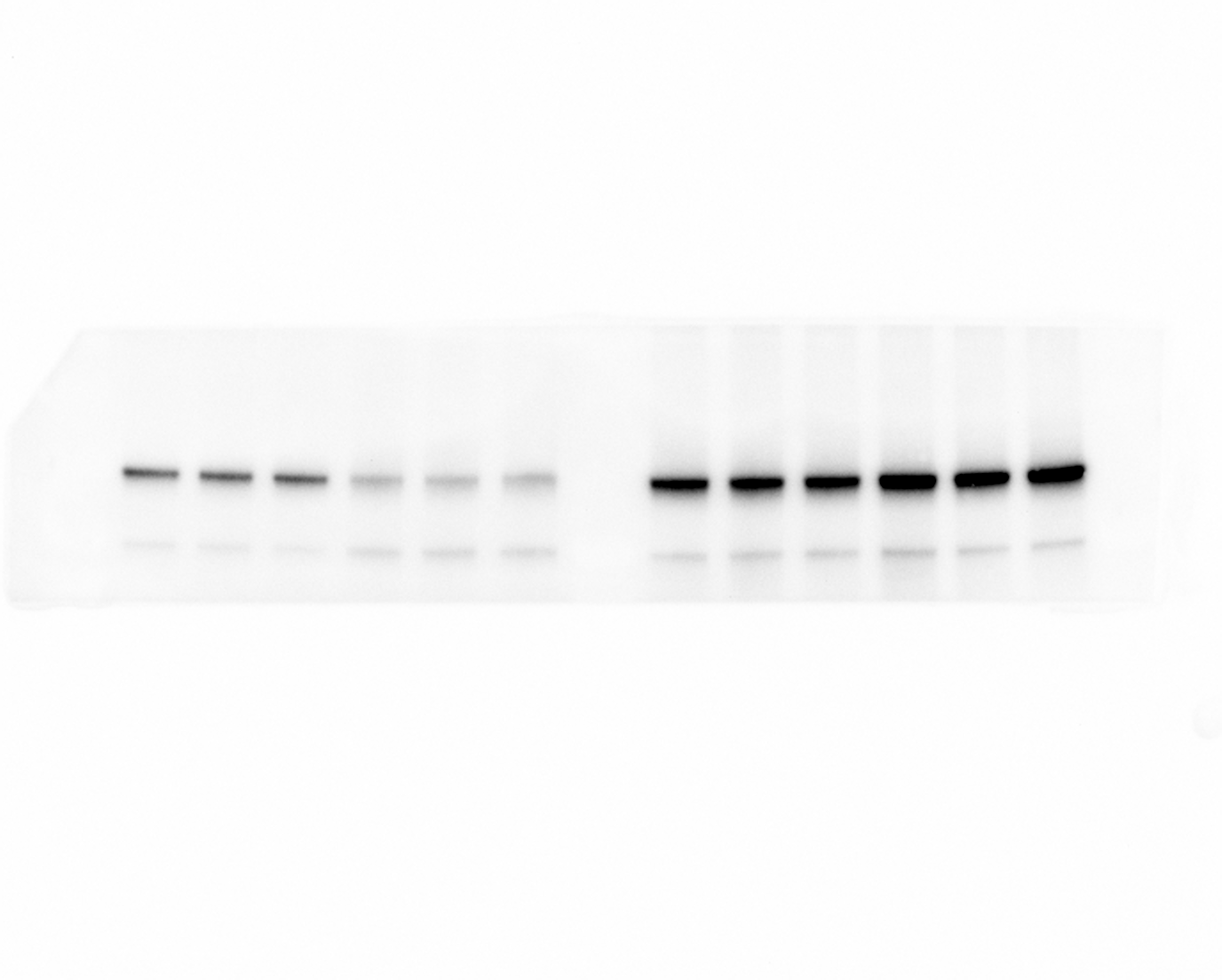

Supplement: Figure 6—figure supplement 1—source data 1. [file elife-69310-fig6-figsupp1-data1.zip › Figure 6-figure supplement 1-source data 1/BAV-TAA patient #2/1-2-MFN1-001-1.Tif]

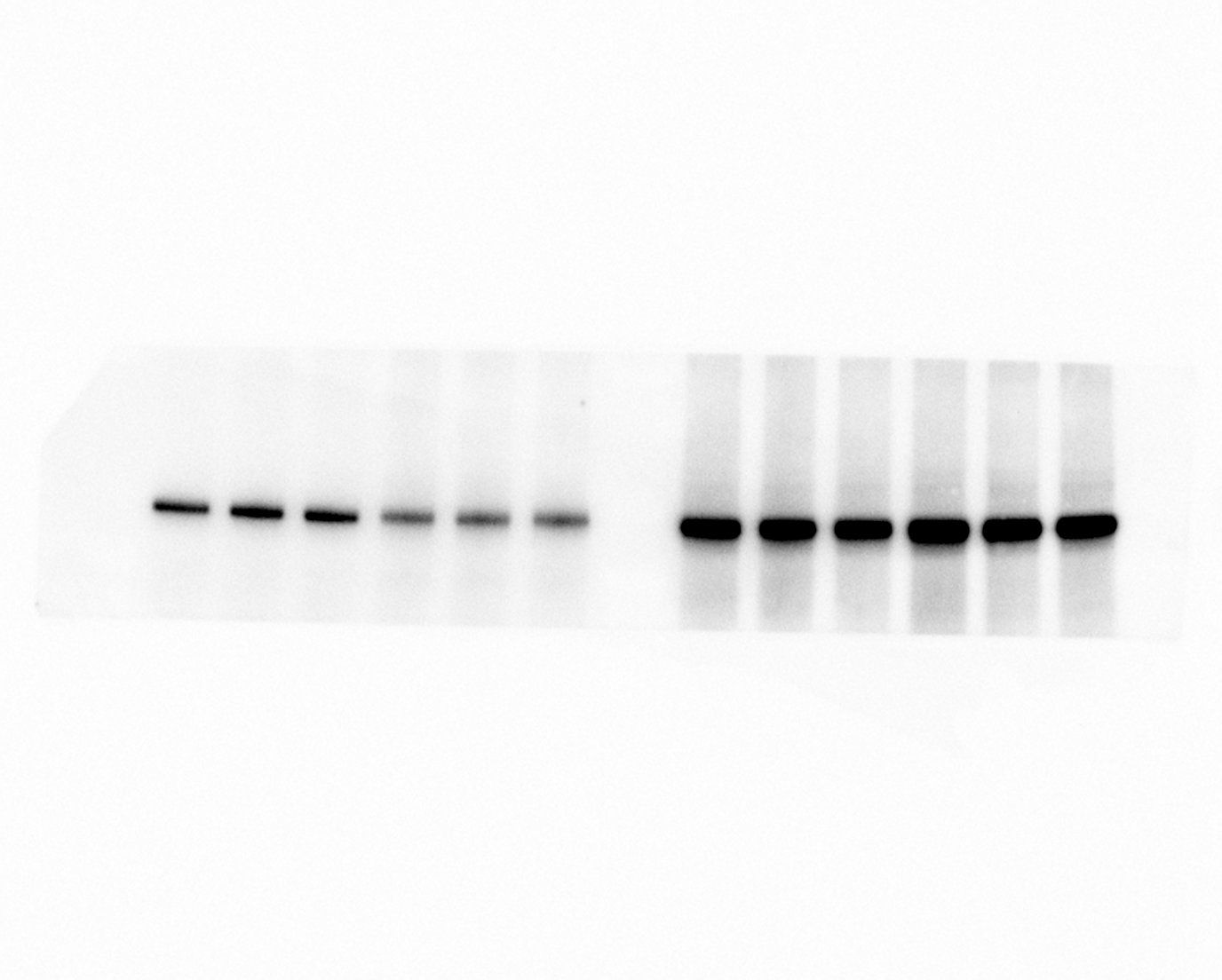

Supplement: Figure 6—figure supplement 1—source data 1. [file elife-69310-fig6-figsupp1-data1.zip › Figure 6-figure supplement 1-source data 1/BAV-TAA patient #2/1-2-MFN2-001.Tif]

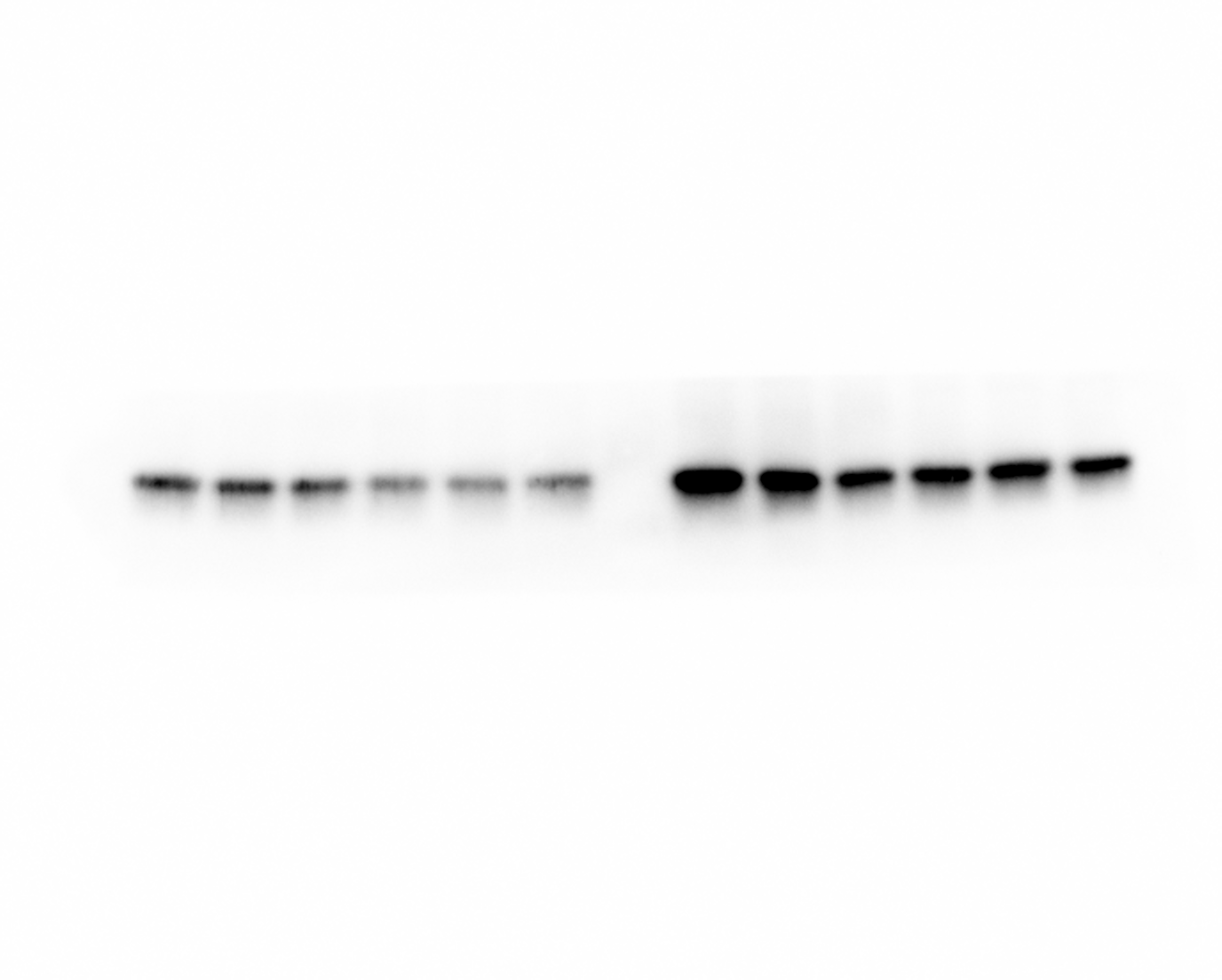

Supplement: Figure 6—figure supplement 1—source data 1. [file elife-69310-fig6-figsupp1-data1.zip › Figure 6-figure supplement 1-source data 1/BAV-TAA patient #2/1-2-SM22-3.Tif]

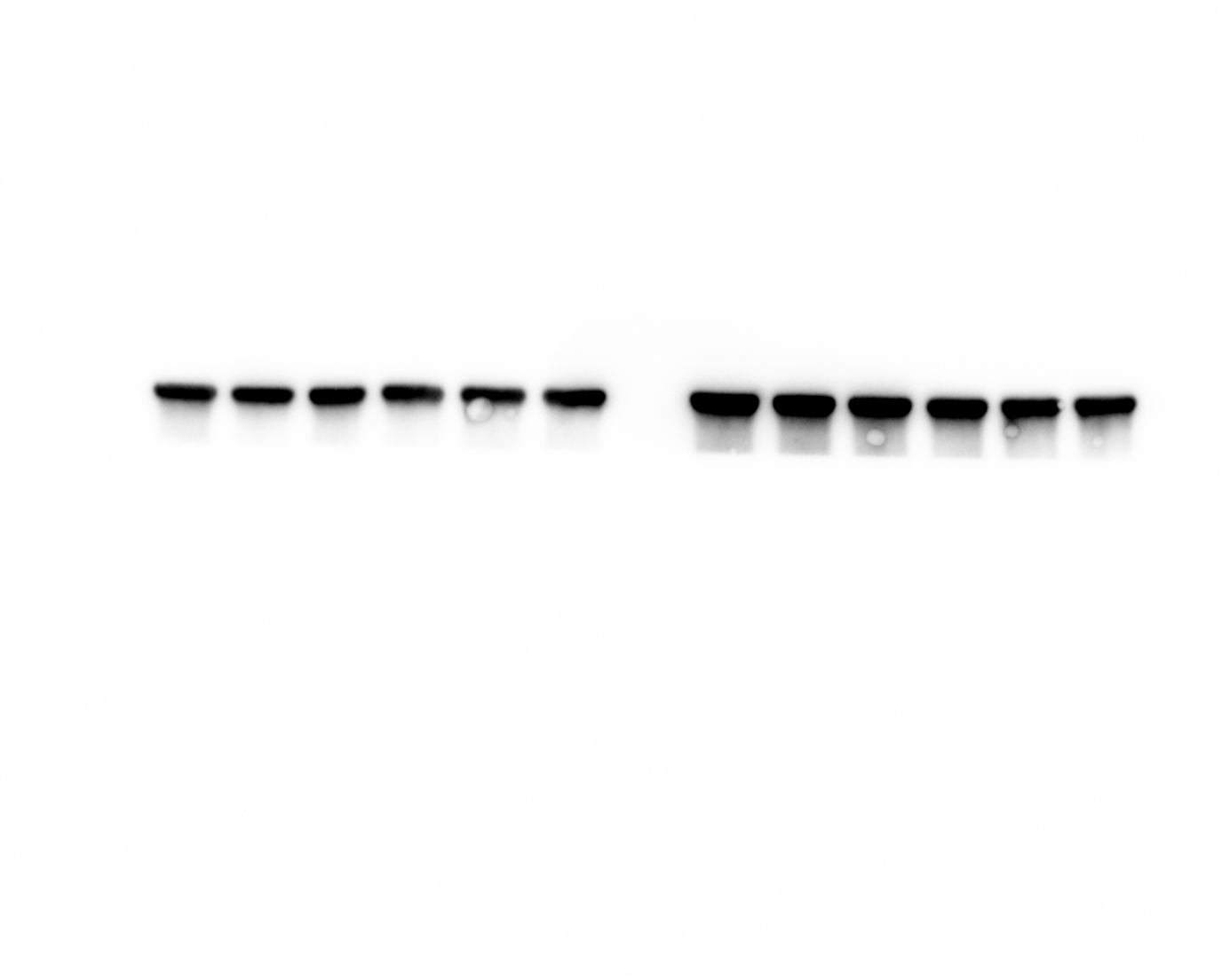

Supplement: Figure 6—figure supplement 1—source data 1. [file elife-69310-fig6-figsupp1-data1.zip › Figure 6-figure supplement 1-source data 1/BAV-TAA patient #3/5-6-B-ACTIN-001-1.Tif]

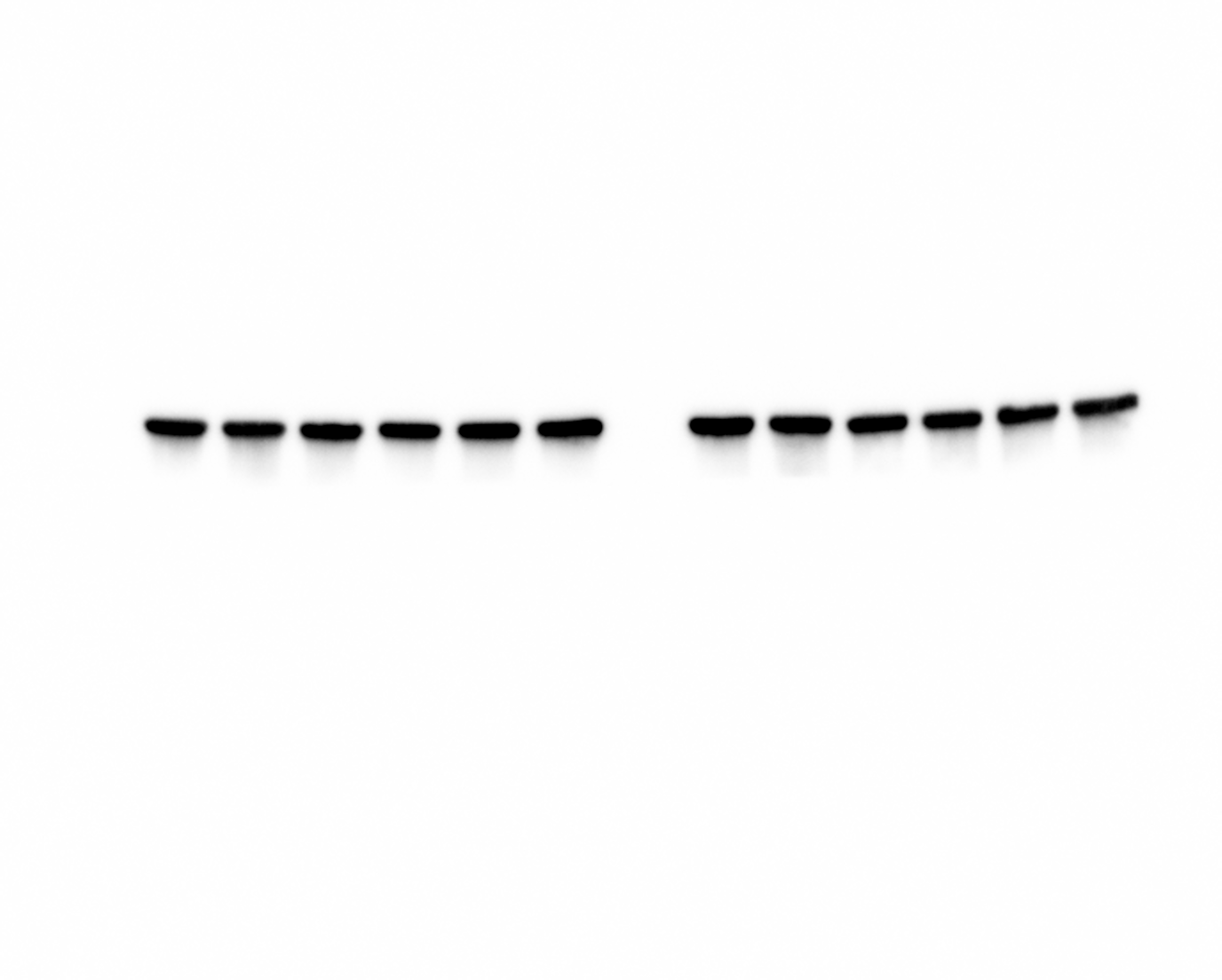

Supplement: Figure 6—figure supplement 1—source data 1. [file elife-69310-fig6-figsupp1-data1.zip › Figure 6-figure supplement 1-source data 1/BAV-TAA patient #3/5-6-B-ACTIN-002-3.Tif]

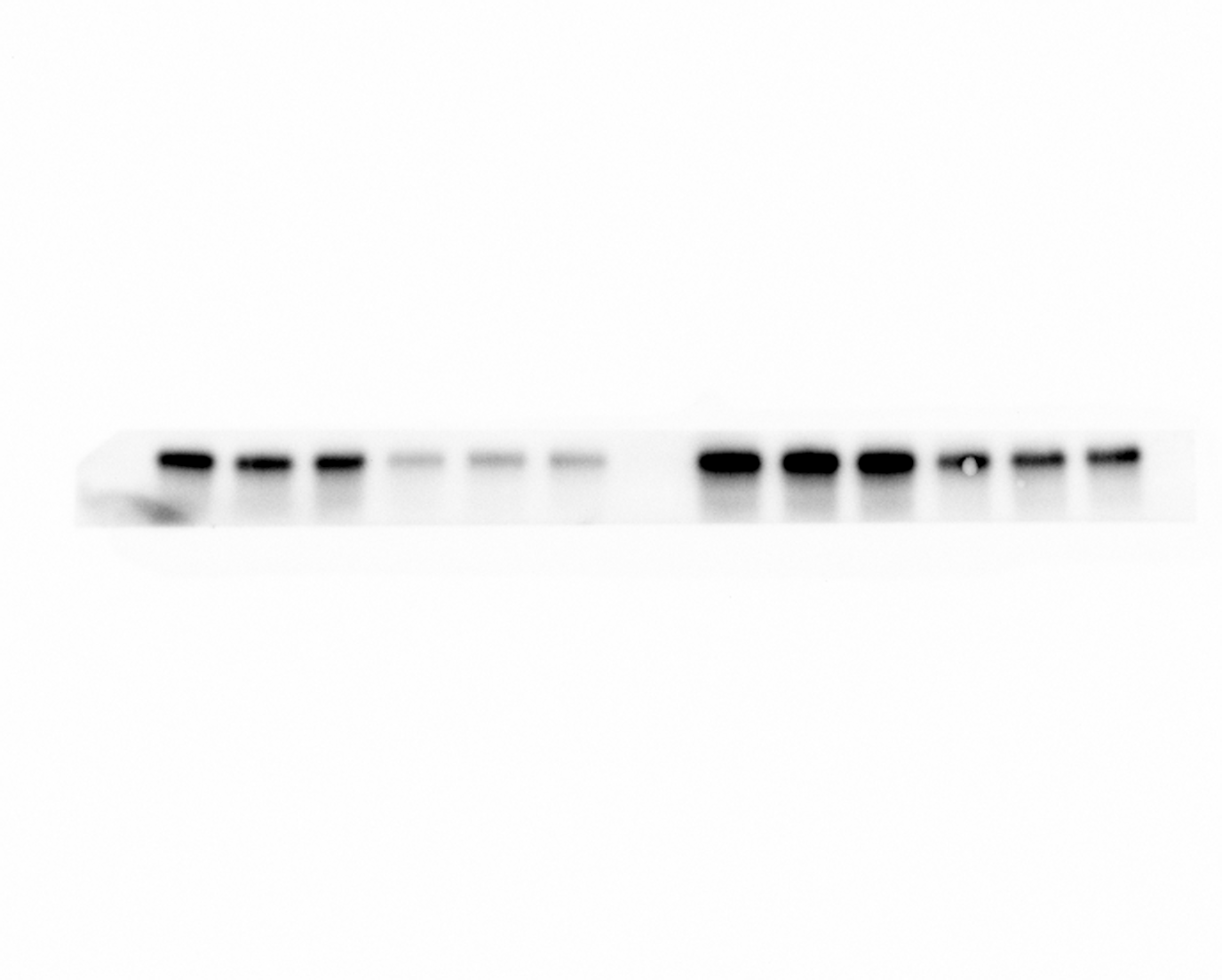

Supplement: Figure 6—figure supplement 1—source data 1. [file elife-69310-fig6-figsupp1-data1.zip › Figure 6-figure supplement 1-source data 1/BAV-TAA patient #3/5-6-CNN1-001-3.Tif]

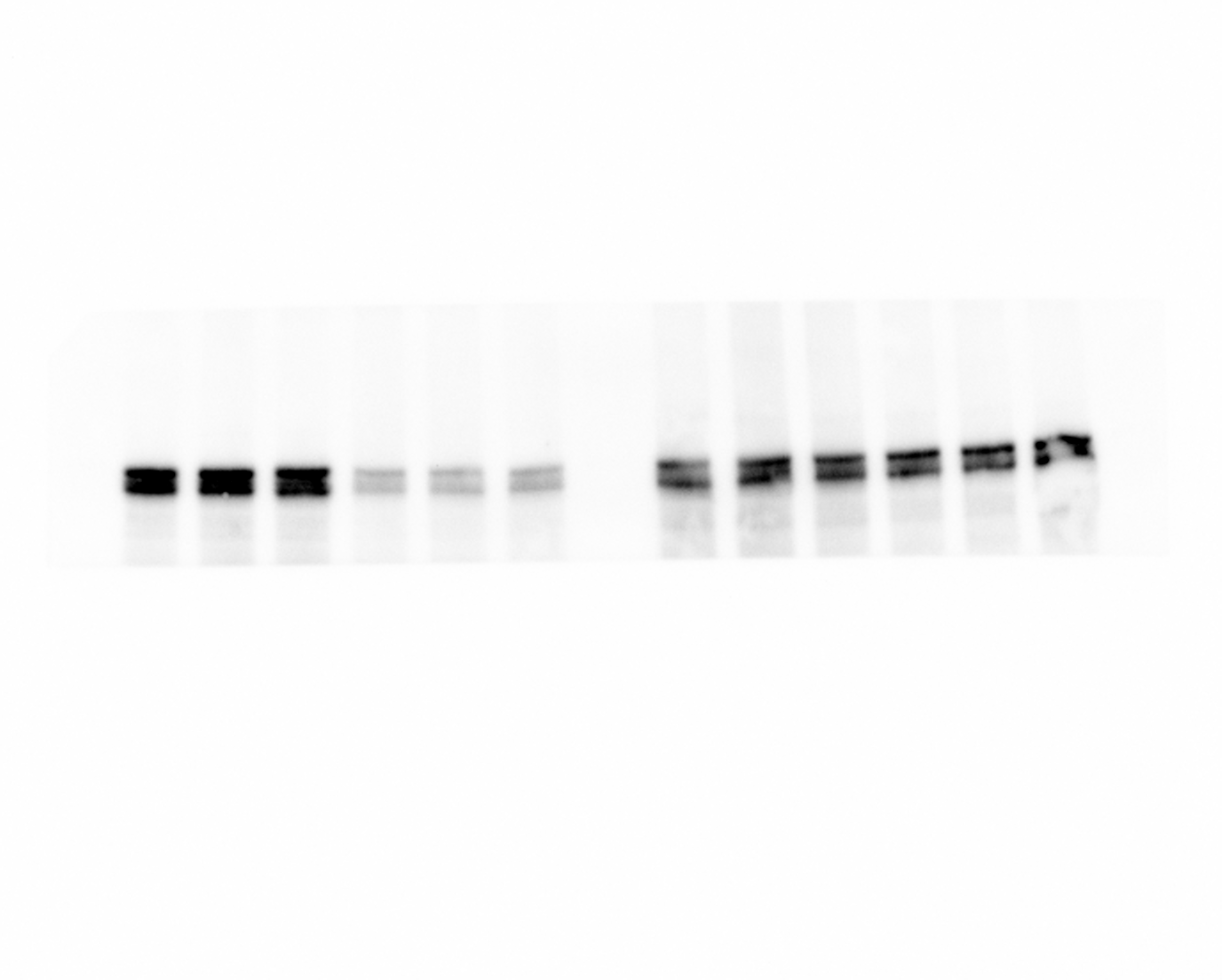

Supplement: Figure 6—figure supplement 1—source data 1. [file elife-69310-fig6-figsupp1-data1.zip › Figure 6-figure supplement 1-source data 1/BAV-TAA patient #3/5-6-DRP1-002-3.Tif]

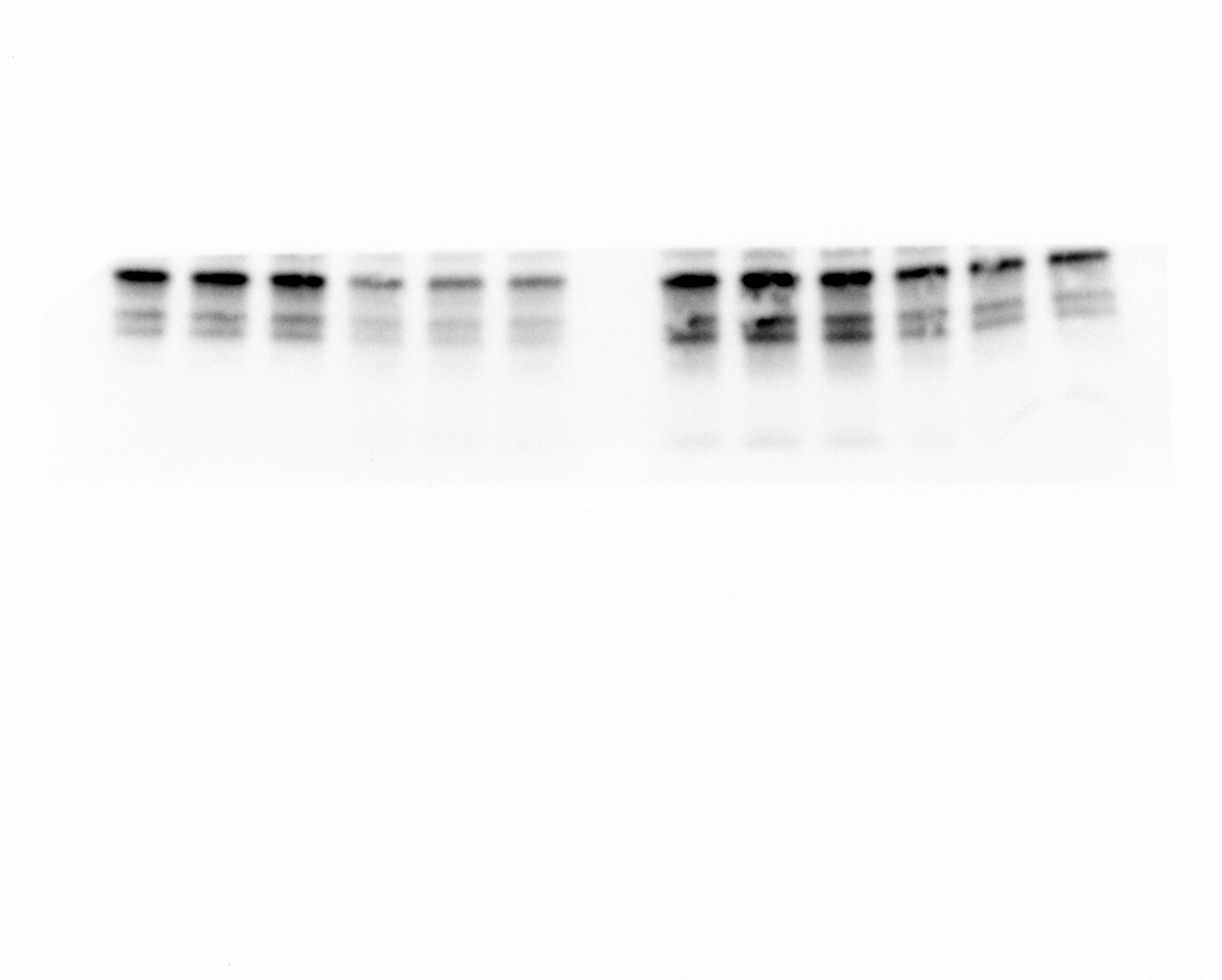

Supplement: Figure 6—figure supplement 1—source data 1. [file elife-69310-fig6-figsupp1-data1.zip › Figure 6-figure supplement 1-source data 1/BAV-TAA patient #3/5-6-MFF-002-3.Tif]

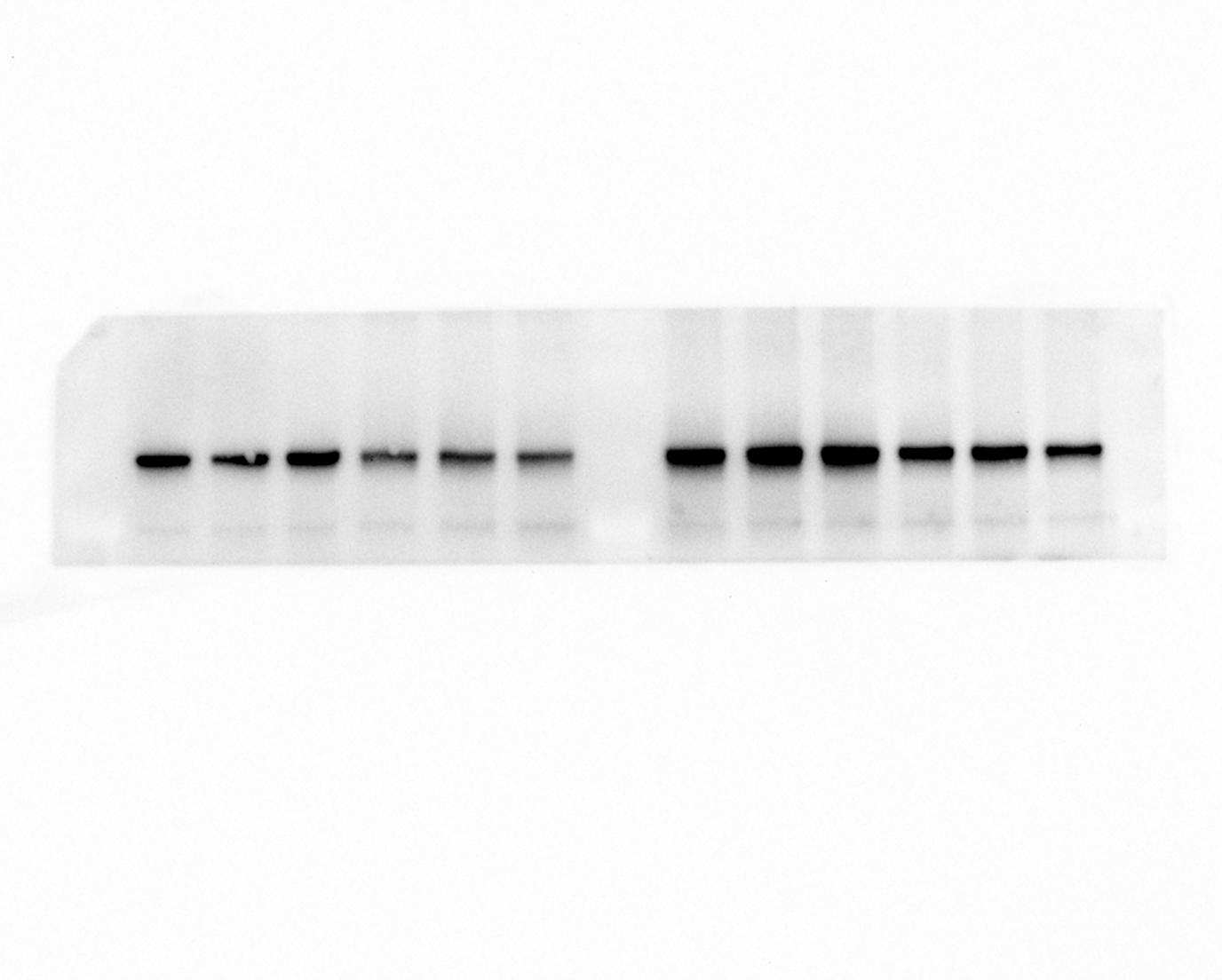

Supplement: Figure 6—figure supplement 1—source data 1. [file elife-69310-fig6-figsupp1-data1.zip › Figure 6-figure supplement 1-source data 1/BAV-TAA patient #3/5-6-MFN1-001-1.Tif]

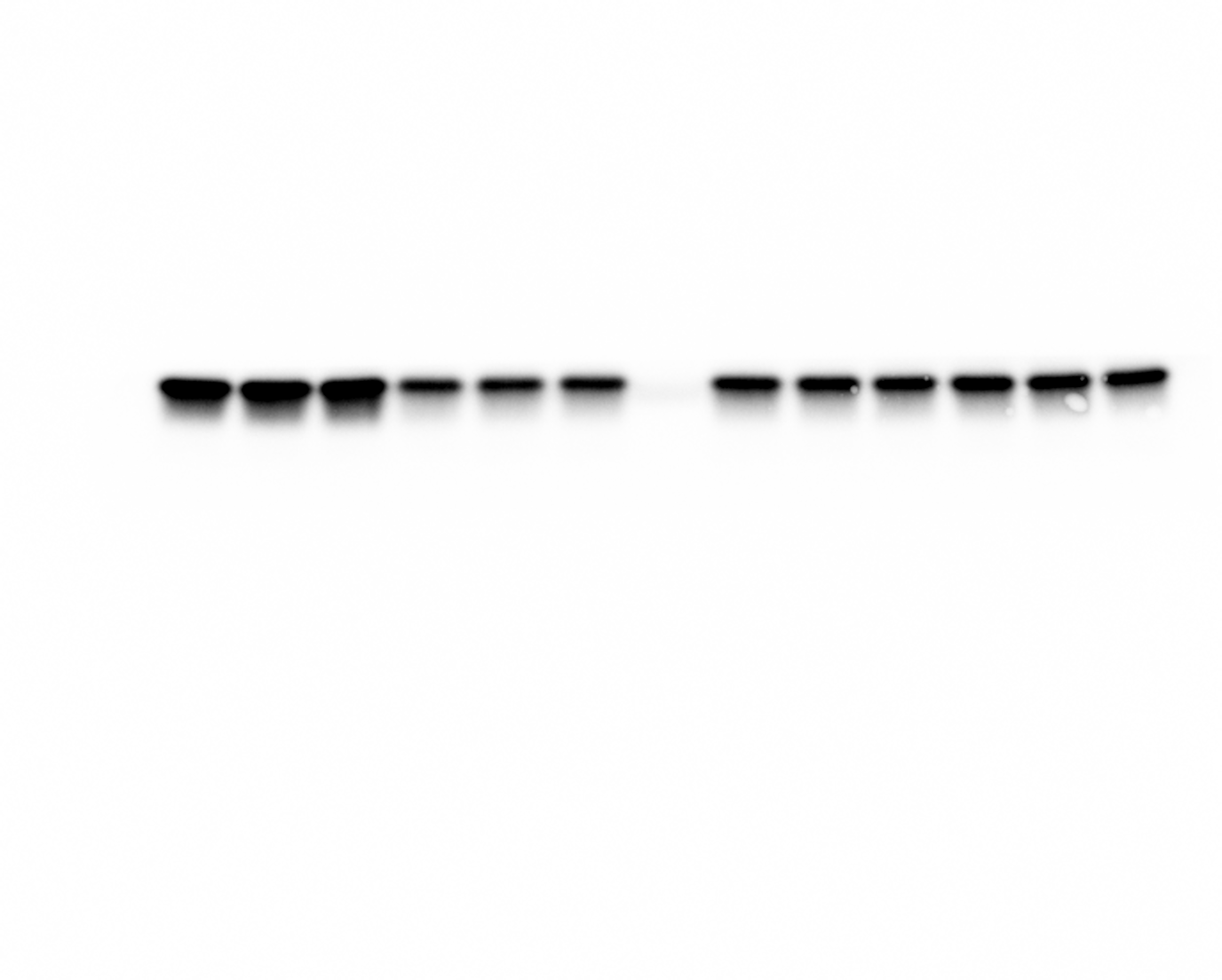

Supplement: Figure 6—figure supplement 1—source data 1. [file elife-69310-fig6-figsupp1-data1.zip › Figure 6-figure supplement 1-source data 1/BAV-TAA patient #3/5-6-SM22-001-3.Tif]

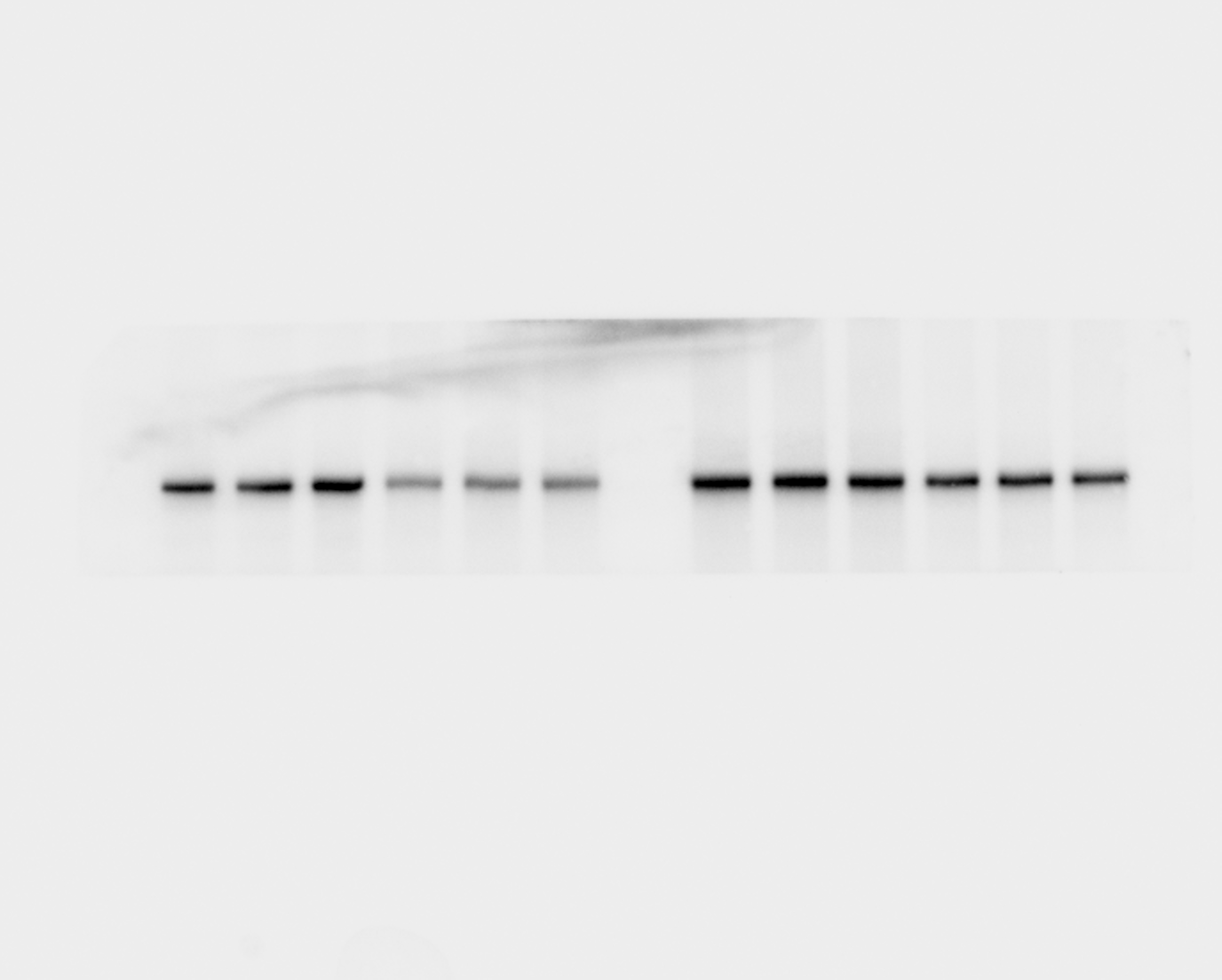

Supplement: Figure 6—figure supplement 1—source data 1. [file elife-69310-fig6-figsupp1-data1.zip › Figure 6-figure supplement 1-source data 1/BAV-TAA patient #3/8bit-5-6-MFN2-003.Tif]

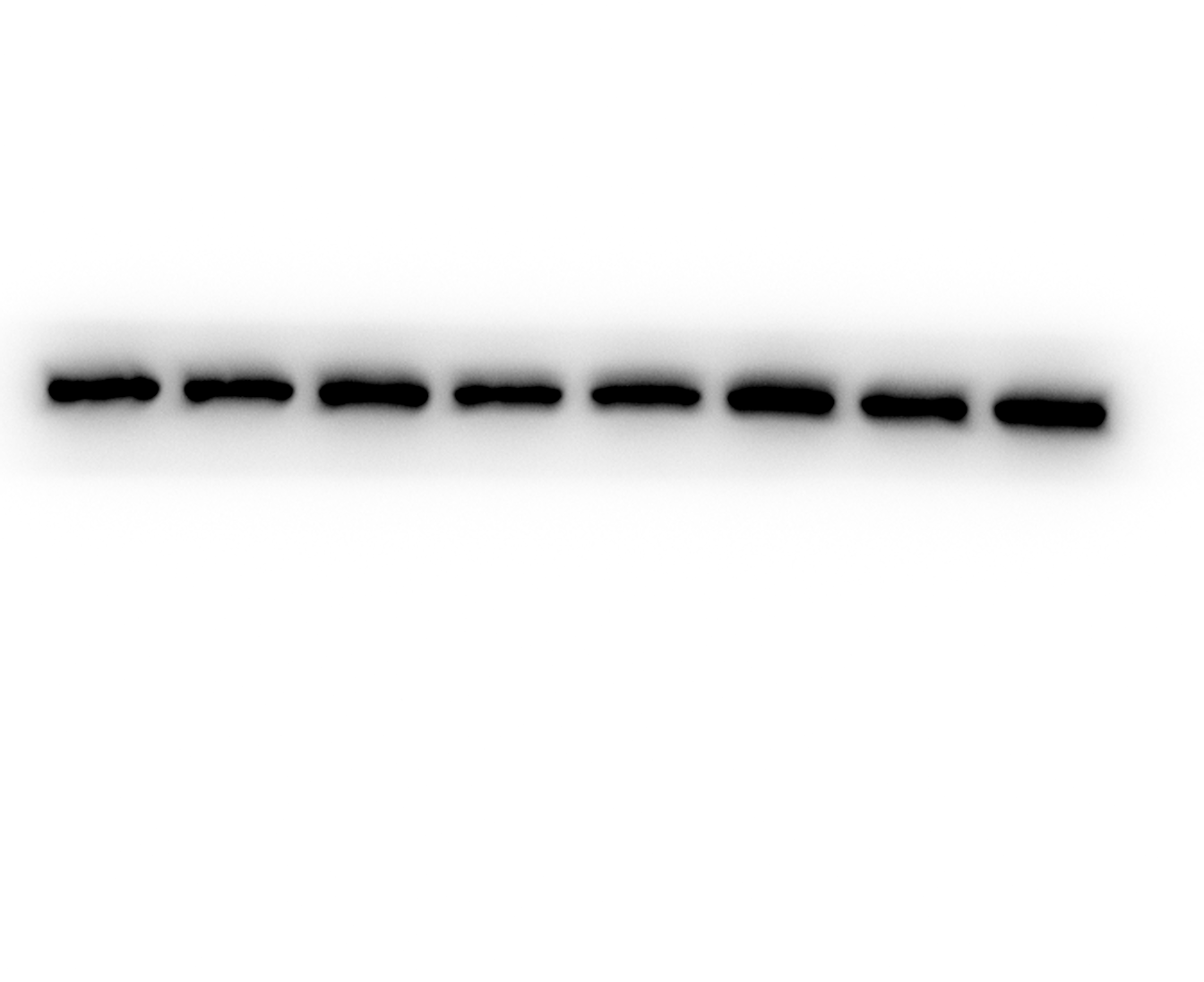

Supplement: Figure 6—figure supplement 1—source data 1. [file elife-69310-fig6-figsupp1-data1.zip › Figure 6-figure supplement 1-source data 1/non-diseased patient #1/B-ACTIN-002-1.Tif]

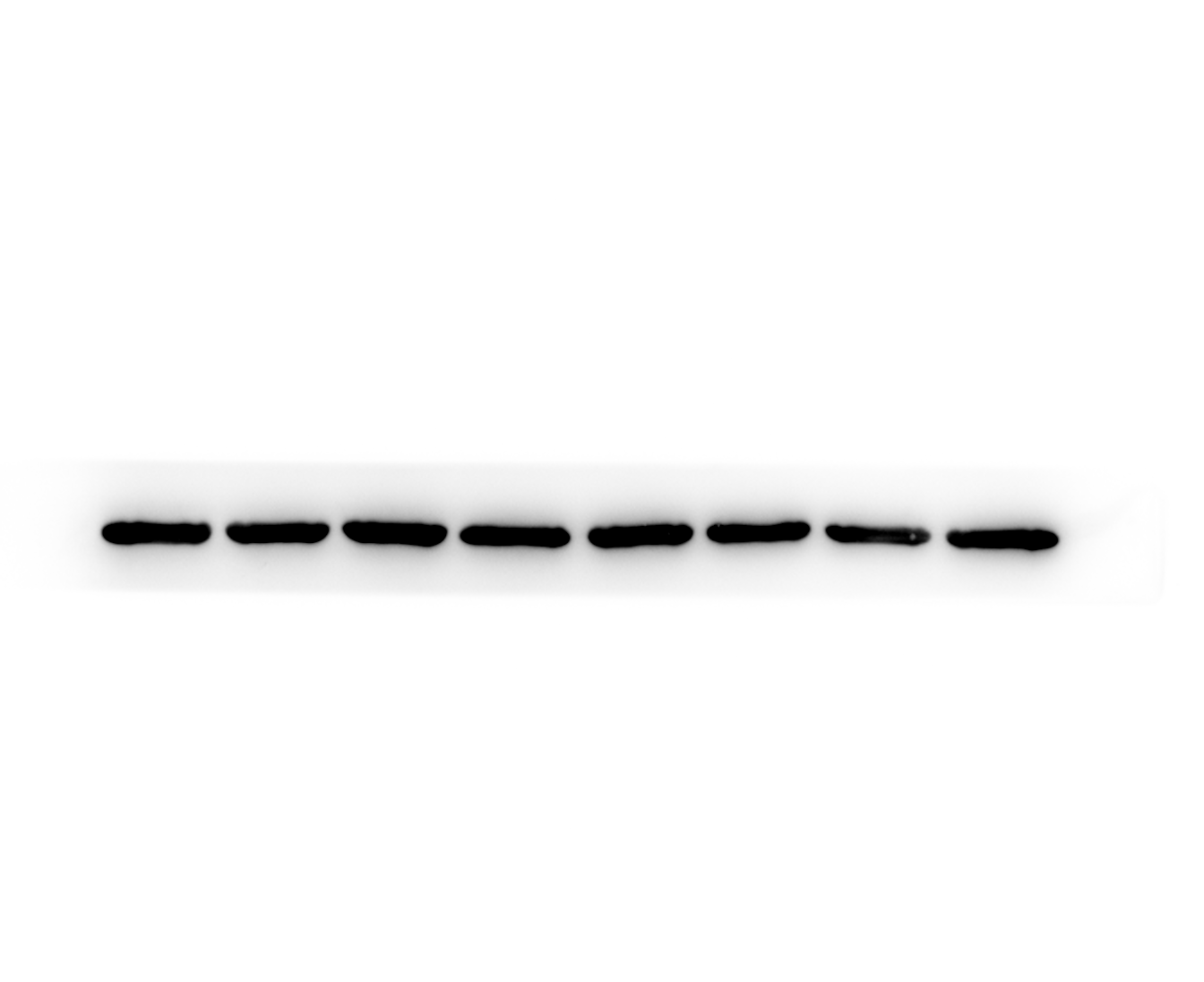

Supplement: Figure 6—figure supplement 1—source data 1. [file elife-69310-fig6-figsupp1-data1.zip › Figure 6-figure supplement 1-source data 1/non-diseased patient #1/B-ACTIN-003-3.Tif]

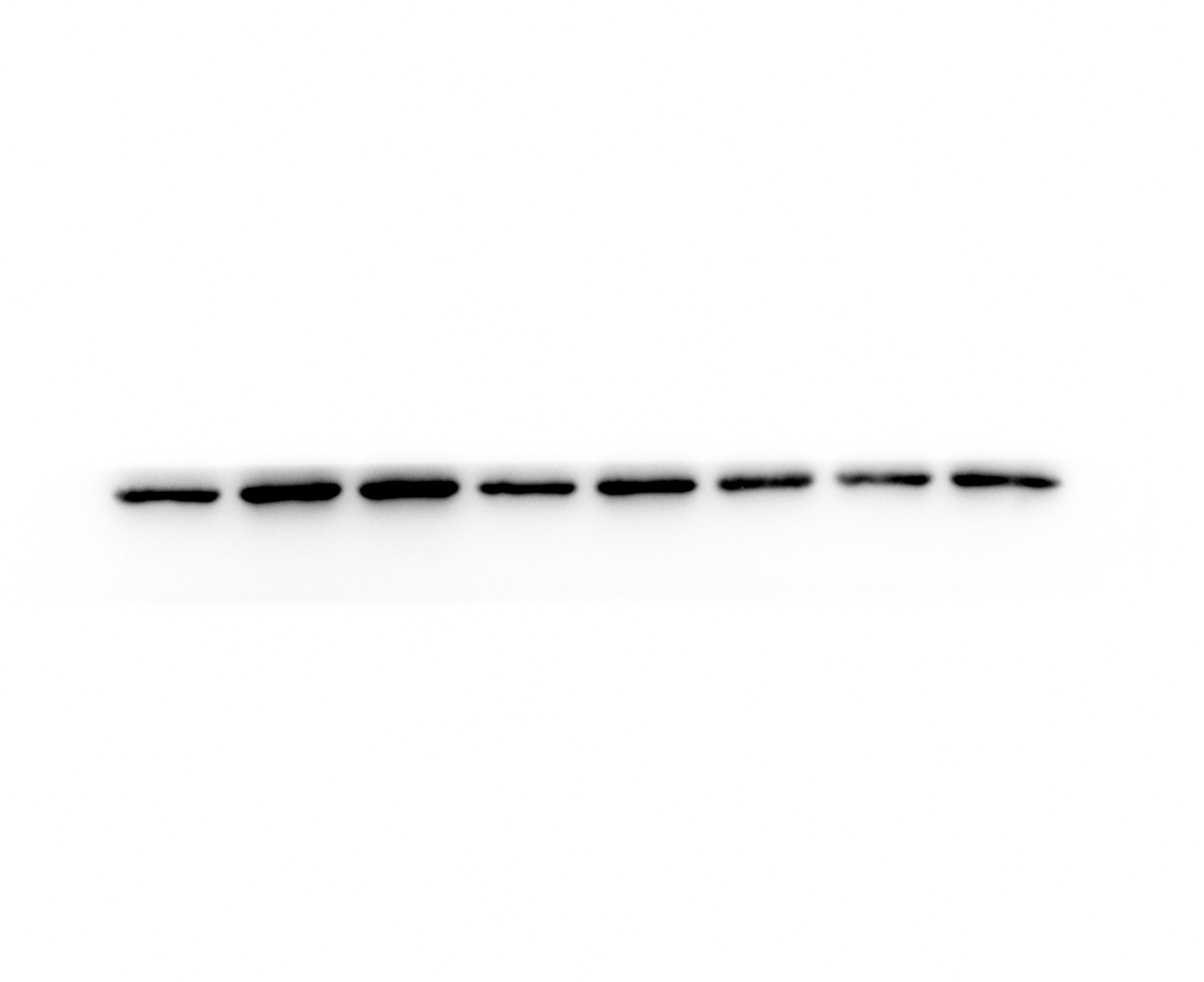

Supplement: Figure 6—figure supplement 1—source data 1. [file elife-69310-fig6-figsupp1-data1.zip › Figure 6-figure supplement 1-source data 1/non-diseased patient #1/CNN1-002-1.Tif]

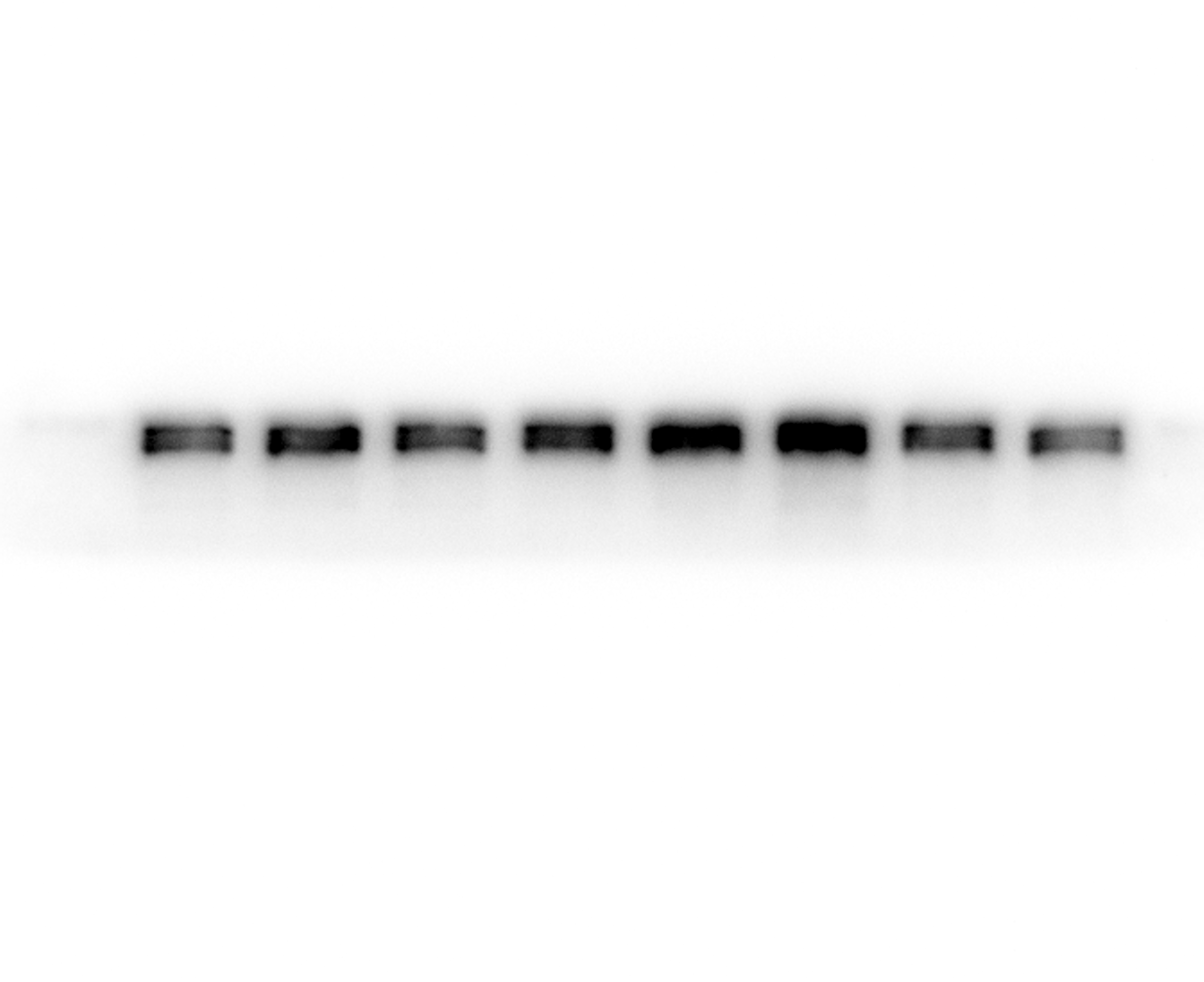

Supplement: Figure 6—figure supplement 1—source data 1. [file elife-69310-fig6-figsupp1-data1.zip › Figure 6-figure supplement 1-source data 1/non-diseased patient #1/DRP1-001-3.Tif]

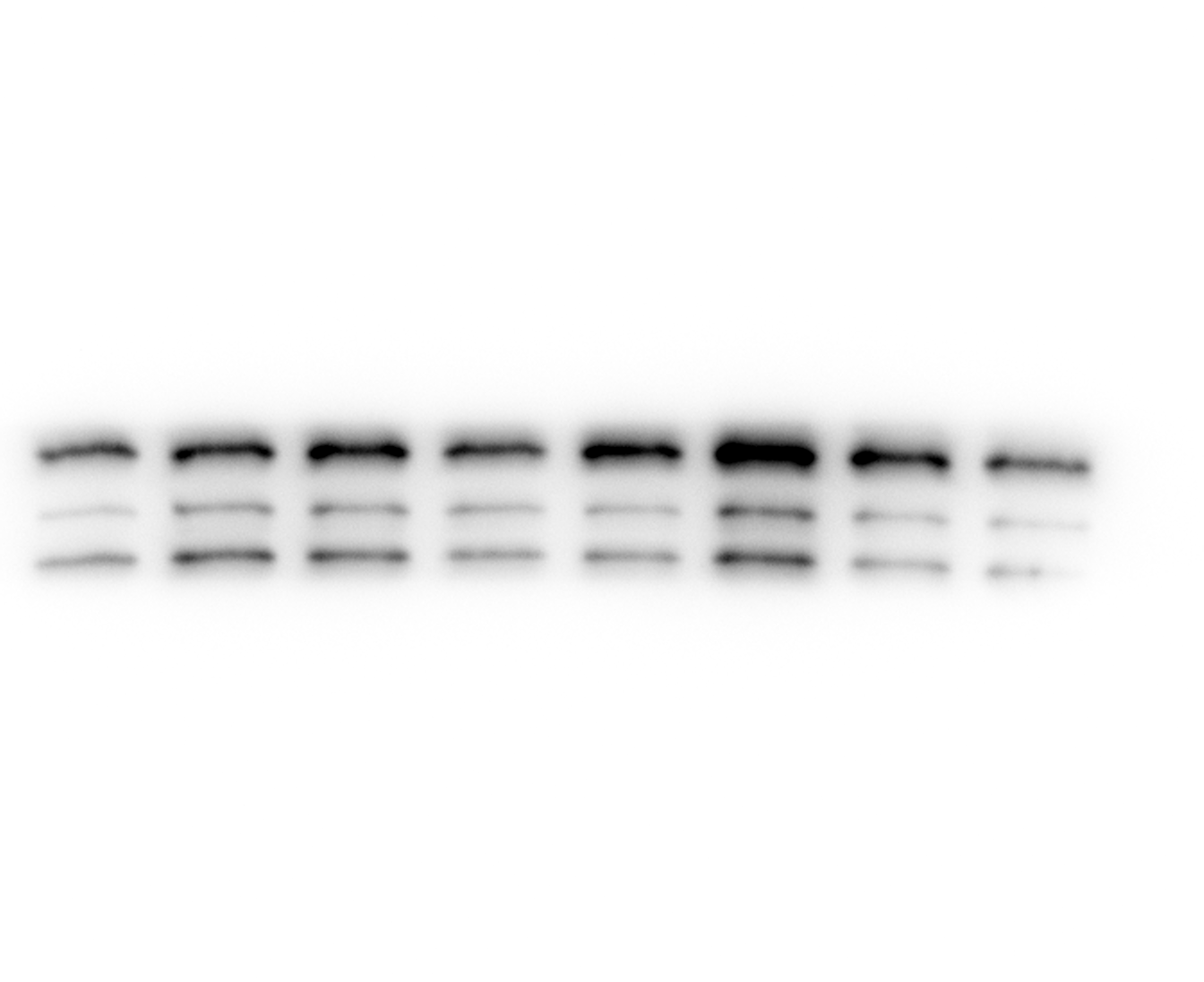

Supplement: Figure 6—figure supplement 1—source data 1. [file elife-69310-fig6-figsupp1-data1.zip › Figure 6-figure supplement 1-source data 1/non-diseased patient #1/MFF-001-1.Tif]

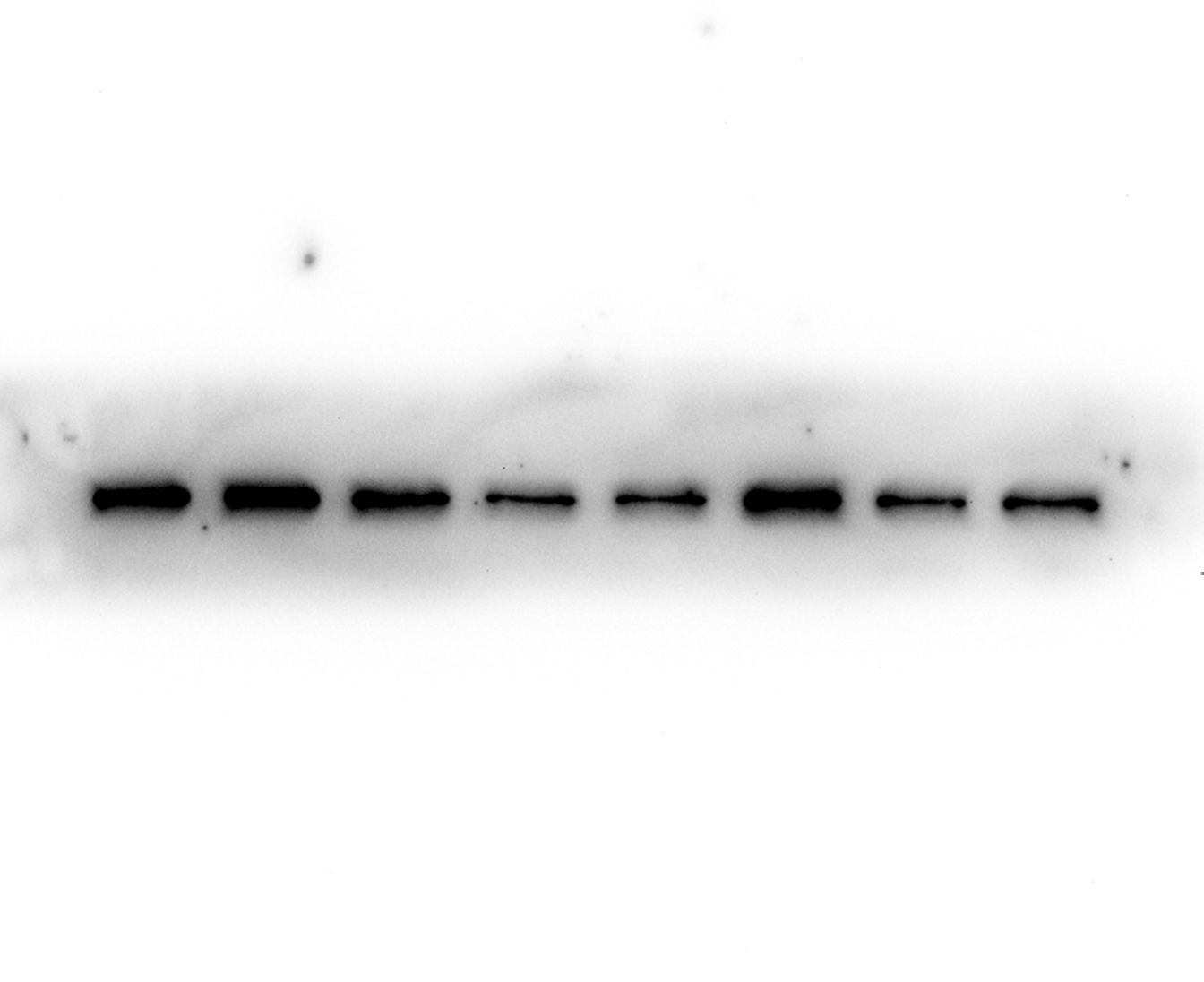

Supplement: Figure 6—figure supplement 1—source data 1. [file elife-69310-fig6-figsupp1-data1.zip › Figure 6-figure supplement 1-source data 1/non-diseased patient #1/MFN1-003-1.Tif]

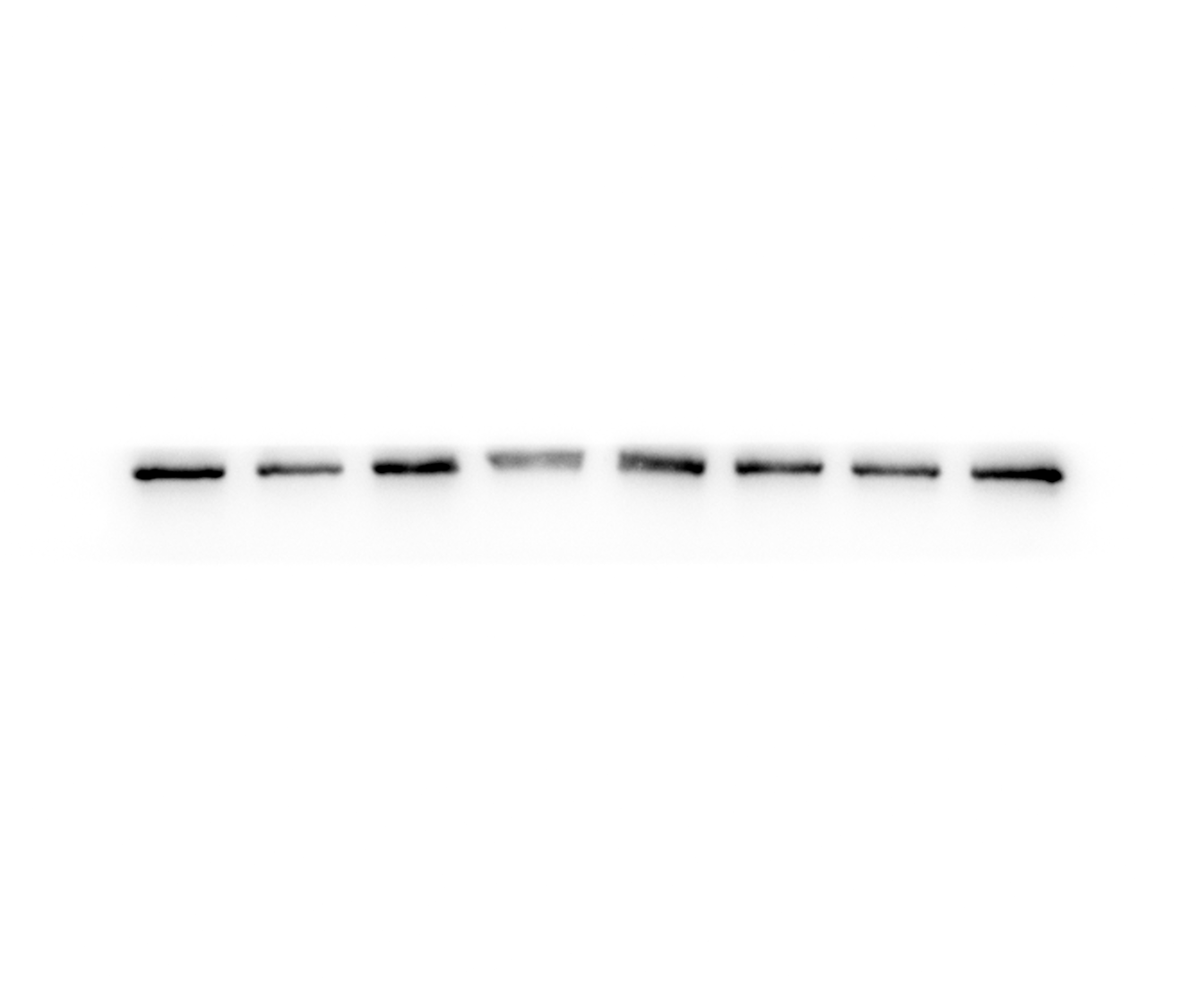

Supplement: Figure 6—figure supplement 1—source data 1. [file elife-69310-fig6-figsupp1-data1.zip › Figure 6-figure supplement 1-source data 1/non-diseased patient #1/MFN2-003-1.Tif]

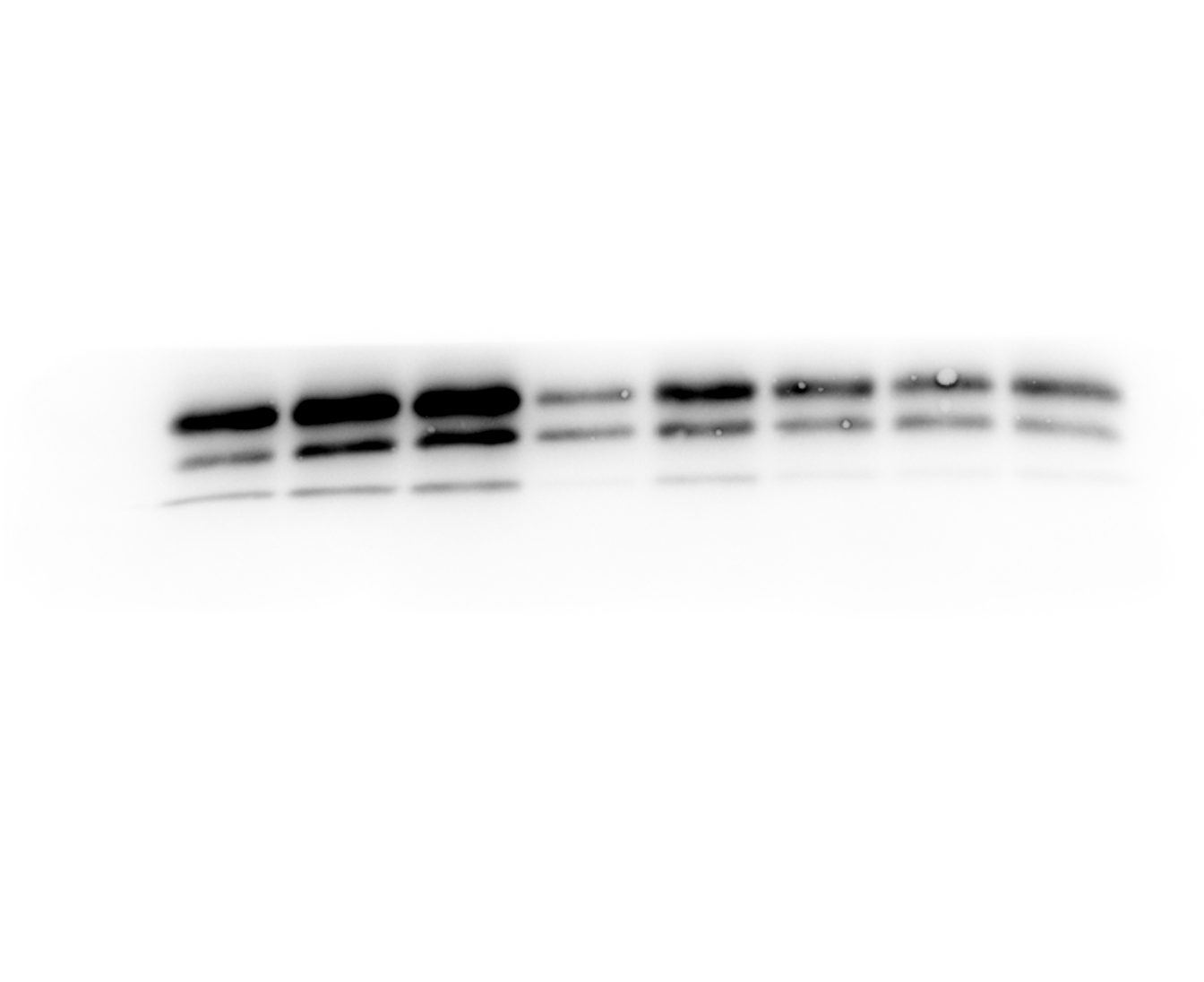

Supplement: Figure 6—figure supplement 1—source data 1. [file elife-69310-fig6-figsupp1-data1.zip › Figure 6-figure supplement 1-source data 1/non-diseased patient #1/SM22-002-3.Tif]

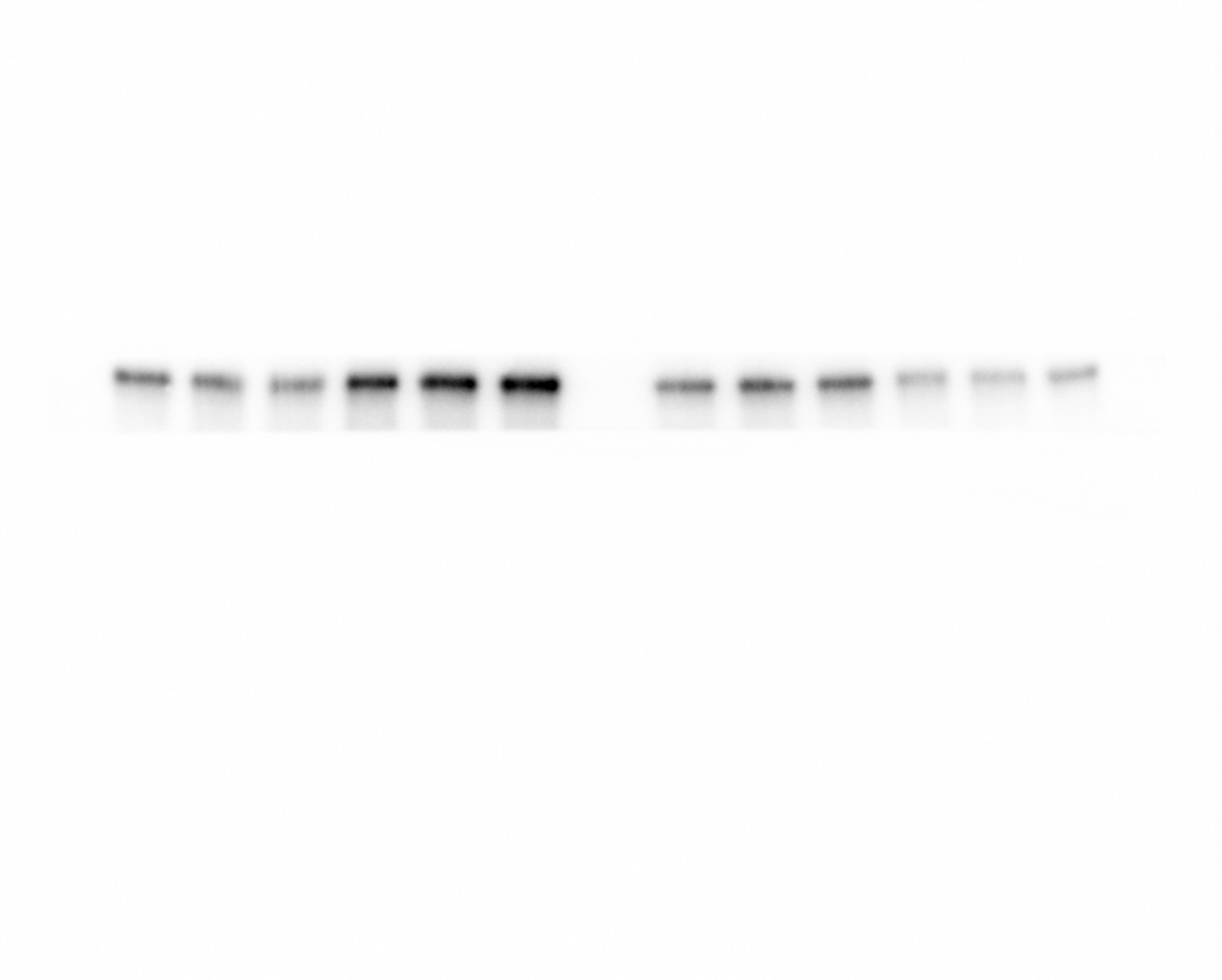

Supplement: Figure 6—figure supplement 1—source data 1. [file elife-69310-fig6-figsupp1-data1.zip › Figure 6-figure supplement 1-source data 1/non-diseased patient #3/3-4-CNN1-001-3.Tif]

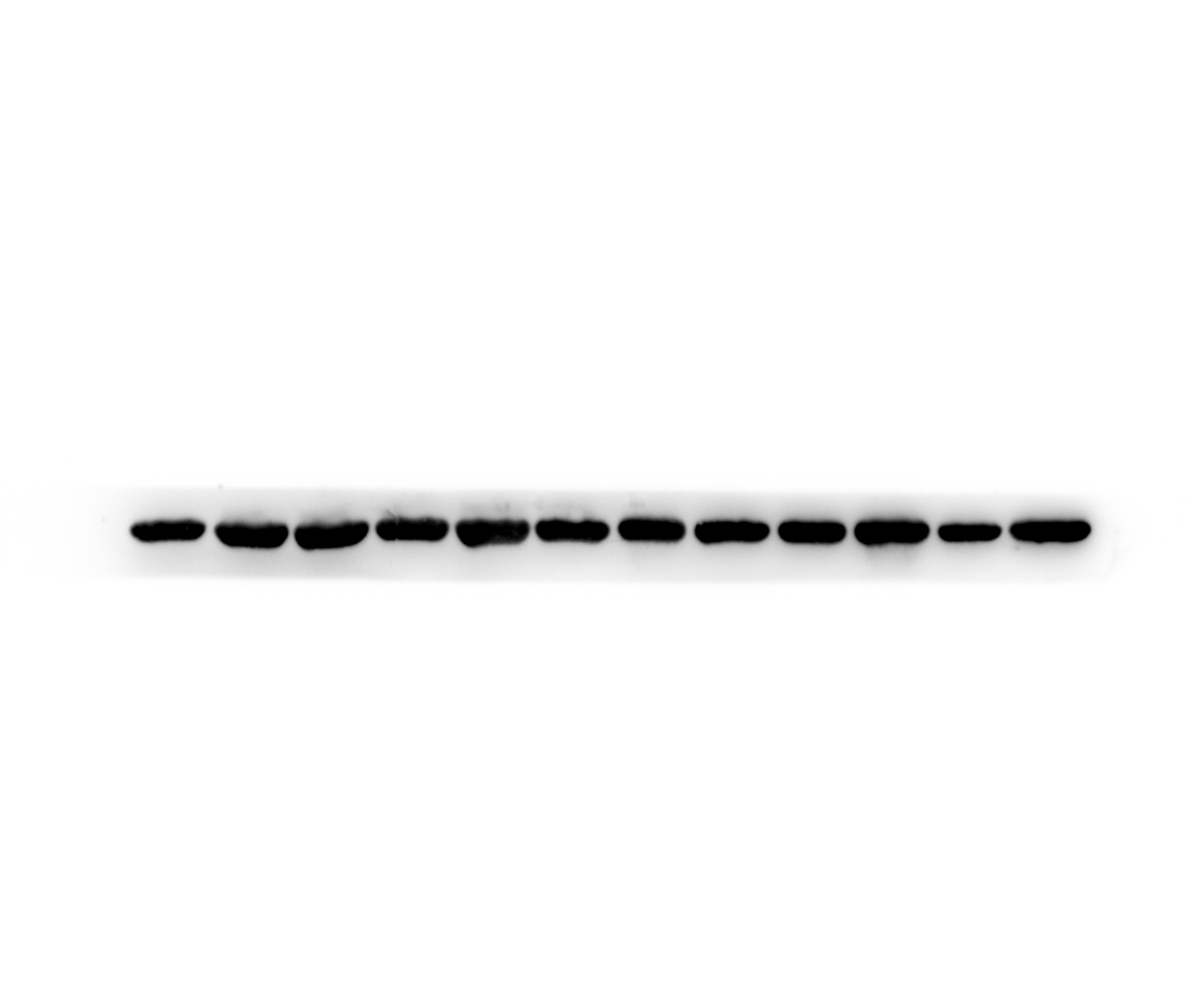

Supplement: Figure 6—figure supplement 1—source data 2. [file elife-69310-fig6-figsupp1-data2.zip › Figure 6-figure supplement 1-source data 2/B-ACTIN-003-2.Tif]

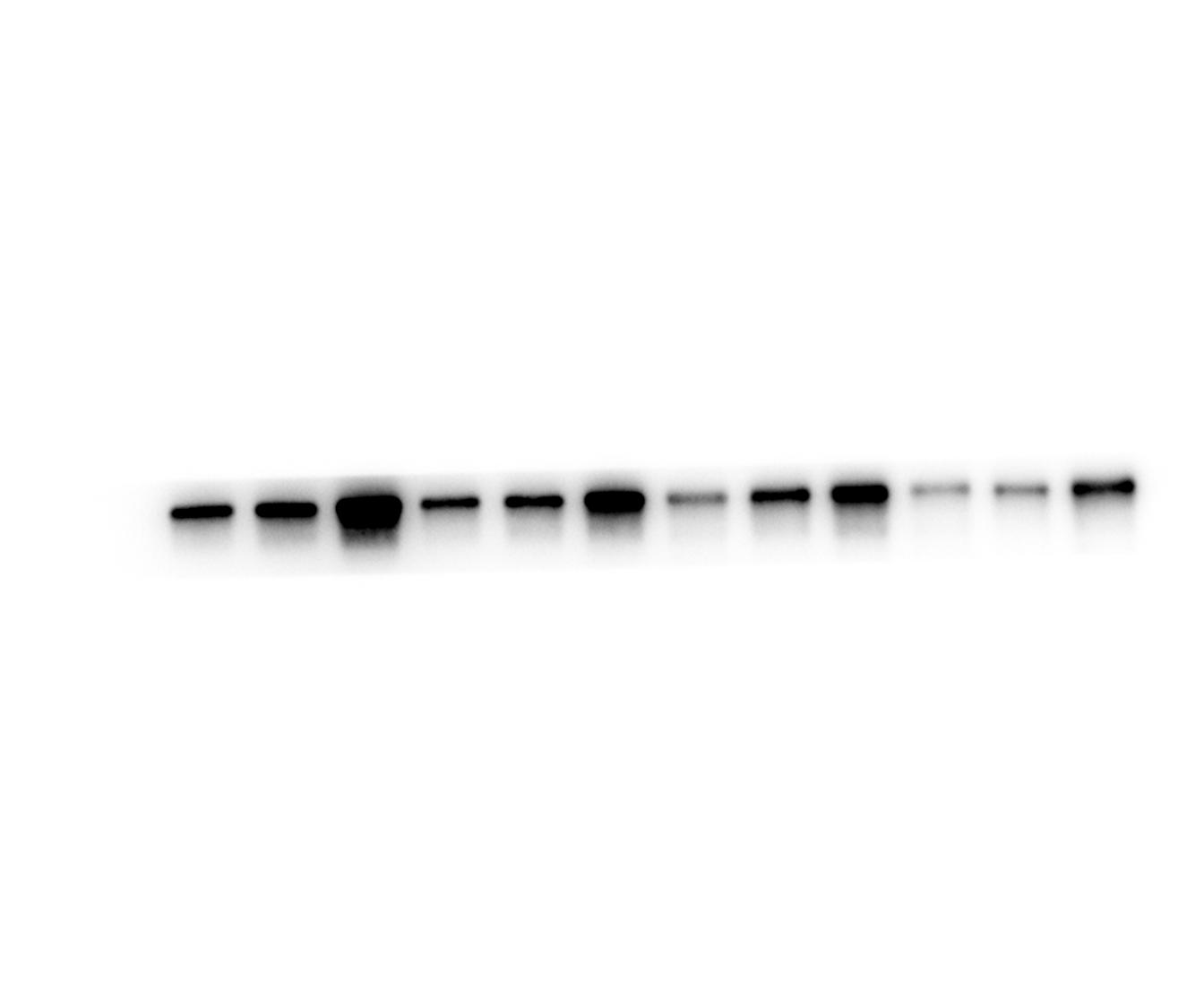

Supplement: Figure 6—figure supplement 1—source data 2. [file elife-69310-fig6-figsupp1-data2.zip › Figure 6-figure supplement 1-source data 2/CNN1-002-3.Tif]

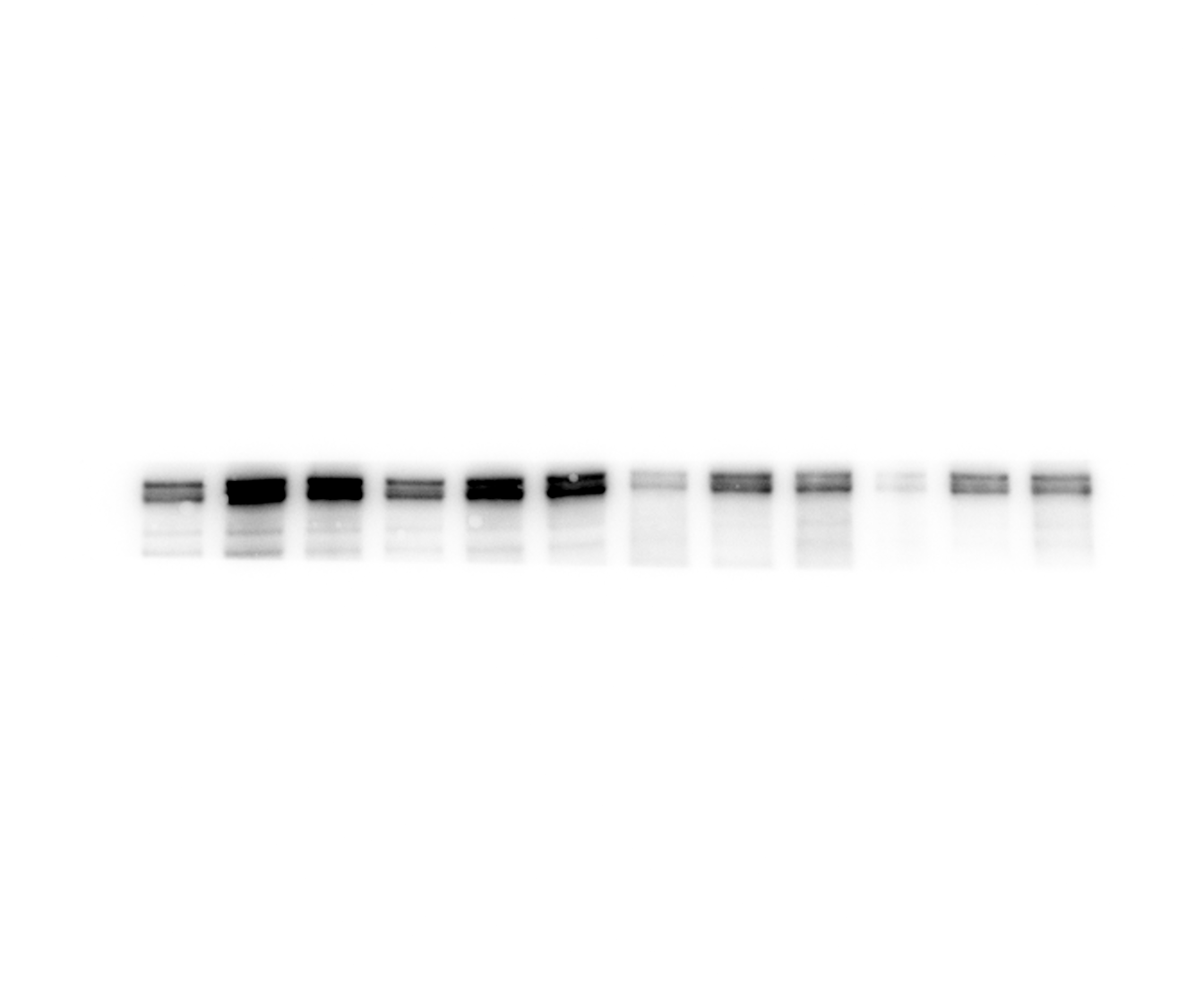

Supplement: Figure 6—figure supplement 1—source data 2. [file elife-69310-fig6-figsupp1-data2.zip › Figure 6-figure supplement 1-source data 2/DRP-1-001-1.Tif]

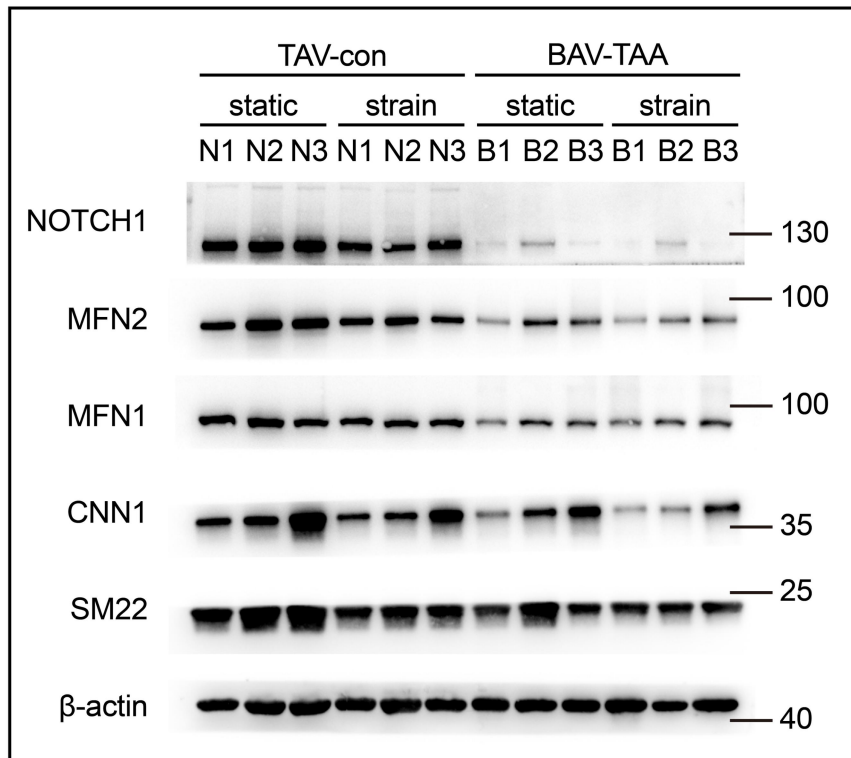

Supplement: Figure 6—figure supplement 1—source data 2. [file elife-69310-fig6-figsupp1-data2.zip › Figure 6-figure supplement 1-source data 2/labeled uncropped WB .pdf]

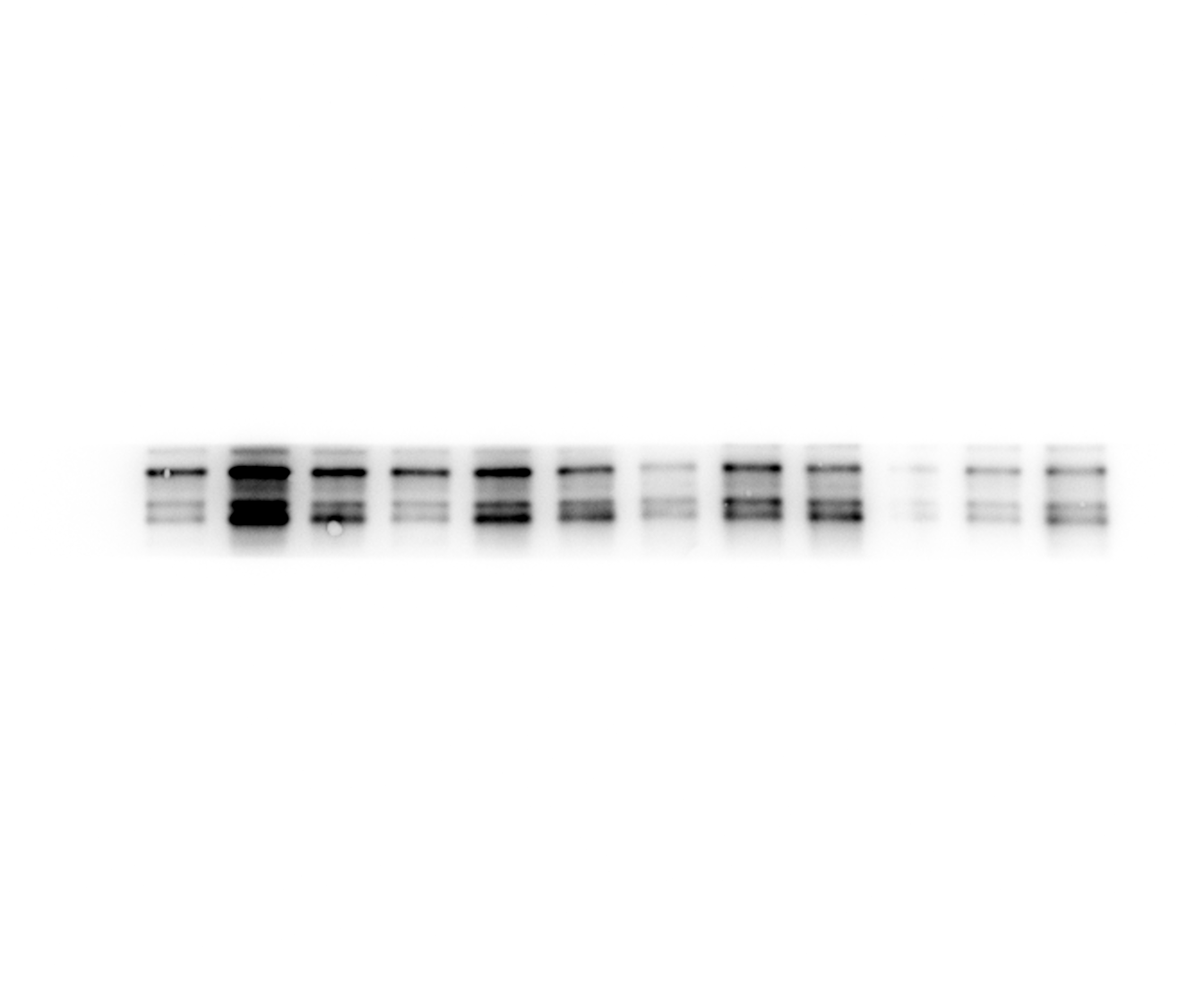

Supplement: Figure 6—figure supplement 1—source data 2. [file elife-69310-fig6-figsupp1-data2.zip › Figure 6-figure supplement 1-source data 2/MFF-001-3.Tif]

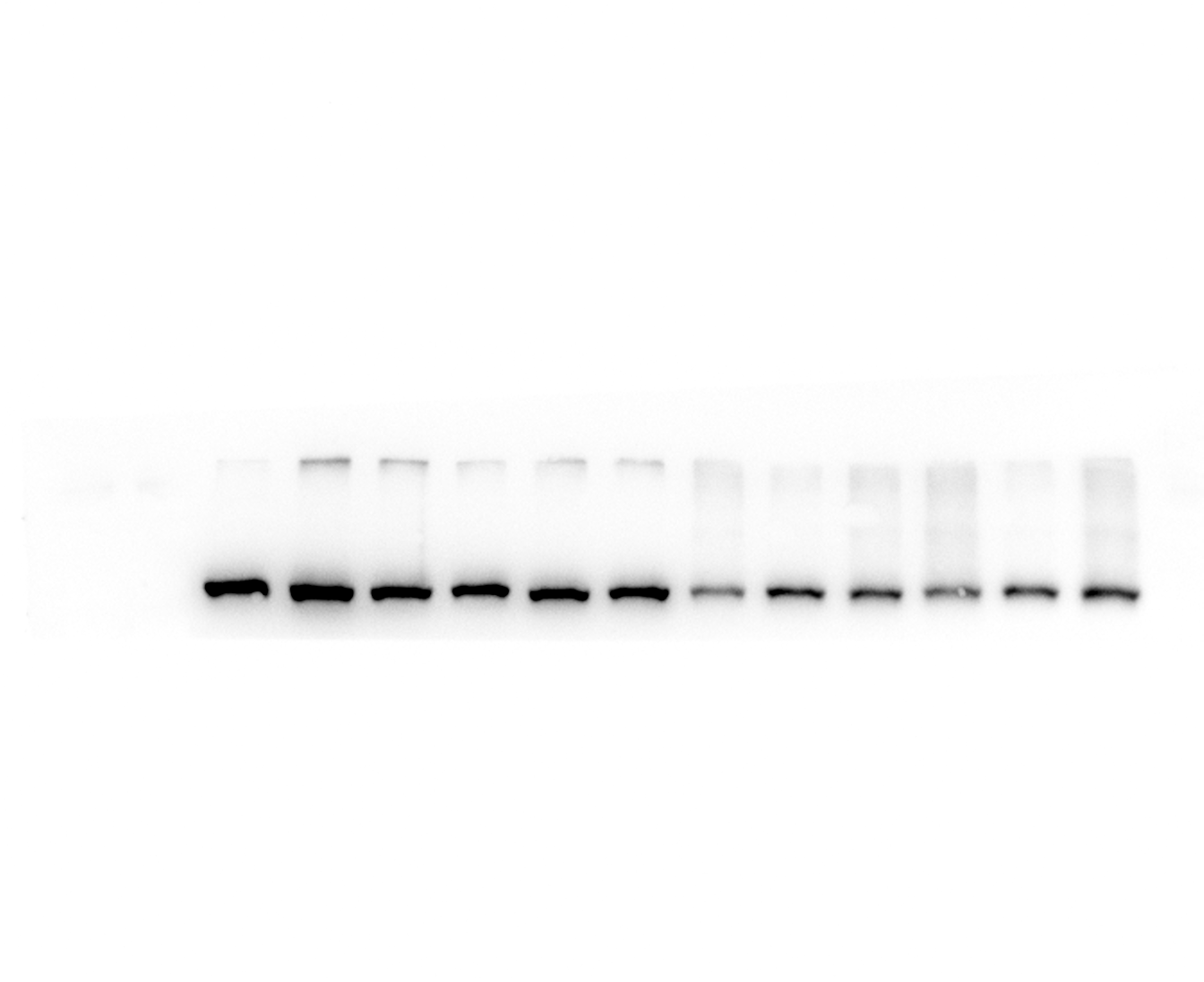

Supplement: Figure 6—figure supplement 1—source data 2. [file elife-69310-fig6-figsupp1-data2.zip › Figure 6-figure supplement 1-source data 2/MFN-1-003-3.Tif]

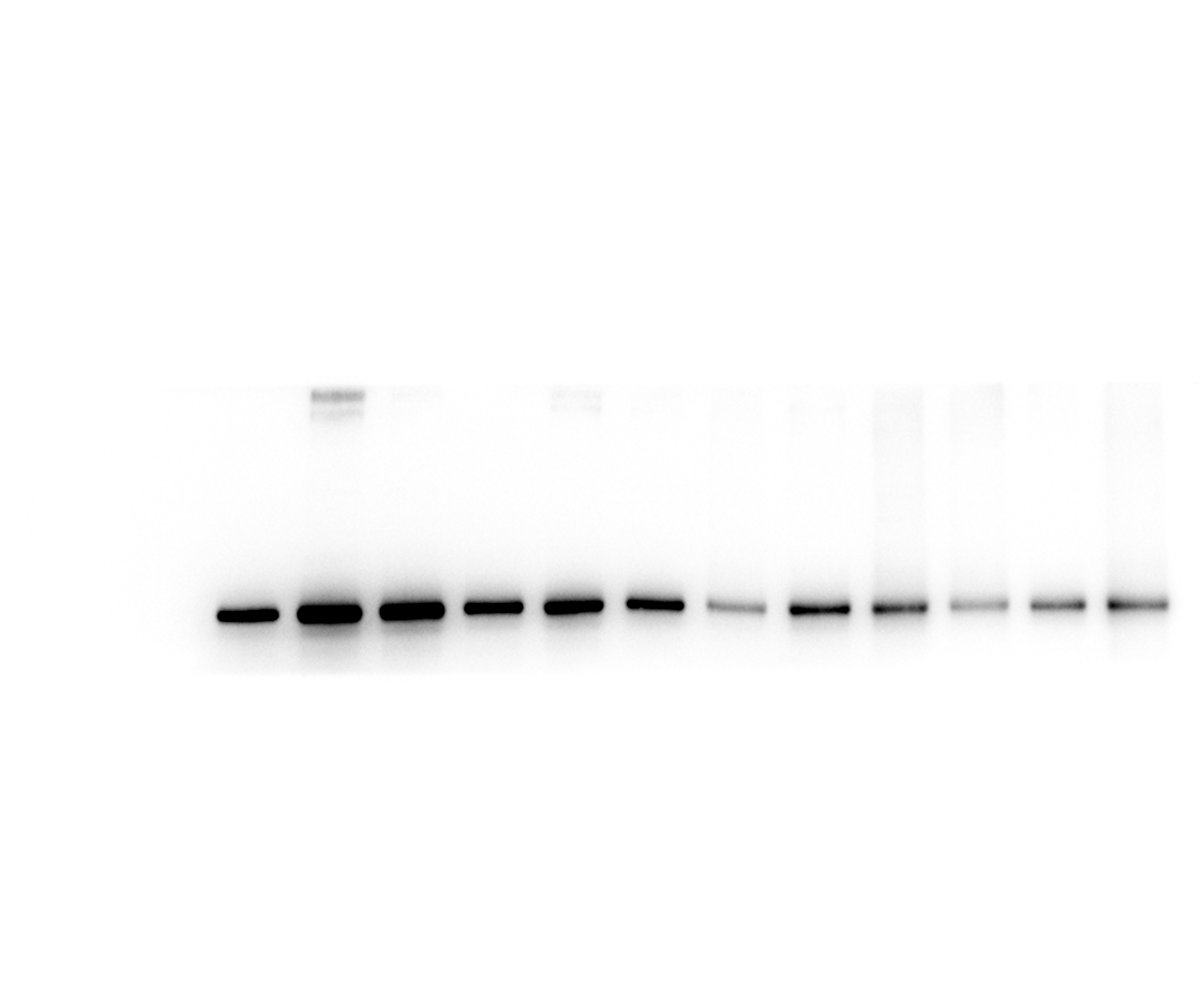

Supplement: Figure 6—figure supplement 1—source data 2. [file elife-69310-fig6-figsupp1-data2.zip › Figure 6-figure supplement 1-source data 2/MFN-2-002-3.Tif]

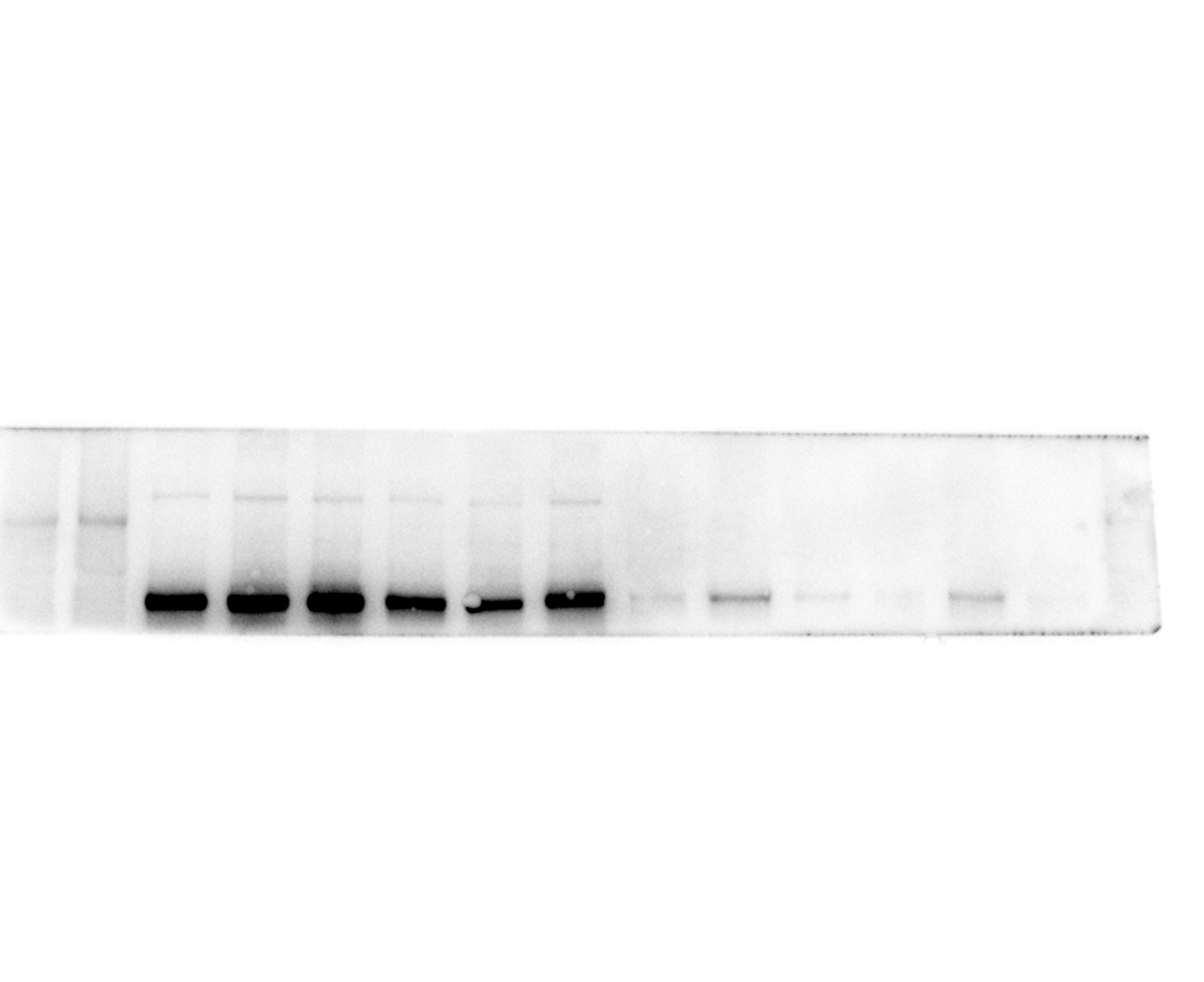

Supplement: Figure 6—figure supplement 1—source data 2. [file elife-69310-fig6-figsupp1-data2.zip › Figure 6-figure supplement 1-source data 2/NOTCH-1-001-3.Tif]

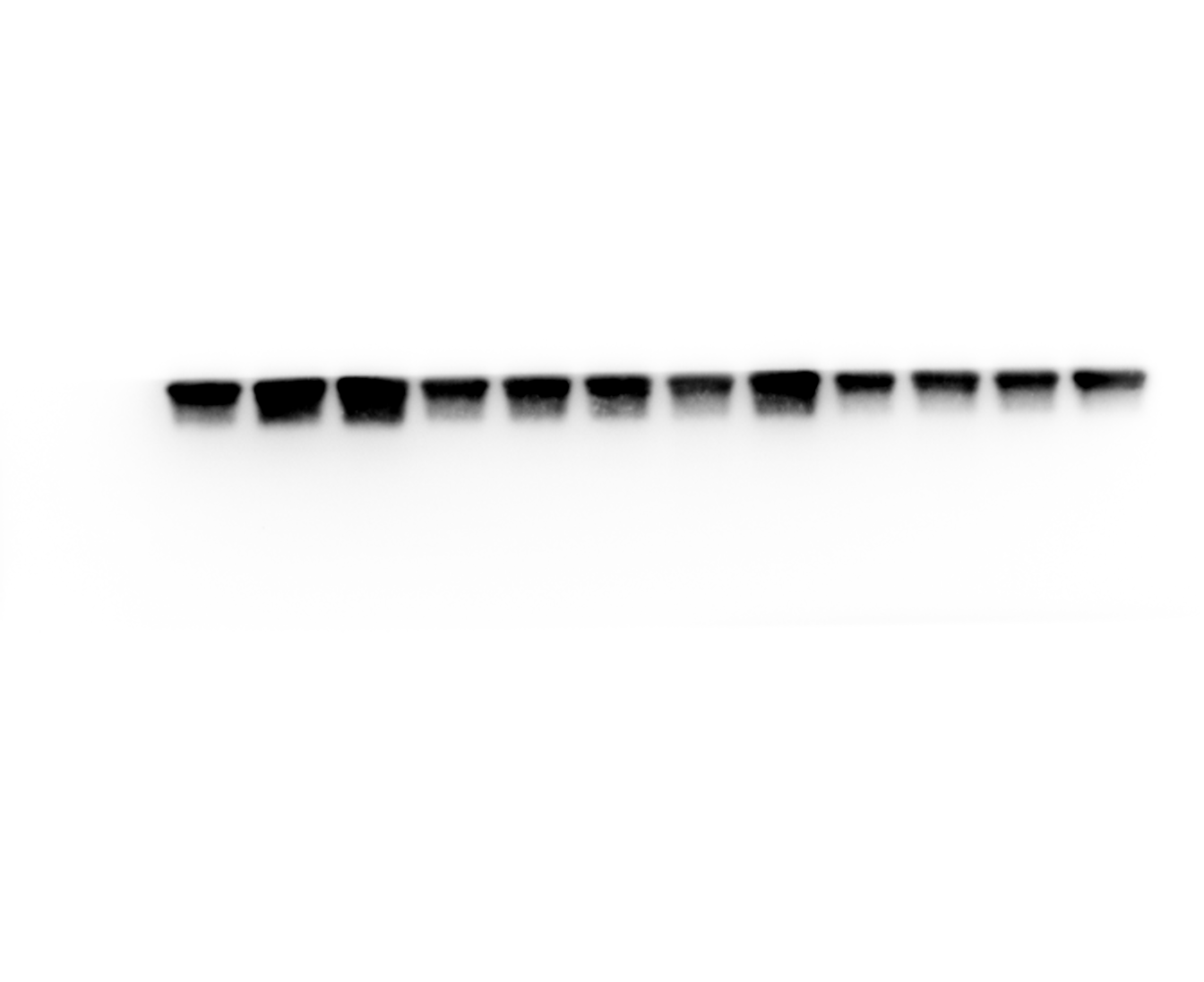

Supplement: Figure 6—figure supplement 1—source data 2. [file elife-69310-fig6-figsupp1-data2.zip › Figure 6-figure supplement 1-source data 2/SM22-002-1.Tif]
