# Supplementary material for: Aorta smooth muscle-on-a-chip reveals impaired mitochondrial dynamics as a therapeutic target for aortic aneurysm in bicuspid aortic valve disease
Source: eLife. 2021 Sep 6;10:e69310. doi: 10.7554/eLife.69310 (PMC8451027; doi:10.7554/eLife.69310)
Supplement: Supplementary file 1. — (a) Clinical characteristics of the patients. (b) Primary antibodies used for western blotting and immunohistochemistry. (c) The enriched canonical pathways identified by ingenuity pathway analysis (IPA). (d) Differential protein expression of mitochondrial dysfunction and oxidative phosphorylation pathways between non-diseased and BAV-TAA tissues. (e) Clinical Characteristics of the p-HAoSMCs from BAV-TAA patients. [file elife-69310-supp1.doc]

**Aorta-on-a-chip reveals impaired mitochondrial dynamics as a therapeutic target for aortic aneurysm in bicuspid aortic valve disease**

Mieradilijiang Abudupataer^1^, Shichao Zhu^1^, Shiqiang Yan^2^, Kehua Xu^2^, Jingjing Zhang^2^, Shaman Luo^2,3^, Wenrui Ma^1^, Md. Fazle Alam^2,3^, Yuyi Tang^2^, Hui Huang^2^, Nan Chen^1^, Li Wang^2^, Guoquan Yan^2^, Jun Li^1^, Hao Lai^1^, Chunsheng Wang^1^*, Kai Zhu^1^*, Weijia Zhang^1,2,3^*

^1^ Department of Cardiac Surgery and Shanghai Institute of Cardiovascular Diseases, Zhongshan Hospital, Fudan University, Shanghai 200032, China; ^2^ Institutes of Biomedical Sciences, Shanghai Medical College, Fudan University, Shanghai 200032, China; ^3^ The State Key Laboratory of Molecular Engineering of Polymers, Fudan University, Shanghai 200438, China.

**Supplementary Information**

**Table of contents:**

Supplementary File 1a. Clinical Characteristics of the patients.

Supplementary File 1b. Primary antibodies used for western blotting and immunohistochemistry.

Supplementary File 1c. The enriched canonical pathways identified by ingenuity pathway analysis (IPA).

Supplementary File 1d. Differential protein expression of mitochondrial dysfunction and oxidative phosphorylation pathways between non-diseased and BAV-TAA tissues.

Supplementary File 1e. Clinical Characteristics of the p-HAoSMCs from BAV-TAA patients.

**Supplementary File 1a. Clinical characteristics of the patients.**

|  | **Non-diseased (N=6)** | **BAV-TAA (N=6)** |
| --- | --- | --- |
| **Male, %** | 4 (66.7) | 4 (66.7) |
| **Age, years** | 62.2±7.9 | 59.3±9.8 |
| **Hypertension, %** | 4 (66.7) | 4 (66.7) |
| **Diabetes, %** | 1 (16.7) | 0 (0) |
| **Hyperlipidemia, %** | 0 (0) | 1 (16.7) |
| **Ascending aortic diameter, mm** | 30.0±1.9 | 51.2±7.0 |
| **Sinus dilation, %** | 0 (0) | 2 (33.3) |
| **Aortic regurgitation (>moderate), %** | 0 (0) | 3 (50.0) |
| **Aortic stenosis (>moderate), %** | 0 (0) | 2 (33.3) |
| **Aortic valve gradient, mean, mm Hg** | N/A | 35.8±24.6 |
| **Aortic flow jet, m/s** | N/A | 3.7±1.3 |

TAA, ascending thoracic aortic aneurysm; BAV-TAA, bicuspid aortic valve-related thoracic aortic aneurysm;

**Supplementary File 1b. Primary antibodies used for western blotting and immunohistochemistry.**

| **Antibody** | **Company (Cat. No.)** | **Working dilutions** |
| --- | --- | --- |
| DRP1 | CST (8570S) | WB:1/1000 |
| MFF | CST (84580S) | WB:1/1000 |
| MFN1 | CST (14739S) | WB:1/1000 |
| MFN2 | CST (11925S) | WB:1/1000 |
| NOTCH1 | Abcam (ab52627) | WB:1/1000 |
| NICD1 | Abcam (ab83232) | WB:1/500 |
| SM22 | Abcam (ab14106) | WB:1/1000 IF: 1/300 |
| CNN1 | Abcam (ab46794) | WB:1/1000 IF: 1/300 |
| Osteopontin (OPN) | Abcam (ab69498) | WB:1/1000 |
| β-actin | Proteintech (HRP-60008) | WB:1/5000 |

**Supplementary File 1c. The enriched canonical pathways identified by ingenuity pathway analysis (IPA).**

| **Ingenuity Canonical Pathways** | **-log(p-value)** | **Ratio** | **z-score** | **Molecules** |
| --- | --- | --- | --- | --- |
| EIF2 Signaling | 10.1 | 0.0943 | -1.941 | AGO3,AKT1,EIF1AX,EIF1AY,EIF4G3,EIF5B,GSK3B,HSPA5,PIK3R4,RALA,RPL18A,RPL32,RPL34,RPL38,RPL6,RPL7A,RPS16,RPS26,RPS7,RRAS |
| Acute Phase Response Signaling | 9.61 | 0.101 | -2.121 | AHSG,AKT1,APOA1,APOA2,C1R,CRP,HPX,HRG,ITIH2,LBP,MBL2,MYD88,ORM1,RALA,RRAS,SAA1,SERPINA3,TF |
| Regulation of eIF4 and p70S6K Signaling | 6.77 | 0.0864 | -2.646 | AGO3,AKT1,EIF1AX,EIF1AY,EIF4G3,ITGA3,ITGA5,ITGB1,PIK3R4,RALA,RPS16,RPS26,RPS7,RRAS |
| FXR/RXR Activation | 4.7 | 0.08 | #NUM! | AHSG,AKT1,APOA1,APOA2,APOF,FETUB,HPX,ORM1,SAA1,TF |
| Oxidative Phosphorylation | 4.56 | 0.0865 | -2.333 | ATP5F1A,ATP5F1E,ATP5PF,COX5B,MT-ND3,NDUFB11,NDUFB3,NDUFB7,NDUFV1 |
| Mitochondrial Dysfunction | 4.37 | 0.0667 | #NUM! | ATP5F1A,ATP5F1E,ATP5PF,COX5B,MAOA,MT-ND3,NDUFB11,NDUFB3,NDUFB7,NDUFV1,TXNRD2 |
| Role of NFAT in Regulation of the Immune Response | 4.12 | 0.0625 | -0.707 | AKT1,GNB3,GNG10,GSK3A,GSK3B,HLA-DRB1,LYN,PIK3R4,RALA,RRAS,XPO1 |
| LXR/RXR Activation | 4.04 | 0.0744 | 1.667 | AHSG,APOA1,APOA2,APOF,HPX,LBP,ORM1,SAA1,TF |
| Sirtuin Signaling Pathway | 3.97 | 0.0495 | 1.134 | AKT1,ATP5F1A,ATP5F1E,ATP5PF,GSK3B,MT-ND3,NDUFB11,NDUFB3,NDUFB7,NDUFV1,POLR1C,SLC25A4,SLC25A5,SLC25A6 |

**Supplementary File 1d. Differential protein expression of mitochondrial dysfunction and oxidative phosphorylation pathways between non-diseased and BAV-TAA tissues.**

| **Uniprot ID** | **Protein name** | **Gene symbol** | **logFC (BAV-TAA/Non-diseased)** | **p-value** |
| --- | --- | --- | --- | --- |
| Q16774 | NADH dehydrogenase [ubiquinone] 1 beta subcomplex subunit 7 | NDUFB7 | 2.463698912 | 0.011577294 |
| P18859 | ATP synthase-coupling factor 6, mitochondrial | ATP5PF | -0.606413665 | 0.039572766 |
| P21397 | Amine oxidase [flavin-containing] A | MAOA | -0.852112763 | 0.016894398 |
| O43676 | NADH dehydrogenase [ubiquinone] 1 beta subcomplex subunit 3 | NDUFB3 | -0.918257482 | 0.033659388 |
| P49821 | NADH dehydrogenase [ubiquinone] flavoprotein 1, mitochondrial | NDUFV1 | -0.76562131 | 0.025004229 |
| P56381 | ATP synthase subunit epsilon, mitochondrial | ATP5F1E | -1.094904386 | 0.031624013 |
| Q9NNW7 | Thioredoxin reductase 2, mitochondrial | TXNRD2 | -2.026106169 | 0.021612436 |
| P25705 | ATP synthase subunit alpha, mitochondrial | ATP5F1A | -0.632130045 | 0.045337778 |
| Q9NX14 | NADH dehydrogenase [ubiquinone] 1 beta subcomplex subunit 11, mitochondrial | NDUFB11 | -0.911637935 | 0.029948461 |
| P03897 | NADH-ubiquinone oxidoreductase chain 3 | ND3 | -1.534964803 | 0.006554674 |
| P10606 | Cytochrome c oxidase subunit 5B, mitochondrial | COX5B | -0.797277995 | 0.017774848 |

**Supplementary File 1e. Clinical Characteristics of the primary HAoSMCs from BAV-TAA patients**

| **Characteristics** | **B-1** | | **B-2** | **B-3** | |
| --- | --- | --- | --- | --- | --- |
| **Gender** | Female | | Male | Male | |
| **Age, years** | 57 | 46 | | | 57 |
| **Ascending aortic diameter, mm** | 55 | 53 | | | 63 |
| **Hypertension** | **+** | **+** | | | **-** |
| **Diabetes** | **-** | **-** | | | **-** |
| **Hyperlipidemia** | **-** | **-** | | | **-** |
| **Sinus** **diameter, mm** | 37 | 42 | | | 43 |
| **Aortic valve gradient, mean, mm Hg** | 15 | 27 | | | 36 |
| **Aortic flow jet, m/s** | 2.9 | 3.3 | | | 3.8 |
